# Catheter Ablation vs Drug Therapy in Patients With Atrial Fibrillation and Nonmodifiable Recurrence Risk Factors: A Secondary Analysis of the CABANA Randomized Clinical Trial

**DOI:** 10.1001/jamanetworkopen.2025.28124

**Published:** 2025-08-21

**Authors:** Zhen Wang, Yanfang Wu, Chao Jiang, Liu He, Ning Zhou, Caihua Sang, Jianzeng Dong, Changsheng Ma

**Affiliations:** 1 Department of Cardiology, Beijing Anzhen Hospital, Capital Medical University, Beijing, China; 2 Engineering Research Center of Medical Devices for Cardiovascular Diseases, Ministry of Education, Beijing, China; 3 National Clinical Research Center for Cardiovascular Diseases, Beijing, China

## Abstract

**Importance:**

Large-scale randomized studies evaluating the impact of catheter ablation on cardiovascular prognoses across different atrial fibrillation (AF) recurrence risk profiles are lacking.

**Objective:**

To investigate the benefits of catheter ablation in patients with varying numbers of nonmodifiable recurrence risk factors (NMRRFs).

**Design, Setting, and Participants:**

This study was a post hoc subanalysis of the multinational, multicenter, open-label Catheter Ablation vs Anti-Arrhythmic Drug Therapy for Atrial Fibrillation (CABANA) randomized clinical trial, for which enrollment occurred from November 2009 to April 2016, with follow-up until December 31, 2017. Individuals with AF and at least 1 stroke risk factor were recruited in the CABANA trial. Only those with complete NMRRF data were included in this secondary analysis. Data were analyzed from November 1, 2023, to May 12, 2025.

**Exposure:**

Patients were categorized into 2 subgroups based on their number of NMRRFs (<3 or ≥3 risk factors).

**Main Outcomes and Measures:**

The primary end point of the CABANA trial was death, disabling stroke, serious bleeding, or cardiac arrest. Four NMRRFs were examined: AF duration more than 1 year, persistent or long-standing persistent AF, age older than 65 years, and female sex. Multivariable Cox proportional hazards regression models with adjustment were performed to investigate the benefit of ablation in each subgroup.

**Results:**

In total, 2185 patients (median age, 67.0 years [IQR, 62.0-72.0 years]; 1373 males [62.8%]) with complete NMRRF data were included. Of these patients, 1100 (50.3%) were randomized to receive catheter ablation and 1085 (49.7%) were randomized to receive drug therapy. Most patients (1469 [67.2%]) had fewer than 3 NMRRFs, while 716 (32.8%) had 3 or more. In patients with fewer than 3 NMRRFs, catheter ablation reduced the primary end point (adjusted hazard ratio [AHR], 0.59 [95% CI, 0.41-0.86]). However, the difference was not significant in those with 3 or more NMRRFs (AHR, 1.55 [95% CI, 0.93-2.58]). The interaction between the primary end point and the NMRRF category was significant (*P* for interaction = .003). Across all NMRRF groups, ablation did not reduce all-cause mortality (<3 NMRRFs: AHR, 0.65 [95% CI, 0.41-1.02] and ≥3 NMRRFs: AHR, 1.23 [95% CI, 0.66-2.33]) but decreased AF recurrence (<3 NMRRFs: AHR, 0.46 [95% CI, 0.40-0.52] and ≥3 NMRRFs: AHR, 0.58 [95% CI, 0.49-0.69]) and improved quality of life throughout follow-up for symptom frequency (<3 NMRRFs: −1.63 [95% CI, −2.18 to −1.07] and ≥3 NMRRFs: −1.15 [95% CI, −1.98 to −0.31]).

**Conclusions and Relevance:**

In this secondary analysis of the CABANA randomized clinical trial, the findings suggest that catheter ablation yielded significant cardiovascular benefits in patients with AF with fewer than 3 NMRRFs compared with drug therapy. This study lays the foundation for more personalized AF management, potentially optimizing resource allocation and influencing the direction of research and clinical practice in this field.

**Trial Registration:**

ClinicalTrials.gov Identifier: NCT00911508

## Key Points

**Question:**

Are nonmodifiable recurrence risk factors (NMRRFs) associated with differences in benefit of catheter ablation compared with drug therapy in patients with atrial fibrillation (AF)?

**Findings:**

In this subanalysis of 2185 patients enrolled in the Catheter Ablation vs Anti-Arrhythmic Drug Therapy for Atrial Fibrillation randomized clinical trial, catheter ablation reduced the primary composite end point of death, disabling stroke, serious bleeding, or cardiac arrest in patients with fewer than 3 NMRRFs. However, it showed no significant benefit of the primary composite end point in patients with 3 or more NMRRFs.

**Meaning:**

The findings suggest that catheter ablation could improve the cardiovascular prognosis for individuals with AF with fewer than 3 NMRRFs, laying the foundation for more personalized AF management and research in the field.

## Teaser

This secondary analysis of the Catheter Ablation vs Anti-Arrhythmic Drug Therapy for Atrial Fibrillation (CABANA) randomized clinical trial examines the impact of catheter ablation compared with drug therapy in patients with atrial fibrillation (AF) and nonmodifiable recurrence risk factors.

## Introduction

Catheter ablation is primarily beneficial for symptom relief and reducing atrial fibrillation (AF) recurrence.^1,2,3,4,5,6^ However, its role in improving cardiovascular prognosis remains uncertain.

Ablation is considered the most effective method for maintaining sinus rhythm.^1,6^ Recurrence, shortly after the blanking period, is generally considered an ablation failure. In this context, predictive models underscore the importance of assessing AF recurrence risk before determining the treatment strategy.^7,8^ Several modifiable risk factors for AF recurrence are crucial determinants of treatment outcomes. These include hypertension, diabetes, heart failure, obesity, alcohol intake, physical inactivity, and sleep apnea (recognized by the European Society of Cardiology Guidelines^8^), as well as smoking (recommended for management by the American College of Cardiology/American Heart Association Joint Committee on Clinical Practice Guidelines^6^). Managing these modifiable risk factors is an independent predictor of arrhythmia-free survival.^9^ Recent guidelines and expert consensus statements reinforce the importance of addressing these factors.^6,8,10^ However, when evaluating patient eligibility for catheter ablation, predicting AF recurrence based on these modifiable factors may be inaccurate due to variations in management intensity and patient adherence after ablation.

AF duration, older age, female sex, and nonparoxysmal AF are independent, nonmodifiable risk factors for AF recurrence following ablation.^7,11^ Due to the nonmodifiable nature of these risk factors, thorough assessment is crucial when determining patient eligibility for catheter ablation. However, evidence from randomized trials assessing the effectiveness of catheter ablation in reducing major cardiovascular events among patients with varying nonmodifiable recurrence risk factors (NMRRFs) is limited, and the clinical dilemma persists for individuals with multiple NMRRFs. Therefore, in this subanalysis of the Catheter Ablation vs Anti-Arrhythmic Drug Therapy for Atrial Fibrillation (CABANA) randomized clinical trial, we aimed to investigate the benefits of catheter ablation compared with drug therapy in patients with varying numbers of NMRRFs.

## Methods

### Data Source

This post hoc secondary analysis of the multinational, multicenter, open-label CABANA randomized clinical trial analyzed data from the CABANA dataset, which is available through the National Heart, Lung, and Blood Institute’s Biologic Specimen and Data Repository Information Coordinating Center.^12^ Enrollment occurred from November 2009 to April 2016, with follow-up until December 31, 2017. Ethical approval was obtained from each site’s institutional review board or ethics committee. All participants provided written informed consent. The study followed the Consolidated Standards of Reporting Trials (CONSORT) reporting guideline for randomized clinical trials.

The original trial protocol and statistical analysis plan are provided in Supplement 1. Race (Black or African American, White, or other [including American Indian or Alaska Native, Asian, Native Hawaiian or Other Pacific Islander, and multiracial]) and ethnicity (Hispanic or Latino, non-Hispanic or non-Latino) were categorized by patients and investigators according to National Institutes of Health–specified categories^13^; these data were collected because we needed to verify that race and ethnicity were comparably distributed between the ablation and drug-therapy groups for our subgroup analyses. Patients were randomly assigned to either the catheter ablation or drug therapy group, with the trial’s design and primary outcomes already documented.^13,14,15^ Pulmonary vein isolation was standard in the ablation group, with additional techniques applied at the investigators’ discretion. Anticoagulation management followed established guidelines,^13^ with anticoagulation recommended for at least 3 months after ablation. Patients with a CHA_2_DS_2_-VASc (congestive heart failure; hypertension; age 75 years or older [doubled]; diabetes; stroke, transient ischemic attack, or thromboembolism [doubled]; vascular disease [prior myocardial infarction, peripheral artery disease, or aortic plaque]; age 65 to 75 years; and sex category [female]) score of 2 or more (scores range from 0 to 9, with higher scores indicating greater thromboembolic risk) were counseled to maintain it. In the drug therapy group, patients were advised to initiate rate-control medications, with a transition to rhythm-control drugs in cases of prior rate-control failure.

### Study Population and Design

Between November 2009 and April 2016, individuals with AF and at least 1 stroke risk factor were recruited and categorized into 2 subgroups based on the number of NMRRFs they possessed (<3 or ≥3 risk factors). The 4 NMRRFs included AF duration more than 1 year, persistent or long-standing persistent AF, age older than 65 years, and female sex. AF duration was defined as the time from the first documented diagnosis of AF to the date of study enrollment. These subgroups were further divided into 4 groups according to their assigned treatment arms.

### Outcome

The primary end points of this study were death, disabling stroke, serious bleeding, or cardiac arrest. Secondary outcomes included all-cause mortality and AF recurrence. In the primary study, the follow-up was set for an average of 48 months or longer, aiming to achieve a 90% expected statistical power level. An expert panel, blinded to treatment assignments, adjudicated the primary outcome events based on predefined criteria. AF recurrence was monitored following a 90-day blanking period. AF status was analyzed during follow-up visits. Furthermore, quality of life was assessed using the Mayo Atrial Fibrillation–Specific Symptom Inventory (MAFSI).^14^

To address potential biases in our analysis, we emulated a hypothetical randomized clinical trial. The emulated target trial framework is reported in eTables 1 and 2 in Supplement 2.

### Statistical Analysis

Data were analyzed from November 1, 2023, to May 12, 2025. Continuous data were presented as mean (SD) or median (IQR), while categorical data were expressed as count (percentage). Baseline group comparisons were conducted using the *t* test, Wilcoxon rank sum test, or χ^2^ test. Cumulative event probabilities were illustrated using Kaplan-Meier curves. Multivariable Cox proportional hazards regression models were adjusted for age, sex, race, AF type, years since the onset of AF, CHA_2_DS_2_-VASc score, history of heart failure, structural heart disease, coronary artery disease, and hypertension.^13^ The proportional hazards assumption was examined by the scaled Schoenfeld residuals test. Covariates that failed this test were handled by stratification within the multivariable Cox proportional hazards regression model. AF recurrence, with death as a competing event, was analyzed using the Fine-Gray model. Adjustments for multiple comparisons were not made because these were exploratory analyses.

In addition to the aforementioned multivariable adjustment, we also used propensity score weighting using overlap weights in subgroup analyses. The covariates balanced by propensity score weighting included age, sex, body mass index, race, AF type, AF duration, New York Heart Association class, structural heart disease, sleep apnea, thromboembolic events, heart failure, coronary artery disease, diabetes, hypertension, prior stroke or transient ischemic attack, CHA_2_DS_2_-VASc score, cigarette smoking, alcohol use, history of cardioversion for AF, prior hospitalization for AF, and family history of AF. We used propensity score–weighted Cox proportional hazards regression and propensity score–weighted Fine-Gray models to enhance covariate balance and adjust for potential confounding.

The MAFSI score (including 2 subscales: a frequency score ranging from 0 to 40, with higher scores indicating greater frequency of symptoms, and a severity score ranging from 0 to 30, with higher scores indicating greater severity) was analyzed using a repeated-measures mixed-effects model, with responses at 3, 12, 24, 36, 48, and 60 months included as outcome variables.^14^ Time, treatment, and time × treatment were considered fixed effects, and the mean differences were reported with 95% CIs. Statistical analyses were conducted using R, version 4.3.2 (R Project for Statistical Computing), with a 2-tailed *P* < .05 indicating statistical significance.

## Results

### Baseline Characteristics

**Figure 1.**
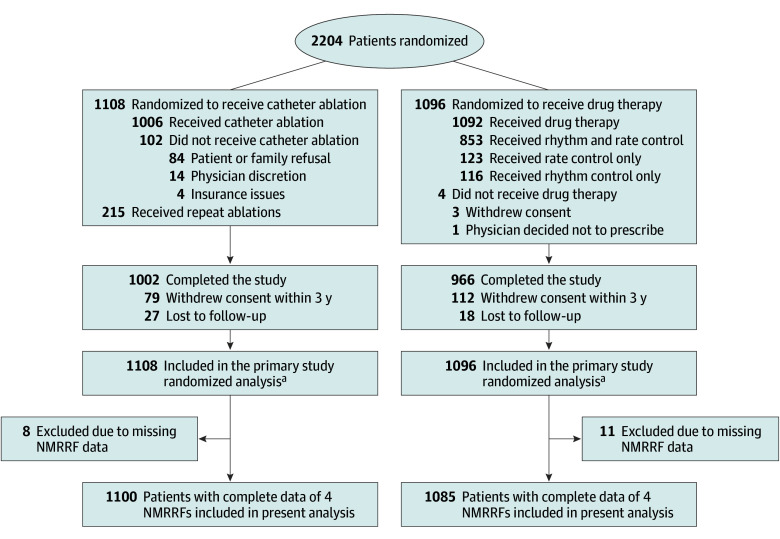
Participant Flow Diagram Due to the absence of a requirement for sites to submit screening logs during recruitment, data on the total number of patients evaluated for eligibility were unavailable. NMRRF indicates nonmodifiable recurrence risk factor. ^a^For participants who did not complete the study (eg, those who withdrew consent or were lost to follow-up), data were included up to the point of consent withdrawal or last contact. The primary and key secondary end points were assessed using a time-to-event analysis, incorporating all available follow-up data. For patients who did not finish the study and had no outcome event, time-to-event data were censored at the date of last contact. There was no imputation of outcome events. At the end of the trial, a search of publicly accessible death registries was conducted for North American participants who were lost to follow-up or withdrew.

From November 2009 to April 2016, 2204 patients from 126 sites in 10 countries were randomly assigned to receive either catheter ablation or drug therapy. Nineteen participants (0.9%) were excluded due to missing NMRRF data. In total, 2185 patients (median age, 67.0 years [IQR, 62.0-72.0 years]; 812 females [37.2%] and 1373 males [62.8%]) with complete data of 4 NMRRFs (AF duration >1 year, persistent or long-standing AF, age >65 years, and female sex) were included in the study (Figure 1). Of these patients, 1100 (50.3%) were randomized to receive catheter ablation, and 1085 (49.7%) were randomized to receive drug therapy; 1469 (67.2%) had fewer than 3 NMRRFs, while 716 (32.8%) had 3 or more. In terms of race, 77 patients (3.5%) were Black or African American, 2010 (92.1%) were White, and 95 (4.4%) were of other race; in terms of ethnicity, 61 patients (2.8%) were Hispanic or Latino and 2119 (97.2%) were non-Hispanic or non-Latino. The median follow-up period was 48 months [IQR, 29-62 months].

Baseline characteristics, as outlined in the Table, reveal that patients with fewer than 3 NMRRFs were generally younger (median age, 65.0 years [IQR, 59.0-69.5 years] vs 70.5 years [IQR, 68.0-74.0 years]) and had a shorter AF history (median time, 0.6 years [IQR, 0.2-2.8 years] vs 2.6 years [IQR, 1.0-5.1 years]), a lower CHA_2_DS_2_-VASc score (median score, 2.0 [IQR, 1.0-3.0] vs 3.0 [IQR, 3.0-4.0]), and a lower proportion of NMRRFs (persistent or long-standing persistent AF: 46.0% vs 79.2% and female sex: 19.9% vs 72.5%) compared with those of patients with 3 or more NMRRFs. Furthermore, patients with fewer than 3 NMRRFs demonstrated a lower incidence of New York Heart Association class II or more (classes range from I to IV, with higher classes indicating more severe physical limitations) (30.3% vs 46.2%) and Canadian Cardiovascular Society class more than II (classes range from 0 to IV, with higher classes indicating greater impact on quality of life due to AF symptoms) (39.9% vs 47.0%). Further differences were observed between the 2 subgroups in rates of sleep apnea (24.9% vs 19.7%), atrial flutter (14.8% vs 11.0%), prior AF hospitalization (35.8% vs 47.3%), and history of direct current cardioversion (32.0% vs 45.9%). Baseline clinical characteristics were well-balanced between treatment groups within each subgroup.

**Table.** Baseline Characteristics of Patients^a^

Characteristic	Patients with <3 NMRRFs				Patients with ≥3 NMRRFs				Overall *P* value^c^
	Overall (n = 1469)	Catheter ablation (n = 733)	Drug therapy (n = 736)	*P* value^b^	Overall (n = 716)	Catheter ablation (n = 367)	Drug therapy (n = 349)	*P* value^b^	
Sex									
Female	293 (19.9)	143 (19.5)	150 (20.4)	.68	519 (72.5)	267 (72.8)	252 (72.2)	.87	<.001
Male	1176 (80.1)	590 (80.5)	586 (79.6)		197 (27.5)	100 (27.2)	97 (27.8)		
Age, median (IQR)	65.0 (59.0-69.5)	65.0 (59.0-70.0)	65.0 (59.0-69.0)	.93	70.5 (68.0-74.0)	70.0 (68.0-74.0)	71.0 (68.0-74.0)	.76	<.001
Age category, y									
<65	697 (47.4)	343 (46.8)	354 (48.1)	.88	59 (8.2)	30 (8.2)	29 (8.3)	.90	<.001
≥65 to <75	629 (42.8)	318 (43.4)	311 (42.3)		498 (69.6)	258 (70.3)	240 (68.8)		
≥75	143 (9.7)	72 (9.8)	71 (9.6)		159 (22.2)	79 (21.5)	80 (22.9)		
Ethnicity									
Hispanic or Latino	42/1465 (2.9)	20/730 (2.7)	22/735 (3.0)	.77	19/715 (2.7)	9/366 (2.5)	10/349 (2.9)	.74	.78
Non-Hispanic or non-Latino	1423/1465 (97.1)	710/730 (97.3)	713/735 (97.0)		696/715 (97.3)	357/366 (97.5)	339/349 (97.1)		
Race									
Black or African American	58/1467 (4.0)	31/732 (4.2)	27/735 (3.7)	.79	19/715 (2.7)	8/367 (2.2)	11/348 (3.2)	.68	.01
White	1334/1467 (90.9)	662/732 (90.4)	672/735 (91.4)		676/715 (94.5)	348/367 (94.8)	328/348 (94.3)		
Other^d^	75/1467 (5.1)	39/732 (5.3)	36/735 (4.9)		20/715 (2.8)	11/367 (3.0)	9/348 (2.6)		
Body mass index, median (IQR)^e^	30.3 (26.8-34.9)	30.1 (26.7-34.6)	30.4 (26.9-35.5)	.19	29.7 (26.1-34.1)	29.7 (26.3-33.9)	29.7 (25.9-34.1)	.88	.007
Cigarette smoking									
Current	130/1465 (8.9)	68/732 (9.3)	62/733 (8.5)	.35	33/715 (4.6)	13/366 (3.6)	20/349 (5.7)	.28	<.001
Past	565/1465 (38.6)	269/732 (36.7)	296/733 (40.4)		220/715 (30.8)	109/366 (29.8)	111/349 (31.8)		
Never	770/1465 (52.6)	395/732 (54.0)	375/733 (51.2)		462/715 (64.6)	244/366 (66.7)	218/349 (62.5)		
Alcohol use									
Current	491/1465 (33.5)	261/730 (35.8)	230/735 (31.3)	.06	219/716 (30.6)	117/367 (31.9)	102/349 (29.2)	.73	<.001
Past	280/1465 (19.1)	124/730 (17.0)	156/735 (21.2)		73/716 (10.2)	36/367 (9.8)	37/349 (10.6)		
Never	694/1465 (47.4)	345/730 (47.3)	349/735 (47.5)		424/716 (59.2)	214/367 (58.3)	210/349 (60.2)		
AF severity (CCS class)									
0	164/1458 (11.2)	76/726 (10.5)	88/732 (12.0)	.86	58/715 (8.1)	29/366 (7.9)	29/349 (8.3)	.28	<.001
I	255/1458 (17.5)	125/726 (17.2)	130/732 (17.8)		82/715 (11.5)	41/366 (11.2)	41/349 (11.7)		
II	457/1458 (31.3)	235/726 (32.4)	222/732 (30.3)		239/715 (33.4)	112/366 (30.6)	127/349 (36.4)		
III	492/1458 (33.7)	245/726 (33.7)	247/732 (33.7)		285/715 (39.9)	152/366 (41.5)	133/349 (38.1)		
IV	90/1458 (6.2)	45/726 (6.2)	45/732 (6.1)		51/715 (7.1)	32/366 (8.7)	19/349 (5.4)		
Heart function severity (NYHA class) ≥II	442/1458 (30.3)	215/724 (29.7)	227/734 (30.9)	.61	329/712 (46.2)	161/366 (44.0)	168/346 (48.6)	.22	<.001
Medical history									
Hypertension or LVH, No. %)	1230 (83.7)	610 (83.2)	620 (84.2)	.60	604 (84.4)	307 (83.7)	297 (85.1)	.59	.71
Hypertension	1178 (80.2)	576 (78.6)	602 (81.8)	.12	581 (81.1)	293 (79.8)	288 (82.5)	.36	.60
Diabetes	387 (26.3)	192 (26.2)	195 (26.5)	.90	168 (23.5)	87 (23.7)	81 (23.2)	.88	.15
Sleep apnea	366 (24.9)	191 (26.1)	175 (23.8)	.31	141 (19.7)	71 (19.3)	70 (20.1)	.81	.01
Coronary artery disease	296 (20.1)	140 (19.1)	156 (21.2)	.32	127 (17.7)	67 (18.3)	60 (17.2)	.71	.18
Heart failure	225 (15.3)	113 (15.4)	112 (15.2)	.93	111 (15.5)	61 (16.6)	50 (14.3)	.40	.92
Family history of AF	174/1464 (11.9)	84/731 (11.5)	90/733 (12.3)	.64	75/715 (10.5)	45/367 (12.3)	30/348 (8.6)	.11	.34
Prior CVA or TIA	141 (9.6)	71 (9.7)	70 (9.5)	.91	75 (10.5)	45 (12.3)	30 (8.6)	.11	.52
Prior CVA	77 (5.2)	41 (5.6)	36 (4.9)	.55	47 (6.6)	27 (7.4)	20 (5.7)	.38	.21
Thromboembolic events	52 (3.5)	23 (3.1)	29 (3.9)	.41	37 (5.2)	17 (4.6)	20 (5.7)	.51	.07
Left ventricle ejection fraction ≤35%	53/1029 (5.2)	29/529 (5.5)	24/500 (4.8)	.62	16/498 (3.2)	9/264 (3.4)	7/234 (3.0)	.79	.09
Comorbidity									
CHA_2_DS_2_-VASc score, median (IQR)	2.0 (1.0-3.0)	2.0 (1.0-3.0)	2.0 (2.0-3.0)	.59	3.0 (3.0-4.0)	3.0 (3.0-4.0)	3.0 (3.0-4.0)	.50	<.001
Type of AF									
Paroxysmal	793 (54.0)	401 (54.7)	392 (53.3)	.50	149 (20.8)	67 (18.3)	82 (23.5)	.22	<.001
Persistent	570 (38.8)	275 (37.5)	295 (40.1)		461 (64.4)	245 (66.8)	216 (61.9)		
Long-standing persistent	106 (7.2)	57 (7.8)	49 (6.7)		106 (14.8)	55 (15.0)	51 (14.6)		
Time since onset of AF, median (IQR), y	0.6 (0.2-2.8)	0.6 (0.2-3.0)	0.6 (0.2-2.6)	.71	2.6 (1.1-5.4)	2.6 (1.0-5.1)	2.7 (1.1-5.8)	.37	<.001
Prior hospitalization for AF	525/1468 (35.8)	260/732 (35.5)	265/736 (36.0)	.85	338/715 (47.3)	184/366 (50.3)	154/349 (44.1)	.10	<.001
Prior direct current cardioversion of AF	470/1468 (32.0)	222/732 (30.3)	248/736 (33.7)	.17	329/716 (45.9)	172/367 (46.9)	157/349 (45.0)	.61	<.001
History of atrial flutter	217 (14.8)	104 (14.2)	113 (15.4)	.53	79 (11.0)	35 (9.5)	44 (12.6)	.19	.02
Prior ablation for atrial flutter	81/1465 (5.5)	34/730 (4.7)	47/735 (6.4)	.15	27/715 (3.8)	14/367 (3.8)	13/348 (3.7)	.96	.08

Abbreviations: AF, atrial fibrillation; CCS, Canadian Cardiovascular Society (classes range from 0 to IV, with higher classes indicating greater impact on quality of life due to AF symptoms); CHA_2_DS_2_-VASc, congestive heart failure, hypertension, age 75 years or older (doubled), diabetes, stroke/transient ischemic attack (TIA)/thromboembolism (doubled), vascular disease (prior myocardial infarction, peripheral artery disease, or aortic plaque), age 65 to 75 years, sex category (female) (scores range from 0 to 9, with higher scores indicating greater thromboembolic risk); CVA, cerebral vascular accident; LVH, left ventricular hypertrophy; NMRRFs, nonmodifiable recurrence risk factors (AF duration >1 year, persistent or long-standing persistent AF, age >65 years, and female sex); NYHA, New York Heart Association (classes range from I to IV, with higher classes indicating more severe physical limitations).

^a^ Data are presented as the No. (%) or No./total No. (%) of patients unless indicated otherwise.

^b^ Comparison between ablation group and drug group.

^c^ Comparison between overall patients with fewer than 3 NMRRFs and overall patients with 3 or more NMRRFs.

^d^ Includes American Indian or Alaska Native, Asian, Native Hawaiian or Other Pacific Islander, and multiracial.

^e^ Calculated as weight in kilograms divided by height in meters squared.

### NMRRFs

The recurrence risk model, which incorporated the treatment group and the 4 NMRRFs, demonstrated similar predictive ability for recurrence at 12 months (area under the curve, 0.66 [95% CI, 0.64-0.69] vs 0.67 [95% CI, 0.64-0.69]; *P* = .22) and 48 months (area under the curve, 0.68 [95% CI, 0.63-0.73] vs 0.68 [95% CI, 0.63-0.73]; *P* = .73) following the blanking period compared with the model that also included modifiable risk factors (body mass index, alcohol consumption, smoking, diabetes, hypertension, heart failure, and sleep apnea) (eFigure 1 in Supplement 2). In the ablation group, Kaplan-Meier curves revealed a higher recurrence rate among patients with more NMRRFs (eFigure 2A in Supplement 2). Patients with fewer than 3 NMRRFs exhibited a significantly lower recurrence rate compared with patients with 3 or more NMRRFs (eFigure 2B in Supplement 2).

After adding left atrial diameter (LAD) into the analysis, only 1092 patients (50.0%) had complete data on 5 NMRRFs (LAD >45 mm, AF duration >1 year, persistent or long-standing AF, age >65 years, and female sex). Among these 1092 patients, the recurrence risk model incorporating the treatment group and 4 NMRRFs demonstrated comparable predictive accuracy for recurrence at the 12-month and 48-month postblanking period when compared with models that additionally included LAD or both LAD and modifiable risk factors (eFigure 3 in Supplement 2).

### Clinical Outcomes by Intention-to-Treat

**Figure 2.**
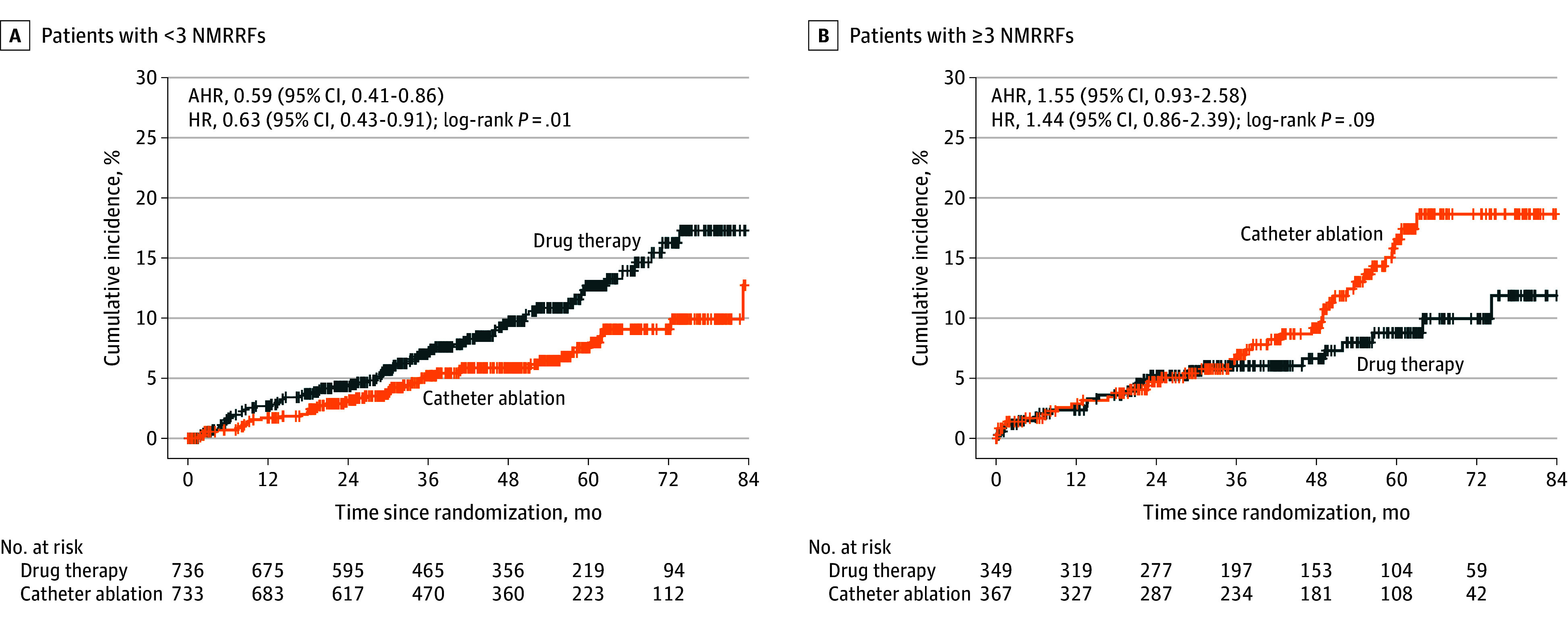
Kaplan-Meier Curves for Catheter Ablation vs Anti-Arrhythmic Drug Therapy for Atrial Fibrillation Trial Primary End Points The adjusted hazard ratio (AHR) is the hazard ratio (HR) estimated by multivariable Cox proportional hazards regression. The HR is estimated by propensity score–weighted Cox proportional hazards regression. NMRRF indicates nonmodifiable recurrence risk factor.

**Figure 3.**
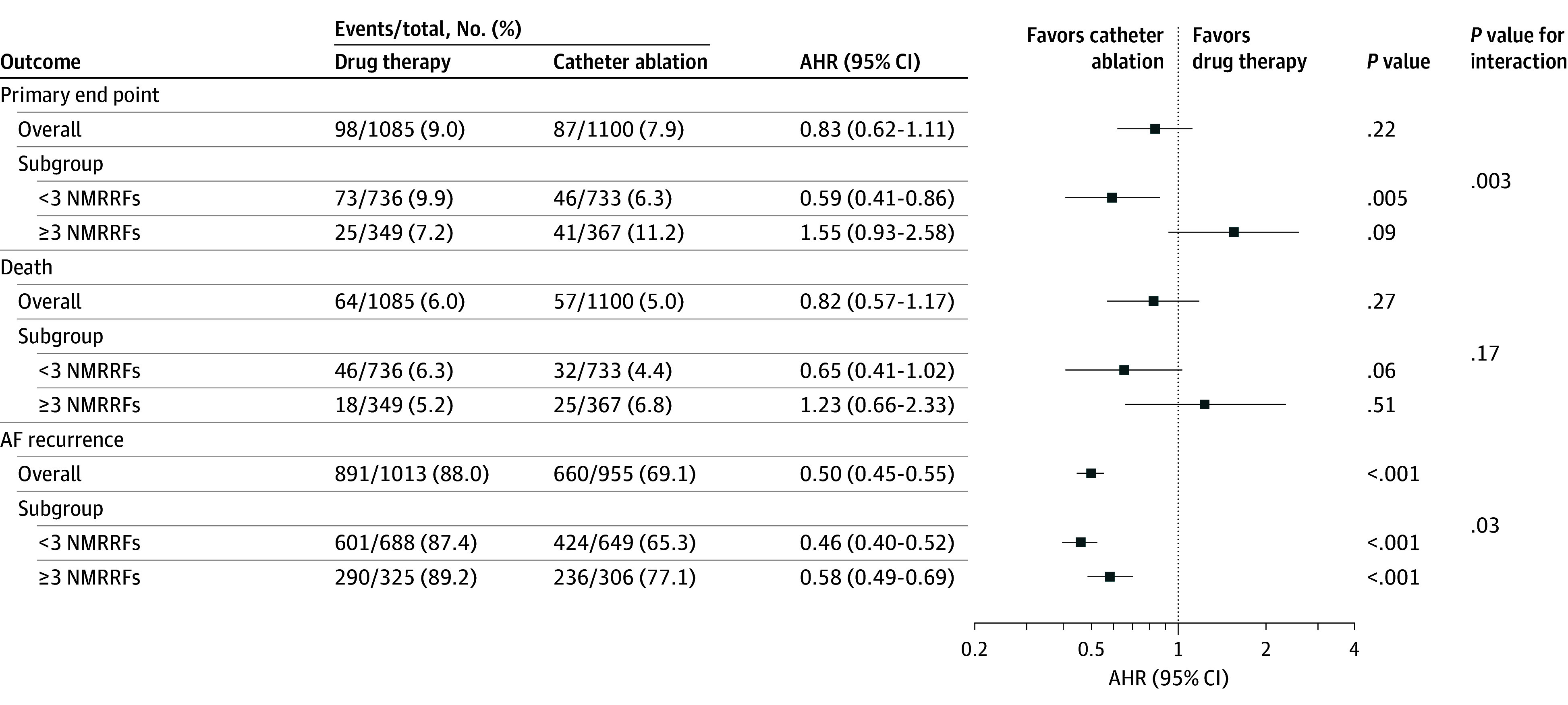
Adjusted Hazard Ratios (AHRs) for Catheter Ablation vs Anti-Arrhythmic Drug Therapy for Atrial Fibrillation (AF) Trial End Points by Intention-to-Treat NMRRF indicates nonmodifiable recurrence risk factor.

During a median follow-up of 48 months [IQR, 29-62 months], ablation significantly reduced the risk of the primary outcome in patients with fewer than 3 NMRRFs (adjusted hazard ratio [AHR], 0.59 [95% CI, 0.41-0.86]). However, this was not observed in patients with 3 or more NMRRFs (AHR, 1.55 [95% CI, 0.93-2.58]), as indicated by *P* = .003 for interaction (Figure 2 and Figure 3). The difference for all-cause mortality was not statistically significant in patients with fewer than 3 NMRRFs (AHR, 0.65 [95% CI, 0.41-1.02]). Similarly, in patients with 3 or more NMRRFs, the difference in all-cause mortality was not significant (AHR, 1.23 [95% CI, 0.66-2.33]) (*P* = .17 for interaction) (Figure 3). The propensity score–weighted analysis demonstrated similar results (eFigure 4 in Supplement 2), with absolute standardized mean differences for all covariates less than 0.1 following overlap weighting.

Segmenting patients into 3 subgroups based on the NMRRF count (≤1, 2, and ≥3 NMRRFs) indicated that the difference was not significant but that patients with fewer than 3 NMRRFs benefited more from ablation in cardiovascular outcomes for the primary end point (≤1: AHR, 0.61 [95% CI, 0.35-1.07] and 2: AHR, 0.61 [95% CI, 0.37-1.00]; *P* = .01 for interaction) and for mortality (≤1: AHR, 0.66 [95% CI, 0.34-1.28] and 2: AHR, 0.68 [95% CI, 0.36-1.27]; *P* = .41 for interaction) (eFigure 5 in Supplement 2). The propensity score–weighted analysis demonstrated similar findings (eFigure 6 in Supplement 2). Following overlap weighting, the absolute standardized mean differences for all covariates were less than 0.1.

Both subgroups experienced reduced AF recurrence compared with those receiving drug therapy (<3 NMRRFs: AHR, 0.46 [95% CI, 0.40-0.52] and ≥3 NMRRFs: AHR, 0.58 [95% CI, 0.49-0.69]; *P* = .03 for interaction) (Figure 3). When patients were categorized into 3 subgroups based on NMRRF count (≤1, 2, and ≥3 NMRRFs), all exhibited significantly lower recurrence rates, highlighting a clear trend in which fewer NMRRFs led to greater reductions (≤1: AHR, 0.41 [95% CI, 0.34-0.50] and 2: AHR, 0.48 [95% CI, 0.41-0.57]; *P* = .01 for interaction) (eFigure 5 in Supplement 2). The propensity score–weighted analysis demonstrated similar findings (eFigure 6 in Supplement 2). After overlap weighting, the absolute standardized mean differences for all covariates were less than 0.1.

### Quality of Life

**Figure 4.**
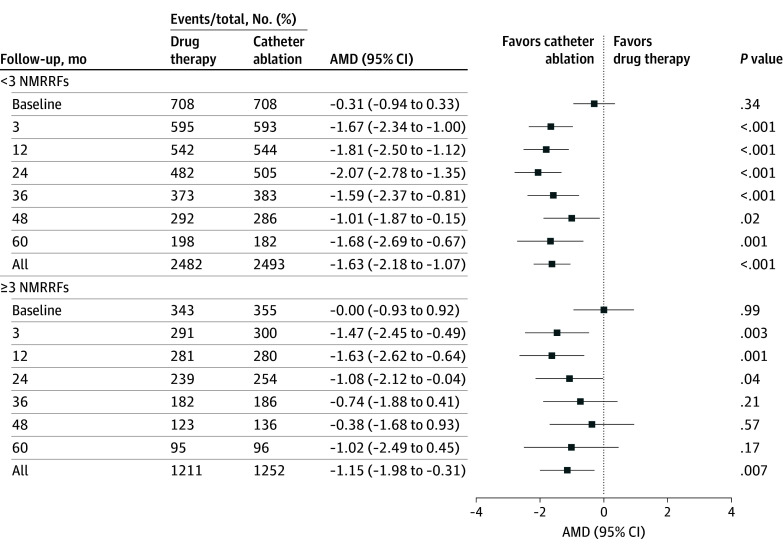
Quality-of-Life Outcomes by Mayo Atrial Fibrillation–Specific Symptom Inventory (MAFSI) Frequency Scoring AMD indicates adjusted mean difference; NMRRF, nonmodifiable recurrence risk factor.

The intention-to-treat analysis revealed that ablation therapy significantly reduced both the MAFSI frequency and severity scores in patients with fewer than 3 NMRRFs both at 12 months (frequency: −1.81 [95% CI, −2.50 to −1.12]; severity: −1.59 [95% CI, −2.14 to −1.04]) and throughout the follow-up (frequency: −1.63 [95% CI, −2.18 to −1.07]; severity: −1.27 [95% CI, −1.72 to −0.83]) (Figure 4 and eFigure 7 in Supplement 2). In patients with 3 or more NMRRFs, ablation also favored reductions in both MAFSI frequency and severity scores (frequency: −1.15 [95% CI, −1.98 to −0.31]; severity: −1.00 [95% CI, −1.68 to −0.32]) throughout the follow-up. The 12-month differences were similarly significant (frequency: −1.63 [95% CI, −2.62 to −0.64]; severity: −1.48 [95% CI, −2.27 to −0.68]) (Figure 4 and eFigure 7 in Supplement 2).

## Discussion

This study provides the first randomized clinical trial evidence, to our knowledge, that catheter ablation, compared with drug therapy, improves cardiovascular prognosis in patients with AF and fewer than 3 NMRRFs. Ablation also diminished AF recurrence and enhanced quality of life, irrespective of NMRRF count.

### NMRRFs

In our study, 4 established NMRRFs were selected: AF duration more than 1 year, persistent or long-standing persistent AF, age older than 65 years, and female sex.^7,16^ Extensive research has linked nonparoxysmal AF and older age to higher rates of postablation AF recurrence.^7,11^ These 2 factors are commonly incorporated into recurrence-risk prediction models, such as APPLE (age, persistent AF, impaired estimated glomerular filtration rate, LAD, and left ventricular ejection fraction),^17^ DR-FLASH (diabetes, kidney dysfunction, persistent form of AF, LAD, age, female sex, and hypertension),^18^ ATLAS (age, type of AF, left atrial volume, sex, and smoking),^19^ and CAAP-AF (coronary artery disease, LAD, age, persistent or long-standing AF, the number of antiarrhythmic drugs failed, and female sex).^20^ Recent meta-analyses have indicated a nonsignificant trend toward a higher recurrence risk in older patients.^21,22^ However, the heterogeneity of the studies included in these meta-analyses necessitates caution in interpretation. Similarly, female sex is recognized as an independent risk factor for AF recurrence in DR-FLASH, ATLAS, and CAAP-AF scores.^18,19,20,23^ Furthermore, a meta-analysis provided strong evidence supporting the association between diagnosis-to-ablation time and AF recurrence.^24^

Numerous modifiable recurrence risk factors have been identified, including obesity,^25^ hypertension,^26^ diabetes,^27^ sleep apnea,^28,29^ and heart failure.^30^ However, many other studies have failed to establish a consistent association between these factors and recurrence after ablation.^11,24^ This disparity may be attributed to variations in the management intensity of modifiable recurrence risk factors and patient adherence. Similarly, in our study, the recurrence prediction model, including 4 NMRRFs, demonstrated comparable predictive potential with the model that included both NMRRFs and modifiable factors.

In the CABANA trial, approximately half of the patients lacked LAD data. Both recurrence prediction models exhibited similar predictive potential with and without LAD. It is well established that AF duration, AF type, and LAD are interrelated, suggesting the possibility of a mediating effect among these factors in relation to AF recurrence. Our findings revealed that using 4 NMRRFs to evaluate AF recurrence may have been sufficient. However, caution is advised when interpreting the results since only half of the patients had LAD data.

In clinical practice, assessing recurrence risk with too many factors is cumbersome. Therefore, our research focused on 4 NMRRFs. Our findings revealed that patients in the ablation arm with fewer than 3 NMRRFs had significantly lower recurrence risk than those with 3 or more NMRRFs, indicating the feasibility of predicting recurrence risk based on these NMRRFs.

### Catheter Ablation for AF With Different Numbers of NMRRFs

Notably, in our study, ablation significantly reduced the risk of the primary end point among patients with fewer than 3 NMRRFs. However, in patients with 3 or more NMRRFs, the difference was not significant. The AF burden is believed to influence cardiovascular prognosis,^31^ as evidenced by the CASTLE-AF (Catheter Ablation vs Standard Conventional Treatment in Patients With Left Ventricular Dysfunction and AF) trial, in which postablation AF burden predicted adverse clinical outcomes in patients with AF and heart failure.^32^ Our results indicated variability in recurrence rates among patients in different recurrence risk subgroups within the ablation arm (eFigure 2 in Supplement 2). Furthermore, patients with different NMRRF counts exhibited divergent benefits through ablation regarding recurrence (eFigure 5 in Supplement 2). Therefore, we hypothesized that patients with fewer than 3 NMRRFs may achieve much greater AF burden reduction through ablation, potentially leading to improved cardiovascular prognosis. Observational studies have suggested that AF recurrence is associated with subsequent cardiovascular events.^33^ Large-scale studies with rigorous postablation monitoring of AF burden are warranted to clarify the relationships among NMRRFs, postablation AF burden, and cardiovascular events, as well as to elucidate the mechanisms linking these factors.

In the EAST (Early Treatment of Atrial Fibrillation for Stroke Prevention Trial) study, early rhythm control was shown to reduce the primary composite outcome of cardiovascular death, stroke, or hospitalization due to heart failure or acute coronary syndrome in patients with multiple comorbidities. However, this benefit was not observed in those with fewer comorbidities.^34^ This finding seems to differ from ours. This discrepancy could be attributed to patients with fewer comorbidities in the EAST study having a lower incidence of end point events, leading to a nonsignificant difference due to insufficient sample size. Furthermore, the treatment strategies in the EAST study and CABANA trial differ. The EAST study mainly compared early rhythm-control drugs with rate-control drugs,^35^ while the CABANA trial compared catheter ablation with drug therapy, with 88.4% of patients in the latter group receiving rhythm-control drugs.^13^

The CASTLE-AF^36^ and CASTLE-HTx (Catheter Ablation for Atrial Fibrillation in Patients With End-Stage Heart Failure and Eligibility for Heart Transplantation)^37^ studies demonstrated that catheter ablation significantly benefits patients with an ejection fraction less than 35% and those with end-stage heart disease, particularly in terms of hard clinical outcomes. However, our findings suggest that more NMRRFs correlate with a lower prognosis benefit. Notably, only about 5% of patients in the CABANA trial had an ejection fraction less than 35%.^13^ In the CASTLE-AF and CASTLE-HTx trials, AF episodes, especially those with rapid ventricular rates, can be potentially fatal due to already compromised cardiac function. Therefore, catheter ablation may significantly improve prognosis by reducing AF recurrence during follow-up. However, in the CABANA trial, the impact of AF on prognosis may take longer to manifest. For patients with 3 or more NMRRFs, in which AF recurrence reduction through ablation is lower, ablation-related prognosis improvement may extend beyond the follow-up period.

Notably, in the CABANA study, only 32.8% of patients presented with 3 or more NMRRFs, which significantly limited the sample size for this subgroup. This small cohort size necessitates cautious interpretation of the observed divergence in Kaplan-Meier curves beginning at 36 months after randomization. At this time point, the number of patients remaining in the analysis was further reduced, potentially amplifying variability and reducing statistical power. Additionally, propensity score–weighted analyses revealed smaller differences between catheter ablation and medical therapy for the primary end point in this subgroup compared with the multivariable Cox proportional hazards regression analysis. Importantly, both analytical approaches indicated no statistically significant difference between the 2 treatment modalities in patients with 3 or more NMRRFs. Therefore, the divergence observed after 36 months is likely attributable to the limited sample size and reduced statistical robustness rather than a definitive indication of treatment superiority. An observational study involving 183 760 patients with AF demonstrated that catheter ablation significantly reduced cardiovascular events.^38^ In contrast, Canadian observational data revealed a reduction in mortality through rhythm control since the beginning of the fifth year.^39^ These findings underscore the importance of extended follow-up trials with larger sample sizes to precisely evaluate the cardiovascular benefits of ablation in patients with 3 or more NMRRFs. Recent evidence demonstrates that pulmonary vein isolation with optimized linear ablation effectively reduces AF recurrence.^40^ In subsequent studies, advancements in catheter ablation techniques may improve the cardiovascular prognosis for patients with 3 or more NMRRFs.

### Limitations

This study has certain limitations. First, as a post hoc analysis of a randomized clinical trial, the study was not prespecified, and the subgroup analysis was not powered enough. The CABANA trial did not use stratified randomization based on the number of NMRRFs. Nevertheless, our findings remained significant after multivariable adjustments. Second, the generalizability of our findings may be limited for groups who were underrepresented in CABANA, such as those with end-stage heart failure. In addition, the CABANA study lacked data on patients’ gender-transition status. Consequently, we could not incorporate this factor into our analysis, potentially limiting the generalizability of our findings to transgender individuals. Third, only participants with complete NMRRF data were analyzed, and selection bias was possible.

## Conclusions

In this secondary analysis of the CABANA randomized clinical trial, the findings suggest that catheter ablation compared with drug therapy could improve cardiovascular prognosis in patients with fewer than 3 NMRRFs. For patients with 3 or more NMRRFs, it remains an effective strategy to reduce AF recurrence and improve quality of life. This study provides a foundation for a more personalized approach to AF management, potentially enhancing patient outcomes and resource allocation. These findings may help guide future research and clinical practice.

## Notes

Supplement 1.
Trial Protocol

Supplement 2. **eFigure 1.** Time-Dependent ROC Curve Analysis of Different Recurrence Prediction Models **eFigure 2.** Kaplan-Meier Curves for AF Recurrence in the Ablation Arm **eFigure 3.** Time-Dependent ROC Curve Analysis of Different Recurrence Prediction Models in Patients With Echocardiograms **eFigure 4.** Propensity Score–Weighted Hazard Ratios of End Points by Intention-to-Treat **eFigure 5.** Adjusted Hazard Ratios of End Points by Intention-to-Treat Among Patients With Different Numbers of NMRRFs **eFigure 6.** Propensity Score–Weighted Hazard Ratios of End Points by Intention-to-Treat Among Patients With Different Numbers of NMRRFs **eFigure 7.** Quality-of-Life Outcomes by MAFSI Severity Scoring **eTable 1.** Emulated Target Trial Framework for Prognosis Among Patients With AF and Fewer Than 3 NMRRFs **eTable 2.** Emulated Target Trial Framework for Prognosis Among Patients With AF and 3 or More NMRRFs

Supplement 3.
Data Sharing Statement
