# Supplementary material for: Catheter Ablation vs Drug Therapy in Patients With Atrial Fibrillation and Nonmodifiable Recurrence Risk Factors: A Secondary Analysis of the CABANA Randomized Clinical Trial
Source: JAMA Netw Open. 2025 Aug 21;8(8):e2528124. doi: 10.1001/jamanetworkopen.2025.28124 (PMC12371518; doi:10.1001/jamanetworkopen.2025.28124)
Supplement: Supplement 1. — Trial Protocol [file jamanetwopen-e2528124-s001.pdf]

Catheter **Ab**lation Versus **An**tiarrhythmic Drug Therapy for **A**trial Fibrillation Trial

Grant Number: HL089709
Grant Number: HL089786
Grant Number: HL089907
Grant Number: HL089645

CABANA TRIAL

Version: 3.5
Date: 22 November, 2013
IDE: G050233
NCT: 00911508
EudraCT-Number: 2011-002532-12

Principal Investigator:

Douglas L. Packer, MD
Mayo Clinic
St. Marys Hospital
Alfred 2-416
1216 2nd St. SW
Rochester, MN 55902

This document contains information that is privileged or confidential and may not be disclosed unless such disclosure is required by applicable laws and regulations. The information in this document is the property of Mayo Clinic and may not be reproduced, published, or disclosed to others without written authorization from Mayo Clinic.

Contact Information

CABANA Clinical and Administrative Center

Mayo Clinic/St. Marys Hospital Al 2-416
1216 2nd St. SW
Rochester, MN 55902

Principal Investigator: Douglas L. Packer, MD
Phone: 507-255-6263
Fax: 507-255-3292
packer.douglas@mayo.edu

Project Director: Kristi H. Monahan, RN
Phone: 507-255-7456
Fax: 507-255-9608
monahan.kristi@mayo.edu

National Heart Lung and Blood Institute

NHLBI
Two Rockledge Centre
Suite 8170, MSC 7940
6701 Rockledge Dr., Rm. 8146
Bethesda, MD 20892-7956
(Fed-Ex Zip code 20817)

Project Officer: Yves D. Rosenberg, MD, MPH
Phone: 301-435-1292
Fax: 301-480-3667
rosenbey@nih.gov

Statistics, Data and Trial Operations Coordinating Center

Duke Clinical Research Institute
2400 Pratt Street
Durham, NC 27705

Principal Investigator: Kerry L. Lee, PhD
Phone: 919-668-8725
Fax: 919-668-7055
kerry.lee@duke.edu

Project Leader: Kathleen L. Moretz, RN
Phone: 919-668-8277
Cell: 919-818-9461
Fax: 919-668-7105
kathleen.moretz@duke.edu

Economic and Quality of Life Coordinating Center

Duke Clinical Research Institute
2400 Pratt Street
Durham, NC 27705

Principal Investigator: Daniel B. Mark, MD, MPH
Phone: 919-668-8775
Fax: 919-668-7060
daniel.mark@duke.edu

Project Leader: Tina Harding, BSN, CCRA
Phone: 919-668-8431
Fax: 919-668-9817
tina.harding@duke.edu

Mayo Biomedical Imaging Resource Center

Mayo Clinic
200 1st St. SW
Medical Science 1
Rochester, MN 55905

Principal Investigator: Richard Robb, PhD
Phone: 507-284-2997
Fax: 507-284-1632
robb.richard@mayo.edu

Co-Investigator: David Holmes, III PhD
Phone: 507-284-2997
Fax: 507-284-1632
holmes.david3@mayo.edu

Co-Investigator: Maryam Rettmann, PhD
Phone: 507-284-2997
Fax: 507-284-1632
rettmann.maryam@mayo.edu

CABANA Core Labs

University of Washington
Division of Cardiology
1959 NE Pacific Street
Box 356422
Seattle, WA 98195

Director ECG/EGM: Jeanne Poole, MD
Phone: 206-685-4176
Fax: 206 616 1022jpoole@u.washington.edu

Seattle Institute Cardiac Research
2710 California Ave. SW, #3410
Seattle, WA 98116
206-529-8333

Director-Clinical Affairs: Jill Anderson, RN, BSN
Phone: 206-529-1114
jander@sicr.org

Medicomp, Inc
7845 Ellis Road
Melbourne, FL 32904
1-800-234-3278 or 1-321-676-0010

President and COO: Dan Balda, MD
Phone: 321-676-0010 x111
dbalda@medicomp.com

Clinical Director: Mauri Wilson, RN
Phone: 321-676-0010 x105
mwilson@medicomp.com

Protocol Synopsis

SPONSOR	National Heart Lung and Blood Institute
PROTOCOL TITLE	**C**atheter **Ab**lation Versus **An**tiarrhythmic Drug Therapy for **A**trial Fibrillation Trial (CABANA)
PROTOCOL TYPE	Investigator Initiated<br>IDE: G050233<br>NCT: 00911508<br>EudraCT-Number: 2011-002532-12
DIAGNOSIS AND MAIN CRITERIA FOR INCLUSION	Subjects who have new onset or under-treated paroxysmal, persistent, or longstanding persistent AF who ***warrant therapy*** for their arrhythmia, that (1) Over the preceding **6** months have:<br>a) ≥2 paroxysmal episodes (***electrocardiographic documentation*** of at least 1 episode) lasting ≥1 hour in duration: (that terminate spontaneously within 7 days or cardioversion is performed within 48h of AF onset)<br>b) ***electrocardiographic documentation*** of 1 persistent AF episode: (sustained for ≥7 days **or** cardioversion is performed more than 48h after AF onset);<br>c) ***electrocardiographic documentation*** of 1 longstanding persistent AF episode: (continuous AF of duration >1 year). (2) are eligible for catheter ablation, (3) are eligible for ≥2 membrane active drugs and/or ≥2 rate control drugs, and (4) are ≥65 yrs of age or <65 yrs with one or more risk factors for stroke (hypertension, diabetes, heart failure, prior stroke or TIA or systemic emboli, Atherosclerotic vascular disease (previous MI, peripheral arterial disease or aortic plaque), or left atrial diameter ≥ 5.0 cm or left atrial volume ≥ 40 cc/m^2). Eligible subjects with persistent or long-standing persistent AF will require at least 1 documented episode. See main protocol for complete inclusion/exclusion criteria.
STUDY HYPOTHESIS	The treatment strategy of percutaneous left atrial catheter ablation for the purpose of eliminating atrial fibrillation (AF) is superior to current state-of-the-art therapy with either rate control or rhythm control drugs for reducing a) the composite endpoint of total mortality, disabling stroke, serious bleeding, or cardiac arrest (primary endpoint; previously the key secondary endpoint) and decreasing total mortality (secondary endpoint; previously the primary endpoint) in subjects with untreated or incompletely

SPONSOR	National Heart Lung and Blood Institute
	treated AF warranting therapy.
STUDY DESIGN	Multicenter, prospective, randomized, open-label clinical trial.
TREATMENT REGIMEN(S)	One-half of the subjects will be randomly allocated to treatment with catheter ablation and one-half will be allocated to drug therapy for either rate control or rhythm control.
DURATION OF STUDY PARTICIPATION	Enrollment will occur over approximately 4 years, and subjects will be followed for an average of approximately 5 years.
NUMBER OF SUBJECTS	2000-2200 with a 1:1 randomization ratio
NUMBER OF SITES	Total number: approximately 180<br>North American Sites: approximately 120<br>Non-North American Sites: approximately 60
PRIMARY ENDPOINT	Composite of: 1) total mortality, 2) disabling stroke, 3) serious bleeding, or 4) cardiac arrest.
SECONDARY OUTCOMES	Total mortality,<br>Total mortality or cardiovascular hospitalization,<br>Total mortality or stroke or cardiovascular hospitalization,<br>Cardiovascular death,<br>Cardiovascular death or disabling stroke,<br>Arrhythmic death or cardiac arrest,<br>Heart failure death,<br>Freedom from recurrent AF,<br>Cardiovascular hospitalization,<br>Medical costs and resource use and cost effectiveness,<br>Quality of life,<br>Composite adverse events,<br>LA size, morphology and function.
INTERIM ANALYSES	An interim examination of key safety and endpoint data will be performed at regular intervals during the course of the trial. The primary objective of these analyses will be to evaluate the accumulating data for a high frequency of negative clinical outcomes. The interim monitoring will also involve a review of the control arm event rates, patient recruitment, compliance with the study protocol, status of data collection, and other factors which reflect the overall progress and integrity of the study.

Investigator Statement

I have read the protocol, including all appendices and agree that it contains all necessary details for me and my staff to conduct this study as described. I will personally oversee the conduct of this study as outlined herein and will make a reasonable effort to complete the study within the time designated.

I will provide all study personnel under my supervision with copies of the protocol and access to all information provided by the Sponsor and or designee. I will discuss this material with them to ensure that they are fully informed about the conduct of the study in general. I am aware that, prior to the commencement of this study, the Institutional Review Board responsible for such matters must approve this protocol in the clinical facility where it will be conducted. I agree to make all reasonable efforts to adhere to the attached protocol.

I agree to provide all subjects with informed consent forms, as required by government and International Conference of Harmonization regulations. I further agree to report any adverse experiences in accordance with the terms of this protocol and FDA regulation 21 CFR 812 (Subpart E).

____ ________________

(Signature of Principal Investigator) (Date)

____

(Please Print: Principal Investigator)

Table of Contents

Contact Information 2

Protocol Synopsis 5

Investigator Statement 7

Table of Contents 8

1.0 Introduction 12
1.1 Background 13
1.1.1 Increasing Incidence and Prevalence 13
1.1.2 Progression of AF 13
1.1.3 Health Care Costs of AF 13
1.2 Clinical Experience with Drug Therapy 14
1.2.1 Anti-Thrombotic Therapy 14
1.2.2 Drug Therapy for Rate Control 14
1.2.3 Drug Therapy for Maintaining Sinus Rhythm 14
1.2.4 Mortality Benefit of Drug Therapy in Randomized Trials 14
1.3 Clinical Experience with Catheter Ablation 16
1.3.1 Single Center Ablation Success Rates 16
1.3.2 CABANA Pilot Study 17
1.4 Rationale for Maintaining Sinus Rhythm 18
1.5 Rationale for Rhythm Monitoring 18
1.6 Rationale for Cost and Quality of Life Investigators 18
1.7 Rationale for Cardiac Imaging Studies 18
1.8 Significance of Trial 19

2.0 Objectives 19
Changes to the Protocol from the Original Study Design 19
2.1. Primary Objective and Hypothesis 20
2.2 Secondary Endpoints/Objectives 20
2.2.1 Total Mortality 20
2.2.2 Composite Mortality / Cardiovascular Hospitalization 20
2.2.3 Freedom from Heart Failure Mortality 20
2.2.4 Freedom from Recurrent Atrial Fibrillation 20
2.2.5 Cost/Economic Impact 21
2.2.6 Quality of Life 21
2.2.7 Adverse Events 21
2.2.8 LA Size and Function 21

3.0 Subject Selection 21
3.1 Inclusion Criteria 21
3.2 Exclusion Criteria 23

4.0 Overview of Study 24
4.1 Trial Design and Time Line 23
4.2 Screening and Pre-randomization Process 23
4.3 Enrollment and Randomization 23

4.4 Post Randomization Procedures 24
4.4.1 Baseline Testing 24
4.4.2 Patient Follow Up 25
4.4.3 Schedule of Assessment 26

5.0 Treatment Arms 26
5.1 Description of Treatment Arms 26
5.1.1 Treatment Strategies 26
5.2 Pharmacologic Approach to AF (Drug Therapy) 27
5.2.1 Drugs Approved for Rate Control 27
5.2.2 Drugs Approved for Maintenance of Sinus Rhythm 27
5.2.3 Drugs for Future Consideration 28
5.2.4 AV Nodal Ablation 28
5.2.5 Changing Drug Therapy 28
5.2.6 Cross-Over Ablative Therapy 28
5.2.7 Compliance with Treatment Protocols/Site Performance 28
5.3 Primary Catheter Ablation for AF (Ablation Therapy) 29
5.3.1 Pre-ablation Assessment 29
5.3.2 Ablation Therapies 29
5.3.3 Approved Ablation Devices 29
5.3.4 Approaches for Futures Consideration 30
5.3.5 Cross-over to Drug Therapy 30
5.3.6 Compliance with Treatment Protocols/Site Performance 30
5.4 Guidelines for Anti-thrombotic Therapy 30
5.4.1 Guidelines for Anticoagulation in Drug Treated Patients 30
5.4.2 Guidelines for Anti-thrombotic Therapy in Ablation Patients 31
5.4.3 Newer Antithrombotic Therapies 31
5.4.4 Items Left to the Discretion of the Physician 31
5.5 Management of Arrhythmia Recurrences During Follow-Up 31

6.0 Definitions & Adjudication for Study Endpoints 32
6.1 Primary Endpoints 32
6.2 Secondary Endpoints & Safety Endpoints 32

7.0 Adverse Events 32
7.1 Adverse Event Classification 33
7.1.1 Anticipated Adverse Events 33
7.1.2 Serious Adverse Event 33
7.1.3 Adverse Event Intensity or Severity Classification 34
7.1.4 Casual Relationship to Medical or Ablative Therapy 34
7.1.5 Device Related Adverse Events 34
7.2 Adverse Event Reporting 35
7.3 Serious Adverse Event Reporting 35
7.4 Expedited Event Reporting 35
7.4.1 Expedited events include: 35
7.5 Adverse Event Documentation 36
7.6 Reporting to Regulatory Authorities 36
7.6.1 Drug Therapy 36
7.6.2 Device Therapy 36
7.6.3 Criteria for Withdrawal of Subjects from Study 37

8.0 Statistical Considerations....37
8.1 Sample Size and Power Considerations 37
8.2 Statistical Analysis 39
8.2.1 Analysis of the Primary Endpoint 39
8.2.2 Analysis of Secondary Endpoints 40
8.2.3 Analysis of Morbidity (Adverse Events) 40
8.2.4 Analysis of Core Lab Measures of LA Size, Morphology and Function...40
8.2.5 "On-Treatment" Analysis 41
8.2.6 Interim Analyses 41
8.2.7 Multiple Comparisons 42
8.2.8 Health Economics Analyses 42
8.2.9 Quality of Life (QOL) Analyses 43

9.0 Data Management and Quality Control 43
9.1 Study Data Collection - All Patients 43
9.2 Electronic Case Report Form (eCRF) 43
9.3 Data Management and Quality 44
9.4 Economic and Quality of Life (EQOL) Data....44

10.0 Investigator Responsibility/Performance 44

11.0 Study Data Reporting and Processing 45
11.1 Site Selection and Monitoring 45
11.1.1 Initiating/Training Sites....46
11.1.2 Terminating a Site 46
11.1.3 Conflicts of Interest 46

12.0 Study Documentation 46

13.0 Source Documentation 47

14.0 Protocol Deviations....47

15.0 Data Transmittal and Record Retention....48

16.0 Study Closeout 48

17.0 Audit/Inspections 48

18.0 Informed Consent....48

19.0 Confidentiality of Subjects 49

20.0 Authorization for Use and Disclosure of Protected Health Information (HIPAA) 49

21.0 Human Subject Protections 50

21.1 Research Subject Selection and Justification of Exclusions 50

22.0 Institutional Review Board/Ethics Committee Review 50

23.0 Publications Policies 50

24.0 Sub-Studies 51
24.1 CABANAgene 51

25.0 References 52

Appendix A Anticipated Adverse Events 59

Appendix B Approved Ablation Devices 61

Appendix C CABANAgene Sub-study 62

1.0 Introduction

This protocol describes The Catheter Ablation Versus Anti-arrhythmic Drug Therapy for Atrial Fibrillation (CABANA) Trial which has the overall goal of establishing the appropriate roles for medical and ablative intervention for atrial fibrillation (AF) in patients who require AF treatment and are at an increased risk for mortality, stroke and other health-related complications associated with AF. The trial aims to enroll a sufficiently broad spectrum of patients to allow far-reaching applicability of the results to the dramatically increasing numbers of patients with this arrhythmia. AF specifically affects patients of advancing age, and is an escalating burden to the health care system. This trial arises out of the AFFIRM, RACE, STAF, and AF CHF investigations of rate vs. rhythm control therapy and the International AF Ablation Registry. An ablation trial evaluating overall mortality and major AF related events, conducted within a population at increased risk, will provide the most compelling evidence for guiding the therapy of this malady. The completion of the 60 patient CABANA Pilot Study provides solid evidence of the feasibility of this landmark study. The primary aim of the CABANA Trial is to test the hypothesis that the treatment strategy of percutaneous left atrial catheter ablation for the purpose of eliminating atrial fibrillation (AF) is superior to current state-of-the-art therapy with either rate control or rhythm control drugs for reducing: 1) total mortality, disabling stroke, serious bleeding, or cardiac arrest as the primary endpoint and 2) total mortality as the secondary endpoint in patients with untreated or incompletely treated AF.

This project is a collaborative effort sponsored by the National Heart, Lung, and Blood Institute and involving the following organizational units:

- Mayo Clinic, Rochester, MN, Douglas L. Packer MD, [Clinical/Admin Coordinating Center]
- Duke Clinical Research Institute (DCRI), Durham, NC, Kerry L. Lee PhD, [Statistics and Data Coordinating Center]
- Duke Clinical Research Institute (DCRI), Durham, NC, Daniel B. Mark MD, [Economics and Quality of Life Coordinating Center]
- Mayo Biomedical Imaging Resource Center, Rochester, MN, Richard Robb PhD, [CT/MR Image Analysis]
- University of Washington/Seattle Institute Cardiac Research (SICR), Seattle WA, Jeanne Poole MD, [ECG/EGM Core Lab]
- International CABANA Investigators involving approximately 180 enrolling centers.

CABANA Trial Organization and Process Flow

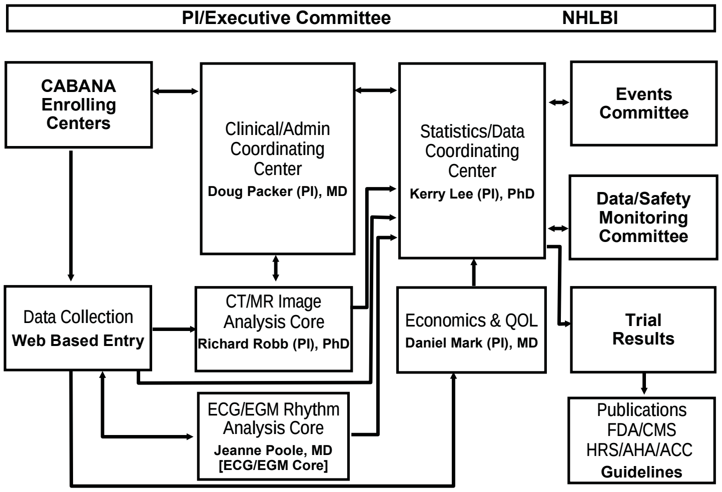

Figure 1

1.1 Background

Atrial Fibrillation: The Problem

1.1.1 Increasing Incidence and Prevalence

In the past 15 years, AF has emerged as the leading arrhythmic malady in terms of both numbers and sequelae. Several studies have documented a 1% incidence of AF in 60 year olds, with an increase to 8-12% in octogenarians [1]. This translates into a prevalence of 2.3 million patients, with a projected increase to 5.6 million by the year 2050 [1-3]. The prevalence of AF in the setting of heart failure is even higher, with up to 45% of patients with class IV heart failure suffering from this arrhythmia [4-6].

1.1.2 Progression of AF

While many patients with paroxysmal AF show little propensity for arrhythmia progression, the majority of studies have document a transition to more persistent AF in a substantial proportion of patients. The Canadian Registry of AF (CARAF) showed that 63% of 757 patients with paroxysmal AF had recurrent arrhythmia over a 5 year period, with progression to permanent AF seen in 25% [7]. Similar numbers have been documented by others [8, 9]. Although progression to chronic AF in patients with little underlying heart disease may be as low as 31% over 3 decades [10], other reports document progression to chronic AF in 48-77% of patients over 8-14 years[11,12]. These differences are due, in part, to the extent of underlying disease [13, 14].

There is also reason to anticipate an increasing impact of this arrhythmia on mortality, stroke, bleeding and cardiac arrest. In the Framingham study [15], both men and women between 55 and 75 years of age with AF showed a near doubling of mortality over the course of 10 years. Within that time frame, the overall death rate was approximately 15% in patients without and 55% in those with AF. This rate is further incremented in those individuals between the ages of 75 and 94 years, in whom the mortality rate increased from 30% to nearly 70% in the presence of AF [15]. Up to 50% of individuals with a new stroke in both population-based and cohort studies have underlying, potentially related, AF [16-18]. This risk increases from an attributable risk of 1.5% in the 6th decade of life to 23.5% in 80-89 year olds [18]. The problem of AF

associated stroke is corroborated by another population-based study from Olmsted County demonstrating an increasing prevalence of stroke related to AF over the past 3 decades, perhaps due to increasing AF occurrence [19].

1.1.3 Health Care Costs of AF

AF related illness is a rapidly increasing burden on the global health care system. Data from the 2003 National Discharge Survey in the US, used to estimate the annual number and prevalence of hospitalizations, showed an increase from 154,086 to 376,487 admissions for AF as the first listed diagnosis and from 787,750 to 2,283,673 for any AF diagnosis [20]. Other studies document an increase in hospitalizations and nursing home need because of this arrhythmia [20, 21]. A recent study suggested that the excess annual health care cost in the US is also 5 times greater in patients with, than without AF [22]. An additional four-fold increase in expense has also been reported in patients with multiple yearly AF recurrences [23]. Similarly, the cost of AF-related care in the UK rose from 244,000,000 Pounds in 1995 to 459,000,000 Pounds in 2000[24]. The cost of AF hospitalization, the foremost driver of AF-related expense, is likewise increasing [24-25], presaging even greater increases in health care costs in the future.

1.2 Clinical Experience with Drug Therapy

The medical approach to patients with AF by definition requires poly-pharmacy. Many patients with recurrent AF require rate control therapy. Others, in whom sinus rhythm is desirable, require treatment with an anti-arrhythmic drug, while patients with risk factors for stroke or peripheral thromboembolic events require anti-thrombotic therapy. Many health care providers also remain unenthusiastic about the limited efficacy and increased proarrhythmic risks of membrane-active agents for AF control.

1.2.1 Anti-Thrombotic Therapy

The importance of anti-thrombotic therapy in the management of patients with AF has been established by the AFASAK, SPAF, BAATAF, EAFT, CAFA and other trials demonstrating a reduction in stroke risk with warfarin [26]. The response to aspirin or clopidogrel and aspirin has been disappointing, as shown in these and the recent ACTIVE Trial [27]. Both the AFFIRM and RACE trials also clearly demonstrated excess stroke morbidity in patients discontinuing anticoagulation and in those with inappropriate INRs [28, 29]. This finding may be related to the occurrence of asymptomatic AF seen in 40-60% of patients [30-33]. Perhaps the most crucial contribution of these trials is the clarion call to maintain anticoagulation, even if sinus rhythm has been restored. It remains unclear whether these data apply to post-ablation patients.

1.2.2 Drug Therapy for Rate Control

In many patients with new onset AF, therapy with single or combination rate control agents can be valuable [34]. Agents such as beta- or calcium channel blockers, or even digitalis may control heart rate in the many patients treated aggressively. The AFFIRM trial demonstrated that 65% of patients so treated had appropriate rate control at 1 year, with 70% at 2 years, and 75% at 3 years [28]. Comparable findings were seen in the RACE trial [29]. Nevertheless, these patients may have a variety of other drug-related side effects.

1.2.3 Drug Therapy for Maintaining Sinus Rhythm

Multiple small observational studies demonstrate a 40-50% chance of maintaining sinus rhythm with membrane active anti-arrhythmic drugs over the course of one year. These data have been validated by recent larger comparative clinical trials. In AFFIRM, 82.4% of patients treated with rhythm control drugs were in sinus rhythm at the end of 1 year, 73.3% at 3 years, and 62.6% at 5 years [28]. This is in comparison with the 43% prevalence of sinus rhythm at 1 year,

which decremented to 34.6% at 5 years in those receiving AV Nodal blocking agents alone. Many of these patients in sinus rhythm at the time of follow-up had recurrent arrhythmia during the time between these evaluations. In the AFFIRM First Drug Study, 33% of patients taking amiodarone had an AF recurrence by 1 year, with 50% by 3 years. This was significantly better than the 52% AF recurrence in sotalol-treated patients at 1 year, and 71% recurrence at 3 years [35]. The findings were similar in RACE, with only 39% remaining in sinus rhythm at the end of the trial [29] and only 38% of patients at 2 years in the STAF Trial [36]. Overall improvements in quality of life (QO) were comparable in rate and rhythm control patients in AFFIRM [28, 37]. Rate control therapy was also more cost effective in these comparative studies [37, 38, 39, 40, 41]. In addition to an increase in morbidity and mortality risk with rhythm control therapy attributable to pro-arrhythmia, AFFIRM rhythm control patients also had excess non-cardiac mortality compared to their rate control counterparts.

1.2.4 Mortality Benefit of Drug Therapy in Randomized Trials

The morbidity and mortality response to drug therapy for AF seen in these studies has been disappointing [28, 29, 36, 38, 42]. In the AFFIRM Trial, conducted in patients at increased risk because of age or the presence of risk factors for stroke, there was no statistically significant difference in mortality outcomes with rate (21.3%) and rhythm (23.8%) control strategies at 5 yrs [hazard ratio 1.15 (0.99-1.34) for rhythm control therapy; p=0.08] [28]. Patients treated with rhythm control were more likely to require rehospitalization (80.1%) than rate control patients (73.0%) [p<0.001] and rhythm control therapy was more expensive [43]. The outcome of the secondary endpoint of death, disabling stroke or anoxic encephalopathy, major bleed, or cardiac arrest was also similar in the two arms. Importantly, similar trends of mortality and composite endpoints were seen in RACE [29], PIAF [38], and STAF [36]. As suggested by accompanying editorial opinion, only 40 to 50% of eligible patients were enrolled in these trials, in part because of bias toward rhythm control in highly symptomatic patients. These data may not be directly applicable to younger patients without heart disease.

The outcomes of rate vs. rhythm control management strategies were also recently examined in patients with underlying heart failure. The AF CHF Trial [42] enrolled 1376 patients with one AF episode in 6 months and either Class II-IV CHF and an EF ≤35%, or Class I CHF with a CHF hospitalization, or an EF ≤25%. 682 patients were randomized to rhythm control (Amiodarone in 82%) versus 694 to rate control (beta blockers in 88% and Digoxin in 75%) [42]. Thirty-one percent had paroxysmal while 69% had persistent AF, and 31% had Class III or IV CHF. There was no difference in the primary endpoint of CV death in rhythm (26.7%) vs. rate control patients (25.2%) [HR=1.06, CI=0.86-1.30, p= 0.59] over 37 months of follow-up. There was no difference in total mortality (31.8% vs. 32.9%, p=0.73), stroke risk (2.6% vs. 3.6%, p=0.32), worsening CHF (27.6% vs. 38.8%, p=0.17) or the composite of CV death, stroke, or worsening CHF (42.7% vs. 45.8%, p=0.20). Bradycardia was more common in those treated to maintain sinus rhythm (8.5% vs. 4.9%, p=0.007) and 21% of patients crossed over from rhythm to rate control therapy.

The findings of this trial, along with AFFIRM, RACE and STAF raise important questions regarding the quest for maintaining sinus rhythm in patients with AF. These studies can be interpreted as supporting the thesis that the restoration and maintenance of sinus rhythm does not alter over-all outcome. If true, this would indicate that 1) other factors are responsible for morbidity and mortality in AF patients, 2) AF is not a risk factor for either total or CV mortality, or 3) this arrhythmia is simply a marker of CV risk related to underlying disease. Alternatively, there may be significant benefit to sinus rhythm, which is offset in these trials by the pro-arrhythmia, heart failure, or organ toxicity attending anti-arrhythmic drug therapy. Disappointing efficacy of drugs in maintaining sinus rhythm would further diminish their appeal.

Any recommendation for rhythm control must be tempered by a consideration of pro-arrhythmic risk and occurrence of intolerable side effects. In AFFIRM, 5% of patients had a pro-arrhythmic event [44], with drug discontinuation because of side effects in 10% of rate control and 30% of rhythm control patients [28, 29]. In addition to poor efficacy, there also may be an inadequate AF gradient between groups of patients due to appreciable undetected AF and accompanying risk in rhythm control patients or unexpectedly common sinus rhythm in rate control patients, both limiting discernible differences between groups. Of note, 31% of patients in AFFIRM had paroxysmal AF and many of the rate control patients had sinus rhythm.

Despite the pessimism potentially generated by these issues, the results of the recent ATHENA trial have provided incentive for the reconsideration of drug therapy for AF [45]. In comparing the class III antiarrhythmic agent dronedarone in 2327 patients against placebo given to 2301 individuals, this study showed a 24% reduction in cardiovascular hospitalization or mortality (p,0.0001), a 29% decreased in cardiovascular mortality (p= 0.03), and a 26% decrease in cardiovascular hospitalization(p ≤0.0001) with active therapy at 22±5 months of follow-up. There were also significantly lower ACS and stroke rates with dronedarone therapy, in the setting of low proarrhythmia and heart failure rates. These data support the potential for cancelation of benefit from drug therapy by untoward toxicities of drug interventions.

1.3 Clinical Experience with Catheter Ablation

1.3.1 Single Center Ablation Success Rates

While these trials have established the benefit/risk ratios of pharmacologic rate and rhythm control therapies, in the absence of longer-term studies, the comparative long-term outcomes of ablative intervention remain unclear. This is particularly true in patients with advancing age or underlying disease. Still, the outcome of the AF CHF trial, viewed within the context of AFFIRM, RACE, and STAF, argues strongly for alternative catheter-based or surgical intervention for AF elimination. The efficacy and safety outcomes of catheter ablation for AF are available in the form of observational studies from individual centers [46-51]. These issues have been reviewed in detail in the recent AF Ablation Consensus Statement [50] and the Venice Chart International Consensus Document on AF Ablation [51]. These clinical studies document AF elimination rates of 55-80% with lasso-guided ablation of paroxysmal AF [46], with a 70-90% success rate using more aggressive wide area circumferential or high frequency, complex electrogram-guided ablative approaches [47,48]. The mechanism of beneficial effect is not completely established but likely includes the isolation of AF-initiating triggers arising within pulmonary veins (PVs), the alteration of parasympathetic and sympathetic nerve ganglia in the posterior LA, and the disruption of candidate initiation and substrate-mediated maintenance mechanisms of AF [47]. These studies have several limitations including absence of randomized enrollment, variability in techniques, differences in end points, the inconsistent use of blanking periods, and an uncertain number of redo procedures. Moreover, the 60-70% ablation success rate seen in the recent International AF Ablation Registry detailing outcome, suggests that the efficacy rate of ablation may be less than seen in earlier observational studies [49]. Furthermore, recent data have suggested an increased recurrence rate 2-3 years after ablation [52].

The complication rates of ablative intervention have been inadequately documented. While recent studies demonstrate a 1% stroke rate and a reduced PV stenosis rate down to 1-2% with ablative intervention, the same International AF Ablation Registry suggested that serious ablation events occur in up to 6% of patients [49]. The occurrence of stroke, PV stenosis, tamponade, and the recently reported lethal atrial-esophageal fistulae [53] indicate that the true complication rates of AF ablation have yet to be established.

Smaller, randomized ablation vs. drug studies include the CACAF [54], RAAFT [55], APAF [56], and A4 [57] Trials. These trials randomized 67-198 patients to rhythm control therapy vs. primary AF ablation. Over a 12-month follow-up period, a 76% average recurrence rate was seen in drug treated patients, which was greater than the 24% recurrence rate in ablated patients [54-57]. These studies also provide additional preliminary information. In the CACAF Trial [54], 1 of the ablation patients (1.5%) and one patient in the control group (1.5%) sustained a stroke, and 2 patients (3%) in the drug control group died over the course of follow-up. In the RAAFT Trial [55], re-hospitalization was required in 19 (54%) of anti-arrhythmic drug-treated patients compared with only 3 (9%) of those ablated ($p<0.001$). No mortality was seen in either treatment group. In these studies, 20-73% of drug treated patients crossed over to ablative therapy. Since patients over the age of 75 years were largely excluded, these early trial results may not apply to the increasing elderly population of patients at risk for untoward AF-related morbidity and mortality. Nevertheless, these data show similar rates of AF elimination, all favoring ablative intervention.

At least 4 studies have assessed the outcome of catheter ablation for AF occurring in the setting of ventricular dysfunction [58-61]. In contrast to the AF CHF Trial, these studies show a substantial improvement in EF/CHF class in patients in whom sinus rhythm was restored and maintained by intervention. Accompanying data suggest that this is more likely in patients with non-ischemic than ischemic cardiomyopathy. Short follow-up periods in these studies make it impossible to draw conclusions regarding mortality and/or morbidity benefit, however.

1.3.2 CABANA Pilot Study

Sixty patients were enrolled in 10 centers and randomized to AF ablation versus drug therapy, indicating the feasibility of randomizing patients with CABANA characteristics [62]. Of the patients enrolled, 77% were men and 23% women. The average age was 59 years (37% ≥65 yrs). Among the 60 patients, 80% had hypertension, 71% had 2 or more co-morbidities, 28% had sleep apnea and 25% had a family history of AF. Thirty two percent of patients had paroxysmal, 37% persistent, and 32% longstanding persistent AF. This pilot experience suggests that the Pivotal study will provide relevant information for a broad spectrum of AF patients. Only 7% of the pilot study patients had Canadian class 0 (asymptomatic) AF, 12% class I, 19% class II, 51% class III, and 11% had class IV symptoms [63]. Twelve percent had class I CHF symptoms, 31% were class II, and 5% had class III CHF. Twenty five percent had been on a single rhythm control drug, and 97% on rate control. Of these patients, 47% had required prior hospitalization for AF and 47% had been previously cardioverted. These patient characteristics are similar to AFFIRM patients and are very different from the typical catheter company PMA patients with paroxysmal AF and no other heart disease.

Twenty-nine patients (48%) were randomized to ablation and 28 of them underwent ablation. All 31 patients (52%) randomized to drug therapy were treated with rate or rhythm control agents. 13% of patients have been treated with rate-control only, 16% with rhythm control agents only, and both in 71%. The overall enrollment rate in this start-up pilot study exceeded the 0.7 patients/center/month needed to complete the pivotal study. In addition, following the change in focus from ablation expert-driven enrollment to non-ablationist co-PI recruitment, enrollment increased to 1.1 patients/site/month. The pilot study demonstrated the feasibility of recruiting and enrolling patients, identified the expected demographics of Pivotal Trial subjects, and has been very helpful in optimizing the study design of the Pivotal Trial.

1.4 Rationale For Maintaining Sinus Rhythm

None of the trials to date address the far-reaching question of the merit of maintaining sinus rhythm for reducing morbidity and mortality. Recent data from the AFFIRM trial provide a glimpse into this issue. In a post-hoc, time-dependent covariate assessment of the relationship between cardiac rhythm and survival, warfarin and sinus rhythm were associated with a lower risk of death [HR=0.53 CI=0.39-0.72 (p< 0.0001)] [64]. Similarly, 18 of 19 primary endpoint events in the STAF trial occurred in those patients remaining in AF [36]. Nearly identical findings were seen in patients in the DIAMOND studies, which reviewed the effect of dofetilide on total mortality in the setting of a prior MI or CHF [65]. The probability of survival in patients treated with dofetilide, who remained in sinus rhythm, was 60% at 36 months as compared to 30% in those remaining in AF, an effect also seen in the placebo group. Pappone et al [48] in his comparative report noted that 72% of all adverse events were observed in patients remaining in AF. In contrast, patients maintaining sinus rhythm in the RACE trial did not show improved survival [66]. It remains unknown whether the ability to maintain sinus rhythm is a marker of improved outcome, or whether sinus rhythm *per se* confers a mortality benefit. The post-hoc nature of the analyses limit the value of these results for guiding clinical practice.

The data from Nademanee and coworkers [67] also bear directly on the feasibility of the CABANA trial. Their study examined mortality and stroke outcomes in 540 patients with AFFIRM characteristics (age >65 or the presence of hypertension, diabetes, atrial enlargement or ventricular dysfunction in patients <65yrs). Their mean age was 69 years, with 165 patients ≥75 years of age, and 124 with an EF<40%. Fourty% had paroxysmal, 26% had persistent, and 34% had permanent AF. Each patient underwent ablation (once in 374, while 121 underwent 2 sessions, 43 underwent 3 sessions and 2 patients 4 sessions). 492 were followed for 4.2±1.1 years. The 2.7% long term mortality rate in 431 (86%) patients remaining in sinus rhythm was substantially better than the 16% death rate in the 63 (14%) who remained in AF (p<0.0001). Sinus rhythm was the most important independent predictor of survival (p=0.002), whereas EF and warfarin had little prognostic value in these patients. These studies taken together, along with data from population-based studies, provide the rationale for a large, multi-center trial to prospectively examine the impact of sinus rhythm on total mortality, disabling stroke, serious bleeding and cardiac arrest in patients with AF. Information from a well-designed randomized mortality trial is critically needed to provide a reasoned basis for the continued application of advanced ablation technology, justifying its concomitant cost, or its earlier application as first line therapy [68].

1.5 Rationale for Rhythm Monitoring

Most studies examining outcomes of drug and ablative therapy use symptomatic episodes of AF for establishing endpoints. While this allows assessment of AF burden and quality of life, it is an inadequate basis for making critical decisions regarding ongoing anticoagulation. Presumably, AFFIRM patients demonstrating excess morbidity after discontinuation of warfarin were felt to have complete control of their AF by anti-arrhythmic drug therapy [28]. Their subsequent events may have been due to undetected silent AF or some other underlying factor. Other studies have documented a moderate prevalence of asymptomatic or “silent” AF in other settings [30-33, 70-72]. Additionally, 10-40% of successfully ablated, asymptomatic patients may have silent AF [71, 72], which raises the possibility of continuing stroke risk or other morbidity, even though symptoms are eliminated [73]. Of note, others have reported far lower asymptomatic AF rates [48, 74, 75]. In addition, patients with asymptomatic AF may be at equivalent risk as their symptomatic counterparts. These issues, and the need to establish simple treatment efficacy, provide a clear rationale for careful monitoring to establish treatment efficacy.

1.6 Rationale for Cost and Quality of Life Investigations

AF is responsible for almost 500,000 in-patient hospitalizations in the US every year [20-21]. The direct medical costs associated with AF account for several billion dollars of excess health care spending per year in this country. At the individual level, AF increases medical spending from 9 to 22 times that for similar patients without AF. The cost effectiveness of ablation has been evaluated in preliminary studies only [76, 77]. Although case control studies have suggested that AF-related impairment of QoL is between that of having an MI and heart failure, improvement is expected with treatment [28, 29, 26, 38, 78-80]. Preliminary data suggest that AF ablation can restore QoL to levels comparable to age/sex matched general populations [81, 82]. Given the epidemic growth of this arrhythmia in the aging US population, more effective therapy offers the potential of relieving suffering in a growing number of citizens. As part of the CABANA research program, we plan to examine economic and QoL outcomes comprehensively using state-of-the-art tools and techniques used in recent or ongoing trials (OAT, STICH and SCD-HeFT).

1.7 Rationale for Cardiac Imaging Studies

Atrial remodeling, occurring with AF has been well documented, although it is difficult to know whether changes in atrial structure precede or follow this arrhythmia or are simply triggered by underlying disease. It also remains uncertain whether a reversal of atrial dysfunction occurs following ablation. In theory, elimination of AF should produce both electrical and anatomic remodeling, regardless of the therapy employed. At present, LA size and function outcomes following ablation are contradictory [83-88]. These data suggest an improvement in LA size and function following more limited PV isolation, although this might be offset by the scarring produced by more aggressive intervention. We also have preliminary data suggesting that LA dysfunction remains beyond the typical 1-2 month time frame used in deciding to stop anti-coagulation after restoring sinus rhythm [89]. CT / MR Image Analysis will be required to document the occurrence of LA remodeling following AF elimination with ablation or drug therapy and establish its impact on overall outcome, stroke risk, and the need for post-ablation anti-coagulation. The real-life prevalence of catastrophic atrial-esophageal fistula formation and PV stenosis will also be established. *While some of this desired information could be obtained echocardiographically, this approach is problematic given difficulties in standardizing image acquisition and interpretation. Furthermore, the echo approach cannot be used to assess pulmonary vein or esophageal status. CT or MR imaging can be standardized both in terms of acquisition and interpretation.*

1.8 Significance of the Trial

This trial is of substantial importance at multiple levels. Clinically, the trial will establish whether the emerging role of aggressive catheter ablation in the treatment of AF is justified by patient outcomes. This is an expensive approach, potentially complicated by life threatening events, which is now performed in thousands of patients without clear evidence of long-term benefit. The issues raised above have not been settled for “curative ablation”. The impact of age, AF type, and underlying disease on the outcome of ablation and drug therapy remain unclear. This study will answer these questions, will document the effect of ablation on AF recurrence and specifically examine health care costs, cost effectiveness, and quality of life outcomes. This study will also assess the role of earlier therapy for AF and the related utility of ablation as first line therapy in patients warranting treatment.

Scientifically, the trial will determine whether the attainment of normal sinus rhythm is a mortality and stroke advantage. No trial to date has prospectively addressed this issue, nor will any currently envisioned 1-2 yr study be sufficiently powered for this purpose. The trial should also

provide outcomes-based support for the role of AF as a modifiable risk factor of increased morbidity and mortality. The trial will also establish the determinants of ablation outcome. The CT/MR Imaging will also elucidate the structural abnormalities contributing to the occurrence of AF in a diverse population of patients and the modulation of those factors by drug or ablative intervention.

From a health care policy standpoint, this trial will help establish the place for medical and non-pharmacologic therapies for this escalating national healthcare dilemma. Aggressive intervention in an increasing number of patients and resulting mushrooming financial burden to society is already taking place at a time of increasingly constrained funding for health care. The Quality of Life and cost components of the trial will firmly establish whether AF ablation represents good value for the money and is an efficient way of improving health in the affected population relative to alternative health care expenditures. It will also allow much better estimation of the impact of the diffusion of ablation technology into the overall health care system. A critical window of opportunity for a randomized trial is now present, and the outcome of this landmark trial will likely shape therapy decisions and health care policy for years to come.

2.0 Objectives

Changes to the Protocol from the Original Study Design

Although CABANA was originally mandated to be a mortality trial, a careful assessment of the progress of the trial was undertaken by the study leadership in early 2013. Completely blinded to any treatment-specific outcome data, the two major issues addressed by the leadership group were (1) a lower than expected aggregated mortality rate, and (2) accrual of patients at a slower rate than projected. Careful consideration of these issues led to a decision to (a) change the primary endpoint of the trial from total mortality to the original key secondary endpoint consisting of the composite of death, disabling stroke, serious bleeding, or cardiac arrest, and (b) reduce the sample size to a number that was consistent with the new primary endpoint and more realistically achievable. There may still be a mortality difference between treatment groups. This was not revealed in the interim review. These changes will be highlighted in the sections of the protocol that follow.

2.1 Primary Objective and Hypothesis

The primary hypothesis of the CABANA trial is that the treatment strategy of percutaneous left atrial catheter ablation for the purpose of eliminating atrial fibrillation (AF) is superior to current state-of-the-art medical therapy with either rate control or rhythm control drugs for decreasing the incidence of the composite endpoint of total mortality, disabling stroke, serious bleeding, or cardiac arrest (primary endpoint) and reducing total mortality (key secondary endpoint) in patients with untreated or incompletely treated AF warranting therapy. The study is powered to detect treatment with percutaneous left atrial catheter ablation will reduce the incidence of this endpoint by $\geq$30% compared to drug therapy. To properly interpret this composite endpoint, the incidence of each of the individual components will also be descriptively examined to assess its relative contribution to the overall composite outcome. The primary endpoint and all secondary endpoints will be carefully assessed and analyzed on an intention to treat basis.

2.2 Secondary Endpoints/Objectives

1. Total mortality
2. Total mortality or cardiovascular hospitalization

3. Total mortality, stroke, or CV hospitalization (for heart failure or acute ischemic events)
4. Cardiovascular death
5. Cardiovascular death or disabling stroke
6. Arrhythmic death or cardiac arrest
7. Heart failure death
8. Freedom from recurrent AF
9. Cardiovascular hospitalization
10. Medical costs, resource utilization, and cost effectiveness
11. Quality of Life
12. Composite adverse events
13. LA size, morphology and function

2.2.1 Total Mortality

Because of the vital importance of assessing the impact of left atrial catheter ablation on total mortality, this endpoint (which is a component of the primary endpoint) will be a specific secondary endpoint in the trial.
Hypothesis: Catheter ablation for AF will reduce total mortality by ≥30% compared to state-of-the-art pharmacologic therapy.

2.2.2 Composite Mortality / Cardiovascular Hospitalization

By reducing the recurrence of AF, the proposed therapies should also reduce cardiovascular hospitalization. Additional secondary endpoints, including cardiovascular hospitalization and the composite of total mortality and cardiovascular hospitalization, will therefore be examined. The composite of total mortality, stroke or cardiovascular hospitalization will also be assessed. *Hypothesis*: Catheter ablation for AF will be significantly (≥25%) more effective than pharmacologic therapy, in reducing cardiovascular hospitalization, the composite of total mortality or cardiovascular hospitalization, and the composite of total mortality, stroke or cardiovascular hospitalization.

2.2.3 Freedom from Heart Failure Mortality

AF may be seen in 40-50% of heart failure patients. Furthermore, heart failure may occur because of uncontrolled ventricular response rates or as an adverse consequence of drug therapy. Heart failure related mortality may be affected differently by the ablative elimination of AF than by ongoing pharmacologic treatment.

2.2.4 Freedom from Recurrent Atrial Fibrillation

The purpose of therapy is to eliminate accompanying symptoms in affected individuals. An additional secondary endpoint will be the elimination of AF, comparing complete freedom from and time to recurrent AF outcomes of ablation and drug therapy. Time to second AF recurrence and AF burden will also be established. Freedom from AF after each ablation performed in an individual subject will be separately tracked.

2.2.5 Cost / Economic Impact

AF is responsible for almost 500,000 in-patient hospitalizations in the US every year and several billion dollars of excess health care expenditures. For patients, AF increases medical spending from 9-22 times that of comparable patients without AF, and this effect is likely to have even greater impact on health care spending in the future given the growth in number of AF patients expected and the increasingly complicated therapies used for these patients. As part of CABANA, we will examine the economic impact of AF ablation using state-of-the-art techniques.

2.2.6 Quality of Life

AF also causes significant impairment of quality of life, at a level between that of an MI and heart failure. Preliminary case control data further suggest that ablative therapy for AF can restore QOL to levels comparable to age and sex matched general populations. Given the epidemic growth of this arrhythmia in an aging population, a more effective therapy offers the potential to relieve suffering in a growing number of US citizens. Therefore, QOL assessment must be included in this trial.

2.2.7 Adverse Events

This trial must provide a clear delineation of the "down side" of therapy and track serious untoward events unique to each approach. Although strict comparisons will be difficult because of therapy-specific adverse events, additional analyses will look at each event type descriptively. With drug therapy, unanticipated heart failure hospitalization, pro-arrhythmic events, amiodarone pulmonary toxicity, and disabling stroke remain most concerning. For AF ablation the adverse events of greatest concern include pulmonary vein (PV) stenosis, atrial-esophageal fistula formation, and disabling stroke occurrence with ablative therapy. Of note, we will also track descriptively other complications and adverse events of both therapies and these will be recorded. We anticipate that the rate of serious adverse events will be similar in both treatment arms, although the specific complications will vary in type and extent.

2.2.8 LA Size and Function

The impact of long-term drug or ablative therapy on LA size and function is unknown [83-89]. Further, the relationship between structure and function measures and morbidity and mortality have not been examined. In addition to screening for PV stenosis, the CT / MR studies will examine these questions in both drug and ablation patients and provide mechanistic information above and beyond descriptive long-term outcomes data.

3.0 Subject Selection

The intent of the CABANA trial is to enroll patients who have new onset or under-treated paroxysmal, persistent, or longstanding persistent AF who ***warrant therapy*** for their arrhythmia. The vast majority of patients will be sufficiently symptomatic to cross the "therapy threshold" to active rate or rhythm control. Asymptomatic patients will be less common, but may be enrolled, if their AF is jeopardizing ventricular function, felt to be aggravating underlying disease, or requiring therapy to prevent sequelae of AF. Each patient will be screened for the inclusion/exclusion criteria listed below, which are similar to those of the AFFIRM trial [64].

3.1 Inclusion Criteria

To be eligible for the trial, subjects must meet all of the following criteria:

1. Over the preceding **6** months have:
   a) ≥2 paroxysmal (***electrocardiographic documentation*** of at least 1) AF episodes lasting ≥1 hour in duration: (that terminate spontaneously within 7 days or cardioversion is performed within 48h of AF onset): or
   b) ***electrocardiographic documentation*** of 1 persistent AF episode: (sustained for ≥7 days or cardioversion is performed more than 48h after AF onset): or
   c) ***electrocardiographic documentation*** of 1 longstanding persistent AF episode: (continuous AF of duration >1 year).
2. Warrant active therapy (within the past 3 months) beyond simple ongoing observation
3. Be eligible for catheter ablation and ≥2 sequential rhythm control and/or ≥2 rate control drugs.

4. Be ≥65 yrs of age, or <65 yrs with **one or more** of the following risk factors for stroke: Hypertension (treated and/or defined as a BP >140/90 mmHg) [90], Diabetes (treated and/or defined as a fasting glucose ≥126 mg/dl) [91], Congestive heart failure (including systolic or diastolic heart failure), Prior stroke, TIA or systemic emboli, Atherosclerotic vascular disease (previous MI, peripheral arterial disease or aortic plaque), LA size >5.0 cm (or volume index ≥40 cc/m^2), or EF ≤35. Subjects <65 yrs of age whose only risk factor is hypertension must have a second risk factor or LV hypertrophy to qualify.
5. Have the capacity to understand and sign an informed consent form.
6. Be ≥18 years of age.

NOTE: Subjects may have recent onset AF (in the past 4-6 months), AF present for a longer time period, or may have been treated with a single anti-arrhythmic drug, providing they remain realistically eligible for ≥2 membrane active drugs and/or ≥2 rate control agents. Patients receiving new drug therapy initiated within the previous 3 months may continue that therapy if randomized to the drug therapy arm. Reasonable expectation of a response to therapy must be present. Subjects will not be excluded because of advancing age or underlying heart disease. Subjects with a history of a single episode of paroxysmal AF do not meet the “crossing the threshold/warranting therapy” litmus test. Subjects with persistent or long-standing persistent AF will require at least 1 documented episode, if it is of sufficient clinical importance that drug or ablative therapy is warranted. Subjects can be randomized before cardioversion, even if restoration of sinus rhythm is a desired endpoint of therapy. Patients may have documented atrial flutter in addition to atrial fibrillation and remain eligible for enrollment.

3.2 Exclusion Criteria

If a subject has ***any*** of the following criteria, he or she may not be enrolled in the study:

1. Lone AF in the absence of risk factors for stroke in patients <65 years of age
2. Patients who in the opinion of the managing clinician should not yet receive any therapy for AF
3. Patients who have failed ≥2 *membrane active* anti-arrhythmic drugs at a therapeutic dose due to inefficacy or side effects (Table 5.2.2)
4. An efficacy failure of full dose amiodarone treatment ≥8 weeks duration at any time
5. Reversible causes of AF including thyroid disorders, acute alcohol intoxication, recent major surgical procedures, or trauma
6. Recent cardiac events including MI, PCI, or valve or bypass surgery in the preceding 3 months
7. Hypertrophic obstructive cardiomyopathy (outflow track)
8. Class IV angina or Class IV CHF (including past or planned heart transplantation)
9. Other arrhythmias mandating anti-arrhythmic drug therapy (i.e. VT, VF)
10. Heritable arrhythmias or increased risk for torsade de pointes with class I or III drugs
11. Prior LA catheter ablation with the intention of treating AF
12. Prior surgical interventions for AF such as the MAZE procedure
13. Prior AV nodal ablation
14. Patients with other arrhythmias requiring ablative therapy
15. Contraindication to appropriate anti-coagulation therapy
16. Renal failure requiring dialysis
17. Medical conditions limiting expected survival to <1 year
18. Women of childbearing potential (unless post-menopausal or surgically sterile)
19. Participation in any other clinical mortality trial (Participation in other non-mortality trials should be reviewed with the clinical trial management center)
20. Unable to give informed consent

NOTE: Exclusion Criterion #3 includes failed membrane active antiarrhythmic drugs started within 3 months prior to enrollment. Prior ablation of the cavo-tricuspid isthmus alone is not an exclusion if the patient develops subsequent recurrent AF. Planned atrial flutter ablation in combination with the left atrial ablation is not an exclusion.

4.0 Overview of Study

4.1 Trial Design and Time Line

This multi-center study will randomize 2000-2200 patients in a 1:1 fashion to a strategy of catheter ablation vs. state-of-the-art drug therapy with either rate or rhythm control, as outlined in Figure 2. Each patient will have untreated or incompletely treated AF, which in the opinion of the investigator warrants therapy. CABANA enrollment will occur over approximately 4 years.. All CABANA patients will be followed an average of approximately 5 years. Assuming criteria for early termination are not reached, the major trial results are expected to be reported in early 2018.

This study will be conducted in accordance with current United States Food and Drug Administration (FDA) Regulations and guidelines, the European Clinical Trials Directive and associated guidelines, International Conference on Harmonization guidelines on Good Clinical Practice, the principles of the Declaration of Helsinki, as well as all other applicable national and local laws and regulations.

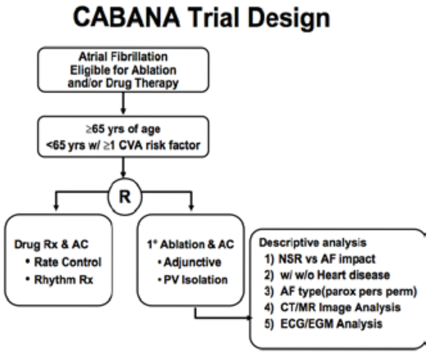

Figure 2

4.2 Screening and Pre-randomization Procedures

Study personnel will assess each subject against each inclusion and each exclusion criterion and the Investigator will determine the subject's eligibility for study participation. The principal investigator or documented members of the research team approved by local Institutional Review Board (IRB) or Ethics Committee (EC) will discuss the underlying rationale for the study, the procedures to be followed, the potential benefits and risks, and other issues mandated by the consent process. The informed consent process and all assessments will be documented in the subject's medical record or comparable source document. Baseline functional status, economic data (including two EQ-5D forms that rate the patient's health by using a 0-100 "thermometer" and asking 5 brief questions), and quality of life data (including a Baseline Questionnaire of validated scales, ie, SF-36, DASI, Toronto Atrial Fibrillation Severity Scale, AF Effects on QOL (AFEQT), Work Productivity and Activity Impairment Instrument (WPAI), Stanford Presenteeism scale and Mayo AF Symptom Index (MAFSI)) will be collected by the Site Coordinator via structured interview prior to randomization.

4.3 Enrollment and Randomization

Eligible patients, who have given written, informed consent and meet all inclusion with no exclusion criterion will undergo 1:1 randomization in an unblinded, parallel arm treatment format to a strategy of drug therapy for rate or rhythm control vs. catheter ablation. Randomization will be accomplished by telephone or internet using a centralized, interactive voice and web

randomization system (IXRS). The enrollment scheme is based on permuted block randomization with stratification by clinical site.

4.4 Post Randomization Procedures

For morbidity and mortality end points, intention-to-treat analysis will begin at randomization. All therapies will be established and optimized in a 3-month treatment initiation period, during which patients may receive alternative drugs or undergo repeat ablation if AF recurs. The duration of this “blanking period” is derived from standard clinical practice and the AF Ablation Consensus Document [50, 51]. Thereafter, therapy will be administered and patients followed for an average of approximately 5 years. Efficacy, (i.e. absence of AF recurrence) will be established by long-term follow-up beginning after the 3-month therapy initiation phase. Any AF occurring after the 3-month blanking period will be considered a treatment failure for the AF recurrence secondary endpoint. Patients will be followed at 6 and 12-month intervals from randomization throughout the trial.

4.4.1 Baseline Testing

Defining the eligibility of patients for the CABANA Trial will require information generated during the course of routine clinical care as dictated by their attending physician. This information should be, consistent with established guidelines, consensus documents, and good clinical practice. Selected baseline testing data will be collected in order to characterize the type, cause and severity of the patient’s AF and the treatments received prior to enrollment. The baseline data will include information from the following clinical evaluations:

1. Relevant medical history including prior and current drug treatment of AF
2. Relevant physical examination
3. 12 lead ECG prior to treatment
4. Blood Tests (INR, creatinine, hemoglobin / hematocrit) (pre-treatment)
5. Trans-thoracic 2-D echocardiography

The trans-thoracic echocardiographic (TTE) data will be used to characterize the substrate underlying the patient’s AF, establishing the presence of LV dysfunction (LVEF), hypertrophy, diastolic dysfunction, or other structural abnormalities. The TTE will also assess LA size and volume, and provide measures of atrial function.

Following a recommended approach consistent with established guidelines, consensus documents, and good clinical practice, a trans-esophageal echocardiographic (TEE) study will be performed within 24 hours prior to ablation in patients with *persistent or longstanding persistent* AF. The TEE may be performed up to 48 hours before the procedure in patients on continuous anticoagulation therapy, such as warfarin at a therapeutic INR or in those appropriately bridged with intravenous un-fractionated or low molecular weight heparin.

The performance of a pre-treatment TEE in patients with *paroxysmal* AF is left to the discretion of the investigator (5.4.4), but is not required in CABANA. Data on the performance and results of all TEEs performed as part of routine care will be collected in the eCRF, however. TEE data may also be used to confirm atrial size and morphology. The approach to cardioversion in drug treated patients should follow the recommendations of AF Treatment Guidelines [92].

In those centers where routine clinical practice includes the performance of pre-ablation and post-ablation CT/MR studies, relevant data from these studies will be collected to serve as a baseline for comparative quantitative LA size, morphology, and function studies, as well as

subsequent follow-up PV and esophageal investigations. In addition at those centers, up to 150 patients randomized to drug therapy will be asked to undergo one research CT/MR scan prior to initiating therapy. This will allow the CABANA study to evaluate and compare atrial structure and function in response to drug or ablative therapy. These drug-treated patients will be recruited from selected centers that are committed to CT/MR assessment of all enrolled patients (both study arms). Since randomization is performed (stratified) within each site, this component of the study will still benefit from the overall study randomization. Optimally, the pre-therapy scans performed in all patients should be within 4 months prior to treatment.

In addition to these clinically dictated studies, baseline economic and QOL data will be obtained after informed consent is obtained, but before randomization occurs.

4.4.2 Patient Follow Up

Follow-up in all patients will occur at 3, 6, and 12 months following randomization during the first year and every 6 months thereafter, with clinic visits, phone follow-up, and other testing as described below. Economic and QOL data, including a full follow-up questionnaire of validated scales, ie, SF-36, DASI, Toronto Atrial Fibrillation Severity Scale, AF Effects on QOL (AFEQT), Work Productivity and Activity Impairment Instrument (WPAI), Stanford Presenteeism scale, will be repeated by trained telephone interviewer staff from the EQOL Coordinating Center (EQOL CC) for patients enrolled in North America and by the Site Coordinator in sites outside North America.

Follow-up visits/calls at 3 and 6 months should be completed within 30 days +/- of the due date (Ex: 3 month visit: completed between 60 days and 120 days of randomization).

Follow-up visits/calls at 12 months and every 6 months thereafter should be completed within 60 days +/- of the due date (Ex: 12 month visit: completed between 300 days and 420 days of randomization).

Medical bills for patients enrolled at US sites will be collected throughout the trial by the EQOL Coordinating Center economic team. The Site Coordinators will complete a one page Rapid Report Form (RRF) at each CABANA study visit documenting any interim hospitalizations and/or ER visits since last contact. These forms will be forwarded to the EQOL Coordinating Center for processing. As part of the economic and QOL data in CABANA, two EQ-5D forms that rate the patient's health by using a 0-100 "thermometer", asking 5 brief questions and Mayo Atrial Fibrillation Symptom Index (MAFSI) will be collected by the Site Coordinator at 3 and 12 months following randomization during the first year and yearly thereafter throughout the trial and entered into the e-CRF.

After enrollment, subjects will either receive a single 'CABANA Box' recording system to be used throughout the entire study, or will be followed using ambulatory event and Holter ECG monitoring as generated during the course of routine clinical care as dictated by their attending physician.

At sites where the 'CABANA Box' has received appropriate approval, all patients enrolled will receive a single 'CABANA Box' recording system to be used for both patient activated event monitoring (throughout the trial), 24 hour autodetect/autocapture (AD:AC) event monitoring and 96 hour full disclosure Holter monitoring throughout the study. During year one, subjects will be asked to record their heart rhythm each month. After the first year, they will be asked for a 24 hour recording twice a year and a 96 hour recording twice a year. Fingertip recordings as well as AD:AC recordings can be transferred via telephone download from the patient's home to the

CABANA monitoring center. Holter recordings will require downloading of information at the enrolling site for data transfer to the CABANA monitoring center. All recordings will be made available to the enrolling center for use in clinical practice.

Follow up monitoring with an alternative system will be required in centers unable to use the "CABANA Box".

CT/MR imaging studies will be performed where clinically indicated or otherwise part of routine clinical care on ablation subjects after ablation therapy (between 90 days post ablation and the 6 month follow-up) and as indicated for PV stenosis management throughout the trial. The 150 drug therapy patients that received the CT/MR scan prior to initiating drug therapy, will also undergo one research CT/MR scan after therapy is fully established (between 90 days post drug treatment initiation and the 6 month follow-up). CT/MR data will be electronically transferred to a server at the Mayo Biomedical Imaging Resource, the CABANA Trial Image Analysis Lab. Scans will be anonymized at the site using a software tool provided by the Imaging Center. Each clinical center will also perform standard site radiology evaluation and assessment of the CT/MR images according to the sites' standard clinical practice.

After completion of the 60 month follow-up, subjects will be asked to extend their participation. If agreed upon, subjects will be asked about their current state of health and any clinical events every 6 months by telephone until the last subject enrolled reaches approximately 36 months follow-up.

Follow-up data will also be obtained at the time of treatment discontinuation, with a crossover in treatment strategy, and at the emergence of any primary or secondary endpoints.

4.4.3 Schedule of Assessment

Assessments	Baseline	Post Therapy	Mo 3	Mo 6	Mo 12	Mo 18	Mo 24	Mo 30	Mo 36	Mo 42	Mo 48	Mo 54	Mo 60	Extended every 6 months
			(+/-) 30 days		(+/-) 60 days									(+/-) 60 days
Medical history<br>Office visit	X		X		X		X		X		X		X	
Medical history<br>Office visit OR phone follow-up				X		X		X		X		X		X
Review Past/Current Medications	X	X(5)	X	X	X	X	X	X	X	X	X	X	X	X
Physical Exam	X			X		X		X		X		X		
Blood Tests<br>(INR, creatinine, hemoglobin, hematocrit)	X													
12 Lead Electrocardiogram (ECG)	X	X(5)												
Transthoracic 2-D Echocardiogram	X													
Trans-esophageal Echocardiogram (1) (2)	X													

Assessments	Baseline	Post Therapy	Mo 3	Mo 6	Mo 12	Mo 18	Mo 24	Mo 30	Mo 36	Mo 42	Mo 48	Mo 54	Mo 60	Extended every 6 months
			(+/-) 30 days		(+/-) 60 days									(+/-) 60 days
CT/MR Evaluation (1) (3)	X		X(4)											
CABANA Box/Ambulatory Monitor Symptom Event Recording		Throughout the 60 month follow-up periods when Atrial Fibrillation symptoms are present												
CABANA Box/Ambulatory Monitor 24-Hour Holter / AC;FD (monthly during Year 1)	X	Monthly / 1,2,3,4,5,7,8,9,10,11				Every 6 months / 15,21,27,33,39,45,51,57								
CABANA Box/Ambulatory Monitor 96-Hour Holter				X	X	X	X	X	X	X	X	X	X	
Economic and QOL Survey SF36, DASI,	X		X		X		X		X		X		X	X
Economic and QOL Survey Toronto AF Severity Scale AFEQT, WPAI, Stanford Presenteeism	X			X		X		X		X		X		X
AF Severity/Symptom Check List MAFSI, EQ5D	X		X		X		X		X		X			X
Review Adverse Events	X	X(5)	X	X	X	X	X	X	X	X	X	X	X	X

(1) Ablation therapy subjects;
(2) Or as warranted throughout trial;
(3) 150 subjects randomized to Drug Therapy;
(4) 3 months following therapy initiation = end of blanking period. Or as indicated for PV stenosis
(5) Hospital discharge or following Drug Therapy initiation

All procedures and laboratory tests during follow-up should be performed whether or not a subject receives treatment according to the protocol.

5.0 Treatment Arms

5.1 Description of Treatment Arms

5.1.1 Treatment Strategies

Rather than comparing a specific drug therapy against any specific ablative intervention, CABANA compares two treatment strategies. The first is that of medical therapy with the intention of controlling AF rhythm or rate. The second strategy is that of ablative intervention designed to eliminate AF. The following guidelines will shape therapy selection and implementation.

5.2 Pharmacologic Approach to AF (Drug Therapy)

It is recommended that drug treatment patients first receive rate control medication and the effect on ventricular response during the blanking period documented. Medication use must conform to the Guidelines for Management of Subjects with AF published in 2006 by the ACC/AHA/ESC [92]. Approved drugs and minimum dosing guidelines are as specified below.

5.2.1 Drugs Approved for Rate Control

Drug	Administration	Minimum recommended daily dosage
Digoxin (Lanoxin)	Oral	0.125-0.25 mg
**Beta Blockers**		
Metoprolol (Toprol)	Oral	50-100 mg
Atenolol (Tenormin)	Oral	50-100 mg
Propranolol (Inderal)	Oral	40-80 mg
Acebutolol (Sectral)	Oral	200-300 mg
Carvedilol (Coreg)	Oral	6.25-25 mg
Nebivolol (Bystolic)	Oral	5-40 mg
Bisoprolol (Monocor)	Oral	2.5-20 mg
**Ca Channel Blockers**		
Diltiazem (Cardizem)	Oral	180-240 mg
Verapamil (Calan)	Oral	180-240 mg

Patients without other heart disease receive beta or calcium channel blockers as first line rate control therapy [95]. Patients with coronary artery disease or heart failure will receive beta-blockers. Patients with ventricular hypertrophy not warranting exclusion would receive either beta- or calcium channel blockers, while patients with heart failure will receive carvedilol, bisoprolol, or metoprolol. Rate control medications may be used in combination and doses adjusted to achieve resting heart rates <80 bpm and exercise heart rates on 6 minute walk or Holter monitoring <110 bpm [28, 92]. These criteria will also be insured by clinical activity assessment in patients capable of exercise, as monitored by the patient's clinical cardiologist.

5.2.2 Drugs Approved for Maintenance of Sinus Rhythm

Drug	Administration	Minimum recommended daily dosage	Type/Level
Propafenone (Rhythmol)	Oral	450-625 mg	1C/B
Flecainide (Tambocor)	Oral	200-300 mg	1C/B
Sotalol (Betapace)	Oral	240-320 mg	Class III/ A
Dofetilide (Tikosyn)	Oral	500-1000 mcg	Class III/A
Amiodarone (Cordarone)	Oral	200-400 mg	Class III/B
Quinidine (Quinaglute/ dex)	Oral	600-900 mcg	Class I, III/B
Dronedarone (Multaq)	Oral	800 mg	Class III

At the documentation of failure of rate control using AFFIRM [28] and AF CHF [43] criteria, or if the patient has previously failed rate control by these criteria or remains symptomatic despite AV nodal blockers, rhythm control may be started using a membrane active drug in an approach consistent with the recommended Guidelines for Management of Subjects with AF [92]. Each patient will be placed on an anti-arrhythmic drug for an appropriate period and cardioverted to sinus rhythm if necessary. During the 3 month therapy initiation period, dosage adjustments can be made or the drug replaced with a different rhythm control drug. In general, patients with no other heart disease may be treated with propafenone, flecainide, sotalol or dofetilide. Patients with underlying ischemic heart disease or isolated diastolic dysfunction may/can be treated with sotalol, dofetilide, or amiodarone. Patients with underlying heart failure can be treated with dofetilide or amiodarone. Multaq (dronedarone) should not be used in patients with NYHA IV heart failure, or NYHA Class II-III heart failure with a recent decompensation requiring hospitalization or referral to a specialized heart failure clinic. Background therapy with upstream

ACE-I / ARBs, statins, fish oil, and aldosterone antagonists will also be encouraged and tracked at each follow-up.

5.2.3 Drugs for Future Consideration

It is anticipated that other rate control agents and membrane-active anti-arrhythmic drugs may become available over the course of the trial. Since a chief aim of the trial is to provide relevant, up-to-date information for guiding drug and ablative therapy for AF, these new treatment strategies will be incorporated in CABANA once approved by the CABANA Innovative Drug Therapy and Executive Committees. Drugs which have been approved by appropriate regulatory agencies outside of the United States may be used within that jurisdiction.

5.2.4 AV Nodal Ablation

Ablation of the AV conduction system may be considered as an alternate measure in patients with inadequate rate control refractory to at least 3 rate control agents or 2 membrane-active anti-arrhythmic drugs.

5.2.5 Changing Drug Therapy

During follow-up, patients may have recurrent AF, which constitutes an endpoint for the AF Recurrence secondary endpoint. Nevertheless, these patients will continue to be followed throughout the study to establish the occurrence of the primary and the secondary composite endpoints. These patients may require other drug therapy, which can be instituted during follow-up, without patient withdrawal from the trial.

5.2.6 Cross-Over to Ablative Therapy

During follow-up, drug arm patients with recurrences may be treated with additional anti-arrhythmic drugs or alternative rate control agents. Of note, most patients included to date in single center trials were treated for over one year before undergoing ablation. Thus, it is anticipated that most patients will be treated for at least 12 months in the drug therapy arm and that patients will be fully informed of this at the time of the consent process. Cross over ablative intervention is strongly discouraged. **Crossovers must be approved by the CABANA Trial Administrative Center. Before approval is granted for a patient randomized to drug therapy to be crossed over to ablation,** sites will be required to provide rationale and documentation that drug therapy options have been exhausted.

5.2.7 Compliance with Treatment Protocols /Site Performance

The success of the trial will be enhanced by careful consideration of 1) participating Site Qualification, 2) Procedure Standardization, 3) Site performance, and 4) Follow-up tracking. High standards in each of these areas will provide the greatest assurance that optimal, protocol-dictated therapy is being delivered and that patients have the best opportunity of responding to it. Each site will be required to provide documentation of expertise in the medical management of AF, including at least 2 of the following: 1) successful participation in AFFIRM, RACE, STAF, PIAF or other multi-center clinical trials; 2) medical management of at least 30 patients with AF; 3) site expertise in clinical practice, research, or quality assurance registries or databases; 4) an active AF clinic; or 5) treatment of >20 patients with sotalol, dofetilide or amiodarone. Patient compliance with medical therapy in rate control patients will be judged from achievement and maintenance of rate control targets noted in Section 5.2. Rates of sinus rhythm among rhythm control patients in the medical arm will be monitored and benchmarked against recent clinical trials including AFFIRM, AF CHF and ATHENA. Frequent phone contacts and newsletters will encourage compliance during the therapy initiation phase. These communications will then be continued regularly during the follow-up phase. Since we desire participation by a true cross section of individuals with AF, enrollment will not be based on availability of health insurance.

The IDE: G050233 approval establishes the approach of this trial as a CMS B2 therapy. As has been seen in the Pilot Study, the therapies in this trial can be billed to Medicare/3rd party payers.

5.3 Primary Catheter Ablation for AF (Ablation Therapy)

5.3.1 Pre-ablation Assessment

Pre-ablation assessment using standard practice patterns as outlined in the AF Ablation Consensus and Venice Chart Documents [50,51], will provide baseline data for safety and efficacy outcomes analyses. All patients who are randomized to ablative intervention will also undergo baseline CT or MR scanning. These studies will form the basis for comparisons of change in LA function with ablative or drug therapy.

5.3.2 Ablation Therapies

A potential limitation of any trial examining ablative intervention for AF is the number of different methodologies used for the primary curative ablation. The NHLBI Work Group on AF Ablation, the AF Ablation Consensus [50] and the Venice Chart [50] documents all agreed that pulmonary vein (PV) isolation is the starting point for AF ablation. PV isolation is therefore the minimum required ablation procedure for every patient in the trial. This may be a variation of: 1) Circular mapping catheter-guided ablation as described by Haissaguerre et al. [50, 93, 94], 2) Antral Isolation using a circular-guided approach as described by Natale et al. [95, 96], or 3) Wide area circumferential ablation [48, 97, 98]. As described in the AF Ablation Consensus Documents [50, 51], the approach in most expert ablation centers has evolved toward larger circumferential ablation outside the PVs. The selection of ablation guidance systems (circular mapping catheters, fluoroscopy, mapping systems and intracardiac ultrasound) is left to the discretion of the site PI, providing that site has performed at least 100 AF ablation cases with that approach. The acute endpoint of ablation is the isolation of all PVs.

Adjunctive procedures such as enlarging the field of ablation for the targeting of complex fractionated atrial electrograms [47], sites of apparent ganglia [99], or the use of additional linear lesions is allowed, but only after wider area or antral PV isolation. The methods for each of these are as outlined in the AF Ablation Consensus Document [50]. Each ablative approach and adjunctive measures will be carefully tracked to establish overall contribution to outcomes. Additional factors such as 1) termination of AF with ablation, 2) non-inducibility with atrial stimulation, and 3) absence of immediately recurrent AF with 10-20 mcg/min of isoproterenol, although controversial, will be tracked. Of note: ablation of the AV conduction system is not considered an alternative for primary ablative intervention.

5.3.3 Approved Ablation Devices

The specific choice of ablation catheters is left to the investigator, but must be one or more of the catheters listed below (see also Appendix B). Catheters which have been approved by appropriate regulatory agencies outside of the United States may be used within that jurisdiction.

Manufacturer	Catheter	Reference#
St. Jude Medical	Livewire TC™ XLS™	P960016
St. Jude Medical	Therapy™ Dual / Thermocouple	P040014
St. Jude Medical	Therapy Cool Path	P060019
Biosense Webster	NAVI-STAR / NAVI-STAR DS	P990025 / P010068
Biosense Webster	Celsius Braided Tip / Long Reach	P950005
Biosense Webster	NAVI-STAR Thermo-Cool	P030031
CryoCath	Freezor® / FreezorMax	P020045

Manufacturer	Catheter	Reference#
Bard	Stinger	P000020
Boston Scientific	Blazer II RF / RPM / SteeroCath /XP	P920047 / P020025
Boston Scientific	Chilli Cooled	P980003

5.3.4 Approaches for Future Consideration
Undoubtedly, primary ablative intervention for AF will evolve over the course of this trial. In order to maximize the potential for the generalizability of CABANA findings to the broader area of AF ablation, newly evolving methods or devices will be permitted, as approved by the Innovative Ablation Therapies / Executive Committees.

5.3.5 Cross-over to Drug Therapy
Patients with recurrent AF during the blanking period can be maintained on anti-arrhythmic drug therapy, although this should be discontinued by the end of the 90-day monitoring period. Subsequent AF recurrence will be considered an endpoint for the AF freedom endpoint. Those who are highly symptomatic may subsequently receive anti-arrhythmic drug therapy, but will be followed on an intention-to-treat basis as part of the ablation treatment group for primary and secondary endpoint assessments.

5.3.6 Compliance with Treatment Protocols /Site Performance
CABANA ablation sites must be pre-qualified for participation by 1) demonstration that each site has performed ≥100 primary AF ablations and 2) satisfactory completion of review of performance-confirming site data by the CABANA Ablation Therapies Committee including: 1) 5 prior ablation reports; 2) demonstration of an adequate prior ablation database to establish the CABANA patient's position in the site's overall series; 3) evidence of acceptable standard follow-up practices and data collection methods; 4) 5 pre and post lasso electrograms establishing acute PV isolation success; and 5) 5 prior Carto/NavX maps showing mapping proficiency. Taken together, these will provide validation of AF ablation expertise.

Site ablation performance will be tracked throughout the trial. Ablation data including ongoing acute success rates in achieving PV isolation, ablation time, fluoroscopy time, and ablation methods captured by ablation eCRFs will be tabulated by the Data Coordinating center. Outliers will be identified for special review. Exact ablation methods, complications, and compliance with the protocol will be reviewed by the DCRI monitors and a member of the Ablation Therapy Committee. Timeliness and accuracy of eCRF data entry will also be monitored.

5.4 Guidelines for Anti-thrombotic Therapy
5.4.1 Guidelines for Anticoagulation in Drug Treated Patients
Patients with risk factors for CVA or peripheral thromboembolic events at the time of enrollment, treated with rate control agents alone, will remain on active anticoagulation therapy (warfarin, dabigatran, rivaroxiban or apixaban) throughout the duration of the trial [92]. Unlike the AFFIRM trial, patients receiving rhythm control therapy will also be required to receive adequate anticoagulation (warfarin, dabigatran, rivaroxiban or apixaban) for the duration of the trial. In the use of warfarin therapy, target INRs of 2 to 3 will be required, unless higher INRs are mandated because of underlying disease.

5.4.2 Guidelines for Anticoagulation Therapy in Ablation Patients
Anticoagulation before, during, and after the ablative intervention will follow the guidelines of the AF Ablation Consensus Document [50]. Prior to the ablative intervention, patients with persistent and long-standing persistent AF should receive adequate anticoagulation (i.e. at least

one month of warfarin anticoagulation (INRs: 2-3)), or have a TEE excluding intra-atrial thrombus at the time of the intervention. During the ablative intervention, maintaining an ACT between 300 and 400 seconds is strongly recommended. Following the ablative intervention, patients will be started on IV heparin or subcutaneous injections of low molecular weight heparin beginning 4 to 6 hours after all sheaths are removed, and appropriate anticoagulation reinstituted the evening of the intervention. Thereafter, low molecular weight heparin is to be maintained until anticoagulated appropriately or standard dose warfarin achieves a target INR of 2 to 3, unless the ablation was performed at a therapeutic INR in patients maintained on warfarin through the ablation. One month following the ablation, dabigatran, rivaroxiban or apixaban may be substituted for warfarin, following recently written guidelines. Three to six months after ablation, warfarin may be replaced by full dose aspirin in patients with a CHADs score ≤1. This would include patients with hypertension without hypertrophy, or those <65 years of age, providing 1) atrial size and function are normal and 2) there is no symptomatic or asymptomatic AF by standard or full-disclosure monitoring. In those patients with a CHADs score ≥2, adequate anticoagulation is to be continued throughout the trial. Randomization of warfarin discontinuation is precluded by the low post-ablation stroke rates that would require at least 10,000 patients for detecting differences in stroke prevalence. Nevertheless, the characteristics, follow-up monitoring results, and long-term anticoagulation status of ablation patients will be compared to descriptively identify predictors of events and profiles of patients at high risk for warfarin discontinuation. The utility of trans-telephonic, auto-detection / full disclosure, and Holter monitoring, as a future aid in the decision to discontinue anticoagulation will also be critically examined.

5.4.3 Newer Antithrombotic Therapies

It is likely during the course of the trial that newer antithrombotic therapies non-inferior to warfarin or dabigatran will be approved. These agents may be used as replacement therapy for warfarin on approval of local regulatory agencies and the Innovative Antithrombotic Therapies/Executive Committees.

5.4.4 Items Left to the Discretion of the Physician

Specific items left to the investigators discretion include: 1) specific choice of rate control vs. rhythm control drug therapy and specific drugs to be used; 2) hospitalization to initiate anti-arrhythmic drug therapy; 3) choice of TEE guided direct current cardioversion (DCCV) vs. DCCV after 4 weeks of appropriate anticoagulation therapy, such as warfarin to an INR of 2-3; 4) pre-ablation TEE assessment in patients with simple paroxysmal AF and hypertension without hypertrophy; 5) continuation of warfarin to maintain a therapeutic INR at the time of catheter ablation; 6) selection of warfarin versus dabigatran, rivaroxiban or apixaban.

5.5 Management of Arrhythmia Recurrences During Follow-Up

During the 3 months of “therapy initiation”, patients randomized to drug therapy may receive alternative drugs. Patients in the ablation arm may undergo repeat ablation in those cases where the recurrent arrhythmia is not felt to be due to the irritation and inflammation of the ablative intervention. These patients may also be treated with rhythm control drug therapy, providing it is discontinued by the end of the blanking or “therapy initiation phase”. Any event occurring during this period will be tracked as an “Early Event”. Long-term follow-up with respect to efficacy will begin 3 months after the initiation of drug therapy or ablation therapy. These events will be used for the determination of overall freedom from recurrent AF, but will not be of sufficient importance to remove the patient from the trial. Patients achieving a secondary endpoint through non-fatal events will be followed throughout the remaining follow-up period.

6.0 Definitions & Adjudication for Study Endpoints

6.1 Primary Endpoints

The primary endpoint is the composite of total mortality, disabling stroke, serious bleeding or cardiac arrest. All components will be adjudicated by an independent Clinical Events Committee (CEC) and the most proximate cause of the event established. A Disabling Stroke will be considered present using a modification of a Rankin Stroke score [100], and will be adjudicated by a Neurologic Events Committee. Serious (or Life-threatening) bleeding will be considered present using a modification of the GUSTO bleeding Scale adapted for use in catheter ablation [101]. These events will be tracked regardless of treatment randomization. The definition for each of these events is listed in the CEC Charter. When there is disagreement between the CEC and the principal investigator, the CEC's decision will be considered final. Procedures for adjudicating events are described in the CEC Charter, which is available upon request. The CEC will also confirm whether a death is cardiac/vascular/non-cardiovascular in origin; as well as witnessed/un-witnessed; or sudden/non-sudden. Cardiac mortality will be further categorized as tachyarrhythmic, bradyarrhythmic, heart failure, or due to other cardiac causes using the events adjudication form. Hospitalization will also be tracked with specific reason for admission (heart failure, acute ischemic event, etc.) determined by the site principal investigator as reported in the eCRF.

6.2 Secondary Endpoints & Safety Endpoints

Secondary endpoint events, including total mortality and a composite of total mortality or cardiovascular hospitalization will be confirmed.

While hospitalization for any atrial fibrillation, atrial flutter or atrial tachycardia in both treatment arms will be carefully tracked and compared, it will be considered as an "*AF recurrence*" and counted against efficacy, not safety. Therefore, *AF recurrence and/or worsening of AF* ***need not*** *be reported as an adverse event.*

7.0 Adverse Events

Adverse events, defined as any undesirable medical occurrence in a clinical study patient which occurs during the course of the study and can be attributed to a device, procedure, or medications required *by a procedure, protocol, or trial* will be tracked throughout the study.

An adverse event (AE) will be considered present if 1) there are untoward signs, symptoms, illnesses, or other medical events that develop or worsen in severity during the course of the study, 2) they are clinically relevant *and* if they are clinically related to the study. Note: Disease, signs symptoms, and or laboratory abnormalities already existing at randomization are not considered adverse events unless they represent an intensity or frequency exacerbation. An adverse event designation should reflect the reason for a diagnosis or abnormal measurement. Procedures planned prior to randomization and the conditions leading to these measures are not adverse events.

An event inherent to an intervention/medication that is expected to occur for a projected duration in some or all subjects is considered unavoidable. Unavoidable adverse events will not be tracked during the trial (*examples: pain at catheter insertion sites during and after a procedure; pain at injection sites; palpitations; nausea due to anesthesia, etc*.).

The DCRI will evaluate any safety information that is spontaneously reported in the time frame specified in the protocol. For each subject, adverse events occurring after randomization must be recorded on the applicable Adverse Events page(s) in the electronic Case Report Form (eCRF). Recording should be done in a concise manner using standard, acceptable medical terms. The adverse event recorded should not be a procedure or a clinical measurement (i.e., a laboratory value or vital sign) but should reflect the reason for the procedure or the diagnosis based on the abnormal measurement. It is the responsibility of the Principal Investigator to oversee the safety of the patients enrolled in the study at his/her site. This responsibility includes careful assessment and appropriate reporting of adverse events. The primary mechanism for reporting adverse events in CABANA is for study personnel at the clinical sites to enter the relevant information and the details and description of each event using the adverse event forms that are part of the InForm electronic data capture (EDC) system being used in the trial.

Again, *AF recurrence and/or worsening of AF* ***need not*** *be reported as an adverse event.*

7.1 Adverse Event Classification

The investigator will evaluate all AEs with respect to seriousness, severity (intensity), and causality (relationship to study therapy or device) according to the following definitions and guidelines:

7.1.1 Anticipated Adverse Events

An anticipated event is one that has been identified in previous studies, published literature, or product labeling to be related to the disease state or therapies. A listing of commonly occurring ‘anticipated/expected’ events in the population being studied can be found in Appendix A. An unanticipated/unexpected adverse event is any adverse event that has not been reported in previous studies, published literature, product labeling, or which is not anticipated in the population being studied (see appendix A).

7.1.2 Serious Adverse Event

An adverse event that warrants additional action, includes *any* untoward event that:

1. Is fatal
2. Is life-threatening
3. Requires inpatient hospitalization or prolongation of existing hospitalization >48 hours. Exceptions- therefore not reportable:
    a. Preplanned (prior to the study) hospital admissions unless the hospitalization is prolonged >48 hours beyond the anticipated length of stay.
    b. Planned admissions (as part of a study – e.g. admission for drug change or titration for treatment of atrial fibrillation)
    c. 23 hour hospitalizations OR observation.
    d. Hospitalization for elective procedure (e.g. cardioversion).
    e. Emergency room visits that do not result in hospitalization.
    f. Hospitalization to titrate or optimize medical therapy to treat recurrence of atrial fibrillation.
4. Results in persistent or significant disability or incapacity.
5. Results in a congenital anomaly or birth defect.
6. Important medical events that may not result in death, be life-threatening, or require inpatient hospitalization may be considered an SAE when, based on appropriate medical judgment, they may jeopardize the patient and may require medical or surgical intervention to prevent one of the outcomes listed above.

7.1.3 Adverse Event Intensity or Severity Classification

The intensity or severity of each AE will be classified according to the following guidelines:

1. **Mild**: Any event that results in minimal transient impairment of a body function and does not threaten damage to a body structure, and/or does not require intervention other than monitoring (easily tolerated).
2. **Moderate**: Any event which results in moderate transient impairment of a body function or damage to a body structure, or which requires intervention.
3. **Severe**: Any event which is life threatening, results or could result in significant permanent impairment of a body function or damage to a body structure, requires significant and timely intervention to prevent permanent impairment of a body function or damage to a body structure, or which is intolerable or places the subject at immediate risk of harm.

7.1.4 Causal Relationship to Medical or Ablative Therapy

The International Council of Harmonization (ICH) Guidelines (1995) indicate that "reasonable causal relationship" means that "there are facts [evidence] or arguments to suggest a causal relationship." The causality assessment must be made by the investigator based on information available at the time that the adverse event eCRF is completed. The initial causality assessment may be revised as new information becomes available.

1. **Definitely related**: there is a reasonable temporal relationship to study therapy or device
   a. follows a known response pattern (e.g., study drug, treatment or device is known to cause this AE)
   b. there is no alternative etiology or explanation for the event
2. **Probably related**: there is a reasonable temporal relationship which
   a. follows a suspected response pattern
   b. no evidence for a more likely alternative etiology though could be unrelated
3. **Possibly related:** there is a reasonable temporal relationship but
   a. equivocal evidence that the event is study related as opposed to an alternative etiology
4. **Probably not related:** there is not a reasonable temporal relationship OR
   a. good evidence for a more likely alternative etiology
5. **Not related:** there is not a temporal relationship OR
   a. clear and compelling evidence that the event is due to an alternative etiology

7.1.5 Device Related Adverse Events

1. **Device-related:** any adverse event for which a causal relationship between the device and the event is a reasonable possibility. The likelihood that the event is device related will also be sub-classified using the approach in 7.1.4 above. The event will be further classified as whether it is a device failure or malfunction (7.1.5.2) and whether it is unanticipated (7.1.5.3), as described in these sections.
2. **Device Failure or Malfunction:** a device has failed if it is used in accordance with the Instructions for Use, but does not perform or function according to Instructions for Use and negatively impacts the treatment by preventing treatment as intended. Such a failure does not necessarily result in significant adverse outcome.
3. **Unanticipated Adverse Device Effect (UADE):** any serious adverse effect on health or safety or any life-threatening problem or death caused by, or associated with a device, if that effect, problem, or death was not previously identified in nature, severity, or degree of incidence in the investigational plan or application (including a supplementary plan or application) or prior medical literature, or any other unanticipated serious problem

associated with a device that relates to the rights, safety, or welfare of subjects. This event needs to be entered into the eCRF SAE pages within 24 hours of the site's knowledge.

7.2 Adverse Event Reporting

The goal is to have an adverse event reporting process that is (a) clear and simple for site investigators and study coordinators to understand and implement, (b) satisfies all regulatory reporting requirements, (c) eliminates any duplication in data collection and reporting, and (d) has a balanced focus on both the drug and ablation arms of the trial.

All related adverse events, serious and non-serious, that occur between the time of randomization and the last study-related procedure/visit will be followed until resolution, stabilization, or to trial completion. Adverse events for subjects who discontinue study participation at any time during the study should be collected/reported through at least the time of discontinuation. In addition, the required Institutional reporting structure will be followed and/or as described in this protocol.

7.3 Serious Adverse Event Reporting

Regardless of causality, the investigator will record all serious adverse events occurring between randomization and the last study-related procedure/visit or completion of the trial into the electronic database within 24 hours of knowledge of the event. DCRI will report all unanticipated/unexpected study related serious adverse events to Mayo Clinic, NHLBI and the DSMB chair within 2 business days of receipt.

The investigator must update the eCRF SAE when important follow-up information (final diagnosis, outcome, results of specific investigations, etc.) becomes available after submission of the initial entry. Follow-up information should be entered within forty eight (48) hours of knowledge. All reportable events will be followed until resolution, stabilization, or to trial completion, whichever occurs first. In addition, the required Institutional reporting structure will be followed and/or as described in this protocol.

7.4 Expedited Event Reporting

Specified events as listed below that meet ***serious*** criteria (see section 7.1.2 of the protocol), are ***related*** **(**possibly/probably/definitely) to either study drug or the ablation device or procedure, and are ***unanticipated/unexpected*** if occurring between randomization through completion of follow-up (end of trial) require ***expedited*** reporting to the DCRI and in turn to the appropriate regulatory agencies.

7.4.1 Expedited events include:

1. Unexpected, SAE related to study drug
2. Unanticipated ablation procedure related events
3. Unanticipated Adverse Device Effect (UADEs)
4. Device failures or malfunctions
5. **Events of Interest (EOI)**: Drug or ablation therapy or ablation procedure related events (index and/or follow-up): The related events that resulted in death, and the following events if they are life-threatening or severe in nature; pro-arrhythmic events, myocardial perforation / tamponade requiring intervention, esophageal atrial fistula, and/or severe pulmonary vein stenosis that were life threatening or classified as severe in nature.

7.5 Adverse Event Documentation

All AEs and SAEs must be documented on the appropriate eCRF. The outcome of each AE and SAEs will be tracked through the major endpoints of the trial, if present. Otherwise these events will be tabulated and addressed descriptively, as gleaned from the eCRF.

Expedited Events must be entered on the appropriate eCRF pages or if the electronic database is unavailable for more than 24 hours, the event would be reported on an *Expedited Event Form* and faxed/emailed to DCRI Safety Surveillance within 24 hours of knowledge of the event. When available, the event must be entered into the electronic data base.

DCRI Safety Surveillance

Telephone: 1-919-668-8624

Toll Free: 1-866-668-7799

Email: Safetysurveillance@mc.duke.edu

Fax: 1-919-668-7138

Toll Free Fax: 1-866-668-7138

It is understood that complete information about the event may not be known at the time the initial report is submitted. The investigator must assess the relationship of the event to the study device or study therapy and should make every attempt to obtain as much information as possible concerning the event. Additional information pertaining to an expedited event should be submitted to DCRI Safety Surveillance as it becomes available. All reported events will be followed until resolution, stabilization, or until the trial completion whichever occurs first.

7.6 Reporting to Regulatory Authorities

7.6.1 Physician Reporting of Drug or Device Adverse Events

Physician reporting of drug or device-related unexpected/unanticipated serious adverse events (as mandated by regulatory authorities) using MedWatch or Council for International Organizations of Medical Sciences (CIOMS) forms, should continue independently of any CABANA reporting.

It is anticipated that pphysicians and/or the appropriate healthcare professional will complete this reporting by, 1) MedWatch- submit form 3500 for drug or device via the FDA's MedWatch Adverse Event Reporting program online at www.fda.gov/MedWatch/report.htm, by phone 1-00-FDA-1088, or by returning the postage-paid FDA form 3500 downloaded from ww.fda.gov/MedWatch/getforms.htm by mail to MedWatch, 5600 Fishers Lane, Rockville, MD 20852-9787 or fax 1-800-FDA-0178, 2) CIOMS- using http://www.cioms.ch/index.htm, postal address: c/- World Health Organization, Avenue Appia, 20 CH - 1211 Geneve, 27 Switzerland, telephone: +41 (0) 22 791 34 13 or fax: +41 (0) 22 791 42 86.

7.6.2 CABANA Reporting of Drug or Device Adverse Events

For CABANA trial purposes, adverse events will be reported through the eCRF submission process designed to facilitate notification to DCRI Safety Surveillance and/or their designee. DCRI Safety Surveillance will review and code all SAEs.

The Safety Surveillance Medical Monitor (a physician trained and experienced in safety reporting) will review Events of Interest, and unexpected/unanticipated SAEs related to ablation

therapy or CABANA approved rate or rhythm control drugs. The decision regarding ultimate classification will be made by **individuals within CABANA Leadership** with expertise in antiarrhythmia and ablation therapies and clinical trial experience.

DCRI Safety Surveillance will notify the NHLBI and Mayo within 1-2 business days of an unexpectedness/unanticipated event determined to be related to the drug, device, and/or therapy. The final report will be submitted to the Regulatory Authorities and all participating investigators per regulations. Investigators are responsible for reporting trial unexpectedness/unanticipated events to their reviewing IRB within 10 working days of first learning of the event.

DCRI Safety Surveillance will notify the NHLBI within 5 business days of all reported Events of Interest (as identified above).

7.6.3 Criteria for Withdrawal of Subjects from Study

No subjects will be removed from the study, and attempts will be made to collect follow-up information for all subjects except those who specifically withdraw consent for release of such information.

8.0 Statistical Considerations

8.1 Sample Size and Power Considerations

In initially planning the CABANA trial, several design factors and research objectives were considered in developing an appropriate sample size for the study. First, the number of patients was determined so there would be a sufficient number of endpoints to provide a high degree of confidence (power $\geq$ 90%) for testing the primary hypothesis. Second, important secondary endpoints were considered. Third, it was considered important for the overall sample to be large enough to permit a prudent examination of treatment effects in selected subgroups of patients where AF ablation might be particularly advantageous, or where the question of a treatment benefit from this invasive procedure is particularly relevant. Important pre-specified subgroups of interest in this study include those defined by age, gender, race, AF type (paroxysmal, persistent, or long-standing persistent), AF duration, heart failure class, presence/absence of underlying structural heart disease, presence/absence of hypertension, and ejection fraction. Finally, the sample size was determined to provide a reasonable level of confidence of detecting clinically important therapeutic effects even if current projections of event rates and treatment differences prove to be optimistic.

As described in Section 2.0, the study was originally designed with total mortality as the primary endpoint. However, in early 2013 a careful assessment of the progress of the trial was undertaken by the study leadership. Completely blinded to any treatment-specific outcome data, the two major issues addressed by the leadership group were (1) a lower than expected aggregated mortality rate, and (2) accrual of patients at a rate much slower than projected. Careful consideration of these issues led to a decision to (a) change the primary endpoint of the trial from total mortality to the key secondary endpoint consisting of the composite of death, disabling stroke, serious bleeding, or cardiac arrest, and (b) reduce the sample size to a number that was consistent with the new primary endpoint. The following paragraphs, which outlined the key considerations in determining the original sample size, are also relevant for the revised sample size.

A combination of data from recent AF trials that reflect patient outcomes when treated with drug therapy (rate control or rhythm control) provide useful information on the range of outcomes that would be expected among the patients enrolled in the drug arm of CABANA. These studies include AFFIRM, RACE, STAF, PIAF and AF CHF. Since the study population in CABANA will be most like patients enrolled in the AFFIRM trial, the most reliable estimates of mortality and other endpoint events applicable to the drug arm of CABANA can be obtained from the AFFIRM data. The five-year mortality in AFFIRM was 23.8% in patients assigned to receive rhythm control drugs and 21.3% in patients assigned to rate control drugs. The crude death rate based on a mean follow-up of 3.5 years was 16.4%. However, the Kaplan-Meier curves in the primary AFFIRM publication reflect mortality rates at 3 years of 13% in the rhythm control arm and 11% in the rate control arm. At 3.5 years, mortality was approximately 16% in the rhythm control arm and 14% in the rate control arm. Based on this information, the mortality rate in the drug-treated arm of CABANA was projected to be approximately 12% after 3 years of follow-up and 15% after 3.5 years of follow-up (3.5 years is the average duration of follow-up originally projected for CABANA). With the original secondary endpoint elevated to become the primary endpoint, the incidence of the new primary endpoint is expected to be higher than the mortality rates.

In addition to the control (drug) arm event rate, a key driver of the sample size is the magnitude of the treatment effect (i.e., the reduction in the primary endpoint expected in the ablation arm). A synthesis of information from multiple published sources [48, 58-61, 67] suggests that the event rate in ablation-treated patients has been relatively low (less than 3% per year), which translates to a projected 3-year event rate of 8-9 % or a 3.5 year rate of 10% or less in the ablation arm of the trial (i.e., a reduction by one-third compared to the drug arm).

Another important factor that must be considered in sample size calculations is the extent to which patients randomized to the drug arm may **cross over** to receive an ablation during the course of their follow-up (if the AF and its symptoms are not adequately controlled by drugs). Although there are strict guidelines regarding changes to a patient's assigned therapy, in certain instances a downstream change may be clinically indicated. All crossovers and reasons for them must be carefully documented. However, crossovers of drug-arm patients to receive ablation will have the impact of reducing the event rate of patients randomized to drug therapy (assuming ablation is effective), and diminishing the magnitude of the treatment effect. Allowance for these crossovers is essential in determining the appropriate sample size. It is possible that up to 25-30% of patients randomized to the drug arm will cross over to ablation at some point during follow-up. Although the length of follow-up in the CABANA Pilot study was only 1 year, the rate of crossover to ablation of patients randomized to drug therapy was only 9.7%.

In addition to drug-arm patients crossing over to ablation, there may also be patients randomized to the ablation arm who require drug therapy. The number of patients randomized to ablation, that following randomization decline the procedure and are treated with drugs, is expected to be small and inconsequential in the sample size calculations. However, there will be patients randomized to ablation that during the course of their follow-up after the ablation are treated with drugs, including in some cases membrane-active antiarrhythmic drugs. For purposes of sample size calculations, these patients are assumed to receive the full benefit of ablation in reducing clinical outcomes, although one might argue that antiarrhythmic drugs, with their possible proarrhythmic effects, might attenuate the clinical benefits of the ablation.

Since the primary treatment comparisons in this study will be based on time-to-event methodology using the log-rank test [102] or equivalently, the Cox proportional hazards model

[103], the approach used for calculating sample size requirements for CABANA was based on the method of Schoenfeld [104] developed for the proportional hazards model.

To provide an adequate number of patients in the trial that will be relatively robust under various assumptions regarding the control-arm event rates and the magnitude of the treatment benefit, 2,000-2,200 patients will be enrolled. With a minimum follow-up of 3 years (amounting to an average follow-up of approximately 5 years), 2,200 patients will provide 90% power for detecting a 30% reduction in the new primary endpoint, and 2,000 patients will provide 88% power, assuming a 3-year event rate in the drug arm of 12% and allowing for a 2% loss to follow-up. Thus, the study will have high power for detecting an important benefit if the control arm event rate is consistent with the rate expected based on previous studies. A sample size in this range will also provide acceptable power (86% with 2200 patients and 82% with 2000 patients) for detecting a 25% reduction with ablation in the primary endpoint if the 3-year drug arm event rate is 15%. Thus we will have good power for detecting a more conservative estimate of the benefit in the composite endpoint if the control arm event rate is higher, but still consistent with previous studies. A 25-30% reduction in primary events will be highly important from a clinical and public health standpoint, given the large population of patients in this country and throughout the world who suffer from AF.

This number of patients (2,000-2,200) will also provide adequate power for detecting a 25% reduction in other important secondary composite endpoints listed in section 2.2 such as the endpoints that involve cardiovascular hospitalization where the incidence is expected to be higher than for the primary endpoint.

8.2 Statistical Analysis

Statistical analyses will be performed by the Coordinating Center. All major treatment comparisons will be performed by intention to treat, and endpoints will be attributed to the treatment arm to which the patients were randomized, regardless of treatment crossover or post-randomization medical care. Statistical comparisons will be performed using two-sided significance tests.

8.2.1 Analysis of the Primary Endpoint

The log-rank test will be the primary analytic tool for comparing outcome differences between the two therapies. Kaplan-Meier estimates of cumulative event rates as a function of follow-up time will be calculated and displayed. Relative risks will be expressed as hazard ratios with 95% confidence intervals generated using the Cox proportional hazards model.

Supplementary analysis involving covariate adjustment will be performed with the Cox model. Such adjustment will be limited to a relatively small, prospectively defined set of patient characteristics that are known *a priori* to have a strong prognostic relationship with the primary endpoint. The covariate-adjusted analysis will serve as a prelude to supplementary analyses examining differential treatment effects. The covariates will include age, sex, race, heart failure class, presence/absence of structural heart disease, whether the patients' AF is paroxysmal, persistent or long-standing persistent, duration of AF, presence/absence of hypertension, and ejection fraction. Cox model analyses may also be performed using appropriate groupings of sites as a stratification factor.

If the data provide evidence of an overall difference in outcome between treatment groups, an assessment will be made of whether the therapeutic effect is similar for all patients, or whether it varies according to specific patient characteristics. This analysis will focus on whether the

relative therapeutic benefit differs according to patient age, sex, race, type of AF, duration of AF, heart failure class, presence/absence of structural heart disease, presence/absence of hypertension, and ejection fraction. These analyses will utilize the Cox model by testing for interactions between treatment and these specific baseline variables. In addition to the formal assessment of treatment interactions, treatment effects characterized by a hazard ratio (with 95% confidence interval) will be calculated and displayed for prospectively-defined subgroups of patients defined by the variables listed above. These descriptive hazard ratios will be carefully interpreted in conjunction with the formal interaction tests.

8.2.2 Analysis of Secondary Endpoints

Secondary endpoints, including total mortality and secondary endpoints 2 through 9 listed in Section 2.2, will all involve time-to-event analyses and thus will be analyzed similar to the primary endpoint using the log-rank test, Cox model, and Kaplan-Meier event-rate estimates.

8.2.3 Analysis of Morbidity (Adverse Events)

Statistical comparisons of major adverse events are challenging in this trial because of therapy-specific differences in the nature of the adverse events. For drug therapy, the major events include unanticipated hospitalizations for heart failure, drug-related proarrhythmic events, and pulmonary toxicity from amiodarone. For the ablation arm, the major events include myocardial perforation with tamponade requiring resuscitation, symptomatic pulmonary vein stenosis requiring intervention, and atrial-esophageal fistula formation. The frequency with which these events (and other serious adverse events) occur will be descriptively tabulated. Treatment comparisons with respect to these serious adverse events will of necessity be more informal rather than statistically rigorous (since different adverse events are expected in the two arms), bringing to bear clinical judgment as to the relative seriousness of these various adverse events. Adverse complications or adverse events of either therapy will be carefully tabulated and descriptively summarized, using statistical comparisons of the treatment groups where appropriate, and interpreting such comparisons in the context of treatment differences in the primary and major secondary clinical endpoints.

8.2.4 Analysis of Core Lab Measures of LA Size, Morphology, and Function

Patients enrolled at a subset of the clinical sites will undergo baseline and follow-up CT/MR imaging studies, and core lab measures of left atrial size, morphology, and function will be examined. Of interest will be the change in atrial size (volume) and function (i.e., atrial ejection fraction) from the baseline to the follow-up study within each treatment arm, and then importantly, a comparison of whether the change in size and function in the patients randomized to ablation therapy differs from the change in size and function of the patients randomized to drug therapy.

The distribution of these measures from the baseline and follow-up studies in each treatment arm will be descriptively characterized using univariate descriptive statistics and graphical tools such as box and whisker plots. The statistical significance of the changes in left atrial size and function from baseline to follow-up within each treatment arm will be assessed using a paired-sample test such as the paired-t test. A two-sample comparison of the two groups with respect to changes in size and function will be performed using general linear models, including the baseline value as a covariate, and assessing treatment group differences. This comparison will be performed for the changes in atrial size (left atrial volume) and also for atrial function (atrial ejection fraction).

In dealing with this type of data, the completeness of which is dependent on obtaining both the baseline and follow-up studies, there will inherently be missing data resulting from patients in

whom it is not possible to obtain both studies (because they die or fail to return for other reasons to enable the follow-up measurements to be made). Every effort will be made to minimize the amount of missing data. However, because some data are expected to be missing, several missing data strategies will be employed in order to assess the sensitivity of the conclusions to the approach used. The approaches that will be considered include (1) analyzing only patients with complete data; (2) assigning "worst case" scores to missing observations; (3) carry forward previously observed data to the observation period missed; and (4) use of likelihood-based methods to impute missing data. By synthesizing the results from these different but complementary approaches, it will be possible to provide a comprehensive assessment with respect to these key mechanistic data.

8.2.5 "On-Treatment" Analysis

Although the intention to treat analyses will serve as the standard for interpreting treatment differences in the key clinical outcomes and constitute the primary analyses in the trial, because a number of patients from the drug arm may cross over to receive ablation during the trial, we will supplement the intent to treat comparisons with "on-treatment" comparisons. The "on-treatment" analysis will involve a comparison of patients who received ablation (even if originally assigned to the drug arm) versus those who did not. Statistically, this will be accomplished using the Cox proportional hazards model with ablation included in the analysis as a time-dependent covariate. Thus, event-free follow-up time for a patient in the drug arm who later crosses over to ablation would be credited to the drug arm until the time of crossover, and at that point, the patient would be shifted to the ablation group. Because treatment assignment is no longer random, results can be biased by an association between the likelihood of treatment change and the risk of a clinical event (i.e., factors that make a patient more likely to cross over may also make them more likely to have an event). Thus such analyses should be cautiously interpreted. The "on-treatment" analyses will also include an assessment of freedom from AF in ablation patients after each ablation performed, including those undertaken after the end of the blanking period.

An analysis using a time-dependent covariate with the Cox model will also be conducted to examine the prognostic relationships of sinus rhythm with clinical outcomes. The analyses described in this section will be covariate adjusted for baseline prognostic factors that are related to the clinical outcomes of interest. These analyses will be strictly exploratory and supplementary to the primary intent to treat analyses.

8.2.6 Interim Analyses

For ethical reasons, an interim examination of key safety and endpoint data will be performed at regular intervals during the course of the trial. The primary objective of these analyses will be to evaluate the accumulating data for an unacceptably high frequency of negative clinical outcomes in any of the treatment arms. In addition, the interim monitoring will also involve a review of the control arm event rates, patient recruitment, compliance with the study protocol, status of data collection, and other factors which reflect the overall progress and integrity of the study. The results of the interim analyses and status reports will be carefully and confidentially reviewed by an NHLBI-appointed Data and Safety Monitoring Board (DSMB).

The DSMB will meet at approximately 6-month intervals to review the accumulating data. Prior to each meeting, the Statistical and Data Coordinating Center will conduct the desired statistical analyses and prepare a summary report that will be carefully reviewed by the DSMB. The extracted data files and analysis programs for each DSMB report will be archived and maintained at the Data Coordinating Center for the life of the study. Reports will be presented describing the progress of patient enrollment, the rates of compliance with therapy, and the

frequency of protocol violations. The Data Coordinating Center will also report on the number (status) of data forms completion, the number of outstanding queries, the number of queries completed, and the number of forms reviewed through on-site monitoring.

These interim safety and efficacy reports introduce well-recognized statistical problems related to the multiplicity of statistical tests performed on an accumulating set of data. To properly account for the repeated interim testing in CABANA, a group sequential method similar to that proposed by O'Brien and Fleming [105] as a guide in interpreting interim analyses will be used. This procedure requires large critical values early in the study, but relaxes (i.e., decreases) the critical value as the trial progresses. Because of the conservatism early in the trial, the critical value at the final analysis is near the "nominal" critical value. Hence the sample size requirements with this group sequential procedure remain essentially the same as the conventional fixed sample size estimate. The actual method for this interim monitoring that will be employed in CABANA is the “spending function” approach to group sequential testing developed by Lan and DeMets [106]. The Lan-DeMets approach only requires specification of the rate at which the Type I error (which in this trial will be α=0.05 for the primary endpoint) will be "spent". This procedure allows "spending" a portion of α at each interim analysis in such a way that at the end of the study, the total Type I error does not exceed 0.05. One such spending function generates boundaries that are nearly identical to the O'Brien-Fleming boundaries. It is this approach that will be used in CABANA, namely two-sided, symmetric O'Brien-Fleming type boundaries generated using the flexible Lan-DeMets approach to group sequential testing. Since the number of looks and the increments between looks need not be predetermined, it allows flexibility in the monitoring process for accommodating additional comparative examinations of the data in response to concerns of the DSMB that may arise during the course of the trial.

The analytic approach that will be used at the interim analyses for assessing treatment differences will be the time-to-event analysis methods described previously, except that interpretation of statistical significance associated with treatment comparisons will be guided using the group sequential monitoring boundaries outlined above.

Judgment concerning the continuation or termination of the study will involve not only the degree of statistical significance observed at the interim analysis, but also the likelihood of achieving significance should enrollment continue to the originally projected sample size. As an aid in this latter assessment, the Coordinating Center will supplement the group sequential analyses outlined above with calculations of conditional power, namely the conditional probability that the treatment comparison will be significant at the end of the trial at the α level used in the design, given the hypothesized treatment difference and the data observed to date. After approximately 50% of the total events have occurred, conditional power for the primary treatment comparison will be computed and provided to the DSMB as part of the interim study reports.

8.2.7 Multiple Comparisons

With the primary hypothesis and the various secondary endpoints that have been outlined, there is a multiplicity of analyses to be performed, which leads to an increased probability that at least one of the comparisons could be "significant" by chance. Although adjustments (e.g., based on the Bonferroni inequality) could be used to preserve the overall type I error level, to adjust for the effects of the repeated significance testing that will occur as part of the interim monitoring (discussed above), plus adjust for the multiplicity of secondary endpoints, would require that very small significance levels be used for every comparison. Although the overall level of significance for the assessment of the primary composite endpoint will be 0.05, to account in

part for the multiplicity of comparisons involving secondary endpoints, a conservative approach will be taken while interpreting those results, taking into account the degree of significance, and looking for consistency across endpoints. The actual p-value for each comparison will be reported to aid in the overall interpretation.

8.2.8 Health Economics Analyses

The health economic analyses for CABANA will consist of two major parts, an empirical intention to treat cost comparison and a cost effectiveness analysis. Primary statistical comparisons between the two treatment arms of empirical costs will be performed by intention-to-treat. The patients enrolled outside the United States will be excluded from the primary cost intention-to-treat analyses. Confidence limits around the observed cost differences will be constructed using bootstrap methods.

The cost-effectiveness analyses will estimate the incremental cost required to add an extra life year with the investigational ablation arm relative to control medical therapy. In secondary analyses, we will incorporate utility weights to estimate the incremental cost per quality adjusted life year gained with ablation, relative to medical therapy. These analyses will be conducted from a societal perspective and will use a lifetime time horizon so that the estimated incremental cost-effectiveness and cost-utility ratios can be compared with societal benchmarks. We will also calculate within-trial cost-effectiveness/cost-utility ratios, although these ratios are limited in their value due to their failure to account for long-term benefits and costs and the absence of comparative benchmarks. Costs will be adjusted for inflation and both costs and life expectancy will be discounted to present value at a 3% annual discount rate. Adjustments for censored data due to staggered entry will be made following the approach of Bang and Tsiatis [107]. Extensive sensitivity analyses will be performed.

8.2.9 Quality of Life (QOL) Analyses

For each of the QOL measures examined in this study, data analysis will proceed in two stages. First, we will provide simple descriptive and comparative analyses by intention-to-treat. Second, we will examine changes over time from baseline and identify the major determinants of those changes using regression analysis. Since there is currently no consensus in the statistical literature about the best way to deal with the multiple comparisons problem arising from testing each individual scale separately, we propose two complementary approaches. First, we will pre-specify functional status (Duke Activity Status Index), AF symptom burden, and patient utilities (EuroQOL-5-D) as the primary QOL comparisons of interest and assign all other comparisons to a secondary (exploratory) status. Second, we will use the average of post-baseline values in the primary analyses of these 3 endpoints to reduce the problem of comparisons at multiple time points. Statistical power estimates for this part of our analysis show that we should have in excess of 90% power to detect ¼ SD differences in our 3 principal QOL endpoints.

9.0 Data Management and Quality Control

9.1 Study Data Collection -- All Patients

The full study dataset will be collected for subjects who enter the randomized/ treatment phase of the study. The primary data collection system for CABANA is based on the electronic data capture (EDC) system, InForm™. All data collected at any point in the trial, except the economic and quality of life information and core lab data are entered into this system. Data from the CT/MR Imaging Lab, the ECG Core Lab, and the Medicomp Monitoring Core Lab are transferred electronically to the Data Coordinating Center on a regular basis.

9.2 Electronic Case Report Form (eCRF)

This study will use web-based electronic CRFs (e-CRFs) developed through a validated, Electronic Record, Electronic Signatures (ERES) compliant platform (21 CFR Part 11). Prior to initiation of the trial, each site will be contacted as to computer availability, hardware specifications, and internet connectivity, to evaluate the capacity of the site to use this type of data collection system. Data will be entered into the InForm™ eCRF by personnel at the clinical sites. The Investigator's site staff who will be entering data will receive training on the system, after which, each person will be issued a unique user identification and password. For security reasons, and in compliance with regulatory guidelines, it is imperative that only the person who owns the user identification and password access the system using their own unique access codes. Access codes are non-transferable. Site personnel who have not undergone training may not use the system and will not be issued user identification and password until appropriate training is completed.

During monitoring visits, the site will make their computer and/or high speed Internet access available to the CRA, so that he/she may verify the data entries with the source documentation. At the conclusion of the study, each enrolling site will be provided with a compact disc (CD) containing PDF files of both the individual subject's data and the audit trail (changes made to the database). This will be maintained at the site according to the requirements for records retention.

Components of the eCRF in CABANA include enrollment and demographics form; a form for recording relevant history, symptoms, physical exam, ECG data and other baseline presenting characteristics; forms to document the details of the drug therapy and ablation procedure; follow-up forms for use at the regular follow-up visits and tracking the patient’s clinical course over time; and event forms for recording the circumstances and details surrounding the occurrence of a death, stroke, cardiac arrest, or bleeding event as well as other adverse events. In addition to the eCRF, there are specialized additional forms for collecting various components of the quality of life and health status information. Detailed instructions for completing the various data forms, along with a detailed Manual of Operations will be provided to each of the clinical sites

9.3 Data Management and Quality

Any out-of-range values and missing or inconsistent key variables are flagged and addressed/answered at the site in real time during the data entry process. When a query is generated on a particular variable, a flag is set in a field in the database enabling the system to track the queries and produce reports of outstanding queries. Queries can also be generated from manual review of the data forms. These queries will be entered into the database and tracked in the same manner as the computer-generated queries. At regular intervals, all data will be transferred from InForm™ to SAS for statistical summarization, data description, and data analysis. Further cross-checking of the data is performed in SAS, and discrepant observations flagged and appropriately resolved through a data query system.

The Data Coordinating Center will perform internal database quality-control checks, and data audits throughout the course of the trial. All clinical site patient-related payments are prompted by completion of data forms with appropriate responses to all data elements.

9.4 Economic and Quality of Life (EQOL) Data

For the CABANA study the economic and quality of life studies will be fully integrated into the clinical trial, including their inclusion in the main trial Informed Consent Form. The EQOL data

will be quality controlled and entered into the study data base at the EQOL Coordinating Center. Measurements of utilities by the EQ-5D and 0-100 Thermometer (EuroQoL) will be obtained throughout the trial in the CABANA EDC system. Statistical analysis of the EQOL data by intention-to-treat will be performed by the EQOL Coordinating Center.

10.0 Investigator Responsibility/Performance

By signing this protocol, the clinical site investigators agree to be responsible for implementing and maintaining quality control and quality assurance systems to ensure that all work incidental to this protocol is conducted and data are generated, documented, and reported in compliance with the protocol, accepted standards of Good Clinical Practice, and all applicable federal, state, and local laws, rules and regulations relating to the conduct of the clinical study.

The Investigator will provide current copies of the study protocol to all Sub-Investigators or other site personnel responsible for study conduct. The investigator must ensure that all site staff involved in the conduct of the trial are familiar with and have appropriate knowledge of the protocol and all study-specific procedures.

The Investigator will provide DCRI and/or their designee with copies of all IRB or EC actions regarding the study.

11.0 Study Data Reporting and Processing

The principal investigator is required to sign the eCRF on the appropriate pages to verify that he/she has accepted responsibility for the recorded data. This review and sign-off may be delegated to a qualified physician appointed as a sub-investigator by the principal investigator. The transfer of duties to a sub-investigator will be recorded on the Delegation list (kept on file at the site).

11.1 Site Selection and Monitoring

To qualify as an investigative site, each group must be committed to enrollment and randomization without bias or inclination toward early cross-over from drug to ablative therapy. Each site must also demonstrate the skill and expertise for participating in clinical trials as judged from their past trial performance. A Co-PI approach to the enrollment and treatment of patients will be required. A cardiac electrophysiologist skilled in the art of AF ablation will work closely with a non-interventional clinical cardiologist or internist. This will also help counter potential bias in favor of ablation. The focus of recruitment will be in Primary and Secondary Care clinics, Hospital ERs, and first referrals to ablation centers. Each site will be strongly encouraged to minimize the time between randomization and ablation to <2 weeks, by "holding" ablation slots.

As part of a concerted effort to follow the study in a detailed and orderly manner in accordance with established principles of Good Clinical Practice and applicable regulations, the Monitoring Plan is being revised with Executive Committee approval. A DCRI study monitor or their designee will no longer perform on-site visits to Active study sites regularly and throughout the study. Rather they will maintain frequent telephone and written communication, as well as perform on-site visits to a subset of sites to ensure data integrity.

The on-site monitoring visits will be made to assure that the Investigator obligations are fulfilled and all applicable regulations and guidelines are being followed. These visits will assure that the

facilities are still acceptable; the protocol and investigational plan are being followed, the IRB/EC has been notified of approved protocol changes as required, complete records are being maintained, appropriate and timely reports have been made to DCRI and/or their designee and the IRB/EC, study drug and study drug inventory are controlled and the Investigator is carrying out all agreed activities.

During on-site monitoring visits, the Monitor will perform a review of all Inclusion/Exclusion criteria of selected randomized subjects. Review of informed consent forms, HIPAA Authorization, all events meeting criteria for expedited event reporting as well as selected SAE's and Events of Interest (EOI)'s will be performed. Additional review will be performed on a site-by-site basis, as warranted. Key variables (inclusion/exclusion criteria, adherence to treatment assignment and safety) on the eCRF will be compared with each subject's source documents. Any discrepancies will be noted and resolved.

During monitoring visits, the site will make their computer and/or high speed Internet access available to the CRA, so that he/she may verify the data entries with the source documentation.

11.1.1 Initiating / Training Sites

Over the first year of the trial, sites will come on-line for active participation once all local regulatory requirements have been met. Unconditional IRB/EC approval will be required at each site. Training for data collection and reporting must be completed. Individual sites will also be trained at the time of the first general investigator meeting held at the beginning of the trial. The specific site's ablation and clinical investigators and the clinical study coordinator will be required to attend these meetings.

11.1.2 Terminating a Site

Any activated site may be terminated from the trial for failure to enroll any subjects, within a reasonable period of time. Also, any site may be suspended from active enrollment, if it fails to meet reasonable enrollment goals or comply with study procedures. Specifically, any site consistently failing to provide timely reports or adequate data quality will be withdrawn from active enrollment. A cross-over rate ≥10% over any 6 month period will result in a warning to that site. A crossover rate >15% will prompt suspension of enrollment. A 3-month period for resolving any operational difficulties will be required prior to reinstating or permanently terminating a specific site to further enrollment. All subjects randomized must be followed until death or the end of the trial, even if the site has been inactivated due to enrollment or study compliance.

11.1.3 Conflicts of Interest: The two principal investigators from each site, along with additional study staff will be required to disclose any present or potential conflicts of interest following guidelines established by the American College of Cardiology.

12.0 Study Documentation

Study documentation includes all electronic data collection forms, source documents, monitoring logs and appointment schedules, sponsor-investigator correspondence and regulatory documents, etc.

The investigator will prepare and maintain complete and accurate study documentation in compliance with Good Clinical Practice standards and applicable federal, state, and local laws,

rules and regulations; and, for each subject participating in the study, promptly complete all original case report forms and such other reports as required by this protocol.

By signing the protocol, the investigator acknowledges that, within legal and regulatory restrictions and institutional and ethical considerations, study documentation will be promptly and fully disclosed and shall be made available at the investigator's site upon request for inspection, copying, review and audit at reasonable times by representatives of DCRI and/or their designee or responsible government agencies as required by law.

The investigator agrees to promptly take any reasonable steps that are requested by DCRI and/or their designee as a result of an audit to cure deficiencies in the study documentation and case report forms.

13.0 Source Documentation

Source documents include all recordings of observations or notations of clinical activities and all reports and records necessary for the evaluation and reconstruction of the clinical study.
Whenever possible, the original recording of an observation should be retained as the source document; however, a photocopy is acceptable provided that it is a clear, legible, and exact duplication of the original document.

Regulations require that Investigators maintain information in the study subject's medical records which corroborate data collected on the CRF (eCRF). In order to comply with these regulatory requirements, the following information will be maintained and made available as required by DCRI and/or their designee monitors and/or regulatory inspectors:

1. Medical history/physical condition of the study subject prior to involvement in the study sufficient to verify protocol entry criteria.
2. Medical record documenting that informed consent was obtained for the subject's participation in the study
3. Notes for each subject visit including results of examinations.
4. Lab results
5. Dated printouts or reports of special assessments, (e.g., ECG reports).
6. Description of adverse events and follow-up of the adverse events (minimally event description, severity, onset date, duration, relation to study drug or device, outcome and treatment for adverse event).
7. Notes regarding concomitant medications taken during the study (including start and stop dates).
8. Subject's condition upon completion of or withdrawal from the study.

14.0 Protocol Deviations

A protocol deviation is defined as an event where the Investigator or site personnel did not conduct the study according to the Investigational Plan or the Investigator Agreement.

Investigators are required to obtain prior approval from CABANA Administration before initiating deviations from the investigational plan or protocol, except where necessary to protect the life or physical well-being of a subject in an emergency. Such approval will be documented in writing and maintained in study files. Unless CABANA Administration has consented to any such deviations in writing, CABANA Administration will not assume any resulting responsibility or

liability. Prior approval is generally not expected in situations where unforeseen circumstances are beyond the Investigator's control, (e.g., subject did not attend scheduled follow-up visit, blood sample lost by laboratory, etc.); however, the event is still considered a deviation.

Deviations will be reported to DCRI and/or their designee regardless of whether medically justifiable, preapproved by CABANA Administration, or taken to protect the subject in an emergency. Subject specific deviations will be reported on the [study specific, e.g., Report of Non-Compliance] Form.

Investigators will maintain documentation of the dates and reasons for each deviation from the protocol, in compliance with the ICH-GCP guidelines, Code of Federal Regulations (CFR) 812.140 and national legislation.

15.0 Data Transmittal and Record Retention

Required data will be recorded on the appropriate eCRF at the time of or as soon as possible after the subject visit or the availability of test results.

As forms are completed or updated, data will be transmitted via the Internet from investigational sites to a central site utilizing state-of-the-art encryption mechanisms to ensure security and confidentiality.

The investigator takes responsibility for retaining adequate and accurate hard copy source documents of all observations and data generated during this study. Such documentation is subject to inspection by DCRI and/or their designee as well as the FDA and other regulatory agencies, as provided by law. DCRI and/or their designee will be contacted if the investigator plans to leave the institution so that arrangements can be made for transfer of responsibilities.

16.0 Study Closeout

The end of the trial is defined as the day of the official end of patient follow-up in the trial.

For clinical trial sites located in the EU, a declaration of the end of the clinical trial will be made according to the procedures outlined in Directive 2001/20/EC. For sites located in countries outside the EU, local regulations will be followed.

Upon completion of the study DCRI and/or their designee will notify the site of closeout and a study closeout visit will be performed. The DCRI monitor and/or their designee will ensure that the Investigator's regulatory files are up to date and complete, and that any outstanding issues from previous correspondences have been resolved. Other issues to be reviewed at the closeout visit include: discussing retention of study files, possibility of site audits, publication policy, and notifying the IRB of study closure.

17.0 Audit/Inspections

DCRI Quality Assurance personnel and/or their designee may conduct audits at the study site(s). Audits will include, but not be limited to: audit trail of data handling and processes, SOPs, drug supply, presence of required documents, the informed consent process, and

comparison of case report forms/database with source documents. The investigator agrees to accommodate and participate in audits conducted at a reasonable time in a reasonable manner, as needed.

Regulatory authorities worldwide may also audit the investigator during or after the study. The investigator should contact the Sponsor immediately if this occurs, and must fully cooperate with governmental (e.g. FDA) audits conducted at a reasonable time in a reasonable manner.

18.0 Informed Consent

The investigator has both ethical and legal responsibility to ensure that each subject being considered for inclusion in this study is given a full explanation of the study. Written informed consent will be obtained from all subjects before any study-related procedures are performed or given.

The principal investigator or IRB-documented members of the research team will approach the patient to obtain written informed consent on an informed consent form (ICF) approved by the same IRB/EC responsible for approval of this protocol. The informed consent document will conform to FDA regulations in 21 CFR Part 50, and/or to the national requirements for informed consent. It must include all elements required by law, local regulations, GCP and International Conference on Harmonization guidelines and study specific procedures. The underlying rationale for the study, the procedures to be followed, the potential benefits, risks, alternatives, and other issues mandated by the consent process will be fully disclosed. If new information becomes available during the course of the trial that may be relevant to the subject’s consent, the Informed Consent Form will be revised and the revised version will be submitted for EC/IRB approval before use.

The investigator agrees to obtain approval from DCRI and/or their designee of any ICF intended for use in the study, prior to submission for IRB approval.

19.0 Confidentiality of Subjects

Subject confidentiality will be maintained throughout the clinical study in a way that ensures the information can always be tracked back to the source data. For this purpose, a unique subject identification code (ID number and subject name code) will be used that allows identification of all data reported for each subject.

Subject information collected in this study and all records will be kept confidential and the subject’s name will not be released by study staff at any time.

When requested patient medical records may be examined by authorized monitors (DCRI and/or their designee) or Clinical Quality Assurance auditors appointed by the sponsor, by appropriate IRB / IEC members and by domestic and foreign regulatory authorities. In all cases, caution will be exercised to assure the data are treated confidentially and that the subject’s privacy is protected.

Furthermore; for clinical trial sites located in the US, the NHLBI has issued CABANA a Certificate of Confidentiality to protect the privacy of research subjects by withholding their identifiable information from all persons not connected with this research.

20.0 Authorization for Use and Disclosure of Protected Health Information (HIPAA)

For clinical trial sites located in the US, an Authorization for use and disclosure of protected health information (PHI) under the HIPAA Privacy Rule [45 CFR § 164.102 *et seq*] will be obtained from every trial subject prior to, or at the time of, enrollment. It will be presented to, and signed by, the subject at the same time as the Informed Consent Form (ICF). The investigator is responsible for obtaining subjects' authorizations and signatures, and for explaining the elements of the HIPAA Authorization form if necessary.

HIPAA Authorization may either be a separate form or included in the study ICF, dependent upon local requirements. If a separate HIPAA document is signed, the investigator will append one signed original of each executed HIPAA Authorization to the trial subject's signed ICF, and file it in the site's regulatory file. If a second copy of the signed ICF is filed in the subject's medical records, an additional copy of the signed HIPAA Authorization form will be appended. Subjects will be given the other signed duplicate for their personal records.
The HIPAA Authorization form will contain all elements required under the HIPAA Privacy Rule. By law, site IRB approval of the Sponsor-provided Authorization form for use in this study is not required, and no such approval will be sought or requested. However, DCRI or their designee, upon request, will provide advance copies of its HIPAA Authorization form to the investigator or the site's privacy board or privacy official, and will work with the site to eliminate any concerns.
The investigator or the site will promptly inform DCRI or their designee of any restrictions on the use or disclosure of PHI of any subject to which the site or the investigator have agreed under the Privacy Rule. The investigator or the site will also promptly inform DCRI or their designee of any written revocation of any subject's HIPAA Authorization.

21.0 Human Subject Protections

.

21.1 Research Subject Selection and Justification of Exclusions

There will be no exclusion from participation in the study on the basis of ethnicity or race. Subjects will undergo a screening process, during which they will have multiple opportunities to ask questions. The Principal Investigator or authorized designee will provide a detailed discussion of the protocol, and answer any questions. The subject will be given time to consider study participation. No coercion or undue influence on this decision will be used. Only those subjects who give written, informed consent and complete enrollment testing will be considered for participation in the study.

22.0 Institutional Review Board/Ethics Committee Review

This study will be initiated only after all required documentation has been reviewed and approved by the respective IRB/EC and competent authority (CA) according to national and international regulations.
The investigator will provide DCRI or their designee with the study approval documentation before the study may begin. The same is applicable for the implementation of changes introduced by amendments.
Where applicable, the investigator must also provide to DCRI and/or their designee the following documentation:

1. A copy of IRB annual re-approval of the protocol per current Title 21 CFR 312.66 regulations and 1997 International Conference on Harmonization guidelines.

2. IRB approval of revisions to the informed consent documents. Administrative changes (such as a change in address or phone number) must be sent to IRBs/Ethics Committees but do not require their approval.
3. The investigator must submit periodic status reports to their EC as required, as well as notification of completion of the study and a final report where applicable.
4. The investigator will provide DCRI or their designee with documentation of all approvals.

23.0 Publication Policies

Trial results will be released in several manuscripts providing outcomes of the trial as a whole. No details will be released prior to the scheduled presentation of the main trial results. Data summaries will be provided to the DSMB and NHLBI as appropriate for trial regulation. Release of other results, including those generated in any sub-study must be done in a manner so as to protect the integrity of the trial as a whole. Portions of the database will not be released to single or groups of enrolling centers for analysis.

The Publication Committee will receive and review applications and potential authors for sub-studies beyond those comprising main trial results, and make recommendations to the Executive Committee for approval. A detailed list of the sub-studies, the requesting investigators, and the timing of applications will be maintained in anticipation of other publications.

All manuscripts, abstracts and presentations will be reviewed by the Publication Committee for scientific merit, appropriateness of the presentation or manuscript, and for authorship. All presentations or publications of <u>any</u> type are expected to maintain the integrity of the main objectives of the overall project. By agreement of the principal investigators, endpoint data will not be presented prior to the release of main study results. Authorship on ancillary studies will depend on contribution to the trial effort.

24.0 Sub-Studies

All activated <u>sites will be given the opportunity to participate or not participate</u>. All proposed sub-studies will be first submitted for review and approval to the respective IRB/EC and competent authority (CA) based on national and international regulations. It is further acknowledged that <u>subjects enrolled in CABANA will have the choice to participate or to not participate</u> in sub-studies.

24.1 CABANAgene

<u>CABANAgene</u>: a resource that will accumulate DNA samples from CABANA subjects to enable subsequent genotype-phenotype studies.

Appendix C, page 62, the CABANAgene protocol, provides the rationale for studies and approaches that are anticipated. The major goal of CABANAgene is to create the resource. Specific projects to use the samples would require approval of the CABANA study group, and phenotypes to be studied would be those collected by and adjudicated by the CABANA study group. The CABANAgene project is included in the Pharmacogenetics Research Network (PGRN) arrhythmia site renewal. Participation under the PGRN umbrella also provides access to advanced genotyping and genetic statistical methods to investigators accessing the CABANAgene resource.

Examples of issues that CABANAgene could address include: 1) predictors of response to antiarrhythmic or rate control drug therapies; 2) predictors of response to warfarin therapy; 3) predictors of response to ablation therapy and; 4) clinical and genetic approaches to defining AF subtypes.

25.0 References

1. Go AS, Hylek EM, Phillips KA, Chang YC, Henault LE, Selby JV, Singer DE. Prevalence of diagnosed AF in Adults. National implications for rhythm management and stroke prevention: the Anticoagulation and Risk Factors In AF (ATRIA). JAMA 2001; 285:2370-75.
2. Lloyd-Jones DM, Wang TJ, Leip EP, Larson MG, Levy D, Vasan RS, D'Agostino RBD, Massaro JM, Beiser A, Wolf PA, Benjamin EJ. Lifetime risk for development of AF. The Framingham Heart study. Circulation 2004; 110:1042-46.
3. Greenlee R, Vidaillet H. Recent progress in the epidemiology of atrial fibrillation. *Current Opinion in Cardiology.* 2005;20(1):7-14.
4. Maisel WH, Stevenson LW. AF in heart failure: Epidemiology, pathophysiology, and rationale for therapy. Am J Cardiol 2003; 91:2D-8D.
5. Wang TJ, Larson MG, Levy D, Vasan RS, Leip EP, Wolf PA, D'Agostino RB, Murabito JM, Kannel WB, Benjamin EJ. Temporal relations of AF and congestive heart failure and their joint influence on mortality: The Framingham Heart Study. Circulation 2003; 107:2920-2925.
6. Cha YM, Redfield MM, Shen WK, Gersh BJ. AF and ventricular dysfunction: a vicious electromechanical cycle. Circulation 2004; 109:2839-2843.
7. Kerr CR. Humphries KH. Talajic M. Klein GJ. Connolly SJ. Green M. Boone J. Sheldon R. Dorian P. Newman D. Progression to chronic atrial fibrillation after the initial diagnosis of paroxysmal atrial fibrillation: results from the Canadian Registry of Atrial Fibrillation. Am Heart J. 149(3):489-96, 2005.
8. Levy S, Maarek M, Coumel P et al. Characterization of different subsets of AF in general practice in France: the ALFA Study. The College of French Cardiologists. Circulation 1999; 99:3028-35.
9. Takahashi N, Seki A, Imataka K, Fujii J. Clinical features of paroxysmal AF. An observation in 94 patients. Jpn Heart J 1981; 22:143-149.
10. Jahangir A, Lee V, Friedman PA, Trusty JM, Hodge DO, Kopecky SL, Packer DL, Hammill SC, Shen WK, Gersh BJ. Long-term progression and outcomes with aging in patients with lone atrial fibrillation: a 30-year follow up study. *Circulation.* 2007; 115(24):3050-66.
11. Kato T, Yamashita T, Sagara K, Linuma H, Fu LT. Progressive nature of paroxysmal atrial fibrillation. Observations from a 14-year follow-up study. *Circulation.* 2004; 68(6):568–72.
12. Hirayma Y, Atarashi H, Kobayashi Y, Horie T, Iwasaki Y, Maruyama M, Miyauchi Y, Ohara T, Yashima M, Takano T. Angiotensin-converting enzyme inhibitor therapy inhibits the progression from paroxysmal atrial fibrillation to chronic atrial fibrillation. *Circulation.* 2005; 69(6):671-676.
13. Prakash R, Green M, Kerr C, Connolly S, Klein G, Sheldon R, Talajic M, Dorian P, Humphries K. The association of left atrial size and occurrence of atrial fibrillation: a prospective cohort study from the Canadian Registry of Atrial Fibrillation. *Am Heart J.* 2004; 148(4):649-654.
14. Saksena S, Hettrick D, Koehler J, Grammatico A, Padeletti L. Progression of paroxysmal atrial fibrillation to persistent atrial fibrillation in patients with bradyarrhythmias. *Am Heart J.* 2007; 154(5):884–892.
15. Benjamin EJ, Wolf PA, D'Agostino RB, Silbershatz H, Kannel WB, Levy D. Impact of AF on the risk of death: the Framingham Heart Study. Circulation 1998; 98:946-952.
16. Wolf PA, Abbott RD, Kannel WB. AF: A major contributor to stroke in the elderly. The Framingham Study. Arch Intern Med. 1987; 147:1561-1564.
17. Wolf PA, Dawber TR, Thomas HE, Kannel WB. Epidemiologic assessment of chronic AF and risk of stroke. The Framingham Study. Neurology 1978; 28:973-977.

18. Wolf PA, Abbott, RD, Kannel WB. AF as an independent risk factor for stroke: The Framingham Study. Stroke 1991; 22:983-988.
19. Tsang TS, Petty GW, Barnes ME, O'Fallon WM, Bailey KR, Wiebers DO, Sicks JD, Christianson TJ, Seward JB, Gersh BJ. The prevalence of AF in incident stroke cases and matched population controls in Rochester, Minnesota: changes over three decades. J Am Coll Cardiol 2003; 42:93-100.
20. Wattigney WA, Mensah GA, Croft JB. Increasing trends in hospitalization for AF in the United States, 1985 through 1999: Implications for primary prevention. Circulation 2003; 108:711-16.
21. Friberg J, Buch P, Scharling H, Gadsboll N, and Jensen GB. Rising rates of hospital admissions for AF. Epidemiology 2003; 14:666–72.
22. Wu E, Birnba H, Mareva M, Tuttle E, Castor A, Jackman W, Ruskin J. Economic burden and co-morbidities of atrial fibrillation in a privately insured population. *Current Medical Research & Opinion.* 2005; 21(10):1693-1699.
23. Reynolds MR, Essebag V, Zimetbaum P, Cohen DJ. Healthcare resource utilization and costs associated with recurrent episodes of atrial fibrillation: the FRACTAL registry. *J Cardiovasc Electrophysiol*. 2007; 18(6):628-33.
24. Steward S, Murphy N, Walker A, McGuire A, McMurray JJV. Cost of an emerging epidemic: an economic analysis of AF in the UK. Heart 2004; 90:286-92.
25. Le Heuzey J, Paziaud O, Piot O, Said M, Copie X, Lavergne T, Guize L. Cost of care distribution in atrial fibrillation patients: The COCAF study. *Am Heart J.* 2004; 147(1):121-126.
26. Hart RG, Benavente O, McBride R, Pearce LA. Antithrombotic therapy to prevent stroke in patients with AF: a meta-analysis. Ann Intern Med 1999; 131:492-501.
27. ACTIVE Writing Group on behalf of the ACTIVE Investigators; Connolly S, Pogue J, Hart R, Pfeffer M, Hohnloser S, Chrolavicius S, Pfeffer M, Yusuf S. Clopidogrel plus aspirin versus oral anticoagulation for atrial fibrillation in the Atrial fibrillation Clopidogrel Trial with irbesartan for prevention of Vascular Events (ACTIVE W): a randomised controlled trial. Lancet. 2006; Jun 10; 367:1903-12.
28. The AF Follow-up Investigation of Rhythm Management (AFFIRM) Investigators. A comparison of rate control and rhythm control in patients with AF. NEJM 2002; 347(23):1825–33.
29. Van Gelder EC, Hagens VE, Bosker HA, Kingma JH, Kamp O, Kingma T, Said SA, Darmanata MI, Timmermans AJM, Tijssen GHP and Crijns HJGM, for the Rate Control versus E lectrical Cardioversion for Persistent AF Study Group. A comparison of rate control and rhythm control in patients with recurrent persistent AF. NEJM 2002;347:1834–40.
30. Ellenbogen KA, Wood MA. Asymptomatic AF and pacemaker diagnostics: the unseen presence. Heart Rhythm 2005; 2:132-3.
31. Strickberger SA, Ip J, Saksena S, Curry K, Bahnson TD, Ziegler PD. Relationship between atrial tachyarrhythmias and symptoms. Heart Rhythm 2005; 2: 125-131.
32. Rodriguez LM, Timmermans C, Wellens HJ. Are electrophysiological changes induced by longer lasting AF reversible? Observations using the atrial defibrillator. Circulation 1999; 100:113-6.
33. Orlov MV, Ghali JK, Araghi-Niknam M, Shefesee L, Sahr D, Hettrick DA, for the Atrial High Rate Trial Investigators. Asymptomatic atrial fibrillation in pacemaker recipients: incidence, progress, and determinants based on the atrial high rate trial. *PACE*. 2007; 30(3):404–11.
34. Farsi R, Kistner D, Sarma JS, Longmate JA, Singh BN. Ventricular rate control in chronic AF during daily activity and programmed exercise: a crossover open-label study of five drug regimens. J Am Coll Cardiol. 1999; 33:304-10.

35. AFFIRM First Drug Study Investigators. Maintenance of sinus rhythm in patients with AF: an AFFIRM substudy of the first antiarrhythmic drug administered. J Am Coll Cardiol 2003; 42:20-29.
36. Carlsson J, Miketic S, Windeler J, Cuneo A, Haun S, Micus S, Walter S, Tebbe U, for the STAF investigators. Randomized trial of rate control versus rhythm control in persistent AF. The strategies of treatment of AF (STAF). J Am Coll of Cardiol. 2003; 41:1690–96.
37. Hagens VE, Ranchor AV, Van Sonderen E, Bosker HA, Kamp O, Tijssen JG, Kingma JH, Crijns HJ, Van Gelder IC, RACE Study Group. Effect of rate or rhythm control on quality of life in persistent AF. Results from the Rate Control Versus Electrical Cardioversion (RACE) Study. J Am Coll Cardiol 2004; 43:241-7.
38. Hohnloser SH, Kuck KH, Lilienthal J, for the PIAF investigators. Rhythm or rate control in AF – Pharmacological intervention in AF (PIAF): a randomized trial. Lancet 2000; 356:1789–94.
39. Thrall G, Lane D, Carroll D, Lip G. Quality of life in patients with atrial fibrillation: a systematic review. *Am J Medicine.* 2006; 119(5):448.e1-448.e19.
40. Fenwick E, Marshall D, Levy A, Nichol G. Using and interpreting cost-effectiveness acceptability curves: an example using data from a trial of management strategies for atrial fibrillation. *BMC Health Services Research.* 2006; 6:52.
41. Pietrasik A, Kosior DA, Niewada M, Opolski G, Latek M, Kaminski B. The cost comparison of rhythm and rate control strategies in persistent atrial fibrillation. *International J of Cardiol.* 2007; 118(1):21-7.
42. Roy D. AF/CHF Trial: Rate as good as rhythm control for AF in heart failure. *2007 American Heart Association Scientific Sessions.* 2007; Late-breaking clinical trial report.
43. Marshall DA, Levy AR, Vidaillet H, Fenwick E, Slee A, Blackhouse G, Green HL, Wyse DG, Nichol G, O'Brien BJ, and the AFFIRM and CORE Investigators. Cost-effectiveness of rhythm versus rate control in AF. Annals of Internal Medicine 2004; 141:653-61.
44. Kaufmann ES, Zimmermann PA, Wang T, Dennish GW, Barrell PD, Chandler ML, Greene HL, and the AFFIRM investigators. Risk of proarrhythmic events in the atrial fibrillation follow up investigation of rhythm management (AFFIRM) study. A multivariate analysis. Journal of the American College of Cardiology 2004; 44:1276–82.
45. Hohnloser SH, Crijns HJ, Van Eickels M, Gaudin C, Page RL, Torp-Pedersen C, Connolly SJ for the ATHENA Investigators. Efffect of Dronedarone on Cardiovascular Events in Atrial Fibrillation. N Engl J Med 2009; 360:668-78.
46. Packer DL. Progress in non-pharmacologic therapy of atrial fibrillation. J Cardiovasc Electrophysiol. 2003 Dec; 14(12 Suppl):S296-309.
47. Nademanee K, McKenzie J, Kosar E, et al. A new approach for catheter ablation of AF: mapping of the electrophysiologic substrate. J Am Coll Cardiol 2004; 43:2044-53.
48. Pappone C, Rosanio S, Augello G, Gallus G, Vicedomini G, Mazzone P, Gulletta S, Gugliotta F, Pappone A, Santinelli V, Tortoriello V, Sala Simone, Zanmgrillo A, Crescenzi G, Benussi S, Alfieri O. Mortality, morbidity, and quality of life after circumferential pulmonary vein ablation for AF. Outcomes from a controlled nonrandomized long-term study. J Am Coll Cardiol. 2003; 42:185-97.
49. Cappato R, Calkins H, Chen SA, Davies W, Lesaka Y, Kalman J, Kim YH, Klein G, Packer DL, Skanes A. Worldwide survey on the methods, efficacy, and safety of catheter ablation for human AF. Circulation 2005; 111:1100-1105.
50. Calkins H, Brugada J, Packer D, et al. HRS/EHRA/ECAS Expert consensus statement on catheter and surgical ablation of atrial fibrillation: Recommendations for personnel, policy, procedures and follow-up. *Heart Rhythm.* 2007; 4(6).
51. Natale A, Raviele A, Arentz T, et al. Venice chart international consensus document on atrial fibrillation ablation. *J Cardiovasc Electrophysiol.* 2007; 18:560-580.

52. Wokhlu A, Hodge DO, Monahan KH, Haroldson J, Asirvatham S, Friedman PA, Munger TM, Packer DL. Impact of presenting AF type of late recurrences at long-term follow up (>18 mo.) after ablation. *Heart Rhythm.* 2007; 4(suppl 5S):S64.
53. Pappone C, Oral H, Santinelli V, Vicedomini G, Lang CC, Manguso F, Torracca L, Benussi S, Alfieri O, Hong R, Lau W, Hirata K, Shikuma N, Hall B, Morady F. Atrio-esophageal fistula as a complication of percutaneous transcatheter ablation of AF. Circulation 2004; 109:2724-2726.
54. Stabile G, Bertaglia E, Senatore G, De Simone A, Zoppo F, Donnici G, Turco P, Pascotto P, Fazzari M, Vitale DF. Catheter ablation treatment in patients with drug-refractory atrial fibrillation: a prospective, multi-centre, randomized, controlled study (Catheter Ablation For The Cure Of Atrial Fibrillation Study). Eur Heart J. 2006; 27(2):216-221.
55. Wazni OM, Marrouche NF, Martin DO, Verma A, Bhargava M, Saliba W, Bash D, Schweikert R, Brachmann J, Gunther J, Gutleben K, Pisano E, Potenza D, Fanelli R, Themistoclakis S, Rosillo A, Bonso A, Natale A. Radiofrequency ablation vs. antiarrhythmic drugs as first-line treatment of symptomatic atrial fibrillation. JAMA 2005; 293: 2634-2640.
56. Pappone C, Augello G, Sala S, et al. A controlled randomized trial of circumferential pulmonary vein ablation versus antiarrhythmic drug therapy for curing paroxysmal atrial fibrillation. The Ablation of Paroxysmal Atrial Fibrillation (APAF) trial. Abstract 302-6. American College of Cardiology 55th Annual Scientific Sessions. Late Breaking Clinical Trials. March 11-14, 2006 Atlanta Georgia.
57. Jais P, Cauchemez B, Macle K, Daoud E, Waldo AL, Ruskin JN, Hocini M, O'Niell MD, Clementy J, Haissaguerre M. Atrial Fibrillation Ablation vs Anti-arrhythmic Drugs: A Multi-center Randomized Trial. Circulation 2008; 118: 2498-2505.
58. Hsu LF, Jais P, Sanders P, Garrigue S, Hocini M, Sacher F, Takahashi Y, Rotter M, Pasquie JL, Scavee C, Bordachar P, Clementy J, Haissaguerre M. Catheter ablation for atrial fibrillation in congestive heart failure. N Engl J Med. 2004; 351(23):2373-2383.
59. Chen M, Marrouche N, Khaykin Y, Gillinov A, Wazni O, Martin D, Rossillo A, Verma A, Cummings J, Erciyes D, Saad E, Bhargava M, Bash D, Schweikert R, Burkhardt D, Williams-Andrews M, Perez-Lugones A, Adbul-Karim A, Saliba W, Natale A. Pulmonary vein isolation for the treatment of atrial fibrillation in patients with impaired systolic function. J Am Coll of Cardiol. 2004; 43(6):1010-1012.
60. Gentlesk P, Sauer W, Gerstenfeld E, et al. Reversal of left ventricular dysfunction following ablation of atrial fibrillation. *J Cardiovasc Electrophysiol.* 2007; 18(1):9-14.
61. Cha YM, Asirvatham SJ, Shen WK, Friedman PA, Munger TM, Monahan KH, Haroldson JC, Hammill SC, Packer DL. Catheter ablation of atrial fibrillation associated with left ventricular dysfunction: Rate, regularity, and atrial contraction: is one more important than the others? Submitted for Publication.
62. Packer DL. CABANA Investigator Site Feasibility Survey. Unpublished, Available at Mayo Clinic Arrhythmia Research Center. 2008.
63. Dorian P, Cvitkovic S, Kerr C, Crysta E, Gillis A, Guerra P, Mitchell L, Roy D, Skanes A, Wyse D. A novel, simple scale for assessing the symptom severity of atrial fibrillation at the bedside: the CCS-SAF scale. *Canadian J of Cardiol.* 2006; 22(5):383-386.
64. AFFIRM Investigators. Relationship between sinus rhythm, treatment, and survival in the AF Follow-up Investigation of Rhythm Management (AFFIRM) Study. Circulation 2004; 109: 1509-1513.
65. Pedersen OD, Bagger H, Keller N, Marchant B, Kober L, Torp-Padersen C. Efficacy of dofetilide in the treatment of AF-flutter in patients with reduced left ventricular function: a Danish investigation of arrhythmia and mortality on dofetilide (DIAMOND) substudy. Circulation 2001; 104:292-6.

66. Rienstra M, Van Gelder IC, Hagens VE, Veeger NJ, Van Veldhuisen DJ, Crijns HJ. Mending the rhythm does not improve prognosis in patients with persistent atrial fibrillation: a subanalysis of the RACE study. *Euro Hrt J*. 2006; 27(3):357–64.
67. Nademanee K, Schwab MC, Kosar EM, Karwecki M, Moran MD, Visessook N, Michael AD, Ngarmukos T. Clinical outcomes of catheter substrate ablation for high-risk patients with atrial fibrillation. *J Am Coll Cardiol*. 2008; 51:843–49.
68. Van Gelder I, Hemels M. The progressive nature of atrial fibrillation: a rationale for early restoration and maintenance of sinus rhythm. *Europace.* 2006; 8(11):943-949.
69. Page RL, Wilkinson WE, Clair WK, McCarthy EA, Pritchett EL. Asymptomatic arrhythmias in patients with symptomatic paroxysmal atrial fibrillation and paroxysmal supraventricular tachycardia. Circulation. 1994; 89(1):224-7.
70. Vasamreddy CR, Dalal D, Dong J, Cheng A, Spragg D, Lamiy SZ, Meininger G, Henrikson CA, Marine JE, Berger, R, and Calkins H. Symptomatic and asymptomatic atrial fibrillation in patients undergoing radiofrequency catheter ablation. J Cardiovasc Electrophysiol 2006; 17:134-39.
71. Hindricks G, Piorkowski C, Tanner H, Kobza R, Gerds-Li JH, Carbucicchio C, Kottkamp H. Perception of atrial fibrillation before and after radiofrequency catheter ablation: relevance of asymptomatic arrhythmia recurrence. Circulation 2005; 112(3): 307-13.
72. Piorkowski C, Kottkamp H, Tanner H, Kobza R, Nielsen JC, Arya A, Hindricks G. Value of different follow-up strategies to assess the efficacy of circumferential pulmonary vein ablation for the curative treatment of atrial fibrillation. J Cardiovasc Electrophysiol 2005; 16(12): 1286-92.
73. Flaker GC, Belew K, Beckman K, Vidaillet H, Kron J, Safford R, Mickel M, Barrell P, AFFIRM Investigators. *Am Heart J*. 2005; 149(4):657–63.
74. Oral H, Veerareddy S, Good E, Hall B, Cheung P, Tamirisa K, Han J, Fortino J, Chugh A, Bogun F, Pelosi F, Jr., Morady F. Prevalence of asymptomatic recurrences of atrial fibrillation after successful radiofrequency catheter ablation. J Cardiovasc Electrophysiol. 2004; 15(8):920-924.
75. Verma A, Minor S, Kilicaslan F, Patel D, Hao S, Beheiry S, Lakkireddy D. Elayi SC, Cummings J, Martin DO, Burkhardt JD, Schweikert RA, Saliba W, Tchou PJ, Natale A. Incidence of atrial arrhythmias detected by permanent pacemakers (PPM) post-pulmonary vein antrum isolation (PVIA) for atrial fibrillation (AF): Correlation with symptomatic recurrence. *J Cardiovasc Electrophysiol*. 2007; 18(6):601–6.
76. Chan P, Vijan S, Morady F, Oral H. Cost-effectiveness of radiofrequency catheter ablation for atrial fibrillation. J Am Coll Cardiol 2006; 47(12): 2513-20.
77. Khaykin Y. Cost-effectiveness of catheter ablation for atrial fibrillation. *Current Opinion in Cardiology.* 2007; 22(1):11-17.
78. Reynolds M, Lavelle T, Essebag V, Cohen D, Zimetbaum P. Influence of age, sex, and atrial fibrillation recurrence on quality of life outcomes in a population of patients with new-onset atrial fibrillation: the Fibrillation Registry Assessing Costs, Therapies, Adverse events and Lifestyle (FRACTAL) study. *Am Heart J.* 2006; 152(6):1097-1103.
79. Falk R. Atrial fibrillation or sinus rhythm? Controversy and contradiction in quality of life outcomes. *J Am Coll Cardiol.* 2006; 48(4):731-733.
80. Schron E, Jenkins L. Quality of life in older patients with atrial fibrillation. *Am J Geriatric Cardiol.* 2005; 14(2):87-90.
81. Weerasooriya R, Jais P, Hocini M, Scavee C, MacLe L, Hsu F, Sandars P, Garrigue S, Clementy J, Haissaguerre M. Effect of catheter ablation on quality of life of patients with paroxysmal atrial fibrillation. *Heart Rhythm* 2005; 2(6): 619-23.
82. Wokhlu A, Hodge DO, Haroldson J, Wock K, Monahan KH, Friedman PA, Asviratham S, Munger TM, Packer DL. Long-term quality of life after ablation for atrial fibrillation: symptom relief or placebo effect? *Heart Rhythm*. 2008: Abstract in press.

83. Pappone C, Oreto G, Rosanio S, Vicedomini G, Tocchi M, Gugliotta F, Salvati A, Dicandia C, Calabro M, Mazzone P, Ficarra E, DiGioia D, Gulletta S, Nardi S, Santinelli V, Benussi S and Alfieri O. Atrial electroanatomic remodeling after circumferential radiofrequency pulmonary vein ablation. Efficacy of an anatomic approach in a large cohort of patients with atrial fibrillation. Circulation. 2001; 104: 2539 - 44.
84. Beukema WP, Elvan A, Sie HT, Misier AR, Wellens HJ. Successful radiofrequency ablation in patients with previous atrial fibrillation results in a significant decrease in left atrial size. *Circulation*. 2005; 112(14): 2089-95.
85. Tops LF, Bax JJ, Zeppenfeld K, Jongbloed MR, van der Wall EE, Schalij M. Effect of radiofrequency catheter ablation for atrial fibrillation on left atrial cavity size. *Am J Cardiol.* 2006; 97(8): 1220-2.
86. Reant P, Lafitte S, Jais P, Serri K, Weerasooriya R, Hocini M, Pillois X, Clementy J, Haissaguerre M, Roudaut R. Reverse remodeling of the left cardiac chambers after catheter ablation after 1 year in a series of patients with isolated atrial fibrillation. *Circulation* 2005; 112(19): 2896-903.
87. Verma, A, Marrouche N, Yamada H, Grimm R, Cummings J, Burkhardt J, Kilicaslan F, Fhargava M, Karim A, Thomas J, Natale A. Usefulness of intracardiac Doppler assessment of left atrial function immediately post-pulmonary vein antrum isolation to predict short-term recurrence of atrial fibrillation. *Am J Cardiol*. 2004; 94(7): 951-4.
88. Lemola K, Desjardins B, Sneider M, Case I, Chugh A, Good E, Han J, Tamirisa K, Tsemo A, Reich S, Tschopp D, Igic P, Elmouchi D, Bogun F, Pelosi F Jr., Kazerooni E, Morady F, Oral H. Effect of left atrial circumferential ablation for atrial fibrillation on left atrial transport function. *Heart Rhythm* 2005; 2(9): 923-8.
89. Chandrasekaran K, Asirvatham S, Packer D. Wide area circumferential ablation impairs the left atrial function significantly compared to Lasso-guided pulmonary vein ablation in chronic atrial fibrillation. Mayo Clinic Data 2006.
90. Rosendorff C, Black HR, Cannon DP, Gersh BJ, Gore J, Izzo JL Jr., Kaplan NM, O'Connor CM, O'Gara PT, Oparil S. Treatment of hypertension in the prevention and management of ischemic heart disease: A scientific statement from the American Heart Association Council for High Blood Pressure Research and the Councils on Clinical Cardiology and Epidemiology and Prevention. Hypertension 2007; 50:e28-55.
91. American Diabetes Association. Diagnosis and classification of diabetes mellitus. *Diabetes Care.* 2008; 31 (Suppl 1):S55-60.
92. Fuster V, Ryden LE, Cannom DS, Crijns HJ, Curtis AB, Ellenbogen KA, Halperin JL, Le Heuzey JY, Kay GN, Lowe JE, Olsson SB, Prystowsky EN, Tamargo JL, Wann S, Smith SC Jr, Jacobs AK, Adams CD, Anderson JL, Antman EM, Halperin JL, Hunt SA, Nishimura R, Ornato JP, Page RL, Riegel B, Priori SG, Blanc JJ, Budaj A, Camm AJ, Dean V, Deckers JW, Despres C, Dickstein K, Lekakis J, McGregor K, Metra M, Morais J, Osterspey A, Tamargo JL, Zamorano JL, American College of Cardiology/American Heart Association Task Force on Practice Guidelines. European Society of Cardiology Committee for Practice Guidelines. European Heart Rhythm Association. Heart Rhythm Society. ACC/AHA/ESC 2006 Guidelines for the Management of Patients with Atrial Fibrillation: a report of the American College of Cardiology/American Heart Association Task Force on Practice Guidelines and the European Society of Cardiology Committee for Practice Guidelines (Writing Committee to Revise the 2001 Guidelines for the Management of Patients With Atrial Fibrillation): developed in collaboration with the European Heart Rhythm Association and the Heart Rhythm Society. Circulation. 114(7):e257-354, 2006 Aug 15.
93. Haissaguerre M, Jais P, Shah D, Takahashi A, Hocini M, Quiniou G, Garrigue S, LeMouroux A, LeMetayer P, Clementy J. Spontaneous initiation of AF by ectopic beats originating in the pulmonary veins. *New England Journal of Medicine.* 1998; 339:659-66.

94. Haissaguerre M, Jais P, Shah D, Lavergne T, Hocini M, Peng J, Roundaut R, Clementy J. Electrophysiological endpoint for catheter ablation of AF initiated from multiple pulmonary venous foci. *Circulation*. 2000; 101:1409-17.
95. Marrouche N, Martin D, Wazni O, Gillinov A, Klein A, Bhargava M, Saad E, Bash D, Yamada H, Jaber W, Schweikert R, Tchou P, Abul-Karim A, Saliba W, Natale A. Phased-array intracardiac echocardiography monitoring during pulmonary vein isolation in patients with AF: Impact on outcome and complications. *Circulation*. 2003; 107:2710–16.
96. Verma A, Marrouche NF, Natale A. Pulmonary vein antrum isolation: intracardiac echocardiography-guided technique. *J Cardiovasc Electrophysiol.* 2004; 15(11):1335-40.
97. Pappone C, Rosanio S, Vicedomini G, Tocchi M, Gugliotta F, Salvati A, Dicandia C, Calabro MP, Mazzone P, Ficarra E, DiGioia C, Gulletta S, Nardi S, Santinelli V, Benussi S, Alfieri O. Atrial electroanatomic remodeling after circumferential radiofrequency pulmonary vein ablation. Efficacy of an anatomic approach in a large cohort of patients with AF. *Circulation.* 2001; 104:2539-44.
98. Packer DL, Bluhm CM, Monahan KH, Asirvatham S, Friedman A, Shen WK, and Haroldson JM. Outcome of AF Ablation Using a Wide Area Circumferential Ablative Approach: A Comparison With Lasso-Guided Ablation. *Heart Rhythm*. 2004; 1(1S):S11.
99. Nakagawa H, Scherlag BJ, Lockwood D, Wolf RK, Peyton M, Wu R, Yokoyama K, Po SS, Herring L, Lazzara R, Jackman WM, Armour JA. Localization of left atrial autonomic ganglionated plexuses using endocardial and epicardial high frequency stimulation in patients with AF. *Heart Rhythm.* 2005; 2(1S):S10. Abstract.
100. Uyttenboogaart M, Stewart RE, Vroomen PCAJ, De Keyser J, Luijckx GJ. Optimizing cutoff scores for the Barthel Index and the Modified Rankin Scale for defining outcome in acute stroke trials. *Stroke.* 2005; 36:1984-87.
101. Rao SV, O'Grady K, Pieper KS, Granger CB, Newby LK, Mahaffey KW, Moliterno DJ, Lincoff AM, Armstrong PW, Van de Werf F, Califf RM, Harrington RA. A Comparison of the Clinical Impact of Bleeding Measured by Two Different Classifications Among Patients with Acute Coronary Syndromes. *J Am Coll Cardiol*. 2006; 47:809-16.
102. Kalbfleisch JD, Prentice RL. *The Statistical Analysis of Failure Time Data (Second Edition).* . New York, NY: John Wiley & Sons, Inc, 2002.
103. Cox DR. Regression models and life-tables (with discussion). J Royal Statist Soc B 1972; 34:187-220.
104. Schoenfeld DA. Sample-size formula for the proportional-hazards regression model. Biometrics 1983; 39(2):499-503. PMID:6354290
105. O'Brien PC, Fleming TR. A multiple testing procedure for clinical trials. Biometrics 1979; 35(3):549-556.56. PMID:497341
106. Lan KKG, DeMets DL. Discrete sequential boundaries for clinical trials. Biometrika 1983; 70:659-663.
107. Bang H, Tsiatis AA. Estimating medical costs with censored data. Biometrika 2000; 87: 329-343.

Appendix A Adverse Events

The following events have been identified as "Anticipated Events" for subjects randomized to drug or ablation therapy for this study.

CATEGORY		ANTICIPATED EVENT		
**CARDIOVASCULAR**				
	1	Air embolism	17	Intraventricular conduction delay
	2	Bradycardia	18	Ischemia
	3	Cardiac arrest	19	Left ventricular dysfunction
	4	Cardiac thromboembolic event	20	Myocardial infarction
	5	Chest pain during energy delivery	21	Myocardial perforation
	6	Clinically relevant AV node dysfunction	22	Pacemaker damage
	7	Clinically relevant sinus node block	23	Pericardial effusion
	8	Complete/permanent AV block	24	Pericarditis
	9	Coronary artery dissection	25	Proarrhythmia; new or worsened arrhythmia
	10	Coronary artery occlusion	26	Persistant PFO / iatrogenic ASD
	11	Coronary artery spasm	27	Prolonged QT
	12	Nonsustained VT	28	Sustained VT
	13	Elevated creatinine phosphokinase (CPK)	29	Tamponade
	14	Heart failure (Class I, II, III, IV)	30	Torsade des pointes
	15	Heart valve damage	31	Transient AV block
	16	Hypotension	32	Ventricular fibrillation
**ENDOCRINE**				
	32	Hyperthyroidism	33	Hypothyroidism
**GENERAL**				
	34	Allergic reaction (skin rash, SOB)	39	Radiation skin burn
	35	Bluish / gray skin tone	40	Skin rashes
	36	Fatigue	41	Temperature elevation
	37	Photosensitivity	42	Volume overload
	38	Radiation related cancers		
**GASTROINTESTINAL**				
	43	Constipation	48	Stomach disorder
	44	Diarrhea	49	Nausea
	45	Esophageal atrial fistula	50	Poor appetite
	46	Esophageal disorder	51	Unusual taste (metallic or other)
	47	Gastroesophageal reflux	52	Vomiting
			53	Abnormal liver functions
**GENITOURINARY**				
	54	Impotence	56	Renal failure
	55	Kidney damage	57	Urinary tract infection
**INFECTIOUS**				
	58	Infection	59	Sepsis
**NEUROLOGIC**				
	60	Alteration of color vision	68	Phrenic nerve damage
	61	Blindness	69	Seizure
	62	Blurred / double vision	70	Stroke
	63	Depression	71	Syncope
	64	Deteriorating vision	72	TIA
	65	Dizziness / light headedness	73	Unsteady gait / imbalance
	66	Headache	74	Vasovagal reaction
	67	Pain	75	Visual migraine

Appendix A Adverse Events (continued)

The following events have been identified as "Anticipated Events" for subjects randomized to drug or ablation therapy for this study.

CATEGORY		ANTICIPATED EVENT		
**PULMONARY**				
	76	Asthma exacerbation	84	Pulmonary hypertension
	77	Hemothorax	85	Pulmonary vein damage/dissection
	78	Lung toxicity	86	Pulmonary vein stenosis
	79	Pleural effusion	87	Pulmonary vein thrombus
	80	Pneumonia	88	Respiratory depression
	81	Pneumothorax	89	Shortness of breath/dyspnea
	82	Pulmonary edema	90	Wheezing
	83	Pulmonary embolism	91	Pleuritic chest pain
**PERIPHERAL VASCULAR**				
	92	A-V fistula	97	Peripheral edema
	93	Bleeding	98	Peripheral thromboembolic event
	94	Bruising / ecchymosis	99	Pseudoaneurysm
	95	DVT	100	Thromboembolic event
	96	Hematoma	101	Vessel trauma

Appendix B

Approved Ablation Devices for CABANA
IDE: G050233

CATHETER	DESCRIPTION	SIZE	PMA#
Biosense Webster	NAVI-STAR	7F/4mm	P990025
Biosense Webster	NAVI-STAR DS	7F/8mm	P010068
Biosense Webster	CELSIUS Braided Tip	7F/4-5mm	P950005
Biosense Webster	CELSIUS Braided Tip	8F/4-5mm	P950005
Biosense Webster	CELCIUS Long Reach	7F/4-5mm	P950005
Biosense Webster	CELCIUS Long Reach	8F/4-5mm	P950005
Biosense Webster	NAVI-STAR™ and CELSIUS™ Thermo-Cool	7.5F/3.5mm	P030031
Biosense Webster	Navistar® RMT	7F/4mm	P990025
Biosense Webster	Navistar® RMT	7F/8mm	P010068
Biosense Webster	Celsius® RMT	7F/4mm	P950005
Biosense Webster	ThermoCool® SF	8F/3.5mm	P990071
Medtronic Cryocath LP	Freezor®	7F/4mm	P020045
Medtronic Cryocath LP	Freezor® Xtra	7F/6mm	P020045
Medtronic Cryocath LP	Freezor® MAX	9F/8mm	P020045
Medtronic Cryocath LP	Arctic Front®	23mm 28mm	P100010
Medtronic	Cardiac Ablation System		P100008
St. Jude Medical	Livewire TC™ XLS™ Medium Sweep	7F/4mm	P960016
St. Jude Medical	Livewire TC™ XLS™ Large Sweep	7F/4mm	P960016
St. Jude Medical	Livewire TC™ XLS™ Medium Curl	7F/4mm	P960016
St. Jude Medical	Livewire TC™ XLS™ Large Curl	7F/4mm	P960016
St. Jude Medical	Livewire TC™ XLS™ Medium Sweep	7F/5mm	P960016/S006
St. Jude Medical	Livewire TC™ XLS™ Large Sweep	7F/5mm	P960016/S006
St. Jude Medical	Livewire TC™ XLS™ Medium Curl	7F/5mm	P960016/S006
St. Jude Medical	Livewire TC™ XLS™ Large Curl	7F/5mm	P960016/S006
St. Jude Medical	Therapy™ Dual8- small	7F/8mm	P040042
St. Jude Medical	Therapy™ Dual 8- medium	7F/8mm	P040042
St. Jude Medical	Therapy™ Dual 8- large	7F/8mm	P040042
St. Jude Medical	Therapy™ Dual 8- X-large	7F/8mm	P040042
St. Jude Medical	Therapy™ Dual -8 extended	7F/8mm	P040042
St. Jude Medical	Therapy™ Dual -8 far reach	7F/8mm	P040042
St. Jude Medical	Therapy™ Thermocouple small	7F/4mm	P040014
St. Jude Medical	Therapy™ Thermocouple medium	7F/4mm	P040014
St. Jude Medical	Therapy™ Thermocouple large	7F/4mm	P040014
St. Jude Medical	Therapy™ Thermocouple X-large	7F/4mm	P040014
St. Jude Medical	Therapy™ Thermocouple extended	7F/4mm	P040014
St. Jude Medical	Therapy™ Thermocouple far reach	7F/4mm	P040014
St. Jude Medical	Safire™ Sm/Med/Lg Sweep	7F/4mm	P960016/S014
St. Jude Medical	Safire™ Sm/Med/Lg Curl	7F/4mm	P960016/S014
St. Jude Medical	Safire™ Sm/Med/Lg Sweep	7F/5mm	P960016/S014
St. Jude Medical	Safire™ Sm/Med/Lg Curl	7F/5mm	P960016/S014
St. Jude Medical	Therapy Cool Path Med Curve	7F/5mm	P060019
St. Jude Medical	Therapy Cool Path Large Curve	7F/5mm	P060019
St. Jude Medical	Therapy Cool Path X-Large Med Curve	7F/5mm	P060019
St. Jude Medical	Therapy Cool Path Flutter Curve	7F/5mm	P060019
Bard	Stinger	4&5mm	P000020
Boston Scientific	Blazer II RF	7F/4mm	P920047
Boston Scientific	Blazer II XP	8F/8mm	P020025
Boston Scientific	Blazer RPM	8F/5mm	P020047
Boston Scientific	Chilli II Cooled	7F4mm	P980003
Boston Scientific	SteeroCath	7F/4mm	P920047

Appendix C

CABANAgene

A Resource to Evaluate Genetic Predictors of Efficacy and Adverse Reactions During the Catheter Ablation Versus Antiarrhythmic Drug Therapy for Atrial Fibrillation Trial (CABANA) (CABANA sub-study protocol)

Dawood Darbar,[1] M.D., and Dan M. Roden,[2] M.D.
[1]Associate Professor of Medicine,
Chief, Arrhythmia Service

[2]Professor of Medicine and Pharmacology
Director, Oates Institute for Experimental Therapeutics
Assistant Vice-Chancellor for Personalized Medicine

Vanderbilt University Medical Center

Table of Contents:

1.0 Background
2.0 Rationale and Specific Aims
3.0 Animal Studies and Previous Human Studies
4.0 Inclusion/Exclusion Criteria
5.0 Enrollment/Randomization
6.0 Study Procedures
7.0 Risks of Investigational Agents/Devices (side effects)
8.0 Reporting of Adverse Events or Unanticipated Problems involving Risk to Participants or Others
9.0 Study Withdrawal/Discontinuation
10.0 Statistical Considerations
11.0 Privacy/Confidentiality Issues
12.0 Follow-up and Record Retention

1.0 Background and Significance

CABANA is comparing two major approaches for management of atrial fibrillation (AF): drugs to maintain sinus rhythm and ablation. As in all other large clinical trials, responses to therapy will be variable in both treatment arms. Abundant evidence points to genetic factors as a contributor to such variability in important human phenotypes such as response to treatment,[1] and the goal of CABANAgene is the creation of a large resource linking to test hypotheses that relate genetic variation to disease susceptibility and treatment responses. Collecting DNA from patients in CABANA will address questions such as which patients are most likely to respond to ablation or drug therapy and which patients are most likely to develop complications with ablation or drug therapy.

Approaches to identifying associations between genotypes and phenotypes: Three general approaches have been used in studies addressing the relationship between genotype and phenotype:

1. Family studies: Initial genotype-phenotype relations were identified by studying kindreds with manifest phenotypes like sickle cell disease, long QT syndrome or hypertrophic cardiomyopathy. Indeed, similar approaches have now been used to identify rare but clinically important genetic variants that cause familial AF.[2-7]
2. Candidate gene studies: Here, the biologic basis for the phenotype in question is first intensively studied, and then the role of genes modulating that biology was explored. This approach has been used to identify genetic variants contributing to AF susceptibility in families with more than one affected member. In addition, candidate gene approaches have been remarkably effective in identifying genetic variants that modulate drug action (pharmacogenomics).[1] These can be in the genes involved in drug transport or metabolism (e.g. members of the CYP superfamily), in genes encoding drug targets (e.g. ion channels genes for antiarrhythmics; adrenergic receptor gene variants for beta-blockers; VKORC1 for warfarin), or in other genes that modulate the biologic context in which drugs interact with their target molecules. The traditional approach interrogates a small number of well-characterized variants in a candidate gene, and relates these to the phenotype of interest; in CABANA, such phenotypes may include beneficial drug effect, failure of drug or ablation, or adverse drug effect. Newer "next-generation" sequencing technologies can interrogate whole candidate genes or pathways, and analytical approaches to identifying true positive signals in this work are in development.
3. "Unbiased" approaches: Human genomes contain millions of common polymorphisms, such as single nucleotide polymorphisms (SNPs) or copy number variations (CNVs). The genome-wide association study (GWAS) paradigm[8,9] compares hundreds of thousands of such variants in cases and controls (or across a continuous trait) to identify, in an unbiased fashion, loci modulating the trait. Because GWAS searches for common variants that confer modest odds ratios, large cohorts are required,[10,11] and there is a high risk of false positive associations; thus, the accrual of a very large well-ascertained cohorts (as in this proposal) represents an important enabling tool for these studies. A compromise approach has been to focus not on high priority single genes or single variants, but to cast a somewhat wider net and focus on variation in genes within a "pathway", e.g. a cholesterol[12] or thrombosis "pathway". Indeed, this approach has been adopted to interrogate >1500 SNPs in 18 candidate genes in an "arrhythmia drug response pathway" to study determinants of drug-induced arrhythmias, among others.[13]

2.0 Rationale and Specific Aims

The primary goal of the Catheter Ablation Versus Antiarrhythmic Drug Therapy for Atrial Fibrillation (CABANA) trial is to establish appropriate roles for medical and ablative intervention by randomizing 2,000 – 2,200 patients. **This protocol will create CABANAgene: a resource that will accumulate DNA samples from CABANA subjects to enable subsequent genotype-phenotype studies, based on the rationales described above.**
Examples of issues that CABANAgene could address include:

- predictors of response to antiarrhythmic or rate control drug therapies
- predictors of response to ablation therapy
- predictors of response to anticoagulation therapy
- genetic factors defining AF subtypes
- predictors of LA morphology size and function
    - Is it primary AF or AF secondary to underlying heart disease
    - Is it early recurrent atrial fibrillation (ERAF)
    - Does it increase the non pulmonary vein foci.
    - Left Atria and Stroke
    - Anticoagulant issues

All projects will require approval of the CABANAgene Principal Investigators and CABANA Executive Committee. The CABANAgene project is included in the Pharmacogenetics of Arrhythmia Therapy (PAT) node of the Pharmacogenomics Research Network (PGRN). CABANAgene's participation under the PGRN umbrella will also provide access to advanced genotyping and genetic statistical methods via this resource.

3.0 Previous Human Studies

PAT investigators, in collaboration with other arrhythmia groups around the country and internationally, have used family studies, candidate gene studies, and unbiased approaches to identify genetic predictors of variability in AF susceptibility and in response to drugs used to treat AF. Examples of these findings are presented here in brief to illustrate the value of a resource such as CABANAgene with well-phenotyped patients and outcomes in advancing AF-related genome science. One important conclusion to draw from the studies presented is that large datasets, such as Framingham, the Vanderbilt AF registry, the German AF network, or CABANAgene are enabling for contemporary unbiased genomic studies.[14-17] In addition, large datasets permit the accrual of subjects with specific intermediate phenotypes that may relate to AF risk. Examples of such phenotypes include prolonged P wave duration,[4] atrial fibrillatory rate derived from frequency-domain analysis of surface ECGs during AF,[18] and right precordial J point elevation, that is reported to be common in lone AF[19] and that we have observed with AF-associated mutations in sodium channel genes.[20] A resource such as CABANAgene will allow much more rigorous exploration of the relationships among these endophenotypes, genetic variants that underlie them, and clinical course in AF.

Family studies

With the support of the NHLBI's Resequencing and Genotyping (RS&G) resource, PAT investigators screened subjects in the Vanderbilt AF Registry for variants in genes encoding ion candidate channel subunits (KCNQ1, KCNE1-5, KCNJ2, KCNA5, sodium channel [α- and β-subunits], L-type Ca^{2+}) and non-ion channel protein candidates (connexin40, PITX2, ANP). Non-synonymous variants in sodium channel α-subunits, β-subunits, and potassium channels have been identified in ~10% of subjects. These cosegregate in extended kindreds (where they are available), and those studied to date show abnormal electrophysiology *in vitro*.[5,20,21] One common feature in these kindreds is that penetrance is variable; that is, each kindred includes multiple mutation

carriers with early onset AF as well as other mutation carriers with no AF by age 50. As described below, GWAS has identified common variants at chromosome 4q25 as risk factors for atrial fibrillation. We studied 12 families in which AF was present in ≥2 individuals who also shared a rare genetic variant in ion channel or related genes thought to cause familial AF. There were a total of 33 subjects with AF before age 40 and 17 mutation carriers who had reached age 50 without AF. We found a very highly statistically significant association between the presence of common 4q25 variants and clinical AF in these families ($P<10^{-9}$): that is, those family members with AF carried both rare mutation as well the common susceptibility allele, while those with only the rare mutation (without the 4q25 susceptibility allele) did not display AF.[22] These data support the overall concept that AF risk includes multiple genomic components; identifying these components requires large datasets. In addition, the rare ion channel and other variants that we and others have identified in patients with AF have obvious (but as yet untested) therapeutic implications. A gain of function potassium channel mutation would be predicted to respond to a potassium blocking drug, while sodium channel blockers would be predicted to be ineffective (and possibly proarrhythmic) in subjects with AF arising from slow conduction or decreased sodium current.

Candidate gene studies

Candidate gene approaches have been especially useful in pharmacogenomics, where single variants in genes responsible for drug disposition or for the interaction of drugs with their pharmacologic targets may produce large and clinically important variability in drug action.[1] This is especially the case for drugs with narrow therapeutic indexes such as antiarrhythmic and anticoagulant agents. We and many others (including the International Warfarin Pharmacogenomic Consortium)[23-27] have shown that ~50% of the variability in warfarin steady state dose is determined by variants in CYP2C9 (responsible for the drug's metabolism) and VKORC1 (encoding the warfarin target). In addition, PAT investigators have shown that the time to initial anticoagulation is determined by VKOC1 variants. The extent to which warfarin use will be supplanted by newer agents in CABANA is uncertain, but the collection of a large set of patients with well-characterized warfarin responses will be of considerable interest to the IWPC and other investigators in the warfarin and anticoagulant areas.

Proarrhythmia is one well-recognized risk during drug therapy for AF. We have used both candidate and unbiased approaches to address risk for drug-induced torsades de pointes (diTdP) in large cohorts generated by collaborations among multiple sites. Screening coding and flanking regions of these has identified potential contributory rare DNA variants, i.e. sub-clinical congenital long QT syndrome, in <10-20% of patients with diTdP. We used a candidate pathway approach to interrogate 1536 common SNPs in 18 candidate genes and identify rs1805128, resulting in *KCNE1* D85N, as a risk allele: the allelic odds ratio for 176 cases versus 530 drug-exposed controls was 9.0 (95% confidence interval: 3.5-22.9), and the variant allele was present in 8.6% of cases, 2.9% of drug-exposed controls, and 1.8% of population controls. Other drug hypothesis: Do drugs create problems due to genetics? Is there a 'hopeless' patient population?

Unbiased approaches

The GWAS paradigm has been successfully applied to identify risk loci for AF at Chromosomes 4q25, 1q21 and 16q22.[14,15,28] Interestingly, these loci not only represent risk factors for the development of AF, but also appear to predict development of AF after cardiac surgery,[29] stroke,[30] and (in smaller studies) the success of ablation therapy[31] and of antiarrhythmic or rate control therapy in AF.[32,33] The biologic underpinnings of this increased risk are now being explored in mouse models and for the 4q25 locus (the one with the largest signal), evidence points to variable expression of a cardiac isoform of the transcription factor PITX2c.[34,35] Consistent with this observation is the finding that multiple SNPs at the 4q25 locus act independently to modulate AF risk;[36] this finding allows the identification of small sets of subjects drawn from larger databases

(such as CABANA) in whom response to ablative or medical therapies may differ on a genetic basis. GWAS has also been applied to study warfarin steady state dose, and has identified variants in CYP2C9, VKORC1, and CYP4F2 (which plays a role in vitamin K metabolism) with major effects, but no other common variants.[25,26]

4.0 Inclusion/Exclusion Criteria

Inclusion/exclusion in this study is dependent only on inclusion/exclusion in the primary study. The CABANA specific criteria can be found in Appendix A.

5.0 Enrollment/Randomization

All enrollment will be performed at CABANA participating centers and subjects enrolled in CABANA are eligible to participate in this ancillary study. There will be no randomization specific to CABANAgene. No subjects will be enrolled at the CABANAgene Coordinating Center.

6.0 Study Procedures

Consent: PGRN has developed standard suggested language for consent to perform genomic analyses of the type proposed here, subject to local IRB revision. We have created a model consent form in which this language was incorporated and this form is provided to each site for local modification if necessary and IRB submission. IRBs at PGRN affiliated centers have adopted this or very similar language in most instances.

Current NIH guidelines require that the results of large genotyping efforts accomplished with NIH funds be deposited in public databases such as the database for Genotypes and Phenotypes (dbGaP). dbGaP submissions include deidentified phenotypic data; thus, current consent forms include consent for such deposits.

Once consent is obtained, a 30 mL blood sample for subsequent DNA extraction will be obtained at any time during the CABANA trial, preferably at the time of a scheduled venipuncture. Each study site will be provided with kits for mailing the samples to the Vanderbilt DNA Resources Core, where DNA extraction and archiving will be performed. Contents of each subject kit and instructions are listed in Appendix B. If it is determined that a sample is damaged/unusable upon arrival, communication with the sender will be made and a replacement sample will be requested.

The Vanderbilt DNA Resources Core has extensive experience in storing and extracting DNA from over 100,000 individuals. 2D bar-coding of samples and aliquots eliminates repetitive paper work, thereby reducing recording errors. All DNA is currently extracted using the automated Autopure system (Gentra systems, Minneapolis). Samples are logged into the system using a study specific code number to match with the assigned DNA Resources Core sample number, and only the sample number is used thereafter. Each sample must be associated with an IRB-approved consent form. The Autopure system generates very high quality, high yield (routinely 20-30 µg/ml), and substantial consistency across samples. All samples are quantitated and stored at 4°C in a locked cold room. An aliquot is also frozen at -20°C to serve as a back-up in case a sample is accidentally contaminated, lost, or depleted. DNA samples for approved CABANA projects can be provided to investigators in a range of formats, most commonly 96-well plates.

Results of the tests run on specific samples will not be shared with or reported to subjects, care providers, or the general public.

Genotyping

CABANA currently anticipates enrolling in 2009-2015, with an average of approximately 5 years follow-up. Thus initial analyses of end-points will not be available until interim analysis early 2018. We anticipate that investigators will develop hypotheses that require accrual of *specific genotypes* (e.g. CYP2D6 and beta-blocker response; 4q25 variants and response to ablation therapy). Further, we anticipate that the size and scope of the CABANA dataset will provide an opportunity for *directed resequencing* to test other hypotheses (e.g. do patients with a specific AF endophenotype have coding region variants in specific candidate genes?). Finally, we anticipate that *unbiased approaches* will be proposed to address other questions (e.g. are there genomic regions that predict response to ablation therapy?); we anticipate that genome-wide analysis will still be the standard for addressing these questions, although whole genome resequencing may be a competing method by 2014.

Funds to support accrual of DNA at participating centers, mailing costs, and DNA extraction at Vanderbilt have been incorporated into the CABANAgene proposal. We anticipate that the process to access CABANAgene samples by CABANA (or other) Investigators will require submission of a specific study plan, including appropriate quality control and statistical analysis, access to clinical variables in the central CABANA dataset, and approval by the CABANA steering committee.

The current PAT budget includes support for genome-wide genotyping and analysis of genotype-drug response phenotype relations and these will be used to develop a separate PAT proposal to access CABANA samples. The specific questions to be addressed and platform to be used will be selected at the time of projected analysis, at the termination of the trial (~3-5 years after study initiation). Technologies available at the Vanderbilt, and at multiple other sites in PGRN, include Affymetrix and Illumina high-throughput systems, as well as robust lower throughput systems for targeted genotyping, such as the Illumina Bead-station, Sequenom, or Taqman. Genotyping laboratories in PGRN adhere to standard practice, including appropriate negative and positive controls, to ensure that the genotyping results are robust. Genotypic and phenotypic data will be combined in an Oracle database to allow biostatistical and genetic epidemiologic analysis. The PAT center has developed expertise in robust quality control and statistical analyses of these large datasets.

7.0 Risks

Minor soreness and/or bruising when blood is taken, feeling lightheaded, or although rare, continued bleeding or infection at the site of the needle stick are risks involved at the participating centers.

The risk of released PHI exists, but this is minimized by the use of code numbers in the DNA Resources Core.

8.0 Reporting of Adverse Events or Unanticipated Problems involving Risk to Participants or Others

Any adverse events or unanticipated problems occurring at participating centers will be reported to the IRB of record. Any release of information that occurs will be immediately reported to the PI and the Vanderbilt IRB.

9.0 Study Withdrawal/Discontinuation

Participation in this study is voluntary. A patient who has agreed to participate in this study may leave at any time. This will not affect their regular care or cause them to lose any benefits they

would normally have. If a patient wants to withdraw, they must contact the primary study physician. Samples in storage at the Vanderbilt University DNA Resources Core will be destroyed upon notification from the participating centers. Research data that have already been gathered using the withdrawn subject's sample will not be withdrawn.

The investigator or research sponsor (NHLBI) may decide to withdraw a subject if the study is stopped. The sponsor may stop the study at any time.

10.0 Statistical Considerations

Study power will depend on the number of genotypes and other variables being assessed (the problem of multiple comparisons), the effect size, the sample size, and variance of the phenotype under study. For genome-wide experiments, even in a set of this magnitude, any analysis is likely to be underpowered and to require replication by analysis in other populations or by other approaches (e.g. studies of biologic function). We anticipate that use of the CABANAgene resource will be conditioned on a statistical plan appropriate for the study size proposed.

11.0 Privacy/Confidential Issues

Samples will only be labeled with a study identification (ID) number. No other data will be included that would identify a person. Any health information which might identify the patient will not be available to any person or group other than the investigators of this study. The files with the ID numbers that link the sample to the patient will be kept in a locked, secure area accessible only to the study team. The results of any study using the resource may be published for all the subjects as a group but will not identify the patient individually.

12.0 Follow-up and Retention

Samples for DNA extraction will be collected and genotyping will be performed the 4th year of the study. Following completion of the primary study, data analyses will be performed once genotyping is complete. Genotype data may be archived indefinitely for subsequent analyses. The DNA sample will be kept for an unknown length of time (maybe years) for future research. The sample will be destroyed when it is no longer needed. Samples will be used for research only and will not be sold or used to make products that could be sold for money.

1. Literature cited

1. Roden DM, Wilke RA, Kroemer HK, Stein CM. Pharmacogenomics: The genetics of variable drug responses. **Circulation, accepted for publication** 2011.
2. Darbar D, Herron KJ, Ballew JD, Jahangir A, Gersh BJ, Shen WK, Hammill SC, Packer DL, Olson TM. Familial atrial fibrillation is a genetically heterogeneous disorder. **J Am Coll Cardiol** 2003;41:2185-2192.
3. Hodgson-Zingman DM, Karst ML, Zingman LV, Heublein DM, Darbar D, Herron KJ, Ballew JD, de Andrade M, Burnett JC, Jr., Olson TM. Atrial Natriuretic Peptide Frameshift Mutation in Familial Atrial Fibrillation. **N Engl J Med** 2008;359:158-165.
4. Darbar D, Hardy A, Haines JL, Roden DM. Prolonged signal-averaged P-wave duration as an intermediate phenotype for familial atrial fibrillation. **J Am Coll Cardiol** 2008;51:1083-1089.
5. Abraham RL, Yang T, Blair M, George ALJ, Roden DM, Darbar D. Mechanism underlying familial atrial fibrillation in an ANP gain-of-function mutation. **Heart Rhythm** 2009;May.
6. Ellinor PT, Yoerger DM, Ruskin JN, Macrae CA. Familial aggregation in lone atrial fibrillation. **Hum Genet** 2005;118:179-184.
7. Ellinor PT, Nam EG, Shea MA, Milan DJ, Ruskin JN, Macrae CA. Cardiac sodium channel mutation in atrial fibrillation. **Heart Rhythm** 2008;5:99-105.
8. Manolio TA, Brooks LD, Collins FS. A HapMap harvest of insights into the genetics of common disease. **J Clin Invest** 2008;118:1590-1605.
9. Manolio TA. Genomewide Association Studies and Assessment of the Risk of Disease. **N Engl J Med** 2010;363:166-176.
10. Burton PR, Hansell AL, Fortier I, Manolio TA, Khoury MJ, Little J, Elliott P. Size matters: just how big is BIG? **International Journal of Epidemiology** 2009;38:263-273.
11. Altshuler D, Daly MJ, Lander ES. Genetic mapping in human disease. **Science** 2008;322:881-888.
12. Chasman DI, Posada D, Subrahmanyan L, Cook NR, Stanton VP, Jr., Ridker PM. Pharmacogenetic study of statin therapy and cholesterol reduction. **JAMA** 2004;291:2821-2827.
13. Kaab S, Sinner MF, George AL, Jr., Wilde AA, Bezzina CR, Schulze-Bahr E, Guicheney P, Bishopric NH, Myerburg RJ, Schott J, Pfeufer A, Hinterseer M, Beckmann BM, Steinbeck G, Perz S, Meitinger T, Wichmann H, Ingram C, Carter S, Norris K, Crawford DC, Ritchie M, Roden D. Abstract 4412: High Density tagSNP Candidate Gene Analysis Identifies IKs as a Major Modulator of Genetic Susceptibility to Drug Induced Long QT Syndrome. **Circulation** 2008;118:S_884.
14. Gudbjartsson DF, Arnar DO, Helgadottir A, Gretarsdottir S, Holm H, Sigurdsson A, Jonasdottir A, Baker A, Thorleifsson G, Kristjansson K, Palsson A, Blondal T, Sulem P, Backman VM, Hardarson GA, Palsdottir E, Helgason A, Sigurjonsdottir R, Sverrisson JT, Kostulas K, Ng MC, Baum L, So WY, Wong KS, Chan JC, Furie KL, Greenberg SM, Sale M, Kelly P, Macrae CA, Smith EE, Rosand J, Hillert J, Ma RC, Ellinor PT, Thorgeirsson G, Gulcher JR, Kong A, Thorsteinsdottir U, Stefansson K. Variants conferring risk of atrial fibrillation on chromosome 4q25. **Nature** 2007;448:353-357.
15. Gudbjartsson DF, Holm H, Gretarsdottir S, Thorleifsson G, Walters GB, Thorgeirsson G, Gulcher J, Mathiesen EB, Njolstad I, Nyrnes A, Wilsgaard T, Hald EM, Hveem K, Stoltenberg C, Kucera G, Stubblefield T, Carter S, Roden D, Ng MCY, Baum L, So WY, Wong KS, Chan JCN, Gieger C, Wichmann HE, Gschwendtner A, Dichgans M, Kuhlenbaumer G, Berger K, Ringelstein EB, Bevan S, Markus HS, Kostulas K, Hillert J, Sveinbjornsdottir S, Valdimarsson EM, Lochen ML, Ma RCW, Darbar D, Kong A, Arnar DO,

Thorsteinsdottir U, Stefansson K. A sequence variant in ZFHX3 on 16q22 associates with atrial fibrillation and ischemic stroke. **Nat Genetics** 2009;41:876-878.
16. Ellinor PT, Lunetta KL, Glazer NL, Pfeufer A, Alonso A, Chung MK, Sinner MF, de Bakker PI, Mueller M, Lubitz SA, Fox E, Darbar D, Smith NL, Smith JD, Schnabel RB, Soliman EZ, Rice KM, Van Wagoner DR, Beckmann BM, van NC, Wang K, Ehret GB, Rotter JI, Hazen SL, Steinbeck G, Smith AV, Launer LJ, Harris TB, Makino S, Nelis M, Milan DJ, Perz S, Esko T, Kottgen A, Moebus S, Newton-Cheh C, Li M, Mohlenkamp S, Wang TJ, Kao WH, Vasan RS, Nothen MM, Macrae CA, Stricker BH, Hofman A, Uitterlinden AG, Levy D, Boerwinkle E, Metspalu A, Topol EJ, Chakravarti A, Gudnason V, Psaty BM, Roden DM, Meitinger T, Wichmann HE, Witteman JC, Barnard J, Arking DE, Benjamin EJ, Heckbert SR, Kaab S. Common variants in KCNN3 are associated with lone atrial fibrillation. **Nat Genet** 2010;42:240-244.
17. Kaab S, Darbar D, van NC, Dupuis J, Pfeufer A, Newton-Cheh C, Schnabel R, Makino S, Sinner MF, Kannankeril PJ, Beckmann BM, Choudry S, Donahue BS, Heeringa J, Perz S, Lunetta KL, Larson MG, Levy D, Macrae CA, Ruskin JN, Wacker A, Schomig A, Wichmann HE, Steinbeck G, Meitinger T, Uitterlinden AG, Witteman JC, Roden DM, Benjamin EJ, Ellinor PT. Large scale replication and meta-analysis of variants on chromosome 4q25 associated with atrial fibrillation. **Eur Heart J** 2009;30:813-819.
18. Husser D, Stridh M, Sornmo L, Roden DM, Darbar D, Bollmann A. A Genotype Dependent Intermediate ECG Phenotype in Patients with Persistent Lone Atrial Fibrillation. **Circ Arrhythmia Electrophysiol** 2009;CIRCEP.
19. Junttila MJ, Raatikainen MJ, Perkiomaki JS, Hong K, Brugada R, Huikuri HV. Familial clustering of lone atrial fibrillation in patients with saddleback-type ST-segment elevation in right precordial leads. **Eur Heart J** 2007;28:463-468.
20. Watanabe H, Darbar D, Kaiser D, Jiramongkolchai K, Chopra S, Kucera G, Stubblefield T, Roden DM. Mutations in Sodium Channel Beta-1 and Beta-2 Subunits associated with Atrial Fibrillation. **Circ Arrhythmia Electrophysiol**. In press.
21. Darbar D, Kannankeril PJ, Donahue BS, Kucera G, Stubblefield T, Haines JL, George AL, Jr., Roden DM. Cardiac sodium channel (SCN5A) variants associated with atrial fibrillation. **Circulation** 2008;117:1927-1935.
22. Darbar D, Rowan SB, Kucera G, Stubblefield T, Blair M, Carter S, Roden DM, Ritchie MD. Chromosome 4q25 variants are genetic modifiers of rare ion channel mutations associated with familial atrial fibrillation. **American Society for Human Genetics annual meeting** 2010.
23. Schwarz UI, Ritchie MD, Bradford Y, Li C, Dudek SM, Frye-Anderson A, Kim RB, Roden DM, Stein CM. Genetic Determinants of Response to Warfarin during Initial Anticoagulation. **N Engl J Med** 2008;358:999-1008.
24. Rieder MJ, Reiner AP, Gage BF, Nickerson DA, Eby CS, McLeod HL, Blough DK, Thummel KE, Veenstra DL, Rettie AE. Effect of VKORC1 haplotypes on transcriptional regulation and warfarin dose. **N Engl J Med** 2005;352:2285-2293.
25. Takeuchi F, McGinnis R, Bourgeois S, Barnes C, Eriksson N, Soranzo N, Whittaker P, Ranganath V, Kumanduri V, McLaren W, Holm L, Lindh J, Rane A, Wadelius M, Deloukas P. A genome-wide association study confirms VKORC1, CYP2C9, and CYP4F2 as principal genetic determinants of warfarin dose. **PLoS Genet** 2009;5:e1000433.
26. Cooper GM, Johnson JA, Langaee TY, Feng H, Stanaway IB, Schwarz UI, Ritchie MD, Stein CM, Roden DM, Smith JD, Veenstra DL, Rettie AE, Rieder MJ. A genome-wide scan for common genetic variants with a large influence on warfarin maintenance dose. **Blood** 2008;112:1022-1027.
27. International Warfarin Pharmacogenetics Consortium, Klein TE, Altman RB, Eriksson N, Gage BF, Kimmel SE, Lee MT, Limdi NA, Page D, Roden DM, Wagner MJ, Caldwell MD,

Johnson JA. Estimation of the warfarin dose with clinical and pharmacogenetic data. **N Engl J Med** 2009;360:753-764.

28. Ellinor PT, Lunetta KL, Glazer NL, Pfeufer A, Alonso A, Chung MK, Sinner MF, deBakker P, Mueller M, Lubitz SA, Fox E, Darbar D, Smith NL, Smith JD, Schnabel R, Soliman EZ, Rice KM, Van Wagoner DR, Beckmann BM, van Noord C, Wang K, Ehret GB, Rotter JI, Hazen S, Steinbeck G, Makino S, Boerwinkle E, Milan DJ, Perz S, Kottgen A, Mobus S, Newton-Cheh C, Mohlenkamp S, Wang TJ, Ramachandran V, Nothen M, Macrae CA, Levy D, Nelis M, Metapalu A, Topol EJ, Psaty BM, Roden DM, Meitinger T, Wichmann HE, Witteman JC, Barnard J, Arking DE, Benjamin EJ, Heckbert SR, Kääb S. Common Variants in the Calcium Activated Potassium Channel, KCNN3, are Associated with Lone Atrial Fibrillation. **submitted** 2009.
29. Body SC, Collard CD, Shernan SK, Fox AA, Liu KY, Ritchie MD, Perry TE, Muehlschlegel JD, Aranki S, Donahue BS, Pretorius M, Estrada JC, Ellinor PT, Newton-Cheh C, Seidman CE, Seidman JG, Herman DS, Lichtner P, Meitinger T, Pfeufer A, Kaab S, Brown NJ, Roden DM, Darbar D. Variation in the 4q25 chromosomal locus predicts atrial fibrillation after coronary artery bypass graft surgery. **Circ Cardiovasc Genet** 2009;2:499-506.
30. Gretarsdottir S, Thorleifsson G, Manolescu A, Styrkarsdottir U, Helgadottir A, Gschwendtner A, Kostulas K, Kuhlenbaumer G, Bevan S, Jonsdottir T, Bjarnason H, Saemundsdottir J, Palsson S, Arnar DO, Holm H, Thorgeirsson G, Valdimarsson EM, Sveinbjornsdottir S, Gieger C, Berger K, Wichmann HE, Hillert J, Markus H, Gulcher JR, Ringelstein EB, Kong A, Dichgans M, Gudbjartsson DF, Thorsteinsdottir U, Stefansson K. Risk variants for atrial fibrillation on chromosome 4q25 associate with ischemic stroke. **Ann Neurol** 2008;64:402-409.
31. Husser D, Adams V, Piorkowski C, Hindricks G, Bollmann A. Chromosome 4q25 variants and atrial fibrillation recurrence after catheter ablation. **J Am Coll Cardiol** 2010;55:747-753.
32. Vaglio JC, Rowan SB, Stubblefield T, Carter S, Roden DM, Darbar D. Genetic and clinical predictors of response to rate control therapy in patients with atrial fibrillation. **Circulation** 2008;118:S828.
33. Rowan SB, Estrada JC, Stubblefield T, Kucera G, Carter S, Roden DM, Darbar D. A single nucleotide polymorphism at 4q25 associated with atrial fibrillation modulates symptomatic response to antiarrhythmic drug therapy. **Heart Rhythm** 2009;6:PO04-7.
34. Mommersteeg MTM, Brown NA, Prall OWJ, de Gier-de Vries C, Harvey RP, Moorman AFM, Christoffels VM. Pitx2c and Nkx2-5 Are Required for the Formation and Identity of the Pulmonary Myocardium. **Circ Res** 2007;101:902-909.
35. Wang J, Klysik E, Sood S, Johnson RL, Wehrens XH, Martin JF. Pitx2 prevents susceptibility to atrial arrhythmias by inhibiting left-sided pacemaker specification. **Proc Natl Acad Sci U S A** 2010;107:9753-9758.
36. Lubitz SA, Sinner MF, Lunetta KL, Makino S, Pfeufer A, Rahman R, Veltman CE, Barnard J, Bis JC, Danik SP, Sonni A, Shea MA, del Monte F, Perz S, Muller M, Peters A, Greenberg SM, Furie KL, van Noord C, Boerwinkle E, Stricker BHC, Witteman J, Smith JD, Chung MK, Heckbert SR, Benjamin EJ, Rosand J, Arking DE, Alonso A, Kaab S, Ellinor PT. Independent Susceptibility Markers for Atrial Fibrillation on Chromosome 4q25. **Circulation** 2010;122:976-984.

Appendix A: CABANA Inclusion / Exclusion Criteria

Inclusion Criteria

To be eligible for the trial, subjects must meet all of the following criteria:

1. Over the preceding **6** months have:
   a) ≥2 paroxysmal (***electrocardiographic documentation*** of at least 1) AF episodes lasting ≥1 hour in duration: (that terminate spontaneously within 7 days or cardioversion is performed within 48h of AF onset): or
   b) ***electrocardiographic documentation*** of 1 persistent AF episode: (sustained for ≥7 days or cardioversion is performed more than 48h after AF onset): or
   c) ***electrocardiographic documentation*** of 1 longstanding persistent AF episode: (continuous AF of duration >1 year).
2. Warrant active therapy (within the past 3 months) beyond simple ongoing observation
3. Be eligible for catheter ablation and ≥2 sequential rhythm control and/or ≥2 rate control drugs.
4. Be ≥65 yrs of age, or <65 yrs with **one or more** of the following risk factors for stroke: Hypertension (treated and/or defined as a BP >140/90 mmHg) [90], Diabetes (treated and/or defined as a fasting glucose ≥126 mg/dl) [91], Congestive heart failure (including systolic or diastolic heart failure), Prior stroke, TIA or systemic emboli, Atherosclerotic vascular disease (previous MI, peripheral arterial disease or aortic plaque), LA size >5.0 cm (or volume index ≥40 cc/m^2), or EF ≤35. Subjects <65 yrs of age whose only risk factor is hypertension must have a second risk factor or LV hypertrophy to qualify.
5. Have the capacity to understand and sign an informed consent form.
6. Be ≥18 years of age.

NOTE: Subjects may have recent onset AF (in the past 4-6 months), AF present for a longer time period, or may have been treated with a single anti-arrhythmic drug, providing they remain realistically eligible for ≥2 membrane active drugs and/or ≥2 rate control agents. Patients receiving new drug therapy initiated within the previous 3 months may continue that therapy if randomized to the drug therapy arm. Reasonable expectation of a response to therapy must be present. Subjects will not be excluded because of advancing age or underlying heart disease. Subjects with a history of a single episode of paroxysmal AF do not meet the "crossing the threshold/warranting therapy" litmus test. Subjects with persistent or long-standing persistent AF will require at least 1 documented episode, if it is of sufficient clinical importance that drug or ablative therapy is warranted. Subjects can be randomized before cardioversion, even if restoration of sinus rhythm is a desired endpoint of therapy. Patients may have documented atrial flutter in addition to atrial fibrillation and remain eligible for enrollment.

3.2 Exclusion Criteria

If a subject has ***any*** of the following criteria, he or she may not be enrolled in the study:

1. Lone AF in the absence of risk factors for stroke in patients <65 years of age
2. Patients who in the opinion of the managing clinician should not yet receive any therapy for AF
3. Patients who have failed ≥2 <u>*membrane active*</u> anti-arrhythmic drugs at a therapeutic dose due to inefficacy or side effects (Table 5.2.2)
4. An efficacy failure of full dose amiodarone treatment ≥8 weeks duration at any time
5. Reversible causes of AF including thyroid disorders, acute alcohol intoxication, recent major surgical procedures, or trauma
6. Recent cardiac events including MI, PCI, or valve or bypass surgery in the preceding 3 months

7. Hypertrophic obstructive cardiomyopathy (outflow track)
8. Class IV angina or Class IV CHF (including past or planned heart transplantation)
9. Other arrhythmias mandating anti-arrhythmic drug therapy (i.e. VT, VF)
10. Heritable arrhythmias or increased risk for torsade de pointes with class I or III drugs
11. Prior LA catheter ablation with the intention of treating AF
12. Prior surgical interventions for AF such as the MAZE procedure
13. Prior AV nodal ablation
14. Patients with other arrhythmias requiring ablative therapy
15. Contraindication to appropriate anti-coagulation therapy
16. Renal failure requiring dialysis
17. Medical conditions limiting expected survival to <1 year
18. Women of childbearing potential (unless post-menopausal or surgically sterile)
19. Participation in any other clinical mortality trial (Participation in other non-mortality trials should be reviewed with the clinical trial management center)
20. Unable to give informed consent

NOTE: Exclusion Criterion #3 includes failed membrane active antiarrhythmic drugs started within 3 months prior to enrollment. Prior ablation of the cavo-tricuspid isthmus alone is not an exclusion if the patient develops subsequent recurrent AF. Planned atrial flutter ablation in combination with the left atrial ablation is not an exclusion.

Appendix B: CABANAgene Kit Contents and Instructions

Patient Kit Contents

I. Blood Draw supplies

one pair (1 pair) disposable gloves
one (1) tourniquet
two (2) alcohol Swabs
one (1) #23 or 21 G Butterfly with tube holder
three (3) 5cc EDTA purple top Vacutainer tubes
one (1) gauze ball
one (1) band-aid

II. 3-Tube Mailing System

one (1) absorbent square
one (1) styrofoam container
one (1) strip of red sealing tape
one (1) biohazard bag
one (1) cardboard outer shipping container
one (1) UN 3373 Diagnostic Specimen label
one (1) FedEx ClinicalPak
one (1) FedEx Airbill with completed *ship to* information

III. Instructions with contact information

Instructions for Blood Draw and Shipping

Blood draw and shipping supplies included in the patient kit are listed above. Please draw blood Monday-Thursday and ship overnight via FedEx (or UPS) for arrival at Vanderbilt Tuesday-Friday am. Notify receiving lab by submitting subject sample(s) at website address

I. Blood Draw

1. Draw 3 purple top Vacutainer tubes (5cc each).
2. Mix the blood well with the anticoagulant by gently inverting the tube several times.
3. Label the tubes with the Sample ID. Please keep the samples at room temperature.

II. Shipping

1. Package samples by securely placing the blood tubes in the styrofoam container slots.
2. Place the absorbent pad on top of the blood tubes and close the container.
3. Peel off the protective strip from the red tape and seal the styrofoam container with the tape.
4. Place the sealed styrofoam container inside the biohazards bag and seal the bag.
5. Place the sealed biohazards bag inside the cardboard outer shipping container.
6. Affix the UN 3373 Diagnostic Specimen label to the outside of the cardboard shipping container.
7. Place the shipping container inside the FedEx Clinical Pak and seal the pack
8. Complete the FedEx Airbill, including date and sender information.
9. Ship overnight at room temperature via FedEx.
10. Notify receiving lab of shipment:
    Login to the member page at website address and *Submit Subject Sample(s)*

III. Ancillary Study Contact: Kris Norris, RN
Phone: 615-936-1131
Fax: 615-936-2222
E-mail: kris.norris@vanderbilt.edu

IV. Shipping Address: Vanderbilt University Medical Center
1266 MRB IV
Nashville, TN 37232-0575

Statistical Analysis Plan for CABANA

Analysis Plan dated February 26, 2018
Duke Clinical Research Institute

Protocol Date: November 22, 2013 (Version 3.5)
Sponsor: National Heart, Lung, and Blood Institute

Table of Contents

1. Study Title 3
2. Study Overview 3
3. Objectives 3
   3.1 Primary Objective 3
   3.2 Secondary Objectives 4
4. Subject Randomization 4
5. Primary Analysis Population 4
6. Data Sources 5
7. General Analysis Methodology 5
   7.1 Demographic and Baseline Analyses 6
   7.2 Disposition of Subjects 6
   7.3 Missing Data 6
8. Primary Endpoint Analysis 7
   8.1 Other Analyses of Primary Endpoint Data (as randomized): 8
9. Analysis of Secondary Endpoints 9
   9.1 All-cause mortality (key secondary endpoint) 9
   9.2 All-cause Mortality or Cardiovascular Hospitalization 10
   9.3 All-cause Mortality, Stroke, or Hospitalization for Heart Failure or Acute Ischemic Events 11
   9.4 Cardiovascular Death 11
   9.5 Cardiovascular Death or Disabling Stroke 12
   9.6 Arrhythmic Death or Cardiac Arrest 13
   9.7 Heart Failure Death 13
   9.8 Recurrent Atrial Fibrillation 14
   9.9 Cardiovascular Hospitalization 15
   9.10 Medical Costs, Resource Use and Cost Effectiveness. 17
   9.11 Quality of Life. 17
   9.12 Major Non-Endpoint Adverse Events 17
   9.13 LA Size, Morphology and Function. 17
10. "On-Treatment" Analysis 18
11. Per-Protocol Analysis 19
12. Multiple Comparisons 19
13. Interim Analyses 20
References 22

1. Study Title

<u>C</u>atheter **<u>Ab</u>**lation Versus **<u>An</u>**tiarrhythmic Drug Therapy for **<u>A</u>**trial Fibrillation (**CABANA**)

2. Study Overview

CABANA is a multi-center randomized clinical trial to assess the safety and efficacy of percutaneous left atrial catheter ablation versus antiarrhythmic drug therapy in subjects who are at least 18 years of age and have new onset or under-treated paroxysmal, persistent, or long-standing persistent atrial fibrillation (AF) and warrant therapy for their arrhythmia. The study population consists of 2,204 subjects enrolled at 126 clinical sites over a period of approximately six years. Subjects were randomized in equal proportions (1:1) to receive either catheter ablation or drug therapy and followed at regular intervals for the duration of the study. The minimum length of follow-up will be slightly less than 2 years, and the median duration of follow-up will be approximately 4 years. At study entry and during the follow-up period, extensive clinical, quality-of-life, and economic data are collected.

3. Objectives

3.1 Primary Objective

The primary objective of CABANA is to determine whether the treatment strategy of percutaneous left atrial catheter ablation for the purpose of eliminating atrial fibrillation will improve clinical outcomes compared to treating the arrhythmia with current state-of-the-art drug therapy consisting of either rate control or rhythm control drugs.

The **primary endpoint** is the time to the first event among a composite of major clinical outcomes consisting of

- Death (all-cause)
- Disabling stroke
- Serious bleeding
- Cardiac arrest

The primary hypothesis of the study is that the treatment strategy of percutaneous left atrial catheter ablation will reduce the incidence of this composite endpoint in patients with untreated or under-treated AF warranting therapy, compared to current state-of-the-art drug therapy.

3.2 Secondary Objectives

The secondary objectives are to compare the following clinical, economic, and quality-of-life outcomes in subjects randomized to catheter ablation versus state-of-the-art drug therapy:

1. **All-cause mortality (key secondary endpoint)**
2. All-cause mortality or cardiovascular hospitalization
3. All-cause mortality, stroke, or hospitalization for heart failure or for an acute ischemic event
4. Cardiovascular death
5. Cardiovascular death or disabling stroke
6. Arrhythmic death or cardiac arrest
7. Heart failure death
8. Recurrent atrial fibrillation
9. Cardiovascular hospitalization
10. Medical costs, resource utilization, and cost effectiveness
11. Health-related quality-of-life
12. Composite adverse events
13. Left atrial size, morphology, and function

To aid in the interpretation of the primary and several of the secondary endpoint comparisons, the individual components of the primary endpoint (in addition to death) will also be summarized between the randomized arms of the trial.

4. Subject Randomization

The treatment modalities being studied in CABANA are clinically-established treatment strategies that are used routinely across the U.S. and around the world. No experimental drug or treatment is involved. The trial intervention is simply the random assignment of the subjects to one or the other of the two treatment strategies.

Eligible subjects who provided written informed consent and met all inclusion and no exclusion criteria were randomly assigned in equal proportions (1:1) to either the catheter ablation arm or the drug arm using permuted blocks, stratified by clinical site. Subject randomization was accomplished by telephone through a centralized toll-free Interactive Voice Response System (IVRS).

5. Primary Analysis Population

Except where otherwise noted, all randomized subjects will be included in the analysis population for assessing the primary and secondary endpoints. All major treatment comparisons between the randomized groups in the trial will be performed according to the principle of "intention-to-treat" (ITT); that is, subjects will be analyzed and endpoints attributed according to the treatment arm to which the patients were randomized, regardless of subsequent crossover or

post-randomization medical care. As explained later in this document (section 10), some additional analyses will be conducted using “on-treatment” data.

6. Data Sources

The enrolling clinical sites complete a series of electronic Case Report Forms (eCRF) for each subject randomized, including demographic, clinical, and quality of life forms at enrollment and at specified follow-up intervals (the follow-up intervals are detailed in the study protocol), event forms for death, neurological events, bleeding, cardiac arrest, and other ancillary forms as required.

Clinical data are collected using the InForm electronic data capture (EDC) system. Except as noted below in section 9.8, rhythm data are collected using the “CABANA Box,” a rhythm monitoring system provided for each patient by Medicomp. The rhythm monitoring data are sent from Medicomp to the Duke Clinical Research Institute (DCRI), where a determination is made as to which rhythms should be sent to the ECG core lab in Seattle for review and adjudication. The core lab sends adjudicated results back to the DCRI. Sites with any subjects not using the “CABANA Box” submit rhythm data on those subjects through a secure portal using the CABANA sharepoint website, which is then processed and submitted to the ECG core lab for adjudication. CT/MRI data are received and processed by Mayo Clinic’s Imaging Analysis Laboratory, and the information is then transferred to the DCRI.

All data collected in the study are managed and analyzed at the CABANA Statistical and Data Coordinating Center at the DCRI. The InForm eCRF data are carefully reviewed, queried as needed, and then downloaded as raw SAS data files, and further review and checking of the data occur. The raw SAS data, analysis datasets, and analysis programs are stored on the secure DCRI statistical server. Final analyses will be performed at the DCRI using SAS version 9.4 or higher (SAS Institute Inc., Cary, NC). All programs written to create analysis datasets and perform analyses will be validated according to SOPs established by the DCRI Statistical Programming group.

7. General Analysis Methodology

As stated previously and except where otherwise noted, all major treatment comparisons will be performed according to the principle of "intention-to-treat.” Subjects will be analyzed and endpoints attributed according to the treatment arm to which patients were randomized, regardless of subsequent crossover or post-randomization medical care.

Appropriate statistical models and tests will be used to examine the effect of the treatment strategies on the primary and secondary endpoints. For time-to-first-event endpoints, the log-rank test[1] and Cox proportional hazards model[2] will be used for statistically comparing the treatment groups unless otherwise specified. Additional perspective regarding the interpretation of the data will be provided through extensive use of confidence intervals and graphical displays, including plots of Kaplan-Meier[3] event-rate estimates. All statistical comparisons will be

performed using two-sided significance tests and a nominal level of significance of $\alpha=0.05$ unless otherwise specified.

7.1. Demographic and Baseline Analyses

Descriptive summaries of the distribution of continuous baseline variables will be presented in terms of percentiles (median, 25th and 75th percentiles), while discrete variables will be summarized in terms of frequencies and percentages. Statistical comparisons of treatment groups with respect to baseline characteristics will be limited to selected variables and disease factors known to influence prognosis. These variables will include: age, sex, race, descriptors of comorbidity, heart failure class, type of AF (paroxysmal, persistent or permanent), years since onset of AF, days from most recent qualifying episode of atrial fibrillation to enrollment, presence/absence of structural heart disease, and CHA_2DS_2-VASc score. For comparisons of treatment groups with respect to continuous or ordinal baseline variables, the nonparametric Wilcoxon rank-sum test will be used. Group comparisons with respect to discrete baseline variables will use the Pearson's chi-square test or Fisher's exact test as appropriate.

7.2 Disposition of Subjects

The disposition of subjects (number randomized, number who received the randomly assigned treatment, number of treatment crossovers, number of patients who withdrew consent, and number lost to follow-up) will be summarized overall and by treatment arm. The summary of treatment crossovers will distinguish the patients randomized to the drug arm who subsequently underwent catheter ablation (crossed over to ablation) during the course of their follow-up and the patients randomized to the ablation arm who never received an ablation. The distribution of the length of follow-up for the overall study population and for each randomized arm will be characterized in terms of percentiles (median, 25th, and 75th percentiles). The number and percentage of patients who had at least 3 years of follow-up, and at least 5 years of follow-up will also be tabulated and reported. Among patients in the drug arm who crossed over to ablation, the distribution of length of follow-up prior to the crossover will be described in terms of percentiles. Also the distribution of follow-up among patients who withdrew consent (i.e., patients who withdrew prior to the protocol-specified 3 years of follow-up) will be summarized in terms of percentiles. Additionally, the number and percentage of patients who withdrew consent before 3 years, after 3 to <5 years, and after 5 years will be tabulated.

7.3 Missing Data

Missing data will be handled differently for covariates and endpoints. Data for covariates (see Section 8.1) are expected to be complete or near complete for all patients. Where missing data do occur, using single-imputation methods, continuous variables will be imputed using the median value and categorical variables will be imputed using the mode.

Most primary and secondary endpoints are time-to-event analyses which rely on the ability to calculate the time from randomization (or in some cases time from the end of the 90-day blanking period) to when the event occurred or the date of last contact for censored individuals. Here, missing data may occur when: 1) a visit form or question that indicates if an event occurred (or not) is not completed, and 2) a form or question indicates that an event occurred, but

the event date is missing. The first category prevents the assignment of whether an event occurred or not, while the second category prevents calculation of time to the event. In all analyses, we consider only non-missing data in the determination of whether or not an event occurred (i.e., cases in category 1 will not be considered). Time-to-event for known events when the event date is missing (category 2), will be assigned based on the visit with which the event is recorded. For example, if the event is reported on the month 3 visit, then the time to event would be set to 91.26 days (365/12 * 3). If the event is reported on the month 6 visit, then the time to event would be set to 182.52 days (365/12 * 6), and so on for all visits up to month 96. If patients do not experience an event, and have an event-free status associated with a visit but no visit date on their last known visit, they will be assigned a censor date in the same manner. For patients with multiple events including a death, any imputed non-death event dates (e.g., cardiovascular (CV) hospitalization) will be forced to occur before the date of death.

If an event or censored status is reported, but both the visit number and event/censored date are missing, then the date of event/censor will be imputed using relevant data and considered on a case-by-case basis.

8. Primary Endpoint Analysis

Primary Endpoint: Time from randomization to the first event among a composite of major clinical outcomes consisting of

- Death (all-cause)
- Disabling stroke
- Serious bleeding
- Cardiac arrest

All possible events are carefully reviewed and adjudicated by a Clinical Events Committee (CEC) completely blinded to the randomized treatment assignment.

Endpoint Definitions: Detailed definitions for each of the events are outlined in the CEC charter. We note that a disabling stroke during follow-up must be documented by a neurologic evaluation or brain CT / MR studies and result in a disabling, irreversible physical limitation defined by a Rankin Stroke Scale ≥ 2. Serious (or life-threatening) bleeding is considered to have occurred if intracranial bleeding or other bleeding occurs at any time during follow-up accompanied by hemodynamic compromise that requires surgical intervention or a transfusion of ≥ 3 units of blood. The serious bleeding definition *does not* include bleeding occurring with cardiac perforation or tamponade.

The time from randomization to the first event among the components of the primary composite endpoint will be measured (in days) for those who experienced an event and calculated as the date of the first event minus the date of randomization. For patients who do not experience any of the component events or who withdraw consent or drop out of the study before experiencing an event, time from randomization to the date of last contact will be used in the analysis, and those patients will be considered as censored observations in the time-to-event analysis. For

interim analyses, the best available endpoint data will be used (i.e., adjudicated data when available; otherwise site-reported event data).

Statistical Methods: The log-rank test[1] will be the primary analytic tool for statistically assessing outcome differences between the two randomized treatment strategies with respect to the primary composite endpoint. This analysis will be conducted using PROC LIFETEST in SAS. A hazard ratio (HR) and 95% confidence interval (CI) summarizing the difference in outcome between the two randomized arms will be computed using the Cox proportional hazards model[2] (PROC PHREG in SAS), using treatment as the only predictor in the model.

In addition to the statistical hypothesis testing, cumulative event rates will be calculated according to the method of Kaplan and Meier[3] for each randomized arm as a function of time from randomization, and the estimated event probabilities will be displayed graphically.

The methods described above will constitute the primary analysis of this endpoint. The primary hypothesis is defined as:

$$H_0: \quad S_a(t) = S_d(t), \text{ vs.}$$

$$H_a: \quad S_a(t) \neq S_d(t),$$

where $S_a(t)$ is the event-free survivor function for the ablation arm of the trial, and $S_d(t)$ is the event-free survivor function for the drug arm, both of which are a function of follow-up time t. In other words, we are testing whether the event-free survival experience of the two arms of the trial is the same vs. whether it is different. The above hypothesis will be tested at a 2-sided significance level of 0.049 in the ITT population to account for a single interim analysis (see Section 13).

Because the Cox model will be used for calculating a hazard ratio and confidence interval to summarize the difference in outcome between the two randomized arms, the proportionality assumption in the Cox model (i.e., constant hazard ratio over time) will be checked and tested through adding an interaction term (treatment*log(t)) to the Cox model, and by examining a plot of Schoenfeld residuals.

The hypothesis as expressed above will also be used for the time-to-event analyses of secondary endpoints described below using a 2-sided significance level of 0.05.

8.1 Other Analyses of Primary Endpoint Data (as randomized):

Treatment comparison with covariate adjustment. Using the Cox model, the effect of treatment strategy will be tested, adjusting for the following prospectively defined set of baseline patient characteristics that are expected to have a prognostic relationship with the primary endpoint: age (as a continuous variable), sex, minority status, baseline AF type, years since onset of AF, coronary artery disease, history of congestive heart failure, structural heart disease, hypertension, and CHA_2DS_2-VASc score. These characteristics will serve as a prelude to analyses for examining differential treatment effects. Structural heart disease will be defined as: history of valve disease with either surgical correction or percutaneous intervention, history of

cardiomyopathy, mitral valve regurgitation of moderate or greater severity, or mitral valve stenosis of moderate or greater severity.

Differential treatment effects. If there is an overall difference in outcome between treatment groups, supplementary analyses will be performed to assess whether the therapeutic effect is consistent across all patients, or whether it varies according to specific patient characteristics. In particular, these analyses will focus on whether the relative therapeutic effect of ablation compared to drug therapy differs according to the following baseline variables:

- Age (<65 , 65 to 74, and ≥75 years)
- Sex (male vs. female)
- Race (white vs. racial minorities)
- AF type (paroxysmal vs. persistent, or long-standing persistent)
- Years since onset of AF (>1 vs ≤1)
- Days from most recent qualifying episode of atrial fibrillation to enrollment (>12 vs ≤12 days)
- NYHA Heart Failure Class at enrollment (no heart failure or Class I vs. ≥ Class II)
- History of congestive heart failure (yes vs. no)
- Structural heart disease (present vs. absent)
- Hypertension (present vs. absent)
- Hypertension with LVH (present vs. absent)
- CHADS2 (0 or 1 vs >1)
- CHA_2DS_2-VASc score (0 or 1 vs >1)
- Sleep Apnea (present vs. absent)
- Family history of atrial fibrillation (yes vs. no)
- Obesity (BMI ≥30 vs. < 30)
- Left ventricular ejection fraction (LVEF) (≤35 vs >35)
- North American vs. other international sites

These analyses will utilize the Cox model and will be accomplished by testing for interactions between the randomized treatment strategy and the specific baseline variables listed above. In addition to the formal assessment of treatment by covariate interactions, the effect of the treatment strategy characterized by a hazard ratio and 95% confidence interval will be calculated and displayed using a forest plot for the subgroups of subjects defined by the variables listed above. These descriptive hazard ratios will be carefully interpreted in conjunction with the formal interaction tests.

The effect of the treatment strategy may also be examined in other subgroups of clinical interest in addition to those listed above.

9. Analysis of Secondary Endpoints

9.1 All-cause mortality (key secondary endpoint)

Endpoint Definition: Time from randomization until death.

For subjects who have not met this endpoint (and thus are censored observations), time from randomization until the subject's last follow-up contact will be used in the analysis.

Great effort will be made to gather mortality information on every randomized subject. For patients who withdraw consent or are lost to follow-up during the course of the trial, mortality databases such as the National Death Index, the Social Security Death Index, Ancestry.com, as well as any other sources of information or national registries, will be consulted in an effort to determine whether the subjects have died. Any subject found through one of these sources to have died will be considered to have met this key secondary endpoint even if not enough information is available for the CEC to classify the mode of death. Subjects for whom no vital status information can be found in these database searches will be censored at the time of their last follow-up contact.

Statistical Methods: The analysis of this key secondary endpoint will use the same methods as described for the primary endpoint in Section 8. Specifically, the log-rank test will be the primary analytic tool for statistically assessing mortality differences between the two randomized treatment strategies. A hazard ratio and 95% confidence interval summarizing the difference in outcome between the two randomized arms will be computed using the Cox model. The level of significance for the assessment of this endpoint will be $\alpha=0.05$. Also, Kaplan-Meier cumulative event rates will be calculated for each randomized arm as a function of time from randomization, and the estimated event probabilities will be displayed graphically.

Supplementary analyses of this secondary endpoint will be performed using the same methods described for the primary endpoint in Section 8.1.

9.2 All-cause Mortality or Cardiovascular Hospitalization

Endpoint definition: Time from randomization to the first event in this composite endpoint (i.e., time from randomization to either death or the subject's first cardiovascular hospitalization).

For subjects who have not experienced either component of this endpoint (i.e., who are censored observations), time from randomization until the subject's last follow-up contact will be used in the analysis.

The cause of hospitalization will be based on the *site-reported* data in the CABANA electronic data capture (EDC) system. For this endpoint, cardiovascular hospitalization will not include a hospitalization to initiate therapy. For example, among patients randomized to the ablation arm, the hospitalization to perform the ablation will not be counted for this secondary endpoint. Similarly, if a patient randomized to the drug arm is hospitalized to monitor the patient while loading the initial drug therapy, that hospitalization will not be counted. All other hospitalizations designated by the clinical site as *cardiovascular* will be counted, including the hospitalization of a patient randomized to the drug arm who is hospitalized for a therapy change (either a change to a different drug therapy or for crossing over to ablation). Hospitalization to perform second or subsequent ablation procedures will be counted as a CV hospitalization event.

Statistical Methods: The analysis of this secondary endpoint will use the same methods as described for the primary endpoint and key secondary endpoint in sections 8 and 9.1, including statistical testing using the log-rank test, calculation of a hazard ratio and 95% confidence interval using the Cox model, and Kaplan-Meier cumulative event rate calculations. The level of significance for the assessment of this endpoint will be α=0.05.

9.3 All-cause Mortality, Stroke, or Hospitalization for Heart Failure or an Acute Ischemic Event

Endpoint definition: Time from randomization to the first event in this composite endpoint.

For subjects who have not experienced any component of this endpoint (i.e., censored observations), time from randomization until the subject's last follow-up contact will be used in the analysis. The stroke component of this endpoint is defined the same as the stroke component of the primary endpoint, namely as a disabling stroke characterized by a Rankin Scale $\geq$ 2. The cause of hospitalization will be based on the *site-reported* data in the CABANA electronic data capture (EDC) system.

Statistical Methods: The analysis of this secondary endpoint will use the same methods as described for the primary endpoint and secondary endpoints in the sections above, including use of the log-rank test, hazard ratio and 95% confidence interval calculated using the Cox model, and Kaplan-Meier cumulative event rate calculations. The level of significance for the assessment of this endpoint will also be α=0.05.

This endpoint is similar to the first co-primary endpoint of the Early treatment of Atrial fibrillation for Stroke prevention Trial (EAST)[4] with the exception that EAST is using cardiovascular mortality rather than all-cause mortality as the death component of the endpoint and also a different definition of stroke (any stroke rather than a disabling stroke). Therefore, to provide results that can be compared to those of EAST, supplemental comparative analyses will be performed using cardiovascular mortality in this endpoint rather than all-cause mortality, and a definition of stroke that corresponds to the EAST definition. The statistical methods for these analyses will employ the competing risk methodology described in the next section for analyzing cardiovascular death, as well as the approach described in the preceding paragraph where all-cause mortality is a component of the endpoint.

9.4 Cardiovascular Death

Endpoint definition: Time from randomization to the occurrence of a cardiovascular (CV) death.

Patient deaths will be classified by the Clinical Events Committee (CEC) as to whether the mode of death was due to a cardiovascular (CV) cause. If insufficient source documents are obtained to allow CEC adjudication of the cause of death, and the CEC classifies the cause of death as "unknown," then the site-reported cause of death (if available) will be used. If neither the site nor the CEC can provide a classification of the cause of death, the death will not be considered as a cardiovascular death. As supplemental analyses, however, this endpoint will also be examined using (a) only the deaths classified by the CEC as cardiovascular, and (b) using deaths

classified by the CEC as cardiovascular, but also including any deaths in the cardiovascular category that are classified as unknown by the CEC.

Statistical Methods: The analysis of this endpoint will be performed using the competing risks methodology of Fine and Gray[5], where death due to a non-cardiovascular cause is considered as a competing risk. This methodology, rather than treating non-cardiovascular death as a censoring event, makes use of the cumulative incidence function, and is performed within the proportional hazards framework using the marginal failure sub-distribution associated with the event of interest (cardiovascular death). The features of the Fine-Gray approach for handling competing risks data are available as part of the PHREG procedure in SAS. The cumulative incidence function for the two arms will be estimated, statistically compared, and graphically presented using the PHREG software features in SAS. For subjects who have not died, time from randomization until the subject's last follow-up contact will be used in the analysis. The level of significance for the assessment of this endpoint will be $\alpha=0.05$.

Additional analysis of this secondary endpoint will be performed using the same methods as described for the primary endpoint and secondary endpoints in the sections above, including use of the log-rank test, hazard ratio and 95% confidence interval generated using the Cox model, and Kaplan-Meier cumulative event rate calculations. Patients who have died but whose cause of death is classified by the CEC as non-cardiovascular will be censored at the time of their non-CV death. The number of such patients is expected to be relatively small in this study. The results of these two different approaches will be carefully examined and synthesized into an overall interpretation of treatment differences with respect to CV death.

9.5 Cardiovascular Death or Disabling Stroke

Endpoint definition: Time from randomization to the occurrence of either a cardiovascular death or disabling stroke.

All potential strokes will be carefully reviewed and classified as disabling by the neurological events committee using the Rankin Stroke Scale as described in Section 8. The neurological committee that reviews and adjudicates the strokes is an important part of the overall clinical events adjudication process. A disabling stroke will be defined as a stroke whose severity on the Rankin Scale is ≥ 2. Cardiovascular deaths will be classified as described in Section 9.4 above.

Statistical Methods: The analysis of this secondary endpoint will also use the competing risks methodology of Fine and Gray[5] described in Section 9.4 for the secondary endpoint of CV death where death due to a non-cardiovascular cause is considered as a competing risk rather than a censoring event. The analysis will make use of the cumulative incidence function, and (as described in Section 9.4) will be performed within the proportional hazards framework using the marginal failure sub-distribution associated with the event of interest. The cumulative incidence function for the two arms will be estimated, statistically compared, and graphically presented using the PHREG procedure in SAS. For subjects who have not died, time from randomization until the subject's last follow-up contact will be used in the analysis. The level of significance for the assessment of this endpoint will be $\alpha=0.05$.

As in the case of the cardiovascular death endpoint, additional analysis of this secondary endpoint will be performed using the log-rank test, hazard ratio and 95% confidence interval generated using the Cox model, and Kaplan-Meier cumulative event rate calculations. Patients who have died but whose cause of death is classified by the CEC as non-cardiovascular will be censored at the time of their non-CV death. Results of these two different approaches will be carefully compared and synthesized into an overall interpretation of treatment differences with respect to the endpoint of CV death or disabling stroke.

9.6 Arrhythmic Death or Cardiac Arrest

Endpoint definition: Time from randomization to the occurrence of either an arrhythmic death or cardiac arrest.

Since cardiac arrest is a component of the primary endpoint, all potential cardiac arrests will be reviewed and adjudicated by the Clinical Endpoints Committee. As described earlier, the mode of death is also classified by the Clinical Endpoints Committee.

Statistical Methods: The analysis of this secondary endpoint will proceed in much the same way as described above for the endpoint of CV death or disabling stroke and use the competing risks methodology of Fine and Gray[5] described in previous sections. In this analysis, death due to a cause other than arrhythmic is considered as a competing risk rather than a censoring event. The analysis will make use of the cumulative incidence function, and will be performed within the proportional hazards framework using the marginal failure sub-distribution associated with the event of interest. The cumulative incidence function for the two arms will be estimated, statistically compared, and graphically presented using the features available in the PHREG procedure in SAS. For subjects who have not died, time from randomization until the subject's last follow-up contact will be used in the analysis. The level of significance for the assessment of this endpoint will be $\alpha=0.05$.

For this endpoint, we will also perform additional analysis using the log-rank test, hazard ratio and 95% confidence interval generated using the Cox model, and Kaplan-Meier cumulative event rate calculations. Patients who have died but whose cause of death is classified by the CEC as other than arrhythmic will be censored at the time of the non-arrhythmic death. The results of these two different approaches will be compared and synthesized into an overall interpretation of treatment differences with respect to the endpoint of arrhythmic death or cardiac arrest.

9.7 Heart Failure Death

Endpoint definition: Time from randomization to the occurrence of a heart failure death.

Classification of deaths as being due to heart failure will be performed by the blinded Clinical Endpoints Committee.

Statistical Methods: The analysis of this endpoint will make use of the competing risks methodology of Fine and Gray[5] described in previous sections for other secondary endpoints. In this case, death due to a cause other than heart failure will considered as a competing risk. The

features of the Fine-Gray approach for handling competing risks data available as part of the PHREG procedure in SAS will be employed to estimate the cumulative incidence function for each of the two arms, to statistically compare these functions, and graphically present them using the software features in SAS. The level of significance for the assessment of this endpoint will also be α=0.05.

Additional analysis of this secondary endpoint will also be performed using the log-rank test, hazard ratio and 95% confidence interval (CI) generated using the Cox model, and Kaplan-Meier cumulative event rate calculations. For this analysis, patients who have died, but their death is classified by the CEC as due to a cause other than heart failure, will be censored at the time of their non-heart failure death. The results of the competing risk analysis and of this traditional analysis will be compared to synthesize an overall interpretation of the treatment differences.

9.8 Recurrent Atrial Fibrillation

Endpoint definition: This particular outcome will be analyzed with different endpoint definitions in order to provide an overall perspective of the extent of the AF burden experienced by the study subjects. The first analysis will consider the first episode of recurrent AF as the endpoint, defined as an episode $\geq$30 seconds following the end of the 90-day blanking period, where the 90-day blanking period begins at the time of receiving the randomly assigned treatment (either ablation or drug therapy). The endpoint will be defined as the time until the first recurrence of AF according to this definition. Similar analyses will be performed where the endpoint is defined as: (a) an episode of *symptomatic* recurrent AF, (b) an episode of atrial fibrillation or atrial flutter or atrial tachycardia, and (c) an episode of *symptomatic* atrial fibrillation, atrial flutter, or atrial tachycardia.

Analysis Population: Because recurrent atrial fibrillation is defined with respect to the end of the blanking period, which begins after receipt of the assigned treatment, all analyses for recurrent atrial fibrillation will include only patients who have received their randomly assigned treatment and live to the end of the blanking period.

In addition, separate analyses will be performed according to the method used for monitoring AF recurrence. As indicated in Section 6 (Data Sources), rhythm data for many of the patients enrolled in CABANA (approximately 2/3 of the randomized patients) are collected using the "CABANA Box," a robust rhythm monitoring system provided by Medicomp. The rhythm monitoring data are sent from Medicomp to the Duke Clinical Research Institute (DCRI), where a set of programmed rules are run to determine which rhythms should be submitted to the ECG core lab for review and adjudication. Following core lab review, the adjudicated results are electronically transmitted back to the DCRI. Sites that are not using the "CABANA Box" (generally these are international sites where use of the CABANA Box was not approved) submit rhythm data through a secure portal using the CABANA website, which is then processed and submitted to the core lab for adjudication. Because the CABANA Box data are more comprehensive and more routinely and rigorously collected, separate analyses of recurrent atrial arrhythmias will be performed using (a) patients whose rhythms were monitored using the CABANA Box, (b) all remaining patients (i.e., non-CABANA Box patients), and (c) the pooled group of all patients (with use of CABANA Box as a covariate if justified). In the case of

patients who initially used the CABANA Box and later ceased to provide information via CABANA Box, the analysis will censor that data at the time of last CABANA Box recording. The primary analysis of this endpoint will be the analysis of patients with CABANA Box data because of the more comprehensive collection of that rhythm monitoring information. Analysis which includes non-CABANA Box patients ("b" and "c" above) will be considered supplementary analysis.

Statistical Analysis: First, descriptive summaries of baseline characteristics of subjects receiving their assigned treatment and living to the end of the blanking period in both treatment arms will be presented to determine how comparable the patients in the two arms are for this analysis. Continuous variables will be presented in terms of percentiles (median, 25th and 75th percentiles), while discrete variables will be summarized in terms of frequencies and percentages.

The treatment arms will be compared using statistical methods described in the sections above for other secondary endpoints, including the competing risk method of Fine and Gray[4], as well as use of the log-rank test, hazard ratio and 95% confidence interval generated using the Cox model, and Kaplan-Meier cumulative event rate calculations. In this case, all-cause mortality will be a competing risk and zero-time (i.e., when the clock starts) for this analysis will be the end of the 90-day blanking period. Analyses will be repeated with adjustment for the baseline covariates described in section 8.1.

Of key interest will be whether the effect of ablation on recurrent atrial arrhythmias is similar or different according to the type of AF the patients experienced prior to enrolling in the trial (i.e., whether the effect varies according to whether patients' baseline AF was paroxysmal, persistent or long-standing persistent). This analysis will utilize the Cox model to test for an interaction between the randomized treatment strategy and the baseline type of AF classified as (a) paroxysmal or (b) persistent or longstanding persistent. In addition to the interaction test, the effect of the treatment strategy characterized by a hazard ratio and 95% confidence interval and Kaplan-Meier event rate calculations will be calculated and displayed for the subgroups of patients whose baseline AF history was (a) paroxysmal and (b) persistent or longstanding persistent. Similar comparative analyses will be performed in subgroups defined by age (<65, 65-74, and $\geq$75) and by CHA_2DS_2-VASc Score (0 or 1 vs >1).

9.9 Cardiovascular Hospitalization

A key part of the analysis and interpretation of the results of this endpoint will be the analysis performed in connection with secondary endpoint #2 (the composite of death or cardiovascular hospitalization). Analysis of cardiovascular hospitalization as an endpoint by itself must be analyzed and interpreted cautiously, particularly if there is a mortality difference between the two arms of the trial. If patients in one arm have lower mortality, that arm may have more hospitalizations simply because more patients are alive and at risk for needing to be hospitalized. Another important aspect of the analysis of this endpoint is that patients may have multiple hospitalizations, and so a comprehensive analysis of hospitalizations must take into account the recurrent hospitalization events rather than simply the first one. Consequently, there will be

several parts to the analysis of this endpoint in addition to a careful examination of the results of the composite endpoint consisting of death or cardiovascular hospitalization (see Section 9.2).

Statistical Methods:

a. The first analysis will consider time until the first cardiovascular hospitalization in each arm and use the competing risks methodology of Fine and Gray[5] where death from any cause is considered as a competing risk. The analysis will make use of the cumulative incidence function, and will be performed within the proportional hazards framework using the approach described in Section 9.4. The cumulative incidence function for each of the two arms will be estimated, statistically compared, and graphically presented using the PHREG procedure in SAS. For subjects who have not experienced a CV hospitalization, time from randomization until the subject's last follow-up contact will be used in the analysis. The level of significance for the assessment of this endpoint will be α=0.05.

b. An additional analysis of this endpoint will be performed using the log-rank test, hazard ratio and 95% confidence interval generated using the Cox model, and Kaplan-Meier cumulative event rate calculations. Patients who have died will be censored at the time of their death. This analysis and the approach in (a) above will be compared and summarized into an overall interpretation of treatment differences with respect to the endpoint of time to the first CV hospitalization.

c. The analyses described above will be extended to a multiple events analysis, designed to take into account multiple hospitalization events that may occur during the course of a patient's follow-up. When considering one event per patient as in (a) or (b) above (i.e., the time until the first event), those events are independent because they occur in different patients. When considering multiple events for a patient, the events are no longer all independent because some of them are occurring within the same patient. This correlation (covariance) must be taken into account in deriving inferences. The multiple-event methods that will be used represent different extensions of Cox model methodology, and take into account the timing of the events and the correlation among multiple events occurring within an individual patient.

   The main statistical approach will use a generalization of the Cox model developed by Andersen & Gill[6,7], with robust standard errors to account for heterogeneity and for the correlation between recurrent events within a patient.

   Similar to time-to-1st event methods, a terminal event (death) could introduce bias in the recurrent events analysis, especially if there is a differential mortality between the study arms. Thus, the Ghosh and Lin[8] nonparametric method can be utilized in calculating the cumulative rate of CV hospitalizations, while accounting for mortality as a competing risk. In addition, the average number of CV hospitalizations per 100-person years utilizing the Ghosh-Lin method can be calculated for each treatment group.

   The Andersen-Gill recurrent events method will be performed using PROC PHREG in SAS software. However, the Ghosh-Lin method requires implementation of a validated package in R software.

9.10 Medical Costs, Resource Use and Cost Effectiveness.

Cost data will be analyzed by the CABANA EQOL Coordinating Center in close collaboration with the Data Coordinating Center, and the analysis approach will be detailed in a separate analytic plan.

9.11 Quality of Life.

Quality of life data will also be analyzed by the CABANA EQOL Coordinating Center in close collaboration with the Data Coordinating Center. Again, the analysis approach will be detailed in a separate analytic plan.

9.12 Major Non-Endpoint Adverse Events.

Major adverse events (AE) will be monitored and reported during the trial. For drug therapy, the major serious or life-threatening events include unanticipated hospitalizations for heart failure, drug-related pro-arrhythmic events, and pulmonary toxicity from amiodarone. For the ablation arm, the major events include symptomatic pulmonary vein stenosis requiring intervention, myocardial perforation/tamponade requiring intervention, and atrial-esophageal fistula formation. The frequency of each of these events (and other serious adverse events such as peripheral thromboembolic events) will be descriptively tabulated using MedDRA System Organ Class. Treatment comparisons with respect to these serious adverse events will be more descriptive rather than statistically rigorous (since different adverse events are expected in the two arms), bringing to bear clinical judgment as to the relative seriousness of these various adverse events. Other adverse complications or adverse events of either therapy will be tabulated and descriptively summarized, using statistical comparisons of the treatment groups based on chi-square tests where appropriate, and interpreting such comparisons in the context of treatment differences in the primary and major secondary clinical endpoints.

9.13 LA Size, Morphology and Function.

For the subjects participating in the CT/MR imaging sub-study, left atrial size, morphology, and function will be examined. Of interest will be the change in atrial size (volume) and function (i.e., atrial ejection fraction) from the pre-treatment to the post-treatment study within each treatment arm, and then importantly, a comparison of whether the change in size and function in the patients randomized to ablation therapy differs from the change in size and function of the patients randomized to drug therapy.

The distribution of these measures from the pre- and post-treatment studies in each treatment arm will be descriptively characterized using summary descriptive statistics and graphical tools including box and whisker plots. The statistical significance of the changes in left atrial volume and atrial ejection fraction from pre- to post-treatment studies within each treatment arm will be assessed using general linear models (GLM), including the baseline value as a covariate, and assessing treatment group differences. If there appears to be differences in other baseline

covariates between groups, adjustment for covariates will be considered. Secondary analyses will use a paired-sample t-test instead of GLM.

In dealing with this type of data, the completeness of which is dependent on obtaining both the pre- and post-treatment studies, there will inherently be missing data resulting from patients in whom it was not possible to obtain both studies (because they died or failed to return for other reasons to enable the follow-up measurements to be made). The completeness of the data will be fully described, and the statistical comparisons will be based on patients for whom both the baseline and follow-up studies are available.

10. "On-Treatment" Analysis

The intention-to-treat analyses in this trial will constitute the primary analyses and will serve as the standard for interpreting treatment differences in the key clinical outcomes. However, because a number of patients in the drug arm may cross over to receive ablation during the trial, and some patients randomized to ablation may not undergo the procedure, we will supplement the intent-to-treat comparisons of the primary endpoint and selected secondary endpoints with "on-treatment" comparisons. The "on-treatment" analysis will involve a comparison of patients who received ablation (even if originally assigned to the drug arm) versus those who did not. Statistically, this will be accomplished using the Cox proportional hazards model with ablation included in the analysis as a time-dependent covariate. This analysis will be repeated for the primary endpoint, all-cause mortality, all-cause mortality or cardiovascular hospitalization, all-cause mortality, stroke, or hospitalization for heart failure or acute ischemic events, cardiovascular death, cardiovascular death or disabling stroke, arrhythmic death or cardiac arrest, heart failure death, and cardiovascular hospitalization with and without adjustment for covariates listed in section 8.1. No analyses will include competing risks, and on-treatment analysis will not be conducted for multiple events analyses (section 9.9). For recurrent atrial fibrillation, we will only conduct this on-treatment analysis for the first episode of recurrent AF without competing risks in the 3 patient populations (Cabana box, non-cabana box, and pooled). Secondary analyses may consider other measures of recurrent atrial fibrillation (e.g., *symptomatic* recurrent AF, atrial fibrillation or atrial flutter or atrial tachycardia, and *symptomatic* atrial fibrillation, atrial flutter, or atrial tachycardia).

The time-dependent ablation variable to be included in the analysis will be created using 3 different methods: For method 1, the ablation variable starts as 0 (no ablation) for all patients. For patients who received ablation, the ablation variable is set to 1 at the time of ablation. In method 2, the ablation variable starts as 1 for all patients who were randomized to ablation and actually received ablation. For all other patients, the ablation variable is started as 0 and set to 1 at the time of ablation if they received ablation during the trial. Method 3 has the ablation variable started as 1 for patients who were randomized to ablation. For all other patients (i.e., randomized to drug), the ablation variable is started as 0 and set to 1 at time of ablation.

Among the 3 methods described above, method 2 will be considered the main statistical method in handling cross-overs via a time-dependent variable in Cox model. The other 2 methods are considered as sensitivity analyses.

Differential treatment effects for the primary endpoint and major secondary endpoints of interest using on-treatment analyses will also be conducted. Subgroups of interest will include those listed in section 8.1.

Because treatment assignment is no longer random, results can be biased by an association between the likelihood of treatment change and the risk of a clinical event (i.e., factors that make a patient more likely to cross over may also make them more likely to have an event). Thus, such analyses will be cautiously interpreted. Each of the analyses described in this section will be covariate adjusted using the covariates described in section 8.1 for the primary endpoint.

11. Per-Protocol Analysis

Using the methods described for the primary endpoint in Section 8, an analysis will also be performed to compare the primary endpoint and the key secondary clinical outcomes of the two treatment strategies among the subset of patients who fully satisfied the inclusion/exclusion criteria and received the treatment to which they were randomly allocated. This analysis will include patients randomized to the drug arm who were treated with drug therapy, and patients randomized to the ablation arm who underwent the ablation within 6 months following enrollment in the trial. The follow-up of drug-arm patients who crossed over to ablation will be censored at the time of the ablation. Patients randomized to the ablation arm who were not ablated within 6 months will be excluded from this analysis. Additional sensitivity analyses will be performed in which the window for ablation is varied to include a shorter interval (3 months) and a longer window (1 year).

The analyses outlined in Sections 10 and 11 will be strictly supplementary to the intent-to-treat analyses described earlier.

12. Multiple Comparisons

With the primary hypothesis and the various secondary endpoints that have been outlined, there is a multiplicity of analyses to be performed, which leads to an increased probability that at least one of the comparisons could be "significant" by chance. There are adjustments (e.g., based on the Bonferroni inequality) that could be used to preserve the overall type I error level. To attempt to adjust for the effects of the repeated significance testing that will occur as part of the interim monitoring (discussed below), plus adjust for the multiplicity of secondary endpoints, would require that very small significance levels be used for every comparison. The overall level of significance will be 0.049 for the assessment of the primary endpoint (see Section 13) and 0.05 for all secondary endpoints. However, to account in part for the multiplicity of comparisons involving secondary endpoints, we will be conservative in the interpretation of the analyses, taking into account the degree of significance, and looking for consistency across endpoints. The actual p-value for each comparison will be reported to aid in the overall interpretation. We have

also pre-specified the primary and secondary outcome variables to avoid over-interpretation of strictly exploratory comparisons.

13. Interim Analyses

For ethical reasons, an interim examination of key safety and endpoint data has been performed at regular intervals during the course of the trial. The primary objective of these analyses was to evaluate the accumulating data for an unacceptably high frequency of negative clinical outcomes in either of the treatment arms. In addition, the interim monitoring involved a review of the aggregated event rates, patient recruitment, compliance with the study protocol, status of data collection, and other factors which reflected the overall progress and integrity of the study. The results of the interim analyses and status reports were carefully and confidentially reviewed by an NHLBI-appointed Data and Safety Monitoring Board (DSMB).

The DSMB met at approximately 6-month intervals to review the accumulating data. Prior to each meeting, the Statistical and Data Coordinating Center (SDCC) at the DCRI conducted the desired statistical analyses and prepared a summary report for review by the DSMB. The extracted data files and analysis programs for each DSMB report were archived and maintained at the DCRI. Reports were presented describing the progress of patient enrollment, the rates of compliance with therapy, and the frequency of protocol violations. The SDCC also reported on the number (status) of data forms completed and the overall quality and completeness of the information.

In CABANA, a group sequential method similar to that proposed by O'Brien and Fleming[9] was used as a guide in interpreting interim analyses. This procedure required large critical values early in the study, but relaxed (i.e., decreased) the critical value as the trial progressed. Because of the conservatism early in the trial, the critical value at the final analysis is near the "nominal" critical value. The actual method used for the interim monitoring was the Lan and DeMets[10] group sequential method with monitoring boundaries based on a two-sided, symmetric O'Brien-Fleming type spending function. The Lan-DeMets approach only required specification of the rate at which the Type I error (which in this trial is $\alpha = 0.05$ for the primary endpoint) was "spent". The procedure allowed "spending" a portion of α at each interim analysis in such a way that at the end of the study, the total Type I error did not exceed 0.05. The specific formulation of the spending function is described in Chapter 5 of Proschan et al (formula 5.4)[11]. Since the number of looks and the increments between looks did not need to be predetermined, it allowed considerable flexibility in the monitoring process for accommodating comparative examinations of the data in response to concerns of the DSMB that may arise during the course of the trial.

The analytic approach used at the interim analyses for assessing treatment differences consisted of the time-to-event analysis methods described previously, except that interpretation of statistical significance associated with treatment comparisons of key study endpoints was guided using the group sequential monitoring boundaries. The critical value of the test statistic and the

corresponding p-value required for significance was presented so that significance could be assessed precisely.

Although the CABANA DSMB has met approximately twice each year, the board has seen interim treatment-specific primary endpoint data only one time during the course of the trial. At all other meetings, only overall (aggregated) event data have been presented to them. As a result of the single treatment-specific DSMB presentation, the overall level of significance for the final analysis of the primary endpoint has been calculated to be .049 (using the O'Brien-Fleming boundary).

References

1. Kalbfleisch JD, Prentice RL. *The Statistical Analysis of Failure Time Data.* 2nd edition. 2002, John Wiley & Sons, Inc. Hoboken, New Jersey.
2. Cox DR. Regression models and life-tables (with discussion). *J Royal Statist Soc B* 1972; 34:187-220.
3. Kaplan EL, Meier P. Nonparametric estimation from incomplete observations. *J Am Statist Assn* 1958; 53:457-481.
4. Kirchhof P, Breithardt G, Camm AJ, Crijns HJ, Kuck K-H, Vardas P, Wegscheider K. Improving outcomes in patients with atrial fibrillation: Rationale and design of the Early treatment of Atrial fibrillation for Stroke prevention Trial. *Am Heart J* 2013; 166:442-448.
5. Fine JP, Gray RJ. A proportional hazards model for the subdistribution of a competing risk. *J Am Statist Assoc* 1999; 94:496-509.
6. Anderson PK, Gill RD. Cox's regression model for counting processes: a large sample study. *Ann Stat* 1982; 10:1100-1120.
7. Lin DY, Wei LJ. The robust inference for the Cox proportional hazards model. *J Am Stat Assoc* 1989; 84:1074-1078.
8. Ghosh D, Lin DY. Nonparametric analysis of recurrent events and death. *Biometrics* 2000; 56:554-62.
9. O'Brien PC, Fleming TR. A multiple testing procedure for clinical trials. *Biometrics* 1979; 35:549-56.
10. Lan KK, DeMets L. Discrete sequential boundaries for clinical trials. *Biometrika* 1983; 70:659-63.
11. Proschan MA, Lan KKG, Wittes JT. *Statistical Monitoring of Clinical Trials – A Unified Approach*. 2006

Summary of Changes in the Protocol

The following changes listed in Table 1 represent clarification, version control, and corrective changes to the CABANA Protocol, Version 3.1, (August 19, 2009). These changes were approved by the CABANA Leadership Team at Mayo Clinic and Duke Clinical Research Institute (DCRI).

Table 1. Summary of Protocol Changes

Section/Page	Version 3.0 June 19, 2009	Version 3.1 August 19, 2009	Rationale
Title Page	NA	**Grant Number:** 1U01HL089709-01A1<br>**Grant Number:** 1U01HL089786-01A1<br>**Grant Number:** 1U01HL089907-01A1<br>**Grant Number:** 1U01HL089645-01A1	Clarification<br>**No change to study procedures.**
Title Page	Version: 3.0	Version: 3.1	Version control<br>**No change to study procedures.**
Title Page	Date: June 19, 2009	Date: August 19, 2009	Version control<br>**No change to study procedures.**
Footer Throughout	June 19, 2009	August 19, 2009	Version control<br>**No change to study procedures.**
Page 6	CRF 312.64B	CFR 812 (Subpart E).	Correction<br>**No change to study procedures.**
4.4.3/24	Symptom Event Recorder	**CABANA Box**<br>Symptom Event Recorder	Clarification<br>**No change to study procedures.**
8.2.8/41	NA	Bang and Tsiatis	Correction<br>**No change in study procedures.**

Summary of Changes in the Protocol

The following changes listed in Table 1 represent clarification, version control, and corrective changes to the CABANA Protocol, Version 3.2, (24 November, 2009). These changes were approved by the CABANA Leadership Team at Mayo Clinic and Duke Clinical Research Institute (DCRI).

Table 1. Summary of Protocol Changes

Section/Page	Version 3.1 19 August, 2009	Version 3.2 24 November, 2009	Rationale
Title Page / 1	Version: 3.1	Version: 3.2	Version control<br>**No change to study procedures.**
Title Page / 1	Date: 19 August, 2009	Date: 24 November, 2009	Version control<br>**No change to study procedures.**
Footer Throughout	19 August, 2009	24 November, 2009	Version control<br>**No change to study procedures.**
Contacts / 2	Not previously listed	**National Heart Lung and Blood Institute**<br><br>NHLBI<br>Two Rockledge Centre<br>Suite 8170, MSC 7940<br>6701 Rockledge Dr.<br>Bethesda, MD 20892-7940<br>(Fed-Ex Zipcode 20817)<br><br>**Project Officer:** Alice M. Mascette, MD<br>Phone:301-435-0504<br>Fax: 301-480-7404<br>mascetta@mail.nih.gov	Clarification<br>**No change to study procedures.**
Contacts / 3	Phone: 507-284-4937<br><br>Not previously listed	Phone: 507-284-2997<br><br>**Co-Investigator:** David Holmes, III PhD<br>Phone: 507-284-2997	Clarification<br>**No change to study procedures.**

		Fax: 507-284-1632<br>Holmes.david3@mayo.edu<br><br>**Data Management**: Maryam Rettmann, PhD<br>Phone: 507-284-2997<br>Fax: 507-284-1632<br>rettmann.maryam@mayo.edu	
TOC / 8	Version 3.1	Updated to version 3.2	Clarification<br>**No change to study procedures.**
4.2 / 23	Baseline economic, functional status, and quality of life data, including SF-36, DASI, Toronto Atrial Fibrillation Severity Scale, and the AF Symptom Checklist will be collected via a structured questionnaire interview conducted by the Site Coordinator prior to randomization.	Baseline functional status, *economic data (including two EQ-5D forms that rate the patient's health by using a 0-100 "thermometer" and asking 5 brief questions)*, and quality of life data *(including a Baseline Questionnaire of validated scales, ie, SF-36, DASI, Toronto Atrial Fibrillation Severity Scale, AF Effects on QOL (AFEQT), Work Productivity and Activity Impairment Instrument (WPAI), and Stanford Presenteeism scale)* will be collected by the Site Coordinator *via structured interview* prior to randomization.	Clarification and to provide outcomes for secondary endpoints and objectives:<br>9. Medical costs, resource utilization, and cost effectiveness<br>10. Quality of Life
4.4.1 / 24	1. Historical examination including Drug History assessment<br>2. Physical examination<br>3. ECG<br>4. Blood Tests (INR, creatinine, hemoglobin, hematocrit)<br>5. 24-hour Holter monitoring<br>6. Trans-thoracic 2-D echocardiography<br>7. Trans-esophageal	*1.* Historical examination including Drug History assessment *(within 60 days prior to randomization)*<br>*2.* Physical examination *(within 60 days prior to randomization)*<br>*3.* ECG *(within 60 days prior to randomization)*<br>*4.* Blood Tests (INR, creatinine, hemoglobin, hematocrit) *(within 7 days prior to treatment)*<br>*5.* 24-hour Holter monitoring *(within 12*	Clarification<br>**No change to study procedures.**

	echocardiogram<br>8. CT /MR evaluation (all ablation patients and 375 Drug patients)<br>9. Economics and Quality of Life Assessment	*months prior to randomization*)<br>*6.* Trans-thoracic 2-D echocardiography *(within 4 months prior to randomization)*<br>*7.* Trans-esophageal echocardiogram *(within 48 hours prior to treatment)*<br>8. CT /MR evaluation (all ablation patients and 375 Drug patients) *(within 4 months prior to treatment)*<br>9. Economics and Quality of Life Assessment *(after consent, prior to randomization)*	
4.4.2 / 24	Follow-up in all patients will occur at 3, 6, and 12 months following randomization during the first year and every 6 months thereafter, with clinic visits, phone follow-up, and other testing as described below. Economic and QOL data, including SF-36, DASI, Toronto Atrial Fibrillation Severity Scale, the AF Symptom Checklist, Cardiac Self-Efficacy and Stanford Presenteeism scale will be repeated during the 3 month follow-up visit and annually by trained telephone interviewer staff from the EQOL Coordinating Center (EQOL CC) for patients enrolled in North America and by the Site Coordinator in sites outside North America. A Brief Follow-up Questionnaire capturing atrial fibrillation severity and symptoms will be collected at 6, 18, 30 and 42 months.	Follow-up in all patients will occur at 3, 6, and 12 months following randomization during the first year and every 6 months thereafter, with clinic visits, phone follow-up, and other testing as described below. Economic and QOL data, including *a full follow-up questionnaire of validated scales, ie,* SF-36, DASI, Toronto Atrial Fibrillation Severity Scale, AF *Effects on QOL (AFEQT), Work Productivity and Activity Impairment Instrument (WPAI),* and Stanford Presenteeism scale, will be repeated *at 3 months* and annually *from randomization* by trained telephone interviewer staff from the EQOL Coordinating Center (EQOL CC) for patients enrolled in North America and by the Site Coordinator in sites outside North America. A Brief Follow-up Questionnaire capturing atrial fibrillation severity and symptoms, *work productivity/activity/presenteeism* will be collected at 6, 18, 30 and 42 months	Clarification and to provide outcomes for secondary endpoints and objectives:<br>9. Medical costs, resource utilization, and cost effectiveness<br>10. Quality of Life

	Medical bills for patients enrolled at US sites will be collected throughout the trial by the EQOL Coordinating Center economic team. The Site Coordinators will complete a one page Rapid Report Form (RRF) at each CABANA study visit documenting any interim all-cause hospitalizations, ER visits since last contact and will forward them to the EQOL Coordinating Center for processing.	*post randomization. All above follow-up visits/calls should be completed within 30 days +/- of the due date. (Ex: 3 month visit: completed between 60 days and 120 days of randomization)*<br><br>Medical bills for patients enrolled at US sites will be collected throughout the trial by the EQOL Coordinating Center economic team. The Site Coordinators will complete a one page Rapid Report Form (RRF) at each CABANA study visit documenting any interim all-cause hospitalizations, ER visits since last contact and will forward them to the EQOL Coordinating Center for processing. *As part of the economic data in CABANA, two EQ-5D forms that rate the patient's health by using a 0-100 "thermometer" and asking 5 brief questions will be collected by the Site Coordinator throughout the trial and entered into the e-CRF.*	
4.4.3 / 26	Not previously listed	*5): Hospital discharge or following Drug Therapy initiation*	Clarification<br>**No change to study procedures.**
5.2.2 / 27	Tambacor<br>Tykosin<br>Quini-glute	*Tambocor*<br>*Tikosyn*<br>*Quinaglute/ dex*	Clarification<br>**No change to study procedures.**
5.2.2 / 27	Not previously listed	*Multaq (dronedarone) should not be used in patients with NYHA IV heart failure, or NYHA Class II-III heart failure with a recent decompensation requiring hospitalization or referral to a specialized heart failure clinic.*	Clarification in response to FDA letter dated October 14, 2009.<br>**No change to study procedures.**
6.2 / 32	Secondary endpoint events, including composite endpoints of	Relocated this statement as a stand-alone paragraph for clarity:	Clarification<br>**No change to study procedures.**

	mortality, disabling stroke, serious bleeding, or cardiac arrest will be confirmed and adjudicated in a similar manner by the CEC. A Disabling Stroke will be considered present using a modification of A Rankin Stroke score [100], and will be adjudicated by a Neurologic Events Committee. Serious (or Life-threatening) bleeding will be considered present using a modification of the GUSTO bleeding Scale adapted for use in catheter ablation [101]. These events will be tracked regardless of treatment randomization. While hospitalization for AF in both treatment arms will be carefully tracked and compared, it will be considered as an "*AF recurrence"* and counted against efficacy, not safety. The definition for each of these events is listed in the CEC Charter. When there is disagreement between the CEC and the principal investigator, the CEC's decision will be considered final. Procedures for adjudicating events are described in the CEC Charter, which is available upon request.	*While hospitalization for AF in both treatment arms will be carefully tracked and compared, it will be considered as an "AF recurrence" and counted against efficacy, not safety. Therefore, AF recurrence and/or worsening of AF does not need to be reported as an adverse event.*	
11.1.2 / 45	Any site may be terminated from the trial if it fails to comply with the above requirements. Specifically,	Any *activated* site may be terminated from the trial *for failure to enroll any subjects, within a reasonable period of*	Clarification requested by the DSMB. **No change to study procedures.**

	any site consistently failing to provide timely reports or inadequate data quality will be withdrawn from active enrollment. A cross-over rate >10% over any 6 month period will result in a warning to that site. A crossover rate >15% will prompt suspension of enrollment. A 3-month period for resolving any operational difficulties will be required prior to reinstating or permanently terminating a specific site to further enrollment. All patients randomized must be followed until death or the end of the trial.	*time. Also, any site may be suspended from active enrollment,* if it fails *to meet reasonable enrollment goals or* comply with *study procedures*. Specifically, any site consistently failing to provide timely reports or adequate data quality will be withdrawn from active enrollment. A cross-over rate >10% over any 6 month period will result in a warning to that site. A crossover rate >15% will prompt suspension of enrollment. A 3-month period for resolving any operational difficulties will be required prior to reinstating or permanently terminating a specific site to further enrollment. All *subjects* randomized must be followed until death or the end of the trial, *even if the site has been inactivated due to enrollment or study compliance.*	

Summary of Changes in the Protocol

The following changes listed in Table 1 represent clarification, version control, and corrective changes to the CABANA Protocol, Version 3.3, (03 March, 2010). These changes were approved by the CABANA Leadership Team at Mayo Clinic and Duke Clinical Research Institute (DCRI).

Table 1. Summary of Protocol Changes

Section/Page	Version 3.2 24 November, 2009	Version 3.3 03 March, 2010	Rationale
Title Page / 1	Version: 3.2	Version: 3.3	Version control<br>**No change to study procedures.**
Title Page / 1	Date: 24 November, 2009	Date: 03 March, 2010	Version control<br>**No change to study procedures.**
Footer Throughout	24 November, 2009	03 March, 2010	Version control<br>**No change to study procedures.**
TOC / 8	Version 3.2	Updated to version 3.3	Clarification<br>**No change to study procedures.**
4.4.1 / 24	All patients will undergo standard baseline evaluation, including:<br>1. Historical examination including Drug History assessment (within 60 days prior to randomization)<br>2. Physical examination (within 60 days prior to randomization)<br>3. ECG (within 60 days prior to randomization)<br>4. Blood Tests (INR, creatinine, hemoglobin, hematocrit) (within 7 days prior to treatment)<br>5. 24-hour Holter monitoring (within 12 months prior to treatment)<br>6. Trans-thoracic 2-D echocardiography (within 4 months prior to randomization)<br>7. Trans-esophageal echocardiogram (within 48 hours prior to treatment)	Defining the eligibility of patients for the CABANA Trial will require information generated during the course of routine clinical care as dictated by their attending physician. This information should be, consistent with established guidelines, consensus documents, and good clinical practice. Selected baseline testing data will be collected in order to characterize the type, cause and severity of the patient's AF and the treatments received prior to enrollment. The baseline data will include information from the following clinical evaluations:<br>1. Relevant medical history including prior and current drug treatment of AF	FDA requested<br>Clarification<br>Delineation between the baseline testing considered part of routine clinical care from that specifically required for the CABANA Trial

	8. CT /MR evaluation (all ablation patients and 375 Drug patients) (within 4 months prior to treatment)<br>9. Economics and Quality of Life Assessment (after consent, prior to randomization)<br><br>The trans-thoracic echocardiographic (TTE) studies are designed to characterize the substrate underlying the patient's AF, establishing the presence of LV dysfunction (LVEF), hypertrophy, diastolic dysfunction, or other structural abnormalities. The TTE will also assess LA size and volume, as well as echo measures of atrial function. Trans-esophageal echocardiographic (TEE) studies are designed to exclude the presence of intra-atrial thrombus as required prior to chemical or direct current cardioversion or ablative therapy. TEE data may also be used to confirm atrial size and morphology data. Information from all baseline testing will be entered into the Electronic Data Collection (EDC) system in specified eCRF fields. CT / MR studies will be undertaken in all ablation patients to serve as a baseline for quantitative LA size, morphology, and function studies, as well as PV and esophageal investigations. In addition, 375 patients randomized to drug therapy will undergo baseline scanning for comparisons in the atrial structure and function studies. These patients will be recruited from centers that are	2. Relevant physical examination<br>3. 12 lead ECG prior to treatment<br>4. Blood Tests (INR, creatinine, hemoglobin / hematocrit) (pre-treatment)<br>5. Trans-thoracic 2-D echocardiography<br><br>The trans-thoracic echocardiographic (TTE) data will be used to characterize the substrate underlying the patient's AF, establishing the presence of LV dysfunction (LVEF), hypertrophy, diastolic dysfunction, or other structural abnormalities. The TTE will also assess LA size and volume, and provide measures of atrial function.<br><br>Following a recommended approach consistent with established guidelines, consensus documents, and good clinical practice, a trans-esophageal echocardiographic (TEE) study will be performed within 24 hours prior to ablation in patients with *persistent or longstanding persistent* AF. The TEE may be performed up to 48 hours before the procedure in patients on continuous warfarin at a therapeutic INR or in those appropriately bridged with intravenous un-fractionated or low molecular weight heparin.<br><br>The performance of a pre-treatment TEE in patients with *paroxysmal* AF is left to the discretion of the investigator (5.4.4), but is not required in CABANA. Data on the performance	

	committed to CT / MR assessment of each enrolled drug patient. Since randomization is performed (stratified) within each site, this component of the study will still benefit from the overall study randomization.	and results of all TEEs performed as part of routine care will be collected in the eCRF, however. TEE data may also be used to confirm atrial size and morphology. The approach to cardioversion in drug treated patients should follow the recommendations of AF Treatment Guidelines [92].<br><br>In those centers where routine clinical practice includes the performance of pre-ablation CT / MR studies, relevant data from these studies will be collected to serve as a baseline for comparative quantitative LA size, morphology, and function studies, as well as subsequent follow-up PV and esophageal investigations. In addition at those centers, up to 375 patients randomized to drug therapy will be asked to undergo baseline research CT/MR scanning to allow the CABANA study to evaluate and compare atrial structure and function in response to drug or ablative therapy. These drug-treated patients will be recruited from selected centers that are committed to CT / MR assessment of enrolled drug patients. Since randomization is performed (stratified) within each site, this component of the study will still benefit from the overall study randomization. Optimally, the scans performed in all patients should be within 4 months prior to treatment.<br><br>In addition to these clinically dictated studies, baseline economic and QOL	

		data will be obtained after informed consent is obtained, but before randomization occurs.	
4.4.2 / 25<br>para 2	Medical bills for patients enrolled at US sites will be collected throughout the trial by the EQOL Coordinating Center economic team. The Site Coordinators will complete a one page Rapid Report Form (RRF) at each CABANA study visit documenting any interim all-cause hospitalizations, ER visits since last contact and will forward them to the EQOL Coordinating Center for processing. As part of the economic data in CABANA, two EQ-5D forms that rate the patient's health by using a 0-100 "thermometer" and asking 5 brief questions will be collected by the Site Coordinator throughout the trial and entered into the e-CRF.	Medical bills for patients enrolled at US sites will be collected throughout the trial by the EQOL Coordinating Center economic team. The Site Coordinators will complete a one page Rapid Report Form (RRF) at each CABANA study visit documenting any interim hospitalizations and/or, ER visits since last contact. These forms will be forwarded to the EQOL Coordinating Center for processing. As part of the economic data in CABANA, two EQ-5D forms that rate the patient's health by using a 0-100 "thermometer" and asking 5 brief questions will be collected by the Site Coordinator at each follow-up visit throughout the trial and entered into the e-CRF.	Clarification<br>Delineation between the baseline testing considered part of routine clinical care from that specifically required for the CABANA Trial
para 3	…(throughout trial)	… (throughout the trial)	
para 4	CT/MR imaging studies will be performed on all ablation subjects at baseline, 3 months post-ablation therapy, and as indicated for PV stenosis management throughout the trial. CT/MR imaging studies will also be performed on 375 drug therapy patients at baseline and 3 months after therapy is fully established (end of blanking period). It is essential that the same imaging modality, acquisition parameters, and imaging techniques be utilized for each subject at all time-points. CT/MR data will	CT/MR imaging studies will be performed where clinically indicated or otherwise part of routine clinical care on ablation subjects at baseline, at 3 months post-ablation therapy, and as indicated for PV stenosis management throughout the trial. At sites where baseline and follow-up CT/MR imaging studies are part of routine care for ablation patients, CT/MR imaging studies will also be performed for research purposes on up to 375 drug therapy patients at baseline and 3 months after therapy is	

	then be electronically transferred to a server at the Mayo Biomedical Imaging Resource, the CABANA Trial Image Analysis Lab. Scans will be anonymized at the site using a software tool provided by the Imaging Center. Each clinical center is required to perform standard site radiology evaluation and assessment of the CT/MR images according to the sites' standard clinical practice.	fully established (end of blanking period). CT/MR data will be electronically transferred to a server at the Mayo Biomedical Imaging Resource, the CABANA Trial Image Analysis Lab. Scans will be anonymized at the site using a software tool provided by the Imaging Center. Each clinical center will also perform standard site radiology evaluation and assessment of the CT/MR images according to the sites' standard clinical practice.	
4.4.3/26	All procedures and laboratory tests required for evaluation of follow-up should be performed whether or not a subject receives treatment according to the protocol.	All procedures and laboratory tests during follow-up should be performed whether or not a subject receives treatment according to the protocol.	Clarification<br>Delineation of testing considered part of routine clinical care from that required for the CABANA Trial
7.1.5 / 34	1. **Device-related:** any adverse event for which a causal relationship between the device and the event is a reasonable possibility. The likelihood that the event is device related will be categorized as listed above, the device-related "cause" will categorized as follows:	1. **Device-related:** any adverse event for which a causal relationship between the device and the event is a reasonable possibility. The likelihood that the event is device related will also be sub-classified using the approach in 7.1.4 above. The event will be further classified as a device failure or malfunction (7.1.5.2) and whether it is unanticipated (7.1.5.3), as described in these sections.	FDA requested Clarification<br>**No change to study procedures.**
Appendix B /60	P95005	P950005	Clarification/Typo<br>**No change to study procedures.**

Summary of Changes in the Protocol

The following changes listed in Table 1 represent clarification, version control, and corrective changes to the CABANA Protocol, Version 3.3, (03 March, 2010). These changes were approved by the CABANA Leadership Team at Mayo Clinic and Duke Clinical Research Institute (DCRI).

Table 1. Summary of Protocol Changes

**Sites/Protocol version**	**North America and International**	**North America and International**	**International with full Clinical Trial Application (CTA)**	
**Section/Page**	**Version 3.3 03 March, 2010**	**Version 3.4 08 August, 2011**	**Version 3.4.1 08 April, 2012**	**Rationale for change**
Title Page / 1	Version: 3.3	Version: 3.4	Version: 3.4.1	Language revisions/insert for compliance with US /Foreign Regulatory Authorities
Title Page / 1	Date: 03 March, 2010	Date: 08 August, 2011	Date: 08 April, 2012	
Title Page / 1			EudraCT-Number: 2011-002532-12	BfArM required
Footer Throughout	03 March, 2010	08 August, 2011	08 April, 2012	
Contact / 2	**Project Officer:**<br>Alice M. Mascette, MD<br>Phone:301-435-0504<br>Fax: 301-480-7404<br>mascetta@mail.nih.gov	**Project Officer:**<br>Alice M. Mascette, MD<br>Phone:301-435-0477<br>Fax: 301-480-7971<br>alice.mascetta@nih.gov	No revision from 3.4	Change of contact details
Contact / 2	**Project Leader**:<br>Kathleen L. Hoffmann, R.N.<br>Phone: 919-668-8277<br>Cell: 919-818-9461<br>Fax: 919-668-7105<br>kathleen.hoffmann@duke.edu	**Project Leader**:<br>Kathleen L. Moretz, R.N.<br>Phone: 919-668-8277<br>Cell: 919-818-9461<br>Fax: 919-668-7105<br>kathleen.moretz@duke.edu	No revision from 3.4	Change of contact details
Synopsis / 5	Enrollment will occur over approximately 3 years, and subjects will be followed a	Enrollment will occur over approximately 4 years, and subjects will be followed a	No revision from 3.4	The enrollment period has been extended

	minimum of 2 years.	minimum of 2 years.		
TOC / 8	Version 3.3	Updated to version 3.4	Updated to version 3.4.1	Change of protocol version
2.2.2 / 20	By reducing the recurrence of AF, the proposed therapies should also reduce all cause and cardiovascular hospitalization.	By reducing the recurrence of AF, the proposed therapies should also reduce cardiovascular hospitalization.	No revision from 3.4	Editorial change to increase the clarity of the text
3.1 / 21	1. Have documented AF episodes ≥1 hour in duration; with ≥2 episodes over 4 months with electrocardiographic documentation of 1 episode or at least 1 episode of AF lasting more than 1 week<br>4. Be ≥65 yrs of age, or <65 yrs with one or more of the following risk factors for stroke: Hypertension defined as a BP >140/90 mmHg [90], Diabetes defined as a fasting glucose ≥126 mg/dl [91], Congestive heart failure (including systolic or diastolic heart failure), Prior stroke or TIA, LA size >5.0 cm (or volume index ≥ 40 cc/m2), or EF ≤35. Subjects <65 yrs of age whose only risk factor is hypertension must have a second risk factor or LV hypertrophy to qualify.	1. Have paroxysmal AF episodes ≥1 hour in duration; with ≥2 episodes over the preceding 6 months with ***electrocardiographic documentation*** of at least 1 episode; or 1 persistent or longstanding persistent episode of AF lasting more than 1 week.<br>4. Be ≥65 yrs of age, or <65 yrs with **one or more** of the following risk factors for stroke: Hypertension (treated and/or defined as a BP >140/90 mmHg) [90], Diabetes (treated and/or defined as a fasting glucose ≥126 mg/dl) [91], Congestive heart failure (including systolic or diastolic heart failure), Prior stroke or TIA, LA size >5.0 cm (or volume index ≥40 cc/m2), or EF ≤35. Subjects <65 yrs of age whose only risk factor is hypertension must have a second risk factor or LV hypertrophy to qualify.		Clarification of the inclusion criteria
3.1 / 22		Patients may have documented atrial flutter in addition to atrial fibrillation and remain eligible	No revision from 3.4	Wording added for clarification that documented atrial

		for enrollment.		flutter is accepted along with atrial fibrillation
3.2 / 22	1. Patients who have failed >2 membrane active anti-arrhythmic drugs at a therapeutic dose due to inefficacy or side effects<br>8. Hypertrophic obstructive cardiomyopathy<br>10. Other mandated anti-arrhythmic drug therapy<br>16. Contraindication to warfarin anti-coagulation<br>20. Participation in any other clinical mortality trial	1. Patients who have failed >2 *membrane active* anti-arrhythmic drugs at a therapeutic dose due to inefficacy or side effects (Table 5.2.2)<br>8. Hypertrophic obstructive cardiomyopathy (outflow track)<br>10. Other arrhythmias mandating anti-arrhythmic drug therapy (i.e. VT, VF)<br>16. Contraindication to appropriate anti-coagulation therapy<br>20. Participation in any other clinical mortality trial (Participation in other non-mortality trials should be reviewed with the clinical trial management center)	No revision from 3.4	Clarification of the exclusion criteria
3.2 / 23		Planned atrial flutter ablation in combination with the left atrial ablation is not an exclusion.	No revision from 3.4	Wording added to clarify that planning/performing an atrial flutter ablation is accepted
4.3 / 23	Randomization will be accomplished by telephone using a centralized, IVRS randomization system.	Randomization will be accomplished by telephone or internet using a centralized, interactive voice and web randomization system (IXRS).	No revision from 3.4	To provide clarifications about the randomization system, with additional information about the internet option.
4.4 / 24	Patients will be followed at 6 and 12-month intervals throughout the trial.	Patients will be followed at 6 and 12-month intervals from randomization throughout the	No revision from 3.4	To clarify the timing for the follow-up procedures

		trial.		
4.4.1 / 24	Following a recommended approach consistent with established guidelines, consensus documents, and good clinical practice, a trans-esophageal echocardiographic (TEE) study will be performed within 24 hours prior to ablation in patients with *persistent or longstanding persistent* AF. The TEE may be performed up to 48 hours before the procedure in patients on continuous warfarin at a therapeutic INR or in those appropriately bridged with intravenous un-fractionated or low molecular weight heparin.<br><br>In those centers where routine clinical practice includes the performance of pre-ablation CT / MR studies, relevant data from these studies will be collected to serve as a baseline for comparative quantitative LA size, morphology, and function studies, as well as subsequent follow-up PV and esophageal investigations. In addition at those centers, up to 375 patients randomized to drug therapy will be asked to undergo baseline research CT/MR scanning to allow the CABANA study to evaluate and compare atrial structure and function in response to drug or ablative therapy. These drug-treated	Following a recommended approach consistent with established guidelines, consensus documents, and good clinical practice, a trans-esophageal echocardiographic (TEE) study will be performed within 24 hours prior to ablation in patients with *persistent or longstanding persistent* AF. The TEE may be performed up to 48 hours before the procedure in patients on continuous anticoagulation therapy, such as warfarin at a therapeutic INR or in those appropriately bridged with intravenous un-fractionated or low molecular weight heparin.<br><br>In those centers where routine clinical practice includes the performance of pre-ablation and post-ablation CT/MR studies, relevant data from these studies will be collected to serve as a baseline for comparative quantitative LA size, morphology, and function studies, as well as subsequent follow-up PV and esophageal investigations. In addition at those centers, up to 375 patients randomized to drug therapy will be asked to undergo one ~~baseline~~ research CT/MR scan prior to initiating therapy. This will allow the	No revision from 3.4	The wording has been changed to allow the use of other anticoagulant drugs in addition to warfarine. The timing for performing the baseline CT/MR scans for the ablation patients and for the drug therapy patients at the selected centers has been clarified.

	patients will be recruited from selected centers that are committed to CT / MR assessment of enrolled drug patients. Since randomization is performed (stratified) within each site, this component of the study will still benefit from the overall study randomization. Optimally, the scans performed in all patients should be within 4 months prior to treatment.	CABANA study to evaluate and compare atrial structure and function in response to drug or ablative therapy. These drug-treated patients will be recruited from selected centers that are committed to CT/MR assessment of all enrolled ~~drug~~ patients (both study arms). Since randomization is performed (stratified) within each site, this component of the study will still benefit from the overall study randomization. Optimally, the pre-therapy scans performed in all patients should be within 4 months prior to treatment.		
4.4.2 / 25	CT/MR imaging studies will be performed where clinically indicated or otherwise part of routine clinical care on ablation subjects at baseline, at 3 months post-ablation therapy, and as indicated for PV stenosis management throughout the trial. At sites where baseline and follow-up CT/MR imaging studies are part of routine care for ablation patients, CT/MR imaging studies will also be performed for research purposes on up to 375 drug therapy patients at baseline and 3 months after therapy is fully established (end of blanking period). CT/MR data will be electronically transferred to a server at the Mayo Biomedical	CT/MR imaging studies will be performed where clinically indicated or otherwise part of routine clinical care on ablation subjects ~~at baseline, at 3 months post~~ after ablation therapy (between 90 days post ablation and the 6 month follow-up) and as indicated for PV stenosis management throughout the trial. ~~At sites where baseline and follow-up CT/MR imaging studies are part of routine care for ablation patients~~. The 375 drug therapy patients that received the CT/MR scan prior to initiating drug therapy, ~~imaging studies~~ will also undergo one ~~be performed for~~ research CT/MR scan after therapy is fully	After enrollment, subjects will either receive a single 'CABANA Box' recording system to be used throughout the entire study, or will be examined several times using ambulatory Holter ECG monitoring as generated during the course of routine clinical care as dictated by their attending physician.<br><br>At sites where the 'CABANA Box' has received the appropriate approvals, all patients enrolled will receive a single 'CABANA Box' recording system to be used for both patient activated event monitoring (throughout the	The Medicomp monitoring system will only be utilized at Institutions or within countries where appropriate approvals have been obtained. Routine monitoring based on clinical care will be recorded within the trial database records.<br><br>The timing for performing post-therapy CT/MR scans for the

	Imaging Resource, the CABANA Trial Image Analysis Lab. Scans will be anonymized at the site using a software tool provided by the Imaging Center. Each clinical center will also perform standard site radiology evaluation and assessment of the CT/MR images according to the sites' standard clinical practice.<br><br>Complete follow-up data will also be obtained at the time of treatment discontinuation, with a crossover in treatment strategy, and at the emergence of any primary or secondary endpoints.	established (between 90 days post drug treatment initiation and the 6 month follow-up) ~~(end of blanking period)~~. CT/MR data will be electronically transferred to a server at the Mayo Biomedical Imaging Resource, the CABANA Trial Image Analysis Lab. Scans will be anonymized at the site using a software tool provided by the Imaging Center. Each clinical center will also perform standard site radiology evaluation and assessment of the CT/MR images according to the sites' standard clinical practice.<br><br>Follow-up data will also be obtained at the time of treatment discontinuation, with a crossover in treatment strategy, and at the emergence of any primary or secondary endpoints.	trial), autodetect/autocapture (AD:AC) event monitoring (one 24 hour period/month and full disclosure Holter monitoring (for 96 hours every 6 months) throughout the entire study. Fingertip recordings as well as AD:AC recordings can be transferred via telephone download from the patient's home to the CABANA monitoring center. Holter recordings will require downloading of information at the enrolling site for data transfer to the CABANA monitoring center. All recordings will be made available to the enrolling center for use in clinical practice.	ablation patients and for the drug therapy patients at the selected centers has been clarified.
5.2.3 / 28		Drugs which have been approved by appropriate regulatory agencies outside of the United States may be used within that jurisdiction.	No revision from 3.4	To explain that the protocol allows the use of new drugs which have been approved by appropriate regulatory agencies outside of the United States within the respective jurisdictions, as a chief aim of the trial is to provide

				relevant, up-to-date information for guiding drug and ablative therapy for AF.
5.2.6 / 28	During follow-up, drug arm patients with recurrences may be treated with additional anti-arrhythmic drugs or alternative rate control agents. Ablative intervention is strongly discouraged. Of note, most patients included to date in single center trials were treated for over one year before undergoing ablation. Patients will be fully informed of this at the time of the consent process. **Crossovers must be approved by the CABANA Trial Administrative Center.**	During follow-up, drug arm patients with recurrences may be treated with additional anti-arrhythmic drugs or alternative rate control agents. Of note, most patients included to date in single center trials were treated for over one year before undergoing ablation. Thus, it is anticipated that most patients will be treated for at least 12 months in the drug therapy arm and that patients will be fully informed of this at the time of the consent process. Cross over ablative intervention is strongly discouraged. **Crossovers must be approved by the CABANA Trial Administrative Center.** **Before approval is granted for a patient randomized to drug therapy to be crossed over to ablation,** sites will be required to provide rationale and documentation that drug therapy options have been exhausted.	No revision from 3.4	To reinforce that crossover of the patients from the drug therapy arm to ablation therapy is discouraged and subject of approval by the CABANA Trial Administrative Center.
5.3.3 / 29		Catheters which have been approved by appropriate regulatory agencies outside of the United States may be used within that jurisdiction.	No revision from 3.4	To clarify catheters which have been approved by appropriate regulatory agencies

				outside of the United States may be used within that jurisdiction, even if they are not listed in the protocol.
5.4.1 / 31	Patients with risk factors for CVA or peripheral thromboembolic events at the time of enrollment, treated with rate control agents alone, will remain on active anticoagulation therapy with warfarin throughout the duration of the trial [92]. Unlike the AFFIRM trial, patients receiving rhythm control therapy will also be required to receive warfarin anticoagulation for the duration of the trial. In both cases, target INRs of 2 to 3 will be required, unless higher INRs are mandated because of underlying disease.	Patients with risk factors for CVA or peripheral thromboembolic events at the time of enrollment, treated with rate control agents alone, will remain on active anticoagulation therapy with ((warfarin, dabigatran) throughout the duration of the trial [92]. Unlike the AFFIRM trial, patients receiving rhythm control therapy will also be required to receive warfarin adequate anticoagulation (warfarin, dabigatran) for the duration of the trial. In both cases the use of warfarin therapy, target INRs of 2 to 3 will be required, unless higher INRs are mandated because of underlying disease.		Changes have been implemented to allow the use of other anticoagulation drugs (dabigatran).
5.4.2 / 31	Anticoagulation before, during, and after the ablative intervention will follow the guidelines of the AF Ablation Consensus Document [50]. Prior to the ablative intervention, patients with persistent and long-standing persistent AF should receive at least one month of warfarin anticoagulation (INRs: 2-3), or	Anticoagulation before, during, and after the ablative intervention will follow the guidelines of the AF Ablation Consensus Document [50]. Prior to the ablative intervention, patients with persistent and long-standing persistent AF should receive adequate anticoagulation (i.e. at least one month of warfarin	No revision from 3.4	Changes have been implemented to allow the use of other anticoagulation drugs (dabigatran), in accordance with the recent guidelines of the AF Ablation Consensus

	have a TEE excluding intra-atrial thrombus at the time of the intervention. During the ablative intervention, maintaining an ACT between 300 and 400 seconds is strongly recommended. Following the ablative intervention, patients will be started on IV heparin or subcutaneous injections of low molecular weight heparin beginning 4 to 6 hours after all sheaths are removed, and warfarin anti-coagulation reinstituted the evening of the intervention. Thereafter, low molecular weight heparin is to be maintained until standard dose warfarin achieves a target INR of 2 to 3, unless the ablation was performed at a therapeutic INR in patients maintained on warfarin through the ablation. Three to six months after ablation, warfarin may be replaced by full dose aspirin in patients with a CHADs score $\leq$ 1. This would include patients with hypertension without hypertrophy, or those <65 years of age, providing 1) atrial size and function are normal and 2) there is no symptomatic or asymptomatic AF by standard or full-disclosure monitoring. In those patients with a CHADs score $\geq$2, warfarin is to be continued throughout the trial. Randomization of warfarin	anticoagulation (INRs: 2-3)), or have a TEE excluding intra-atrial thrombus at the time of the intervention. During the ablative intervention, maintaining an ACT between 300 and 400 seconds is strongly recommended. Following the ablative intervention, patients will be started on IV heparin or subcutaneous injections of low molecular weight heparin beginning 4 to 6 hours after all sheaths are removed, and ~~warfarin~~ appropriate anticoagulation reinstituted the evening of the intervention. Thereafter, low molecular weight heparin is to be maintained until anticoagulated appropriately or standard dose warfarin achieves a target INR of 2 to 3, unless the ablation was performed at a therapeutic INR in patients maintained on warfarin through the ablation. One month following the ablation, Dabigatran may be substituted for warfarin, following recently written guidelines. Three to six months after ablation, warfarin may be replaced by full dose aspirin in patients with a CHADs score $\leq$ 1. This would include patients with hypertension without hypertrophy, or those <65 years of age, providing 1) atrial size		Document.

	discontinuation is precluded by the low post-ablation stroke rates that would require at least 10,000 patients for detecting differences in stroke prevalence. Nevertheless, the characteristics, follow-up monitoring results, and long-term anticoagulation status of ablation patients will be compared to descriptively identify predictors of events and profiles of patients at high risk for warfarin discontinuation. The utility of trans-telephonic, auto-detection / full disclosure, and Holter monitoring, as a future aid in the decision to discontinue anticoagulation will also be critically examined.	and function are normal and 2) there is no symptomatic or asymptomatic AF by standard or full-disclosure monitoring. In those patients with a CHADs score ≥2, adequate anticoagulation ~~warfarin~~ is to be continued throughout the trial. Randomization of warfarin discontinuation is precluded by the low post-ablation stroke rates that would require at least 10,000 patients for detecting differences in stroke prevalence. Nevertheless, the characteristics, follow-up monitoring results, and long-term anticoagulation status of ablation patients will be compared to descriptively identify predictors of events and profiles of patients at high risk for warfarin discontinuation. The utility of trans-telephonic, auto-detection / full disclosure, and Holter monitoring, as a future aid in the decision to discontinue anticoagulation will also be critically examined.		
5.4.3 / 31	It is likely during the course of the trial that newer antithrombotic therapies non-inferior to warfarin will be approved. These agents may be used as replacement therapy for warfarin on approval of Innovative Antithrombotic Therapies/Executive Committees.	It is likely during the course of the trial that newer antithrombotic therapies non-inferior to warfarin or dabigatran will be approved. These agents may be used as replacement therapy for warfarin on approval of Innovative Antithrombotic Therapies/Executive	No revision from 3.4	Changes have been implemented to allow the use of other anticoagulation drugs (dabigatran).

		Committees.		
5.4.4 / 31	Specific items left to the investigators discretion include: 1) specific choice of rate control vs. rhythm control drug therapy and specific drugs to be used; 2) hospitalization to initiate anti-arrhythmic drug therapy; 3) choice of TEE guided direct current cardioversion (DCCV) vs. DCCV after 4 weeks of warfarin to an INR of 2-3; 4) pre-ablation TEE assessment in patients with simple paroxysmal AF and hypertension without hypertrophy; 5) continuation of warfarin to maintain a therapeutic INR at the time of catheter ablation.	Specific items left to the investigators discretion include: 1) specific choice of rate control vs. rhythm control drug therapy and specific drugs to be used; 2) hospitalization to initiate anti-arrhythmic drug therapy; 3) choice of TEE guided direct current cardioversion (DCCV) vs. DCCV after 4 weeks of appropriate anticoagulation therapy, such as warfarin to an INR of 2-3; 4) pre-ablation TEE assessment in patients with simple paroxysmal AF and hypertension without hypertrophy; 5) continuation of warfarin to maintain a therapeutic INR at the time of catheter ablation; 6) selection of warfarin versus dabigatran.	No revision from 3.4	Changes have been implemented to allow the use of other anticoagulation drugs (dabigatran).
6.2 / 32	While hospitalization for AF in both treatment arms will be carefully tracked and compared, it will be considered as an *"AF recurrence"* and counted against efficacy, not safety. Therefore, AF recurrence and/or worsening of AF does not need to be reported as an adverse event.	While hospitalization for AF in both treatment arms will be carefully tracked and compared, it will be considered as an *"AF recurrence"* and counted against efficacy, not safety. Therefore, AF recurrence and/or worsening of AF ~~does~~ **should not** be reported as an adverse event.	No revision from 3.4	Change implemented to increase the clarity of the text.
7.0 / 32	An adverse event (AE) will be considered present if 1) there are untoward signs, symptoms, illnesses, or other medical events that develop or worsen in severity during the course of the study, 2) they are clinically	An adverse event (AE) will be considered present if 1) there are untoward signs, symptoms, illnesses, or other medical events that develop or worsen in severity during the course of the study, 2) they are clinically	No revision from 3.4	The text had been updated to provide additional guidance and clarifications to the investigators for the assessment and reporting of

	relevant and if they are clinically related to the study. Note: Disease, signs symptoms, and or laboratory abnormalities already existing at randomization are not considered adverse events unless they represent an intensity or frequency exacerbation. Surgical procedures themselves are not adverse events; they are therapeutic measures for conditions that require surgery. The condition for which the surgery is required may be an adverse event. Surgical procedures planned prior to randomization and the conditions leading to these measures are not adverse events.	relevant **and** if they are clinically related to the study. Note: Disease, signs symptoms, and or laboratory abnormalities already existing at randomization are not considered adverse events unless they represent an intensity or frequency exacerbation. An adverse event designation should reflect the reason for a diagnosis or abnormal measurement. ~~Surgical procedures themselves are not adverse events; they are therapeutic measures for conditions that require surgery. The condition for which the surgery is required may be an adverse event. Surgical~~ Procedures planned prior to randomization and the conditions leading to these measures are not adverse events.		adverse events.
7.0 / 33	The DCRI will evaluate any safety information that is spontaneously reported in the time frame specified in the protocol. For each subject, adverse events occurring after randomization must be recorded on the applicable Adverse Events page(s) in the electronic Case Report Form (eCRF). Recording should be done in a concise manner using standard, acceptable medical terms. The adverse event recorded should	The DCRI will evaluate any safety information that is spontaneously reported in the time frame specified in the protocol. For each subject, adverse events occurring after randomization must be recorded on the applicable Adverse Events page(s) in the electronic Case Report Form (eCRF). Recording should be done in a concise manner using standard, acceptable medical terms. The adverse event	No revision from 3.4	The text had been updated to provide additional guidance and clarifications to the investigators for the assessment and reporting of adverse events.

	not be a procedure or a clinical measurement (i.e., a laboratory value or vital sign) but should reflect the reason for the procedure or the diagnosis based on the abnormal measurement. It is the responsibility of the Principal Investigator to oversee the safety of the study at his/her site. This safety monitoring will include careful assessment and appropriate reporting of adverse events.	recorded should not be a procedure or a clinical measurement (i.e., a laboratory value or vital sign) but should reflect the reason for the procedure or the diagnosis based on the abnormal measurement. It is the responsibility of the Principal Investigator to oversee the safety of the **patients enrolled in the** study at his/her site. **The responsibility for safety oversight includes careful assessment and appropriate reporting of adverse events. The primary mechanism for reporting adverse events in CABANA is for study personnel at the clinical sites to enter the relevant information and the details and description of each event using the adverse event forms that are part of the InForm electronic data capture (EDC) system being used in the trial.**		
7.1.1 / 33	An anticipated event is one that has been previously identified in previous studies, published literature, or product labeling to be related to the disease state or therapies. A listing of 'Anticipated' events can be found in Appendix A. An unanticipated adverse event is any occurring that has not been previously reported (see	An anticipated event is one that has been previously identified in previous studies, published literature, or product labeling to be related to the disease state or therapies. A listing of '**anticipated/expected'** events can be found in Appendix A. An unanticipated**/unexpected** adverse event is any occurring that has not been reported **in**	No revision from 3.4	The text had been updated to provide additional guidance and clarifications to the investigators for the assessment and reporting of adverse events.

	appendix A).	**previous studies, published literature, or product labeling** (see appendix A).		
7.1.2 / 33	c. 23 hour hospitalizations.	c. 23 hour hospitalizations **(observation)**.	No revision from 3.4	
7.1.3 / 34	1. **Mild**: Any event that results in minimal transient impairment of a body function and does not threaten damage to a body structure, and/or does not require intervention other than monitoring.	1. **Mild**: Any event that results in minimal transient impairment of a body function and does not threaten damage to a body structure, and/or does not require intervention other than monitoring **(easily tolerated).**	No revision from 3.4	The text had been updated to provide additional clarifications to the investigators for the assessment of adverse events.
7.1.4 / 34	1. **Definitely related**: reasonable temporal relationship to study therapy or device<br>a. follows a known response pattern (e.g., study drug, treatment or device is known to cause this AE)<br>b. there is no alternative etiology or explanation for the event<br>2. **Probably related**: reasonable temporal relationship<br>a. follows a suspected response pattern<br>b. no evidence for a more likely alternative etiology though could be unrelated<br>3. **Possibly related:** reasonable temporal relationship<br>a. equivocal evidence that the event is study related as opposed to an	**The International Council of Harmonization (ICH) Guidelines (1995) indicate that "reasonable causal relationship" means that "there are facts [evidence] or arguments to suggest a causal relationship." The causality assessment must be made by the investigator based on information available at the time that the adverse event eCRF is completed. The initial causality assessment may be revised as new information becomes available.**<br>1. **Definitely related**: **there is a** reasonable temporal relationship to study therapy or device<br>a. follows a known response pattern (e.g., study drug, treatment or device is known to	No revision from 3.4	Change implemented to increase the clarity of the text.

	alternative etiology<br>4. **Probably not related:** does not have a reasonable temporal relationship OR<br>a. good evidence for a more likely alternative etiology<br>5. **Not related:** does not have a temporal relationship OR<br>a. clear and compelling evidence that the event is due to an alternative etiology<br>b. ICH guidelines (1995) clarify “reasonable causal relationship” to mean that “there are facts [evidence] or arguments to suggest a causal relationship.” The causality assessment must be made by the investigator based on information available at the time that the adverse event eCRF is completed. The initial causality assessment may be revised as new information becomes available.<br><br>All adverse events, serious and non-serious, that occur between the time of randomization and the last study-related procedure/visit will be followed until resolution, stabilization, or until the last subject enrolled	cause this AE)<br>b. there is no alternative etiology or explanation for the event<br>2. **Probably related**: **there is a** reasonable temporal relationship **which**<br>a. follows a suspected response pattern<br>b. no evidence for a more likely alternative etiology though could be unrelated<br>3. **Possibly related: there is a** reasonable temporal relationship **but**<br>a. equivocal evidence that the event is study related as opposed to an alternative etiology<br>4. **Probably not related: there is** not a reasonable temporal relationship OR<br>a. good evidence for a more likely alternative etiology<br>5. **Not related: there is** not ~~have~~ a temporal relationship OR<br>a. clear and compelling evidence that the event is due to an alternative etiology<br>~~b. ICH guidelines (1995) clarify “reasonable causal relationship” to mean that “there are facts [evidence] or arguments to suggest a~~		

	completes the follow-up phase of the trial. Adverse events for subjects who discontinue study participation at any time during the study should be collected/reported through at least the time of discontinuation. In addition, required Institutional reporting structure will be followed and/or as described in this protocol.	causal relationship." The causality assessment must be made by the investigator based on information available at the time that the adverse event eCRF is completed. The initial causality assessment may be revised as new information becomes available.		
7.2 / 35	All adverse events, serious and non-serious, that occur between the time of randomization and the last study-related procedure/visit will be followed until resolution, stabilization, or until the last subject enrolled completes the follow-up phase of the trial. Adverse events for subjects who discontinue study participation at any time during the study should be collected/reported through at least the time of discontinuation. In addition, required Institutional reporting structure will be followed and/or as described in this protocol.	The goal is to have an adverse event reporting process that is (a) clear and simple for site investigators and study coordinators to understand and implement, (b) satisfies all regulatory reporting requirements, (c) eliminates any duplication in data collection and reporting, and (d) has a balanced focus on both the drug and ablation arms of the trial.<br><br>All related adverse events, serious and non-serious, that occur between the time of randomization and the last study-related procedure/visit will be followed until resolution, stabilization, or until the last subject enrolled completes the follow-up phase of the to trial completion. Adverse events for subjects who discontinue study participation at any time during	No revision from 3.4	The text had been updated to provide additional guidance and clarifications to the investigators for the assessment and reporting of adverse events.

		the study should be collected/reported through at least the time of discontinuation. In addition, the required Institutional reporting structure will be followed and/or as described in this protocol.		
7.3 / 35	Regardless of causality, the investigator will record all serious adverse events occurring between randomization and the last study-related procedure/visit or completion of the trial into the electronic database within 24 hours of knowledge of the event. DCRI will report all unanticipated adverse device effects (UADE) to Mayo Clinic and the DSMB chair within 2 business days of receipt.	Regardless of causality, the investigator will record all serious adverse events occurring between randomization and the last study-related procedure/visit or completion of the trial into the electronic database within 24 hours of knowledge of the event. DCRI will report all unanticipated**/unexpected** adverse **events** to Mayo Clinic and the DSMB chair within 2 business days of receipt.	No revision from 3.4	Change implemented to increase the clarity of the text.
7.4 / 35	Specified events as listed below that meet serious criteria (see section 7.1.2 of the protocol) are unanticipated and probably/definitely related, if occurring between randomization through completion of follow-up (end of trial) require ***expedited*** reporting by the DCRI and in turn to the appropriate regulatory agencies.	Specified events as listed below that meet ***serious*** criteria (see section 7.1.2 of the protocol), ***related (***possibly/probably/definitely) to either study drug or the ablation device or procedure, and are ***unanticipated/unexpected*** if occurring between randomization through completion of follow-up (end of trial) require ***expedited*** reporting **to** the DCRI and in turn to the appropriate regulatory agencies.	No revision from 3.4	The text had been updated to provide additional guidance to the investigators for the assessment of adverse events.
7.4.1 / 35	1. Unanticipated ablation procedure related events	**1. Unexpected, SAE related to study drug**	No revision from 3.4	The text had been updated to clarify

	2. Unanticipated Adverse Device Effect events (UADEs)<br>3. Device Failures or Malfunctions<br>4. **Events of Interest (EOI)**: Ablation therapy or Procedure related events (index and/or follow-up): All events that resulted in death, myocardial perforation with tamponade requiring intervention, esophageal atrial fistula, and/or severe pulmonary vein stenosis that were life threatening or classified as severe in nature.	2. Unanticipated ablation procedure related events<br>3. Unanticipated Adverse Device Effect (UADEs)<br>4. Device failures or malfunctions<br>5. **Events of Interest (EOI)**: Drug or ablation therapy or ablation procedure related events (index and/or follow-up): All events that resulted in death, pro-arrhythmic events, myocardial perforation ~~with~~ / tamponade requiring intervention, esophageal atrial fistula, and/or severe pulmonary vein stenosis that were life threatening or classified as severe in nature.		that unexpected adverse events related to study drug also require expedited reporting.
7.5 / 36	Expedited Events must be entered on the appropriate eCRF pages or if the electronic database is unavailable reported on an *Expedited Event Form* and faxed to DCRI Safety Surveillance within 24 hours of knowledge of the event. When available, the event must be entered into the electronic data base.<br>**DCRI Safety Surveillance**<br>Telephone: 1-919-668-8624<br>Toll Free: 1-866-668-7799<br>Fax: 1-919-668-7138<br>Toll Free Fax: 1-866-668-7138<br>**Safety Surveillance Medical Reviewer**<br>Lynda Szczech, M.D.<br>Telephone: 1-919-668-8918	Expedited Events must be entered on the appropriate eCRF pages or if the electronic database is unavailable for more than 24 hours, the event would be reported on an *Expedited Event Form* and faxed/emailed to DCRI Safety Surveillance within 24 hours of knowledge of the event. When available, the event must be entered into the electronic data base.<br>**l Safety Surveillance**<br>phone: 1-919-668-8624<br>ree: 1-866-668-7799<br>l: Safetysurveillance@mc.duke.ed<br>1-919-668-7138	No revision from 3.4	The text had been updated to provide clarifications to the investigators for reporting adverse events to DCRI Safety Surveillance

	Toll Free Fax: 1-866-668-7138			
7.6 / 36	The DCRI Safety Surveillance Medical Monitor will review all SAEs for expectedness.		No revision from 3.4	The text had been removed as the information is also provided in section 7.6.2.
7.6.1 /36	Since the CABANA Trial is not under an Investigational New Drug Application, the principle investigator and/or designee at the site will be required to complete and submit form 3500A for reporting serious adverse events (SAEs) that are drug related and unexpected via the FDA's MedWatch Adverse Event Reporting program, DCRI Safety Surveillance and/or their designee. Events may be reported online at www.fda.gov/MedWatch/report.htm, by phone 1-800-FDA-1088, or by returning the postage-paid FDA form 3500 which may be downloaded from www.fda.gov/MedWatch/getforms.htm by mail to MedWatch, 5600 Fishers Lane, Rockville, MD 20852-9787 or fax 1-800-FDA-0178. DCRI Safety Surveillance will be responsible for notifying the NIH. Sites should fax a copy of the completed MedWatch form and a cover sheet documenting the date and time the MedWatch form was submitted.	**Physician Reporting of Drug or Device Adverse Events Physician reporting of drug or device-related unexpected/unanticipated serious adverse events (as mandated by regulatory authorities) using MedWatch or Council for International Organizations of Medical Sciences (CIOMS) forms, should continue independently of any CABANA reporting. It is anticipated that physicians and/or the appropriate healthcare professional will complete this reporting by, 1) MedWatch- submit form 3500 for drug or device via the FDA's MedWatch Adverse Event Reporting program online at www.fda.gov/MedWatch/report.htm, by phone 1-00-FDA-1088, or by returning the postage-paid FDA form 3500 downloaded from ww.fda.gov/MedWatch/getforms.htm by mail to MedWatch, 5600 Fishers Lane, Rockville,**	No revision from 3.4	The text had been updated to provide additional guidance and clarifications to the investigators for reporting of adverse events.

	**All Events of Interest**: (as identified above) will require expedited reporting to the NIH by the DCRI Safety Surveillance within 5 business days of initial notification.	**MD 20852-9787 or fax 1-800-FDA-0178, 2) CIOMS- using http://www.cioms.ch/index.htm, postal address: c/- World Health Organization, Avenue Appia, 20 CH - 1211 Geneve, 27 Switzerland, telephone: +41 (0) 22 791 34 13 or fax: +41 (0) 22 791 42 86.**		
7.6.2 / 36	The DCRI Medical Monitor will determine which device-related expedited events meet "unanticipated" criteria (not labeled in the literature). Unanticipated adverse device effects (UADEs) will be reported to the NIH within 1-2 business days, and to the FDA and all participating investigators within 10 working days of DCRI Safety Surveillance's initial notification of the event. Investigators are responsible for reporting UADEs to their reviewing IRB within 10 working days of first learning of the effect. MedWatch surveillance will remain the responsibility of the site using the same approach as noted above.	**CABANA Reporting of Drug or Device Adverse Events For CABANA trial purposes, adverse events will be reported through the eCRF submission process designed to facilitate notification to DCRI Safety Surveillance and/or their designee.** The DCRI Safety Surveillance Medical Monitor **(a physician trained and experienced in safety reporting)** will review all SAEs for **expectedness. potential expected/unexpected; anticipated/unanticipated status.** The decision regarding ultimate classification will be made by individuals within CABANA Leadership with expertise in antiarrhythmia and ablation therapies and clinical trial experience.<br>**All Events of Interest**: (as identified above) will require expedited reporting to the NIH by the DCRI Safety Surveillance within 5 business	No revision from 3.4	The text had been updated to clarify the process followed by DCRI Safety Surveillance for the assessment of adverse events.

		days of initial notification.		
7.6.3 / 36	The CABANA exclusion criteria specifically excludes women of childbearing potential unless post-menopausal or surgically sterile. If a pregnancy does occur during the follow-up period, please notify the DCRI Safety Surveillance at 919-668-8624. Pregnancies are not considered adverse events. Complications or medical problems associated with a pregnancy are considered AEs and may be SAEs. Complications or medical problems are reported as AEs/SAEs if they occur during the study follow-up period according to the protocol.	~~The CABANA exclusion criteria specifically excludes women of childbearing potential unless post-menopausal or surgically sterile. If a pregnancy does occur during the follow-up period, please notify the DCRI Safety Surveillance at 919-668-8624. Pregnancies are not considered adverse events.~~	No revision from 3.4	The section about women of childbearing potential was removed, as it is not applicable for this study.
8.1 / 37	Another important factor that must be considered in these calculations is the extent to which patients randomized to the drug arm may **cross over** to receive an ablation during the course of their follow-up (because the AF and its symptoms are not adequately controlled by drugs).	Another important factor that must be considered in these calculations is the extent to which patients randomized to the drug arm may **cross over** to receive an ablation during the course of their follow-up (~~because~~if the AF and its symptoms are not adequately controlled by drugs).	No revision from 3.4	Editorial changes to increase the clarity of the text.
8.2.1 / 39	The log-rank test will be the primary analytic tool for comparing mortality differences between the two therapies. Kaplan-Meier estimates of cumulative mortality rates as a function of follow-up time will be calculated and displayed.	The log-rank test will be the primary analytic tool for comparing mortality differences between the two therapies. Kaplan-Meier estimates of cumulative mortality rates as a function of follow-up time will be calculated and displayed.	No revision from 3.4	Editorial changes to increase the clarity of the text.

	Relative risks will be expressed as hazard ratios with 95% confidence intervals using the Cox proportional hazards model	Relative risks will be expressed as hazard ratios with 95% confidence intervals generated using the Cox proportional hazards model		
11.1 / 45	During monitoring visits, the Monitor will perform a one hundred percent (100%) review of all Inclusion/Exclusion criteria, informed consent, HIPAA Authorization, all events meeting criteria for expedited event reporting as well as safety and efficacy endpoints. Additional review will be performed on a site-by-site basis, as warranted by the findings of previous monitoring visits. Key variables (demographics, inclusion/exclusion criteria, and safety) on the eCRFs) will be compared with each subject's source documents. Any discrepancies will be noted and resolved.	During monitoring visits, the Monitor will perform a **ten** percent (10%) review of all Inclusion/Exclusion criteria **of the randomized subjects since last periodic monitoring visit. Review of 100% of the** informed consent **forms**, HIPAA Authorization, all events meeting criteria for expedited event reporting as well as **10% of SAE's and** Events of Interest **EOI will be performed**. Additional review will be performed on a site-by-site basis, as warranted by the findings of previous monitoring visits. Key variables (demographics, inclusion/exclusion criteria, and safety) on the eCRFs) will be compared with each subject's source documents. Any discrepancies will be noted and resolved.	No revision from 3.4	The text had been updated to clarify the expectations during site monitoring visits
13.0 / 47	6. Description of adverse events and follow-up of the adverse events (minimally event description, severity, onset date, duration, relation to study device, outcome and treatment for adverse event).	6. Description of adverse events and follow-up of the adverse events (minimally event description, severity, onset date, duration, relation to study drug or device, outcome and treatment for adverse event).	No revision from 3.4	Editorial changes to increase the clarity of the text.
14.0 / 48	Investigators will maintain	No revisions for 3.3	Investigators will maintain	Editorial changes to

	documentation of the dates and reasons for each deviation from the protocol, in compliance with Code of Federal Regulations (CFR) 812.140.		documentation of the dates and reasons for each deviation from the protocol, in compliance with the ICH-GCP guidelines, Code of Federal Regulations (CFR) 812.140 and national legislation.	reflect the accurate legislation locally for all sites within all their countries respectively
16.0 / 49	Upon completion of the study (defined by all subjects have completed all follow-up visits, all eCRFs are complete, and all queries have been resolved, DCRI and/or their designee will notify the site of closeout and a study closeout visit will be performed. The DCRI monitor and/or their designee will ensure that the Investigator's regulatory files are up to date and complete, and that any outstanding issues from previous correspondences have been resolved. Other issues to be reviewed at the closeout visit include: discussing retention of study files, possibility of site audits, publication policy, and notifying the IRB of study closure.	No revisions for 3.3	The end of trial is defined as the day of the last visit of the last subject in the trial.<br><br>For clinical trial sites located in the EU, a declaration of the end of the clinical trial will be made according to the procedures outlined in Directive 2001/20/EC. For sites located in countries outside the EU, local regulations will be followed.<br><br>Upon completion of the study ~~(defined by all subjects have completed all follow-up visits, all eCRFs are complete, and all queries have been resolved,)~~ DCRI and/or their designee will notify the site of closeout and a study closeout visit will be performed. The DCRI monitor and/or their designee will ensure that the Investigator's regulatory files are up to date and complete, and that any outstanding issues from previous correspondences have been resolved. Other issues to be reviewed at the	Editorial changes defining the end of the trial.<br><br>Language inserted for regulatory compliance

			closeout visit include: discussing retention of study files, possibility of site audits, publication policy, and notifying the IRB of study closure.	
18.0 / 50	The principal investigator or IRB-documented members of the research team will approach the patient to obtain written informed consent. The underlying rationale for the study, the procedures to be followed, the potential benefits, risks, alternatives, and other issues mandated by the consent process will be fully disclosed. Written informed consent will be documented on an informed consent form (ICF) approved by the same IRB responsible for approval of this protocol, The ICF will conform to FDA regulations in 21 CFR Part 50, and to the institutional requirements for informed consent and applicable regulations. The investigator agrees to obtain approval from DCRI and/or their designee of any ICF intended for use in the study, prior to submission for IRB approval.	No revisions for 3.3	The principal investigator or IRB-documented members of the research team will approach the patient to obtain written informed consent on an informed consent form (ICF) approved by the same IRB/EC responsible for approval of this protocol. The informed consent document will conform to FDA regulations in 21 CFR Part 50, and/or to the national requirements for informed consent. It must include all elements required by law, local regulations, GCP and International Conference on Harmonization guidelines and study specific procedures. The underlying rationale for the study, the procedures to be followed, the potential benefits, risks, alternatives, and other issues mandated by the consent process will be fully disclosed. If new information become available during the course of the trial that may be relevant to the subject's consent, the Informed Consent Form will be revised and the revised version will be	Language revised/ inserted for regulatory compliance

			submitted for EC/IRB approval before use. Written informed consent will be documented on an informed consent form (ICF) approved by the same IRB responsible for approval of this protocol. The ICF will conform to FDA regulations in 21 CFR Part 50, and to the institutional requirements for informed consent and applicable regulations.	
19.0 / 50	Subject information collected in this study will comply with the standards for protection of privacy of individually identifiable health information as promulgated in the Health Assurance Portability and Accountability Act and as mandated in Title 45 CFR, Parts 160 and 164. All records will be kept confidential and the subject's name will not be released by study staff at any time. Subject records will not be released to anyone other than DCRI and/or their designee, and responsible regulatory authorities when requested. In all cases, caution will be exercised to assure the data are treated confidentially and that the subject's privacy is protected.	No revisions for 3.3	Subject information collected in this study will comply with the standards for protection of privacy of individually identifiable jhealth information as promulgated in the Health Assurance Portability and Accountability Act and as mandated in Title 45 CFR, Parts 160 and 164 and all records will be kept confidential and the subject's name will not be released by study staff at any time.<br><br>Subject records will not be released to anyone other than DCRI and/or their designee, and responsible regulatory authorities. The subject must be informed that his/her personal trial-related data will be used by the sponsor in accordance with the local data	Language revised/ inserted for accuracy and regulatory compliance

			protection legislation. The subject will also be informed that, when requested, his / her medical records may be examined by authorized monitors (DCRI and/or their designee) or Clinical Quality Assurance auditors appointed by the sponsor, by appropriate IRB / IEC members and by domestic and foreign regulatory authorities. In all cases, caution will be exercised to assure the data are treated confidentially and that the subject's privacy is protected.	
20.0 / 51	An Authorization for use and disclosure of protected health information (PHI) under the HIPAA Privacy Rule [45 CFR § 164.102 *et seq*] will be obtained from every trial subject prior to, or at the time of, enrollment.	No revisions from 3.3	~~A~~ For clinical trial sites located in the US, an Authorization for use and disclosure of protected health information (PHI) under the HIPAA Privacy Rule [45 CFR § 164.102 *et seq*] will be obtained from every trial subject prior to, or at the time of, enrollment.	Language revised/ accuracy and regulatory compliance
22.0 / 51	The appropriate IRB/EC must approve the protocol and informed consent documents, agree to monitor the conduct of the study, and agree to review study progress periodically, at intervals not to exceed 1 year. The investigator will provide DCRI or their designee with documentation that the IRB has approved the study *before* the study may begin.	No revisions from 3.3	This study will be initiated only after all required legal documentation has been reviewed and approved by the respective IRB / EC and competent authority (CA) according to national and international regulations. ~~The appropriate IRB/EC must approve the protocol and informed consent documents, agree to monitor the conduct~~	Language revised/ accuracy and regulatory compliance

	In addition, the investigator must provide the following documentation to DCRI or their designee.<br>1. IRB annual re-approval of the protocol, per current Title 21 CFR 312.66 regulations and 1997 International Conference on Harmonization guidelines.<br>2. IRB approval of revisions to the informed consent documents or any amendments to the protocol. Any revisions to the protocol that may increase subject risk exposure must be approved prior to implementation. Administrative changes (such as a change in address or phone number) must be sent to IRBs/Ethics Committees but do not require their approval. The investigator will provide DCRI or their designee with documentation of all approvals.		~~of the study, and agree to review study progress periodically, at intervals not to exceed 1 year.~~ The investigator will provide DCRI or their designee with the study approval documentation before the study may begin. ~~documentation that the IRB has approved the study *before* the study may begin.~~<br>The same is applicable for the implementation of changes introduced by amendments.<br>Where applicable~~, In addition~~, the investigator must also provide to DCRI and/or their designee the following ~~documentation to DCRI or their designee.~~<br>1. A copy of IRB annual re-approval of the protocol, per current Title 21 CFR 312.66 regulations and 1997 International Conference on Harmonization guidelines.<br>2. IRB approval of revisions to the informed consent documents ~~or any amendments to the protocol. Any revisions to the protocol that may increase subject risk exposure must be approved prior to implementation.~~ Administrative changes (such as a change in address or phone number) must be sent to IRBs/Ethics	

			Committees but do not require their approval. ~~The investigator will provide DCRI or their designee with documentation of all approvals.~~<br>3. The investigator must submit periodic status reports to their EC as required, as well as notification of completion of the study and a final report where applicable.<br>4. The investigator will provide DCRI or their designee with documentation of all approvals.	
24.0 / 52		**24.0 Sub-Studies**<br><br>**24.1 CABANAgene**<br>CABANAgene: a resource that will accumulate DNA samples from CABANA subjects to enable subsequent genotype-phenotype studies.<br><br>Appendix C, page 62, the CABANAgene protocol, provides the rationale for studies and approaches that are anticipated. The major goal of CABANAgene is to create the resource. Specific projects to use the samples would require approval of the CABANA study group, and phenotypes to be studied would be those collected by and adjudicated by the CABANA	**24.0 Sub-Studies**<br><br>All activated sites will be given the opportunity to participate or not participate. All proposed sub-studies will be first submitted for review and approval to the respective IRB / EC and competent authority (CA) based on national and international regulations. It is further acknowledged that subjects enrolled in CABANA will have the choice to participate or to not participate in sub-studies.	Language added for clarity that all Sub-Studies are optional for activated sites and enrolled subjects.<br><br>CABANA is comparing two major approaches for management of atrial fibrillation (AF): drugs to maintain sinus rhythm and ablation. As in all other large clinical trials, responses to therapy will be variable in both treatment arms. Abundant evidence

		study group. The CABANAgene project is included in the Pharmacogenetics Research Network (PGRN) arrhythmia site renewal. Participation under the PGRN umbrella also provides access to advanced genotyping and genetic statistical methods to investigators accessing the CABANAgene resource.<br><br>Examples of issues that CABANAgene could address include: 1) predictors of response to antiarrhythmic or rate control drug therapies; 2) predictors of response to warfarin therapy; 3) predictors of response to ablation therapy and; 4) clinical and genetic approaches to defining AF subtypes.		points to genetic factors as a contributor to such variability in important human phenotypes such as response to treatment, and the goal of CABANAgene is the creation of a large resource linking to test hypotheses that relate genetic variation to disease susceptibility and treatment responses. Collecting DNA from patients in CABANA will address questions such as which patients are most likely to respond to ablation or drug therapy and which patients are most likely to develop complications with ablation or drug therapy.
Appendix A / 60	Deleted (see below)	Added (see below)		The list of Anticipated Events has been updated according to the most recent clinical data.

Appendix C / 62		Sub study CABANAgene (see protocol V3.4, Appendix C)		Same as above in 24.0

~~Appendix A~~ DELETED

The following events have been identified as "Anticipated Events" for subjects randomized to medical therapy for this study.

Medical Therapy events

1. Bradycardia
2. Heart Block
3. Bundle Branch block
4. Intraventricular conduction delay
5. Hypotension
6. Dizziness / light headedness
7. Syncope
8. Shortness of breath
9. Fatigue
10. Wheezing
11. Asthma exacerbation
12. Nausea/vomiting
13. Depression
14. Impotence
15. Peripheral edema
16. Heart failure
17. Skin rashes
18. Diarrhea
19. Constipation
20. Poor appetite
21. Alteration of color vision
22. Blurred / double vision
23. Deteriorating vision
24. Blindness
25. Unusual metallic taste or changes in taste
26. Hyperthyroidism
27. Hypothyroidism
28. Abnormal liver functions
29. Photosensitivity
30. Bluish / gray skin tone
31. Unsteady gait / imbalance
32. Lung toxicity/interstitial inflammation
33. Drug related nonsustained VT
34. Sustained VT
35. Prolonged QT/Torsade des pointes
36. Proarrhythmia; new or worsened
37. Ventricular fibrillation
38. Ventricular tachycardia
39. Stroke / thromboembolic event
40. Renal failure
41. Seizure
42. Allergic reaction or anaphylaxis

The following events have been identified as "Anticipated Events" for subjects randomized to ablative therapy for this study.

Ablation Therapy or procedure related events:

1. Related to catheter insertion:
   a. Infection
   b. Sepsis
   c. Bleeding
   d. Bruising / ecchymosis
   e. Pain
   f. Hematoma (not requiring blood transfusion)
   g. Pseudoaneurysm
   h. A-V fistula
   i. Vessel trauma
   j. DVT
   k. Urinary tract infection
2. Pulmonary embolism
3. Pneumothorax
4. Hemothorax
5. Pleural effusion
6. Pneumonia

DELETED

1. Related to medications: as listed under Medical therapy with the following additions:
    a. Allergic reaction (skin rash, SOB)
    b. Hypotension
    c. Kidney damage
    d. Respiratory depression
    e. Left ventricular dysfunction
    f. Headache / nausea
    g. Bleeding from heparin
    h. Visual migraine
    i. Complete AV block
    j. Transient AV block
    k. Permanent AV block
    l. Volume overload
2. Related to catheter manipulation:
    a. Myocardial perforation
    b. Pericardial effusion
    c. Tamponade
    d. Myocardial infarction
    e. Other ischemic event
    f. Coronary artery spasm
    g. Coronary artery occlusion
    h. Coronary artery dissection
    i. Stroke
    j. TIA
    k. Peripheral thromboembolic event
    l. Cardiac thromboembolic event
    m. Air embolism
    n. Heart valve damage
    o. Clinically relevant sinus node block
    p. Clinically relevant AV node dysfunction
    q. Damage to Pacemaker or pacemaker leads
3. Related to ablation:
    a. Chest pain during energy delivery
    b. Pericarditis
    c. Radiation skin burn
    d. Radiation related cancers
    e. Phrenic nerve damage
    f. Pulmonary vein stenosis
    g. Pulmonary vein damage/dissection
    h. Pulmonary vein thrombus
    i. Pulmonary edema
    j. Pulmonary hypertension
    k. Esophageal atrial fistula
    l. New or worsened Gastroesophageal reflux
    m. Pleuritic chest pain
    n. Elevated creatinine phosphokinase (CPK)
    o. Temperature elevation
    p. Vasovagal reaction
    q. Esophagus or stomach erosion disorder, esophageal achalasia, esophageal ulcers, or stomach emptying disorder

Appendix A Adverse Events

The following events have been identified as "Anticipated Events" for subjects randomized to drug or ablation therapy for this study.

CATEGORY		ANTICIPATED EVENT		
CARDIOVASCULAR				
	1	Air embolism	17	Intraventricular conduction delay
	2	Bradycardia	18	Ischemia
	3	Cardiac arrest	19	Left ventricular dysfunction
	4	Cardiac thromboembolic event	20	Myocardial infarction
	5	Chest pain during energy delivery	21	Myocardial perforation
	6	Clinically relevant AV node dysfunction	22	Pacemaker damage
	7	Clinically relevant sinus node block	23	Pericardial effusion
	8	Complete/permanent AV block	24	Pericarditis
	9	Coronary artery dissection	25	Proarrhythmia; new or worsened arrhythmia
	10	Coronary artery occlusion	26	Prolonged QT
	11	Coronary artery spasm	27	Sustained VT
	12	Nonsustained VT	28	Tamponade
	13	Elevated creatinine phosphokinase (CPK)	29	Torsade des pointes
	14	Heart failure (Class I, II, III, IV)	30	Transient AV block
	15	Heart valve damage	31	Ventricular fibrillation
	16	Hypotension		
ENDOCRINE				
	32	Hyperthyroidism	33	Hypothyroidism
GENERAL				
	34	Allergic reaction (skin rash, SOB)	39	Radiation skin burn
	35	Bluish / gray skin tone	40	Skin rashes
	36	Fatigue	41	Temperature elevation
	37	Photosensitivity	42	Volume overload
	38	Radiation related cancers		
GASTROINTESTINAL				
	43	Constipation	48	Stomach disorder
	44	Diarrhea	49	Nausea
	45	Esophageal atrial fistula	50	Poor appetite
	46	Esophageal disorder	51	Unusual taste (metallic or other)
	47	Gastroesophageal reflux	52	Vomiting
			53	Abnormal liver functions
GENITOURINARY				
	54	Impotence	56	Renal failure
	55	Kidney damage	57	Urinary tract infection
INFECTIOUS				
	58	Infection	59	Sepsis
NEUROLOGIC				
	60	Alteration of color vision	68	Phrenic nerve damage
	61	Blindness	69	Seizure
	62	Blurred / double vision	70	Stroke
	63	Depression	71	Syncope
	64	Deteriorating vision	72	TIA
	65	Dizziness / light headedness	73	Unsteady gait / imbalance
	66	Headache	74	Vasovagal reaction
	67	Pain	75	Visual migraine

Appendix A Adverse Events (continued)

The following events have been identified as “Anticipated Events” for subjects randomized to drug or ablation therapy for this study.

CATEGORY		ANTICIPATED EVENT		
**PULMONARY**				
	76	Asthma exacerbation	84	Pulmonary hypertension
	77	Hemothorax	85	Pulmonary vein damage/dissection
	78	Lung toxicity	86	Pulmonary vein stenosis
	79	Pleural effusion	87	Pulmonary vein thrombus
	80	Pneumonia	88	Respiratory depression
	81	Pneumothorax	89	Shortness of breath/dyspnea
	82	Pulmonary edema	90	Wheezing
	83	Pulmonary embolism	91	Pleuritic chest pain
**PERIPHERAL VASCULAR**				
	92	A-V fistula	98	Peripheral edema
	93	Bleeding	99	Peripheral thromboembolic event
	94	Bleeding from heparin	100	Pseudoaneurysm
	95	Bruising / ecchymosis	101	Thromboembolic event
	96	DVT	102	Vessel trauma
	97	Hematoma		

Summary of Changes in the Protocol

The following changes listed in Table 1 represent clarification, version control, and corrective changes to the CABANA Protocol, Version 3.5, (22 November, 2013). These changes are approved by the CABANA Leadership Team, Mayo Clinic and Duke Clinical Research Institute (DCRI).

Table 1. Summary of Protocol Changes

Section/Page	Version 3.4.1 08 April, 2012	Version 3.5 22 November, 2013	Rationale
Title Page	Version: 3.4.1	Version: 3.5	
Title Page	Date: 08 April, 2012	Date:22 November, 2013	
Footer Throughout	08 April, 2012	22 November, 2013	
Contact	**Project Officer:**<br>Alice M. Mascette, MD<br>Phone:301-435-0477<br>Fax: 301-480-7971<br>alice.mascette@nih.gov	**Project Officer:**<br>Yves D. Rosenberg, MD, MPH<br>Phone: 301-435-1292<br>Fax: 301-480-3667<br>rosenbey@nih.gov	Personnel Change
Synopsis<br><br>DIAGNOSIS AND MAIN CRITERIA FOR INCLUSION	Subjects who have new onset or under-treated paroxysmal, persistent, or longstanding persistent AF who warrant therapy for their arrhythmia, with (1) documented atrial fibrillation (AF) ≥1 hour in duration, (2) ≥2 episodes over the preceding ***6*** months with ***electrocardiographic documentation*** of at least 1 episode; or 1 persistent or longstanding persistent episode of AF lasting more than 1 week, (3) are eligible for catheter ablation, (4) are eligible for ≥2 sequential rhythm control and/or ≥3 rate control drugs, and (5) are ≥65 yrs of age, or <65 yrs with **one or more** of the following risk factors for stroke: (Hypertension, Diabetes, Congestive heart failure, Prior stroke or TIA, LA size >5.0 cm or volume index ≥40 cc/m2, or EF ≤35).<br>See main protocol for complete inclusion/exclusion criteria.	Subjects who have new onset or under-treated paroxysmal, persistent, or longstanding persistent AF who **warrant therapy** for their arrhythmia, that<br>(1) Over the preceding **6** months have:<br>a) ≥2 paroxysmal episodes (***electrocardiographic documentation*** of at least 1 episode) lasting ≥1 hour in duration: (that terminate spontaneously within 7 days or cardioversion is performed within 48h of AF onset)<br>b) ***electrocardiographic documentation*** of 1 persistent AF episode: (sustained for ≥7 days or cardioversion is performed more than 48h after AF onset)<br>c) ***electrocardiographic documentation*** of 1 longstanding persistent AF episode: (continuous AF of duration >1 year).<br><br>(2) are eligible for catheter ablation,<br><br>(3) are eligible for $\geq$2 membrane active drugs and/or $\geq$2 rate control drugs, and<br>(4) are $\geq$65 yrs of age or <65 yrs with one or more risk factors for stroke	Clarification of entry criteria and required documentation for each of the 3 AF types.<br><br>To improve enrollment, the DSMB suggested the

		(hypertension, diabetes, heart failure, prior stroke or TIA or systemic emboli, Atherosclerotic vascular disease (previous MI, peripheral arterial disease or aortic plaque), or left atrial diameter ≥ 5.0 cm or left atrial volume ≥ 40 cc/m^2). Eligible subjects with persistent or long-standing persistent AF will require at least 1 documented episode. See main protocol for complete inclusion/exclusion criteria.	addition of CHADS-VASc criteria and decrease rate control drugs to ≥2.
STUDY HYPOTHESIS	The treatment strategy of percutaneous left atrial catheter ablation for the purpose of eliminating atrial fibrillation (AF) is superior to current state-of-the-art therapy with either rate control or rhythm control drugs for reducing total mortality (primary endpoint) and decreasing the composite endpoint of total mortality, disabling stroke, serious bleeding, and cardiac arrest (secondary endpoint) in subjects with untreated or incompletely treated AF warranting therapy.	The treatment strategy of percutaneous left atrial catheter ablation for the purpose of eliminating atrial fibrillation (AF) is superior to current state-of-the-art therapy with either rate control or rhythm control drugs for reducing a) the composite endpoint of total mortality, disabling stroke, serious bleeding, or cardiac arrest (primary endpoint; previously the key secondary endpoint) and decreasing total mortality (secondary endpoint; previously the primary endpoint) in subjects with untreated or incompletely treated AF warranting therapy.	Due to a lower than expected aggregated mortality rate, the DSMB suggested: change the primary endpoint of the trial from total mortality to the original key secondary endpoint consisting of the composite of death, disabling stroke, serious bleeding, or cardiac arrest
DURATION OF STUDY PARTICIPATION	Enrollment will occur over approximately 4 years, and subjects will be followed a minimum of 2 years.	Enrollment will occur over approximately 4 years, and subjects will be followed for an average of approximately 5 years.	Due to the accrual of patients at a slower rate than projected DSMB suggested: decreasing the sample size and extend the follow-up
NUMBER OF SUBJECTS	3000 with a 1:1 randomization ratio	2000-2200 with a 1:1 randomization ratio	
NUMBER OF SITES	Total number: 140<br>North American Sites: 100<br>Non-North American Sites: 40	Total number: approximately 180<br>North American Sites: approximately 120<br>Non-North American Sites: approximately 60	Possible number and distribution of sites needed to complete the Trial
PRIMARY ENDPOINT	Total mortality	Composite of: 1) total mortality, 2) disabling stroke, 3) serious bleeding, or 4) cardiac arrest.	DSMB suggested: Primary secondary a composite
SECONDARY	Cardiovascular death, Cardiovascular	Total mortality,	DSMB suggested:

OUTCOMES	death or disabling stroke, Arrhythmic death or cardiac arrest, Heart failure death, Freedom from recurrent AF, Cardiovascular hospitalization, Medical costs and resource use and cost effectiveness, Quality of life, Composite adverse events, LA size, morphology and function.	Total mortality or cardiovascular hospitalization, Total mortality or stroke or cardiovascular hospitalization,<br>Cardiovascular death,<br>Cardiovascular death or disabling stroke,<br>Arrhythmic death or cardiac arrest,<br>Heart failure death,<br>Freedom from recurrent AF,<br>Cardiovascular hospitalization,<br>Medical costs and resource use and cost effectiveness,<br>Quality of life,<br>Composite adverse events,<br>LA size, morphology and function.	Secondary endpoint revised
TOC /8		Updated to version 3.5	
1.0 /12<br>Introduction	An ablation trial evaluating overall mortality, conducted within a population at increased risk, will provide the most compelling evidence for guiding the therapy of this malady. The completion of the 60 patient CABANA Pilot Study provides solid evidence of the feasibility of this landmark study. The primary aim of the CABANA Trial is to test the hypothesis that the treatment strategy of percutaneous left atrial catheter ablation for the purpose of eliminating atrial fibrillation (AF) is superior to current state-of-the-art therapy with either rate control or rhythm control drugs for reducing total mortality	An ablation trial evaluating overall mortality and major AF related events, conducted within a population at increased risk, will provide the most compelling evidence for guiding the therapy of this malady. The completion of the 60 patient CABANA Pilot Study provides solid evidence of the feasibility of this landmark study. The primary aim of the CABANA Trial is to test the hypothesis that the treatment strategy of percutaneous left atrial catheter ablation for the purpose of eliminating atrial fibrillation (AF) is superior to current state-of-the-art therapy with either rate control or rhythm control drugs for reducing: 1) total mortality, disabling stroke, serious bleeding, or cardiac arrest as the primary endpoint and 2) total mortality as the secondary endpoint in patients with untreated or incompletely treated AF.	DSMB suggested revisions to the primary and secondary outcomes
1.1.2 /13<br>Progression of AF	There is also reason to anticipate an increasing impact of this arrhythmia on mortality.	There is also reason to anticipate an increasing impact of this arrhythmia on mortality, stroke, bleeding and cardiac arrest.	DSMB suggested revisions
1.4 /17	These studies taken together, along with	These studies taken together, along with	DSMB suggested

Rationale for Maintaining Sinus Rhythm	data from population-based studies, provide the rationale for a large, multi-center trial to prospectively examine the impact of sinus rhythm on overall mortality in patients with AF.	data from population-based studies, provide the rationale for a large, multi-center trial to prospectively examine the impact of sinus rhythm on total mortality, disabling stroke, serious bleeding and cardiac arrest in patients with AF.	revisions
1.8 /19 Significance of the Trial	Scientifically, the trial will determine whether the attainment of normal sinus rhythm is a mortality advantage.	This study will also assess the role of earlier therapy for AF and the related utility of ablation as first line therapy in patients warranting treatment. Scientifically, the trial will determine whether the attainment of normal sinus rhythm is a mortality and stroke advantage.	DSMB suggested revisions
2.0 /20 Objectives		**Changes to the Protocol from the Original Study Design** Although CABANA was originally mandated to be a mortality trial, a careful assessment of the progress of the trial was undertaken by the study leadership in early 2013. Completely blinded to any treatment-specific outcome data, the two major issues addressed by the leadership group were (1) a lower than expected aggregated mortality rate, and (2) accrual of patients at a slower rate than projected. Careful consideration of these issues led to a decision to (a) change the primary endpoint of the trial from total mortality to the original key secondary endpoint consisting of the composite of death, disabling stroke, serious bleeding, or cardiac arrest, and (b) reduce the sample size to a number that was consistent with the new primary endpoint and more realistically achievable within the funding period of the trial. There may still be a mortality difference between treatment groups. This was not revealed in the interim review. These changes will be highlighted in the sections of the protocol that follow.	Clarity and overview of the protocol revisions

2.1 /20<br>Primary Objective and Hypothesis	The primary hypothesis of the CABANA trial is that the treatment strategy of percutaneous left atrial catheter ablation for the purpose of eliminating atrial fibrillation (AF) is superior to current state-of-the-art medical therapy with either rate control or rhythm control drugs for reducing total mortality (primary endpoint) and decreasing the composite endpoint of total mortality, disabling stroke, serious bleeding, or cardiac arrest (key secondary endpoint) in patients with untreated or incompletely treated AF warranting therapy. It is anticipated that treatment with percutaneous left atrial catheter ablation will reduce mortality ≥30% compared to drug therapy. All endpoints will be carefully assessed and analyzed on an intention to treat basis.	The primary hypothesis of the CABANA trial is that the treatment strategy of percutaneous left atrial catheter ablation for the purpose of eliminating atrial fibrillation (AF) is superior to current state-of-the-art medical therapy with either rate control or rhythm control drugs for decreasing the incidence of the composite endpoint of total mortality, disabling stroke, serious bleeding, or cardiac arrest (primary endpoint) and reducing total mortality (secondary endpoint) in patients with untreated or incompletely treated AF warranting therapy. It is anticipated that treatment with percutaneous left atrial catheter ablation will reduce the incidence of this endpoint by ≥30% compared to drug therapy. To properly interpret this composite endpoint, the incidence of each of the individual components will also be descriptively examined to assess its relative contribution to the overall composite outcome. The primary endpoint and all secondary endpoints will be carefully assessed and analyzed on an intention to treat basis.	DSMB suggested revisions to the primary outcomes
2.2 /20<br>Secondary Endpoints/Objectives	1. Total mortality, disabling stroke, serious bleeding, or cardiac arrest<br>2. Total mortality or cardiovascular hospitalization	1. Total mortality<br>2. Total mortality or cardiovascular hospitalization<br>3. Total mortality, stroke, or CV hospitalization (for heart failure or acute ischemic events)	DSMB suggested revisions to the secondary outcomes
2.2.1 /21<br>~~Composite Morbidity~~ / Total Mortality	This trial will determine whether catheter ablation for the elimination of AF has an impact on the composite end point of total mortality, disabling stroke, serious bleeding, or cardiac arrest, when compared to drug therapy. This is the key secondary endpoint. The hypothesis regarding this endpoint is that catheter ablation for AF will result in a significant	Because of the vital importance of assessing the impact of left atrial catheter ablation on total mortality, this endpoint (which is a component of the primary endpoint) will be a specific secondary endpoint in the trial.<br>*Hypothesis:* Catheter ablation for AF will reduce total mortality by ≥30% compared to state-of-the-art pharmacologic therapy.	DSMB suggested revisions to the secondary outcomes

	(>25%) reduction in the incidence of this composite endpoint. To detect the relative contribution of these component events, the incidence of each individual component will also be descriptively examined to aid in the interpretation of the composite outcome. Because of a mechanistic concordance of events, arrhythmic death and cardiac arrest will be considered together as a composite secondary endpoint. Cardiovascular death and disabling stroke will be similarly grouped.		
2.2.2 /21 Composite Mortality / Cardiovascular Hospitalization	By reducing the recurrence of AF, the proposed therapies should also reduce cardiovascular hospitalization. Additional secondary endpoints, including cardiovascular hospitalization and the composite of total mortality and cardiovascular hospitalization, will therefore be examined. *Hypothesis*: Catheter ablation for AF will be significantly (>=25%) more effective than pharmacologic therapy, in reducing cardiovascular hospitalization and the composite of total mortality or cardiovascular hospitalization.	By reducing the recurrence of AF, the proposed therapies should also reduce cardiovascular hospitalization. Additional secondary endpoints, including cardiovascular hospitalization and the composite of total mortality and cardiovascular hospitalization, will therefore be examined. The composite of total mortality, stroke or cardiovascular hospitalization will also be assessed. *Hypothesis*: Catheter ablation for AF will be significantly (≥25%) more effective than pharmacologic therapy, in reducing cardiovascular hospitalization, the composite of total mortality or cardiovascular hospitalization, and the composite of total mortality, stroke or cardiovascular hospitalization.	DSMB suggested revisions to the secondary outcomes
2.2.4 /21 Freedom from Recurrent Atrial Fibrillation	Time to second AF recurrence and AF burden will also be established.	Time to second AF recurrence and AF burden will also be established. Freedom from AF after each ablation performed in an individual subject will be separately tracked.	Clarification as it relates to the tracking of recurrent AF for ablation therapy
3.1 /22 Inclusion Criteria	1. Have paroxysmal AF episodes ≥1 hour in duration; with ≥2 episodes over the preceding 6 months with ***electrocardiographic documentation***	1. Over the preceding **6** months have a) paroxysmal AF episodes (that terminate spontaneously within 7 days or cardioversion is performed within 48h of	Clarification of entry criteria and required documentation for each of the 3 AF types.

	of at least 1 episode; or 1 persistent or longstanding persistent episode of AF lasting more than 1 week.<br><br>2. Warrant active therapy beyond simple ongoing observation<br><br>3. Be eligible for catheter ablation and ≥2 sequential rhythm control and/or ≥3 rate control drugs.<br>4. Prior stroke, TIA or systemic emboli, Atherosclerotic vascular disease (previous MI, peripheral arterial disease or aortic plaque), LA size >50<br>NOTE: providing they remain realistically eligible for ≥2 membrane active drugs and/or ≥3 rate control agents.	AF onset): that is ≥1 hour in duration with ≥2 episodes with ***electrocardiographic documentation*** of at least 1 episode; or b)***electrocardiographic documentation*** of 1 persistent AF episode: (sustained for ≥7 days or cardioversion is performed more than 48h after AF onset) or c) ***electrocardiographic documentation*** of 1 longstanding persistent AF episode: (continuous AF of duration >1 year).<br>2.Warrant active therapy (within the past 3 months) beyond simple ongoing observation<br>3. Be eligible for catheter ablation and ≥2 sequential rhythm control and/or ≥2 rate control drugs.<br>4. Prior stroke, TIA or systemic emboli, Atherosclerotic vascular disease (previous MI, peripheral arterial disease or aortic plaque), LA size >50<br>NOTE: providing they remain realistically eligible for ≥2 membrane active drugs and/or ≥2 rate control agents. Patients receiving new drug therapy initiated within the previous 3 months may continue that therapy if randomized to the drug therapy arm.	To improve enrollment, the DSMB suggested the addition of CHADS-VASc criteria and decrease rate control drugs to >2.<br><br>Protocol clarification
3.2 /23<br>Exclusion Criteria	4. More than one week of amiodarone treatment in the past 3 months<br>5. An efficacy failure of full dose amiodarone treatment >12 weeks duration at any time.<br>NOTE: Prior ablation of the cavo-tricuspid isthmus alone is not an exclusion if the patient develops subsequent recurrent AF. Planned atrial flutter ablation in combination with the left atrial ablation is not an exclusion.	4. REMOVED<br>4. An efficacy failure of full dose amiodarone treatment >8 weeks duration at any time.<br>NOTE: Exclusion Criterion #3 includes failed membrane active antiarrhythmic drugs started within 3 months prior to enrollment. Prior ablation of the cavo-tricuspid isthmus alone is not an exclusion if the patient develops subsequent recurrent AF. Planned atrial flutter ablation in combination with the left atrial ablation is not an exclusion	To enhance enrollment<br><br>Protocol clarification
4.1 /23<br>Trial Design and	This multi-center study will randomize 3000 patients in a 1:1 fashion to a	This multi-center study will randomize 2000-2200 patients in a 1:1 fashion to a	DSMB suggested to decrease the sample

Time Line	strategy of catheter ablation vs. state-of-the-art drug therapy for either rate or rhythm control, as outlined in Figure 2. Each will have untreated or incompletely treated AF, which in the opinion of the investigator warrants therapy. CABANA enrollment will occur over approximately 3 years, beginning in the 3rd quarter of 2009. All CABANA patients will be followed a minimum of 2 years. Assuming criteria for early termination are not reached, the major trial results are expected to be reported in 2015.	strategy of catheter ablation vs. state-of-the-art drug therapy with either rate or rhythm control, as outlined in Figure 2. Each patient will have untreated or incompletely treated AF, which in the opinion of the investigator warrants therapy. CABANA enrollment will occur over approximately 4 years. All CABANA patients will be followed an average of approximately 5 years. Assuming criteria for early termination are not reached, the major trial results are expected to be reported in early 2018.	size
4.2 /24<br>Screening and Pre-Randomization Procedures	The principal investigator or documented members of the research team will discuss the underlying rationale for the study<br><br>Work Productivity and Activity Impairment Instrument (WPAI), Stanford Presenteeism scale will be collected	The principal investigator or documented members of the research team approved by local Institutional Review Board (IRB) or Ethics Committee (EC) will discuss the underlying rationale for the study,<br>Work Productivity and Activity Impairment Instrument (WPAI), Stanford Presenteeism scale and Mayo AF Symptom Index (MAFSI)) will be collected	Protocol clarification
4.4 /24<br>Post Randomization Procedures	and patients followed for a minimum of approximately 2 years.	and patients followed for an average of approximately 5 years.	DSMB suggested Extension of the follow-up
4.4.1 /25<br>Baseline testing	up to 375 patients randomized to drug therapy will be asked to undergo one research CT/MR scan	up to 150 patients randomized to drug therapy will be asked to undergo one research CT/MR scan	Recalculation of numbers to meet objectives
4.4.2 /25<br>Patient Follow Up	A brief Follow up Questionnaire capturing atrial fibrillation severity and symptoms, work productivity/activity/presenteeism will be collected at 6, 18, 30, and 42 months post randomization.<br><br>Follow-up visits/calls should be completed within 30 days +/- of the due date<br><br>asking 5 brief questions and Mayo Atrial Fibrillation Symptom Index (MAFSI) will	REMOVED<br><br>Follow-up visits/calls at 3 and 6 months should be completed within 30 days +/- of the due date<br>Follow-up visits/calls at 12 months and every 6 months thereafter should be completed within 60 days +/- of the due date (Ex: 12 month visit: completed between 300 days and 420 days of randomization).<br>asking 5 brief questions and Mayo Atrial Fibrillation Symptom Index (MAFSI) will be collected by the Site Coordinator at 3	Extension of the follow-up window to allow more flexibility<br><br>Reduction in number of surveys/patient to reduce subject burden

	be collected by the Site Coordinator at each follow up visit throughout the trial and entered into the e-CRF.<br><br>All patients enrolled will receive a single 'CABANA Box' recording system to be used for both patient activated event monitoring (throughout the trial), autodetect/autocapture (AD:AC) event monitoring (one 24 hour period/month) and full disclosure Holter monitoring for 96 hours every 6 months throughout the study.<br><br>The 375 drug therapy patients	and 12 months following randomization during the first year and yearly thereafter throughout the trial and entered into the e-CRF.<br>After enrollment, subjects will either receive a single 'CABANA Box' recording system to be used throughout the entire study, or will be followed using ambulatory event and Holter ECG monitoring as generated during the course of routine clinical care as dictated by their attending physician.<br>At sites where the 'CABANA Box' has received appropriate approval, all patients enrolled will receive a single 'CABANA Box' recording system to be used for both patient activated event monitoring (throughout the trial), 24 hour autodetect/autocapture (AD:AC) event monitoring *and* 96 hour full disclosure Holter monitoring throughout the study.<br>During year one, subjects will be asked to record their heart rhythm each month. After the first year, they will be asked for a 24 hour recording twice a year and a 96 hour recording twice a year.<br>Follow up monitoring with an alternative system will be required in centers unable to use the "CABANA Box".<br><br>The 150 drug therapy patients<br><br>After completion of the 60 month follow-up, subjects will be asked to extend their participation. If agreed upon, subjects will be asked about their current state of health and any clinical events every 6 months by telephone until the last subject enrolled reaches approximately 36 months follow-up.	The Medicomp monitoring system will only be utilized at Institutions or within countries where appropriate approvals have been obtained. Routine monitoring based on clinical care will be recorded within the trial database records.<br><br>To decrease subject burden, use of the heart monitor (CABANA Box) has been reduced.<br><br>Recalculation of numbers to meet objectives.<br><br>Extended follow-up to achieve an approximate trial wide follow-up of an average of 5 years.
4.4.3 /27		Updated Schedule of Assessment	Clarification

5.4 /32<br>Guidelines for Anti-thrombotic Therapy	(warfarin, dabigatran)<br><br>These agents may be used as replacement therapy for warfarin on approval of Innovative Antithrombotic Therapies/Executive Committees.	(warfarin, dabigatran, rivaroxiban or apixaban)<br>These agents may be used as replacement therapy for warfarin on approval of local regulatory agencies and the Innovative Antithrombotic Therapies/Executive Committees.	Innovative Drug Therapy committee approved new anticoagulation drugs for use within CABANA
6.1 /33<br>Primary Endpoints	The primary endpoint event of mortality, will be adjudicated by an independent Clinical Events Committee (CEC) and the most proximate cause of that event established. The CEC will also confirm whether a death is cardiac/vascular/non-cardiovascular in origin; as well as witnessed/un-witnessed; or sudden/non-sudden. Cardiac mortality will be further categorized as tachyarrhythmic, bradyarrhythmic, heart failure, or due to other cardiac causes using the events adjudication form.	The primary endpoint is the composite of total mortality, disabling stroke, serious bleeding or cardiac arrest. All components will be adjudicated by an independent Clinical Events Committee (CEC) and the most proximate cause of the event established. A Disabling Stroke will be considered present using a modification of a Rankin Stroke score [100], and will be adjudicated by a Neurologic Events Committee. Serious (or Life-threatening) bleeding will be considered present using a modification of the GUSTO bleeding Scale adapted for use in catheter ablation [101]. These events will be tracked regardless of treatment randomization. The definition for each of these events is listed in the CEC Charter. When there is disagreement between the CEC and the principal investigator, the CEC's decision will be considered final. Procedures for adjudicating events are described in the CEC Charter, which is available upon request. The CEC will also confirm whether a death is cardiac/vascular/non-cardiovascular in origin; as well as witnessed/un-witnessed; or sudden/non-sudden. Cardiac mortality will be further categorized as tachyarrhythmic, bradyarrhythmic, heart failure, or due to other cardiac causes using the events adjudication form. Hospitalization will also be tracked with specific reason for admission (heart failure, acute ischemic	DSMB suggested revisions to the primary outcomes<br><br>Placement within protocol changed<br><br>Placement within protocol changed

		event, etc) determined by the site principal investigator as reported in the eCRF.	
6.2 /34<br>Secondary Endpoints & Safety Endpoints	Secondary endpoint events, including composite endpoints of total mortality, disabling stroke, serious bleeding, or cardiac arrest will be confirmed and adjudicated in a similar manner by the CEC.<br>A Disabling Stroke will be considered present using a modification of a Rankin Stroke score [100], and will be adjudicated by a Neurologic Events Committee. Serious (or Life-threatening) bleeding will be considered present using a modification of the GUSTO bleeding Scale adapted for use in catheter ablation [101]. These events will be tracked regardless of treatment randomization. The definition for each of these events is listed in the CEC Charter. When there is disagreement between the CEC and the principal investigator, the CEC's decision will be considered final. Procedures for adjudicating events are described in the CEC Charter, which is available upon request.<br><br>While hospitalization for AF in both treatment arms	Secondary endpoint events, including total mortality and a composite of total mortality or cardiovascular hospitalization will be confirmed.<br><br>REMOVED<br><br>While hospitalization for any atrial fibrillation, atrial flutter or atrial tachycardia in both treatment arms	DSMB suggested revisions to the secondary outcomes. Secondary endpoints as listed will be confirmed but not adjudicated by the CEC.
7.1.1 /35<br>Anticipated Adverse Events	A listing of 'anticipated/expected' events can be found in Appendix A. An unanticipated/unexpected adverse event is any adverse event that has not been reported in previous studies, published literature, or product labeling (see appendix A).	A listing of commonly occurring 'anticipated/expected' events in the population being studied can be found in Appendix A. An unanticipated/ unexpected adverse event is any adverse event that has not been reported in previous studies, published literature, product labeling, or which is not anticipated in the population being studied (see appendix A).	The text had been updated to provide additional guidance and clarifications to the investigators for the assessment and reporting of adverse events.
7.3 /36	DCRI will report all unanticipated/	DCRI will report all unanticipated/	

Serious Adverse Event Reporting	unexpected adverse events to Mayo Clinic and the DSMB chair	unexpected study related serious adverse events to Mayo Clinic, NHLBI and the DSMB chair	
7.4.1 /37 Expedited events include	**Events of Interest (EOI)**: Drug or ablation therapy or ablation procedure related events (index and/or follow-up): All events that resulted in death, pro-arrhythmic events, myocardial perforation / tamponade requiring intervention, esophageal atrial fistula, and/or severe pulmonary vein stenosis that were life threatening or classified as severe in nature.	**Events of Interest (EOI)**: Drug or ablation therapy or ablation procedure related events (index and/or follow-up): The related events that resulted in death, and the following events if they are life-threatening or severe in nature; pro-arrhythmic events, myocardial perforation / tamponade requiring intervention, esophageal atrial fistula, and/or severe pulmonary vein stenosis that were life threatening or classified as severe in nature.	
7.6.2 /38 CABANA Reporting of Drug or Device Adverse Events	For CABANA trial purposes, adverse events will be reported through the eCRF submission process designed to facilitate notification to DCRI Safety Surveillance and/or their designee.<br><br>The DCRI Safety Surveillance Medical Monitor (a physician trained and experienced in safety reporting) will review all SAEs for potential expected/unexpected; anticipated/unanticipated status. The decision regarding ultimate classification will be made by **individuals within CABANA Leadership** with expertise in antiarrhythmia and ablation therapies and clinical trial experience.<br>DCRI Safety Surveillance will notify the NIH within 1-2 business days of an unexpectedness/unanticipated event<br><br>DCRI Safety Surveillance will notify the NIH within 5 business days of all reported Events of Interest (as identified above).	For CABANA trial purposes, adverse events will be reported through the eCRF submission process designed to facilitate notification to DCRI Safety Surveillance and/or their designee. DCRI Safety Surveillance will review and code all SAEs.<br>The Safety Surveillance Medical Monitor (a physician trained and experienced in safety reporting) will review Events of Interest, and unexpected/unanticipated SAEs related to ablation therapy or CABANA approved rate or rhythm control drugs. The decision regarding ultimate classification will be made by **individuals within CABANA Leadership** with expertise in antiarrhythmia and ablation therapies and clinical trial experience.<br>DCRI Safety Surveillance will notify the NHLBI and Mayo within 1-2 business days of an unexpectedness/unanticipated event<br>DCRI Safety Surveillance will notify the NHLBI within 5 business days of all reported Events of Interest (as identified above).	The text had been updated to clarify the process followed by DCRI Safety Surveillance for the assessment of adverse events.
8.1 /39	Second, important secondary endpoints	Second, important secondary endpoints	Reporting of new

Sample Size and Power Considerations	were considered, including the key composite endpoint of death, disabling stroke, serious bleeding, or cardiac arrest.<br><br>crossovers of drug-arm patients to receive ablation will have the impact of reducing the mortality rate of patients<br><br>If we assume that patients who cross over receive a similar reduction in mortality as the benefit hypothesized for patients initially randomized to ablation, the 3.5 year control-arm event rate will	were considered.<br>As described in Section 2.0, the study was originally designed with total mortality as the primary endpoint. However, in early 2013 a careful assessment of the progress of the trial was undertaken by the study leadership. Completely blinded to any treatment-specific outcome data, the two major issues addressed by the leadership group were (1) a lower than expected aggregated mortality rate, and (2) accrual of patients at a rate much slower than projected. Careful consideration of these issues led to a decision to (a) change the primary endpoint of the trial from total mortality to the key secondary endpoint consisting of the composite of death, disabling stroke, serious bleeding, or cardiac arrest, and (b) reduce the sample size to a number that was consistent with the new primary endpoint. The following paragraphs, which outlined the key considerations in determining the original sample size, are also relevant for the revised sample size.<br><br>the most reliable estimates of mortality and other endpoint events applicable to the drug arm of CABANA<br>With the original secondary endpoint elevated to become the primary endpoint, the incidence of the new primary endpoint is expected to be higher than the mortality rates.<br><br>crossovers of drug-arm patients to receive ablation will have the impact of reducing the event rate of patients<br><br>REMOVED	calculations based on revised primary and secondary endpoints

	drop from 15% to approximately 12-13%. The treatment effect will also be reduced.<br>the method of Schoenfeld [104].<br><br>To provide an adequate number of patients in the trial that will be relatively robust under various assumptions regarding the control-arm event rates and the magnitude of the treatment benefit, 3,000 patients will be enrolled. This number will provide 90% power for detecting a 30% mortality reduction, allowing for a 2% loss to follow-up. Thus, the study will have high power for detecting an important benefit if the control arm event rate is consistent with or even slightly lower than expected based on previous studies. This number will also provide power > 80% for detecting a 25% reduction in mortality with ablation if the 3.5-year event rate in the drug arm is 13% or higher. Thus we have good power for detecting a more conservative estimate of the mortality benefit if the control arm event rate is consistent with previous studies. A 25-30% mortality reduction will be highly important from a clinical and public health standpoint, given the large population of patients in this country and throughout the world who suffer from AF. Since the event rates for the key composite secondary endpoint will be higher than the primary mortality endpoint, 3000 patients will also provide >90% power for detecting a 25% reduction in the important secondary endpoint consisting of the composite of total mortality, disabling stroke, serious bleeding, or cardiac arrest.	the method of Schoenfeld [104] developed for the proportional hazards model.<br><br>To provide an adequate number of patients in the trial that will be relatively robust under various assumptions regarding the control-arm event rates and the magnitude of the treatment benefit, 2,000-2,200 patients will be enrolled. With a minimum follow-up of 3 years (amounting to an average follow-up of approximately 5 years), 2200 patients will provide 90% power for detecting a 30% reduction in the new primary endpoint, and 2000 patients will provide 80% power, assuming a 3-year event rate in the drug arm of 12% and allowing for a 2% loss to follow-up. Thus, the study will have high power for detecting an important benefit if the control arm event rate is consistent with the rate expected based on previous studies. A sample size in this range will also provide acceptable power (86% with 2200 patients and 82% with 2000 patients) for detecting a 25% reduction with ablation in the primary endpoint if the 3-year drug arm event rate is 15%. Thus we will have good power for detecting a more conservative estimate of the benefit in the composite endpoint if the control arm event rate is higher, but still consistent with previous studies. A 25-30% reduction in primary events will be highly important from a clinical and public health standpoint, given the large population of patients in this country and throughout the world who suffer from AF.	

		This number of patients (2,000-2,200) will also provide adequate power for detecting a 25% reduction in other important secondary composite endpoints listed in section 2.2 such as the endpoints that involve cardiovascular hospitalization where the incidence is expected to be higher than for the primary endpoint.	
8.2.1 /41 Analysis of the Primary Endpoint	The log-rank test will be the primary analytic tool for comparing mortality differences between the two therapies. Kaplan-Meier estimates of cumulative mortality rates as a function of follow-up time will be calculated and displayed. Relative risks will be expressed as hazard ratios with 95% confidence intervals generated using the Cox proportional hazards model. Supplementary analysis involving covariate adjustment will be performed with the Cox model. Such adjustment will be limited to a relatively small, prospectively defined set of patient characteristics that are known *a priori* to have a strong prognostic relationship with mortality. The covariate-adjusted analysis will serve as a prelude to supplementary analyses examining differential treatment effects. The covariates will include age, sex, race, heart failure class, presence/absence of structural heart disease, whether the patients' AF is paroxysmal, persistent or long-standing persistent, duration of AF, presence/absence of hypertension, and ejection fraction. Cox model analyses may also be performed using appropriate groupings of sites as a stratification factor.	The log-rank test will be the primary analytic tool for comparing outcome differences between the two therapies. Kaplan-Meier estimates of cumulative event rates as a function of follow-up time will be calculated and displayed. Relative risks will be expressed as hazard ratios with 95% confidence intervals generated using the Cox proportional hazards model. Supplementary analysis involving covariate adjustment will be performed with the Cox model. Such adjustment will be limited to a relatively small, prospectively defined set of patient characteristics that are known *a priori* to have a strong prognostic relationship with the primary endpoint. The covariate-adjusted analysis will serve as a prelude to supplementary analyses examining differential treatment effects. The covariates will include age, sex, race, heart failure class, presence/absence of structural heart disease, whether the patients' AF is paroxysmal, persistent or long-standing persistent, duration of AF, presence/absence of hypertension, and ejection fraction. Cox model analyses may also be performed using appropriate groupings of sites as a stratification factor.	Language revised based on change in primary and secondary endpoints.
8.2.2 /41 Analysis of Secondary Endpoints	Secondary endpoints including the important mortality/morbidity composite endpoint consisting of death, disabling	Secondary endpoints, including total mortality and secondary endpoints 2 through 9 listed in Section 2.2, will all	Language revised based on change in primary and secondary endpoints.

	stroke, serious bleeding, or cardiac arrest, and secondary endpoints 2 through 8 listed in Section 2.2 all involve time-to-event analyses and thus will be analyzed similar to the primary endpoint using the log-rank test, Cox model, and Kaplan-Meier event rate estimates	involve time-to-event analyses and thus will be analyzed similar to the primary endpoint using the log-rank test, Cox model, and Kaplan-Meier event-rate estimates.	
8.2.6 /43<br>Interim Analyses	interpretation of statistical significance associated with treatment comparisons of key study endpoints will be guided using the group sequential stopping boundaries outlined above.<br>After approximately 25% of the total events have occurred, conditional power for the primary treatment comparison	interpretation of statistical significance associated with treatment comparisons will be guided using the group sequential monitoring boundaries outlined above.<br>After approximately 50% of the total events have occurred, conditional power for the primary treatment comparison	Language revised based on change in primary and secondary endpoints.
8.2.7 /44<br>Multiple Comparisons	overall level of significance for the assessment of the primary mortality endpoint will be 0.05,	overall level of significance for the assessment of the primary composite endpoint will be 0.05,	Language revised based on change in primary and secondary endpoints.
11.1 /47<br>Site Selection and Monitoring	As part of a concerted effort to follow the study in a detailed and orderly manner in accordance with established principles of Good Clinical Practice and applicable regulations, a DCRI study monitor or their designee will visit study sites regularly. They will maintain frequent telephone and written communication.	As part of a concerted effort to follow the study in a detailed and orderly manner in accordance with established principles of Good Clinical Practice and applicable regulations, the Monitoring Plan is being revised with Executive Committee approval. A DCRI study monitor or their designee will no longer perform on-site visits to Active study sites regularly and throughout the study. Rather they will maintain frequent telephone and written communication, as well as perform on-site visits to a subset of sites to ensure data integrity.	Accurate communication of the Site monitoring process.
18.0 /50<br>Informed Consent	obtain written informed consent. The underlying rationale for the study, the procedures to be followed, the potential benefits, risks, alternatives, and other issues mandated by the consent process will be fully disclosed.	obtain written informed consent on an informed consent form (ICF) approved by the same IRB/EC responsible for approval of this protocol. The informed consent document will conform to FDA regulations in 21 CFR Part 50, and/or to the national requirements for informed consent. It must include all elements required by law, local regulations, GCP and International Conference on	Language revised/ inserted for regulatory compliance

	Written informed consent will be documented on an informed consent form (ICF) approved by the same IRB responsible for approval of this protocol, The ICF will conform to FDA regulations in 21 CFR Part 50, and to the institutional requirements for informed consent and applicable regulations.	Harmonization guidelines and study specific procedures. The underlying rationale for the study, the procedures to be followed, the potential benefits, risks, alternatives, and other issues mandated by the consent process will be fully disclosed. If new information becomes available during the course of the trial that may be relevant to the subject's consent, the Informed Consent Form will be revised and the revised version will be submitted for EC/IRB approval before use.<br>REMOVED	
19.0 /51 Confidentiality of Subjects	Subject information collected in this study<br>will comply with the standards for protection of privacy of individually identifiable health information as promulgated in the Health Assurance Portability and Accountability Act and as mandated in Title 45 CFR, Parts 160 and 164.<br>All records will be kept confidential and the subject's name will not be released by study staff at any time.<br>Subject records will not be released to anyone other than DCRI and/or their designee, and responsible regulatory authorities when requested.<br>In all cases, caution will be exercised to assure the data are treated confidentially and that the subject's privacy is protected.	Subject information collected in this study<br>REMOVED<br>and all records will be kept confidential and the subject's name will not be released by study staff at any time.<br>REMOVED<br>When requested patient medical records may be examined by authorized monitors (DCRI and/or their designee) or Clinical Quality Assurance auditors appointed by the sponsor, by appropriate IRB / IEC members and by domestic and foreign regulatory authorities. In all cases, caution will be exercised to assure the data are treated confidentially and that the subject's privacy is protected. Furthermore; for clinical trial sites located in the US, the NHLBI has issued CABANA a Certificate of Confidentiality to protect the privacy of research subjects	Language revised/ inserted for regulatory compliance. Please note; for clinical trial sites located in the US, CABANA now has a Certificate of Confidentiality

		by withholding their identifiable information from all persons not connected with this research.	
22.0 /52<br>IRB / EC Committee Review	The appropriate IRB/EC must approve the protocol and informed consent documents, agree to monitor the conduct of the study, and agree to review study progress periodically, at intervals not to exceed 1 year. The investigator will provide DCRI or their designee with documentation that the IRB has approved the study *before* the study may begin. In addition, the investigator must provide the following documentation to DCRI or their designee.<br>1. IRB annual reapproval of the protocol, per current Title 21 CFR 312.66 regulations and 1997 International Conference on Harmonization guidelines.<br>2. IRB approval of revisions to the informed consent documents or any amendments to the protocol. Any revisions to the protocol that may increase subject risk exposure must be approved prior to implementation. Administrative changes (such as a change in address or phone number) must be sent to IRBs/Ethics Committees but do not require their approval. The investigator will provide DCRI or their designee with documentation of all approvals.	This study will be initiated only after all required documentation has been reviewed and approved by the respective IRB/EC and competent authority (CA) according to national and international regulations.<br>The investigator will provide DCRI or their designee with<br>the study approval documentation before the study may begin. The same is applicable for the implementation of changes introduced by amendments.<br>Where applicable, the investigator must also provide to DCRI and/or their designee the following documentation:<br>1. A copy of IRB annual re-approval of the protocol per current Title 21 CFR 312.66 regulations and 1997 International Conference on Harmonization guidelines.<br>2. IRB approval of revisions to the informed consent documents.<br>Administrative changes (such as a change in address or phone number) must be sent to IRBs/Ethics Committees but do not require their approval.<br>3. The investigator must submit periodic status reports to their EC as required, as well as notification of completion of the study and a final report where applicable.<br>4. The investigator will provide DCRI or their designee with documentation of all approvals.	Language revised/ accuracy and regulatory compliance
Appendix A /61		Added<br>26. Persistent PFO/iatrogenic ASD	The list of Anticipated Events has been updated according to the most recent clinical data.
Appendix B/63		Biosense Webster ThermoCool® SF<br>Medtronic Cryocath LP Arctic Front®	Updated approved catheter list

		Medtronic Cardiac Ablation System	
Appendix C /64<br>CABANAgene<br>Appendix A:<br>CABANA<br>Inclusion/Exclusion<br>Criteria	Sample size 3000<br><br>2 year follow-up	Revised accordingly in sections referring to sample size (2,000-2,200) and duration follow-up (an average of approximately 5 years).<br><br>Revised per CABANA protocol Version3.5	Reflection of the changes within the main CABANA Trial population, follow-up timeline, and Inclusion/Exclusion
