# Supplementary material for: Catheter Ablation vs Drug Therapy in Patients With Atrial Fibrillation and Nonmodifiable Recurrence Risk Factors: A Secondary Analysis of the CABANA Randomized Clinical Trial
Source: JAMA Netw Open. 2025 Aug 21;8(8):e2528124. doi: 10.1001/jamanetworkopen.2025.28124 (PMC12371518; doi:10.1001/jamanetworkopen.2025.28124)
Supplement: Supplement 2. — eFigure 1. Time-Dependent ROC Curve Analysis of Different Recurrence Prediction Models eFigure 2. Kaplan-Meier Curves for AF Recurrence in the Ablation Arm eFigure 3. Time-Dependent ROC Curve Analysis of Different Recurrence Prediction Models in Patients With Echocardiograms eFigure 4. Propensity Score–Weighted Hazard Ratios of End Points by Intention-to-Treat eFigure 5. Adjusted Hazard Ratios of End Points by Intention-to-Treat Among Patients With Different Numbers of NMRRFs eFigure 6. Propensity Score–Weighted Hazard Ratios of End Points by Intention-to-Treat Among Patients With Different Numbers of NMRRFs eFigure 7. Quality-of-Life Outcomes by MAFSI Severity Scoring eTable 1. Emulated Target Trial Framework for Prognosis Among Patients With AF and Fewer Than 3 NMRRFs eTable 2. Emulated Target Trial Framework for Prognosis Among Patients With AF and 3 or More NMRRFs [file jamanetwopen-e2528124-s002.pdf]

Supplemental Online Content

Wang Z, Wu Y, Jiang C, et al. Catheter ablation vs drug therapy in patients with atrial fibrillation and nonmodifiable recurrence risk factors: a secondary analysis of the CABANA randomized clinical trial. *JAMA Netw Open*. 2025;8(8):e2528124. doi:10.1001/jamanetworkopen.2025.28124

eFigure 1. Time-Dependent ROC Curve Analysis of Different Recurrence Prediction Models
eFigure 2. Kaplan-Meier Curves for AF Recurrence in the Ablation Arm
eFigure 3. Time-Dependent ROC Curve Analysis of Different Recurrence Prediction Models in Patients With Echocardiograms
eFigure 4. Propensity Score–Weighted Hazard Ratios of End Points by Intention-to-Treat
eFigure 5. Adjusted Hazard Ratios of End Points by Intention-to-Treat Among Patients With Different Numbers of NMRRFs
eFigure 6. Propensity Score–Weighted Hazard Ratios of End Points by Intention-to-Treat Among Patients With Different Numbers of NMRRFs
eFigure 7. Quality-of-Life Outcomes by MAFSI Severity Scoring
eTable 1. Emulated Target Trial Framework for Prognosis Among Patients With AF and Fewer Than 3 NMRRFs
eTable 2. Emulated Target Trial Framework for Prognosis Among Patients With AF and 3 or More NMRRFs

This supplemental material has been provided by the authors to give readers additional information about their work.

© 2025 Wang Z et al. *JAMA Network Open*.

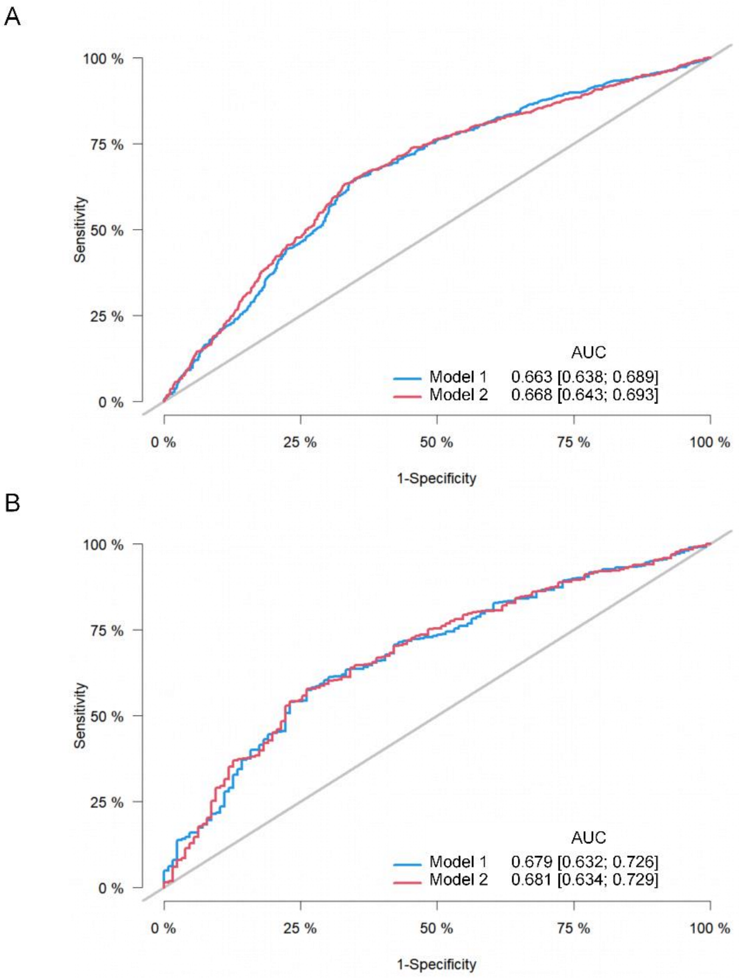

eFigure 1. Time-Dependent ROC Curve Analysis of Different Recurrence Prediction Models

A: ROC curve of AF recurrence in 12 months. B: ROC curve of AF recurrence in 48 months.

Model 1 includes the treatment group and four NMRRFs (AF duration >1 year, persistent or long-standing persistent atrial fibrillation, age >65 years, and female sex).

Model 2 includes modifiable risk factors (body mass index, alcohol consumption, smoking, diabetes, hypertension, heart failure, and sleep apnea) and all factors in Model 1.

AF=atrial fibrillation, AUC=area under the curve, ROC=receiver operating characteristic, NMRRFs=non-modifiable recurrence risk factors.

© 2025 Wang Z et al. *JAMA Network Open*.

AF=atrial fibrillation, AUC=area under the curve, ROC=receiver operating characteristic, NMRRFs=non-modifiable recurrence risk factors.

© 2025 Wang Z et al. *JAMA Network Open*.

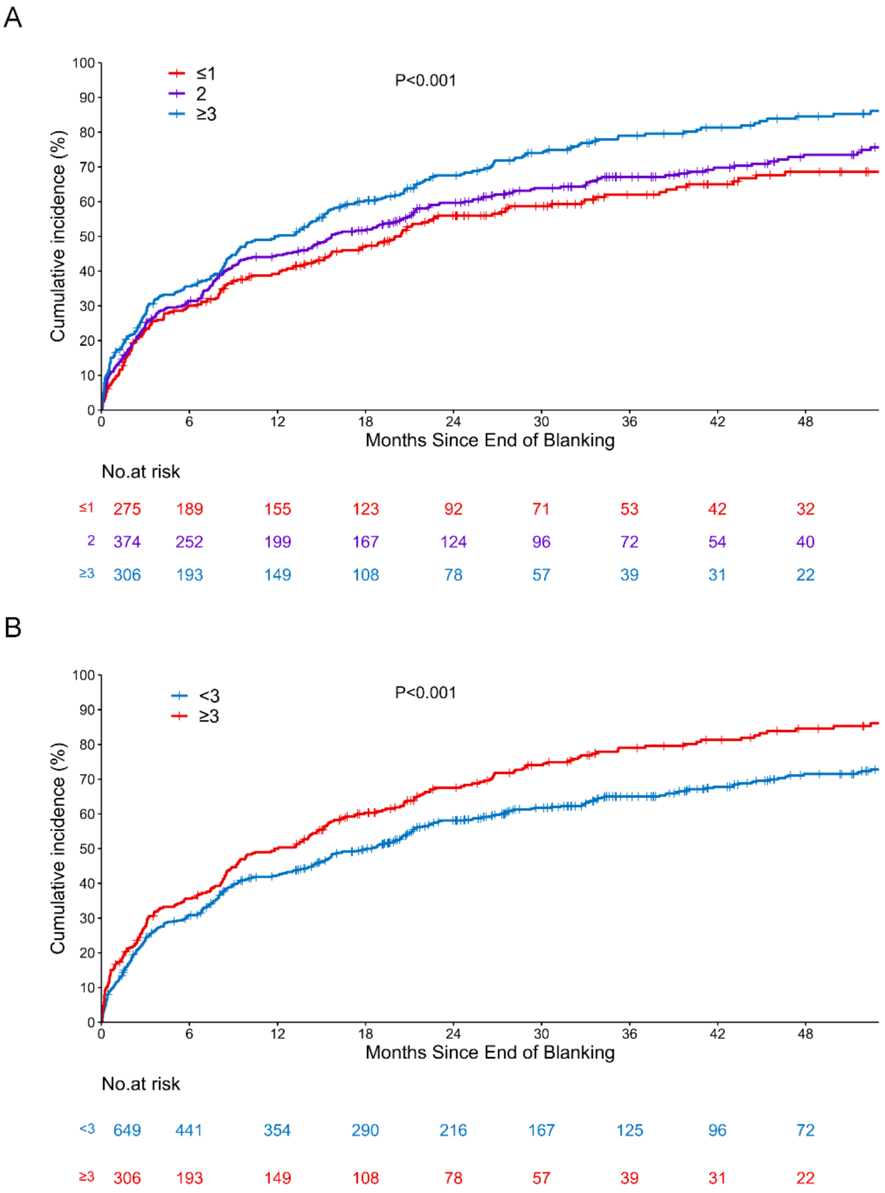

eFigure 2. Kaplan-Meier Curves for AF Recurrence in the Ablation Arm

A: Kaplan–Meier curves for AF recurrence among patients with different numbers of NMRRFs.

B: Kaplan–Meier curves for AF recurrence among patients with fewer than three NMRRFs and patients with three or more NMRRFs.

Non-modifiable recurrence risk factors included AF duration >1 year, persistent or long-standing persistent atrial fibrillation, age >65 years, and female sex.

AF=atrial fibrillation, NMRRFs=non-modifiable recurrence risk factors.

© 2025 Wang Z et al. *JAMA Network Open*.

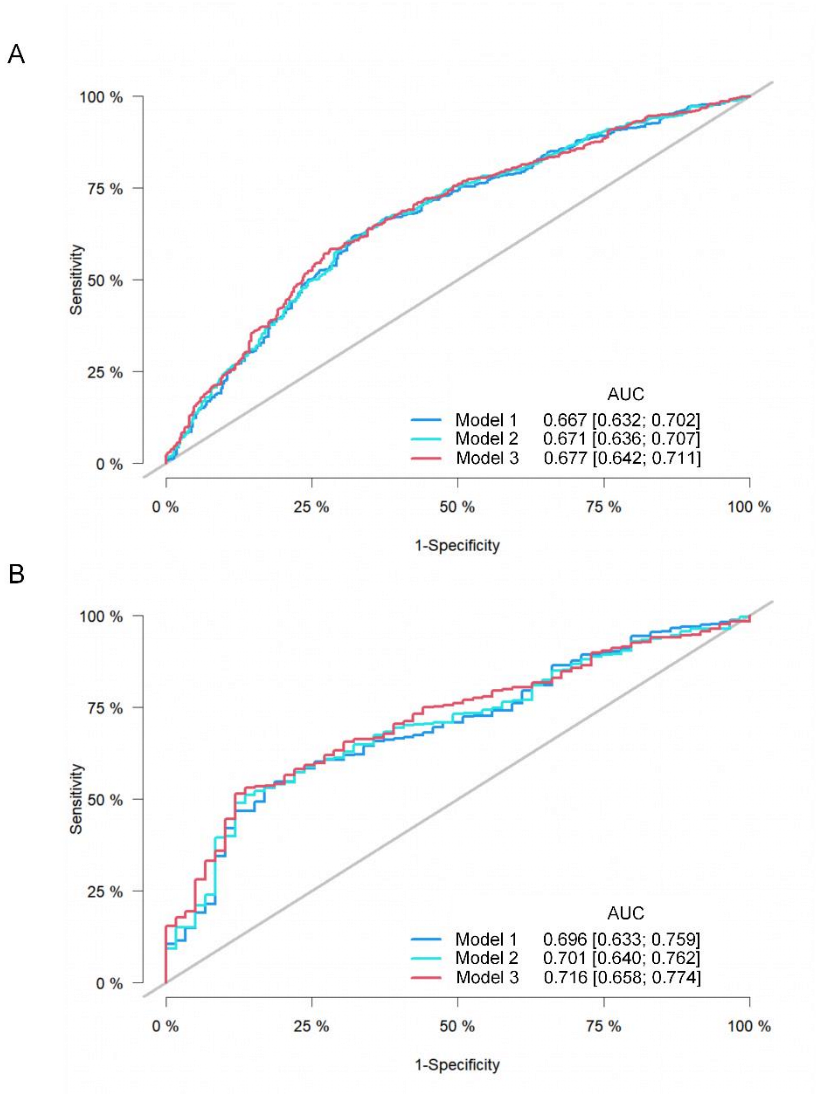

eFigure 3. Time-Dependent ROC Curve Analysis of Different Recurrence Prediction Models in Patients With Echocardiograms

A: ROC curve of AF recurrence in 12 months. B: ROC curve of AF recurrence in 48 months. Model 1 includes treatment group, AF duration >1 year, persistent or long-standing persistent atrial fibrillation, age >65 years, and female sex. Model 2 includes left atrial diameter >45 mm and all factors in Model 1. Model 3 includes modifiable risk factors (body mass index, alcohol consumption, smoking, diabetes, hypertension, heart failure, and sleep apnea) and all factors in Model 2.

© 2025 Wang Z et al. *JAMA Network Open*.

Outcome	Number of event/N(%) Drug	Ablation	HR (95%CI)	p value
**Primary Endpoint**				
< 3 NMRRFs	73/722 (10.1%)	46/709 (6.5%)	0.63 (0.43 - 0.91)	0.01
≥ 3 NMRRFs	25/339 (7.4%)	39/356 (11.0%)	1.44 (0.86 - 2.39)	0.17
**Death**				
< 3 NMRRFs	46/722 (6.4%)	32/709 (4.5%)	0.70 (0.45 - 1.11)	0.13
≥ 3 NMRRFs	18/339 (5.3%)	23/356 (6.5%)	1.19 (0.64 - 2.23)	0.58
**AF recurrence**				
< 3 NMRRFs	590/676 (87.3%)	411/630 (65.2%)	0.47 (0.42 - 0.54)	<0.001
≥ 3 NMRRFs	281/316 (88.9%)	232/300 (77.3%)	0.58 (0.48 - 0.69)	<0.001

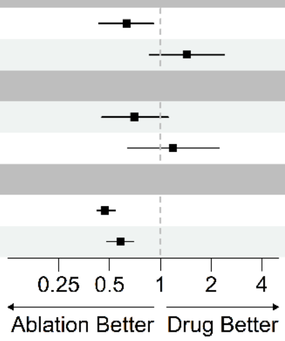

eFigure 4. Propensity Score–Weighted Hazard Ratios of End Points by Intention-to-Treat

Non-modifiable recurrence risk factors included AF duration >1 year, persistent or long-standing persistent atrial fibrillation, age >65 years, and female sex.

AF=atrial fibrillation, HR= hazard ratio, CI=confidence interval, NMRRFs=non-modifiable recurrence risk factors.

© 2025 Wang Z et al. *JAMA Network Open*.

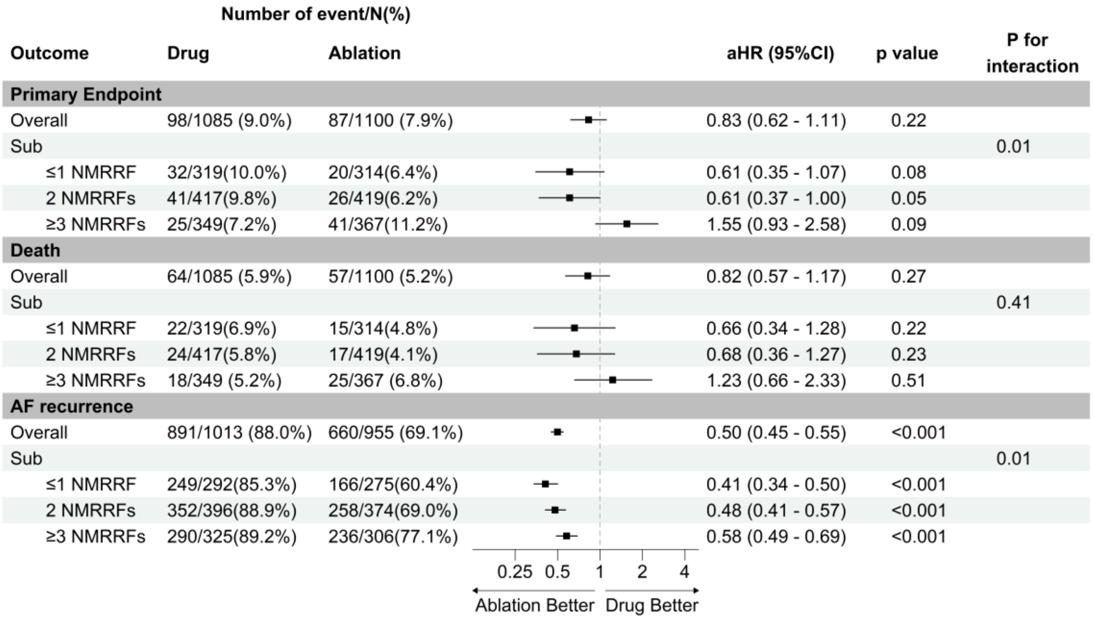

eFigure 5. Adjusted Hazard Ratios of End Points by Intention-to-Treat Among Patients With Different Numbers of NMRRFs

Non-modifiable recurrence risk factors included AF duration >1 year, persistent or long-standing persistent atrial fibrillation, age >65 years, and female sex.

AF=atrial fibrillation, aHR=adjusted hazard ratio, CI=confidence interval, NMRRFs=non-modifiable recurrence risk factors.

© 2025 Wang Z et al. *JAMA Network Open*.

Outcome	Number of event/N(%) Drug	Ablation	HR (95%CI)	p value
**Primary Endpoint**				
≤1 NMRRF	32/313 (10.2%)	20/308 (6.5%)	0.69 (0.39 - 1.22)	0.20
2 NMRRFs	41/409 (10.0%)	26/401 (6.5%)	0.60 (0.36 - 0.98)	0.04
≥ 3 NMRRFs	25/339 (7.4%)	39/356 (11.0%)	1.44 (0.86 - 2.39)	0.17
**Death**				
≤1 NMRRF	22/313 (7.0%)	15/314 (4.9%)	0.80 (0.41 - 1.56)	0.51
2 NMRRFs	24/409 (5.9%)	17/401 (4.2%)	0.71 (0.38 - 1.33)	0.29
≥ 3 NMRRFs	18/339 (5.3%)	23/356 (6.5%)	1.19 (0.64 - 2.23)	0.58
**AF recurrence**				
≤1 NMRRF	244/287 (85.0%)	164/271 (60.5%)	0.44 (0.36 - 0.54)	<0.001
2 NMRRFs	346/389 (88.9%)	247/359 (68.8%)	0.49 (0.41 - 0.58)	<0.001
≥ 3 NMRRFs	281/316 (88.9%)	232/300 (77.3%)	0.58 (0.48 - 0.69)	<0.001

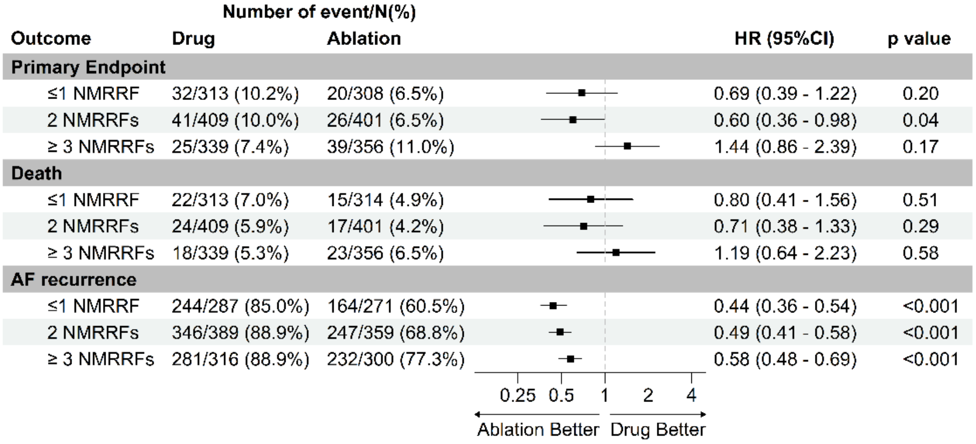

eFigure 6. Propensity Score–Weighted Hazard Ratios of End Points by Intention-to-Treat Among Patients With Different Numbers of NMRRFs

Non-modifiable recurrence risk factors included AF duration >1 year, persistent or long-standing persistent atrial fibrillation, age >65 years, and female sex.

AF=atrial fibrillation, HR= hazard ratio, CI=confidence interval, NMRRFs=non-modifiable recurrence risk factors.

© 2025 Wang Z et al. *JAMA Network Open*.

Follow up	Number of Patients: Drug	Ablation	Adjusted mean difference	p value
**< 3 NMRRFs**				
Baseline	708	708	-0.14 (-0.65, 0.37)	0.59
3	595	593	-1.30 (-1.83, -0.76)	<0.001
12	542	544	-1.59 (-2.14, -1.04)	<0.001
24	482	505	-1.62 (-2.19, -1.05)	<0.001
36	373	383	-1.29 (-1.92, -0.66)	<0.001
48	292	286	-0.84 (-1.53, -0.15)	0.02
60	198	182	-1.25 (-2.06, -0.44)	0.003
All	2482	2493	-1.27 (-1.72, -0.83)	<0.001
**≥ 3 NMRRFs**				
Baseline	343	355	0.06 (-0.68, 0.80)	0.88
3	291	300	-1.35 (-2.14, -0.57)	0.001
12	281	280	-1.48 (-2.27, -0.68)	<0.001
24	239	254	-0.76 (-1.59, 0.07)	0.07
36	182	186	-0.59 (-1.50, 0.33)	0.21
48	123	136	-0.46 (-1.50, 0.58)	0.39
60	95	96	-0.87 (-2.04, 0.30)	0.15
All	1211	1252	-1.00 (-1.68, -0.32)	0.004

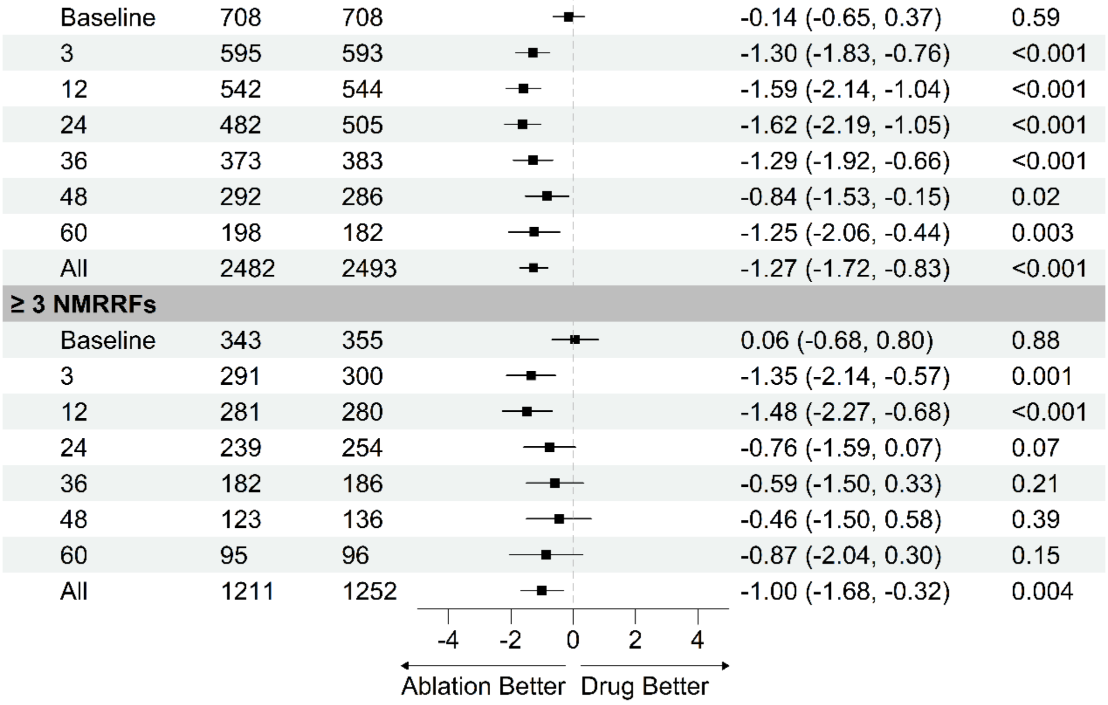

eFigure 7. Quality-of-Life Outcomes by MAFSI Severity Scoring

Non-modifiable recurrence risk factors included AF duration >1 year, persistent or long-standing persistent atrial fibrillation, age >65 years, and female sex.

MAFSI=Mayo Atrial Fibrillation-Specific Symptom Inventory, NMRRFs=non-modifiable recurrence risk factors.

© 2025 Wang Z et al. *JAMA Network Open*.

eTable 1. Emulated Target Trial Framework for Prognosis Among Patients With AF and Fewer Than 3 NMRRFs

Protocol Component	Target Trial	Emulated Trial
Aim	To evaluate the risk of a composite outcomes (including all-cause mortality, disabling stroke, major bleeding, or cardiac arrest) among patients with AF who have fewer than three NMRRFs.	Same as for the target trial.
Eligibility Criteria	● Aged 65 years and older or younger than 65 years with 1 or more risk factors for stroke (hypertension, heart failure, history of stroke, diabetes, or other heart problems).<br>● Had 2 or more episodes of paroxysmal AF or 1 episode of persistent AF in the prior 6 months.<br>● Suitable for catheter-based treatment or rhythm and/or rate control drug therapy.<br>● Had fewer than 3 NMRRFs (AF duration >1 year, persistent or long-standing persistent AF, age > 65, and female sex).<br>● Without a prior left atrial catheter ablation for AF.<br>● Without a history of failure of two or more antiarrhythmic drugs.	Same as for the target trial.
Treatment Strategies	1. Catheter ablation<br>Ablation procedures all included pulmonary vein isolation. The addition of ancillary ablation techniques, were left to the discretion of the investigators.<br>2. Drug therapy<br>Patients randomized to drug therapy receive rate control medications first. If the patient had previously failed rate control therapy, then rhythm control drug therapy could be initiated in an approach consistent with contemporaneous guidelines.	Same as for the target trial.
Treatment Assignment	Individuals are randomly assigned and are aware of the treatment to which they have been assigned.	1. The CABANA trial did not use stratified randomization based on the number of NMRRFs. But baseline characteristics were similar between the ablation and drug therapy groups.<br>2. The results were adjusted for a pre-specified set of baseline patient characteristics (age, sex, race, AF

© 2025 Wang Z et al. *JAMA Network Open*.

Protocol Component	Target Trial	Emulated Trial
		type, AF duration, history of heart failure, structural heart disease, CHA_2DS_2-VASc score, history of coronary artery disease, and hypertension).<br>3. We also used propensity score weighting (overlap weighting) to strengthen covariate balance and address potential confounding.
Follow-up	Starts at the time of grouping and ends on the day of death, disabling stroke, serious bleeding, cardiac arrest, loss of follow-up, or the end of the study period (December 31, 2017).	Same as for the target trial.
Outcomes	Primary endpoint: a composite of death, disabling stroke, serious bleeding, or cardiac arrest.<br>Secondary endpoints: all-cause mortality, recurrence of atrial fibrillation.	Same as for the target trial.
Causal Contrasts	Intention-to-treat effect.	Same as for the target trial.
Statistical Analysis	Comparison of the rates and adjusted hazard ratios, among individuals assigned to each treatment strategy.	Same as for the target trial.

AF=atrial fibrillation. NMRRFS=non-modifiable recurrence risk factors.

© 2025 Wang Z et al. *JAMA Network Open*.

eTable 2. Emulated Target Trial Framework for Prognosis Among Patients With AF and 3 or More NMRRFs

Protocol Component	Target Trial	Emulated Trial
Aim	To evaluate the risk of a composite outcomes (including all-cause mortality, disabling stroke, major bleeding, or cardiac arrest) among patients with AF who have three or more NMRRFs.	Same as for the target trial.
Eligibility Criteria	● Aged 65 years and older or younger than 65 years with 1 or more risk factors for stroke (hypertension, heart failure, history of stroke, diabetes, or other heart problems).<br>● Had 2 or more episodes of paroxysmal AF or 1 episode of persistent AF in the prior 6 months.<br>● Suitable for catheter-based treatment or rhythm and/or rate control drug therapy.<br>● Had three or more NMRRFs (AF duration >1 year, persistent or long-standing persistent AF, age > 65, and female sex).<br>● Without a prior left atrial catheter ablation for AF.<br>● Without a history of failure of two or more antiarrhythmic drugs.	Same as for the target trial.
Treatment Strategies	1. Catheter ablation<br>Ablation procedures all included pulmonary vein isolation. The addition of ancillary ablation techniques were left to the discretion of the investigators.<br>2. Drug therapy<br>Patients randomized to drug therapy receive rate control medications first. If the patient had previously failed rate control therapy, then rhythm control drug therapy could be initiated in an approach consistent with contemporaneous guidelines.	Same as for the target trial.
Treatment Assignment	Individuals are randomly assigned and are aware of the treatment to which they have been assigned.	1. The CABANA trial did not use stratified randomization based on the number of NMRRFs. But baseline characteristics were similar between the ablation and drug therapy groups.<br>2. The results were adjusted for a pre-specified set of baseline patient characteristics (age, sex, race, AF type, AF duration, history of heart failure, structural heart disease,

© 2025 Wang Z et al. *JAMA Network Open*.

Protocol Component	Target Trial	Emulated Trial
		CHA_2DS_2-VASc score, history of coronary artery disease, and hypertension).<br>3. We also used propensity score weighting (overlap weighting) to further strengthen covariate balance and address potential confounding.
Follow-up	Starts at the time of grouping and ends on the day of death, disabling stroke, serious bleeding, cardiac arrest, loss of follow-up, or the end of the study period (December 31, 2017).	Same as for the target trial.
Outcomes	Primary endpoint: a composite of death, disabling stroke, serious bleeding, or cardiac arrest.<br>Secondary endpoints: all-cause mortality, recurrence of atrial fibrillation.	Same as for the target trial.
Causal Contrasts	Intention-to-treat effect.	Same as for the target trial.
Statistical Analysis	Comparison of the rates and adjusted hazard ratios, among individuals assigned to each treatment strategy.	Same as for the target trial.

AF=atrial fibrillation. NMRRFS=non-modifiable recurrence risk factors.

© 2025 Wang Z et al. *JAMA Network Open*.
