# Supplementary material for: Catheter Ablation vs Drug Therapy in Patients With Atrial Fibrillation and Nonmodifiable Recurrence Risk Factors: A Secondary Analysis of the CABANA Randomized Clinical Trial
Source: JAMA Netw Open. 2025 Aug 21;8(8):e2528124. doi: 10.1001/jamanetworkopen.2025.28124 (PMC12371518; doi:10.1001/jamanetworkopen.2025.28124)
Supplement: Supplement 3. — Data Sharing Statement [file jamanetwopen-e2528124-s003.pdf]

Data Sharing Statement

Wang. Catheter Ablation vs Drug Therapy in Patients With Atrial Fibrillation and Nonmodifiable Recurrence Risk Factors. *JAMA Netw Open*. Published August 21, 2025.
doi:10.1001/jamanetworkopen.2025.28124

Data
Additional Information: Catheter Ablation vs Anti-arrhythmic Drug Therapy for Atrial Fibrillation Trial (CABANA) ClinicalTrials.gov ID: NCT00911508 URL: https://clinicaltrials.gov/study/NCT00911508
Data available: Yes
Data types: Deidentified participant data
How to access data: https://biolincc.nhlbi.nih.gov/studies/cabana/
When available: beginning date: 07-28-2023

Supporting Documents
Document types: None

Additional Information
Who can access the data: researchers whose proposed use of the data has been approved
Types of analyses: for any purpose
Mechanisms of data availability: after approval of a proposal
